# The Biodiversity of the Mediterranean Sea: Estimates, Patterns, and Threats

**DOI:** 10.1371/journal.pone.0011842

**Published:** 2010-08-02

**Authors:** Marta Coll, Chiara Piroddi, Jeroen Steenbeek, Kristin Kaschner, Frida Ben Rais Lasram, Jacopo Aguzzi, Enric Ballesteros, Carlo Nike Bianchi, Jordi Corbera, Thanos Dailianis, Roberto Danovaro, Marta Estrada, Carlo Froglia, Bella S. Galil, Josep M. Gasol, Ruthy Gertwagen, João Gil, François Guilhaumon, Kathleen Kesner-Reyes, Miltiadis-Spyridon Kitsos, Athanasios Koukouras, Nikolaos Lampadariou, Elijah Laxamana, Carlos M. López-Fé de la Cuadra, Heike K. Lotze, Daniel Martin, David Mouillot, Daniel Oro, Saša Raicevich, Josephine Rius-Barile, Jose Ignacio Saiz-Salinas, Carles San Vicente, Samuel Somot, José Templado, Xavier Turon, Dimitris Vafidis, Roger Villanueva, Eleni Voultsiadou

**Affiliations:** 1 Institut de Ciències del Mar, Scientific Spanish Council (ICM-CSIC), Barcelona, Spain; 2 Biology Department, Dalhousie University, Halifax, Canada; 3 Fisheries Center - Aquatic Ecosystems Research Laboratory, University of British Columbia, Vancouver, Canada; 4 Evolutionary Biology & Ecology Lab, Albert-Ludwigs-University, Freiburg, Germany; 5 Laboratoire Ecosystèmes Lagunaires UMR 5119, Université Montpellier 2, Montpellier, France; 6 Laboratoire Ecosystèmes & Ressources Aquatiques UR03AGRO1, Institut National Agronomique de Tunisie, Tunis, Tunisia; 7 Centre d'Estudis Avançats de Blanes, Scientific Spanish Council (CEAB-CSIC), Blanes, Spain; 8 Dipartimento per lo studio del Territorio e delle sue Risorse, Università di Genova, Genova, Italy; 9 Carrer Gran, Argentona, Spain; 10 Department of Zoology, Aristoteleio University of Thessaloniki, Thessaloniki, Greece; 11 Hellenic Centre for Marine Research, Institute of Marine Biology and Genetics, Heraklion, Greece; 12 Dipartimento Scienze del Mare, Polytechnic University of Marche, Ancona, Italy; 13 Istituto di Scienze Marine, Consiglio Nazionale dell Ricerche, Ancona, Italy; 14 National Institute of Oceanography, Israel Oceanographic and Limnological Research, Haifa, Israel; 15 Haifa University and Oranim Academic College, Haifa, Israel; 16 The WorldFish Center, Philippine Office, Los Baños, Philippines; 17 Hellenic Centre for Marine Research, Institute of Oceanography, Heraklion, Greece; 18 Laboratorio de Biología Marina - Departamento de Fisiología y Zoología, Universidad de Sevilla, Sevilla, Spain; 19 Mediterranean Institute for Advanced Studies, Scientific Spanish Council (IMEDEA-CSIC), Esporles, Spain; 20 Istituto Superiore per la Ricerca e la Protezione Ambientale, Chioggia, Italy; 21 Zoology Department, University of the Basque Country, Bilbao, Spain; 22 Carrer Nou, Creixell, Spain; 23 Météo-France, Centre National de Recherches Météorologiques, Toulouse, France; 24 Museo Nacional de Ciencias Naturales, Scientific Spanish Council (MNCN-CSIC), Madrid, Spain; 25 Department of Ichthyology and Aquatic Environment, University of Thessaly, Nea Ionia, Greece; NOAA/NMFS/SWFSC, United States of America

## Abstract

The Mediterranean Sea is a marine biodiversity hot spot. Here we combined an extensive literature analysis with expert opinions to update publicly available estimates of major taxa in this marine ecosystem and to revise and update several species lists. We also assessed overall spatial and temporal patterns of species diversity and identified major changes and threats. Our results listed approximately 17,000 marine species occurring in the Mediterranean Sea. However, our estimates of marine diversity are still incomplete as yet—undescribed species will be added in the future. Diversity for microbes is substantially underestimated, and the deep-sea areas and portions of the southern and eastern region are still poorly known. In addition, the invasion of alien species is a crucial factor that will continue to change the biodiversity of the Mediterranean, mainly in its eastern basin that can spread rapidly northwards and westwards due to the warming of the Mediterranean Sea. Spatial patterns showed a general decrease in biodiversity from northwestern to southeastern regions following a gradient of production, with some exceptions and caution due to gaps in our knowledge of the biota along the southern and eastern rims. Biodiversity was also generally higher in coastal areas and continental shelves, and decreases with depth. Temporal trends indicated that overexploitation and habitat loss have been the main human drivers of historical changes in biodiversity. At present, habitat loss and degradation, followed by fishing impacts, pollution, climate change, eutrophication, and the establishment of alien species are the most important threats and affect the greatest number of taxonomic groups. All these impacts are expected to grow in importance in the future, especially climate change and habitat degradation. The spatial identification of hot spots highlighted the ecological importance of most of the western Mediterranean shelves (and in particular, the Strait of Gibraltar and the adjacent Alboran Sea), western African coast, the Adriatic, and the Aegean Sea, which show high concentrations of endangered, threatened, or vulnerable species. The Levantine Basin, severely impacted by the invasion of species, is endangered as well.

This abstract has been translated to other languages (File S1).

## Introduction

**Figure 1:**
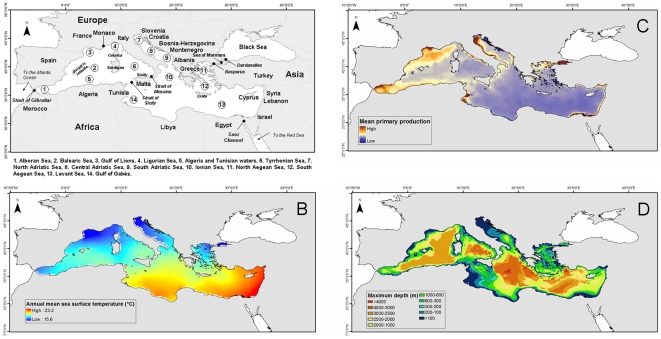
Biogeographic regions and oceanographic features of the Mediterranean Sea. (A) Main biogeographic regions, basins, and administrative divisions of the Mediterranean Sea, (B) Annual mean sea surface temperature (°C) (2003, NOAA), (C) Annual mean relative primary production (2002, Inland and Marine Waters Unit, Institute for Environment and Sustainability, EU Joint Research Centre, Ispra, Italy), and (D) maximum average depth (m) (NOAA).

The *Mare medi terraneum* (in Latin) describes the Mediterranean as a “sea in the middle of the land.” This basin is the largest (2,969,000 km^2^) and deepest (average 1,460 m, maximum 5,267 m) enclosed sea on Earth (Figure 1a).

Situated at the crossroads of Africa, Europe, and Asia, the Mediterranean coasts have witnessed the flourishing and decline of many civilizations. The region was an important route for merchants and travelers of ancient times, allowing for trade and cultural exchange, and today it is notable for contributions to global economy and trade. Its coasts support a high density of inhabitants, distributed in 21 modern states, and it is one of the top tourist destinations in the world, with 200 million tourists per year [1].

The Mediterranean Sea connects through the Strait of Gibraltar to the Atlantic Ocean in the west and through the Dardanelles to the Sea of Marmara and the Black Sea in the northeast. In the southeast, the Suez Canal links the Mediterranean to the Red Sea and the Indian Ocean (Figure 1a). In the Strait of Sicily, a shallow ridge at 400 m depth separates the island of Sicily from the coast of Tunisia and divides the sea into two main subregions: the western (area  = 0.85 million km^2^) and the eastern (area = 1.65 million km^2^).

General oceanographic conditions in the Mediterranean have been previously described in detail [e.g., [2]–[5]]. It is a concentration basin: evaporation is higher in its eastern half, causing the water level to decrease and salinity to increase from west to east. The resulting pressure gradient pushes relatively cool, low-salinity water from the Atlantic across the Mediterranean basin. This water warms up to the east, where it becomes saltier and then sinks in the Levantine Sea before circulating west and exiting through the Strait of Gibraltar. The climate in the region is characterized by hot, dry summers and cool, humid winters. The annual mean sea surface temperature shows a high seasonality and important gradients from west to east and north to south (Figure 1b) [3]. The basin is generally oligotrophic, but regional features enrich coastal areas through changing wind conditions, temporal thermoclines, currents and river discharges, and municipal sewage [6], [7], [8] (Figure 1c). The basin is characterized by strong environmental gradients [9], in which the eastern end is more oligotrophic than the western. The biological production decreases from north to south and west to east and is inversely related to the increase in temperature and salinity.

The Mediterranean has narrow continental shelves and a large area of open sea. Therefore, a large part of the Mediterranean basin can be classified as deep sea (Figure 1d) and includes some unusual features: (1) high homothermy from 300–500 m to the bottom, where temperatures vary from 12.8°C–13.5°C in the western basin to 13.5°C–15.5°C in the eastern, and (2) high salinity of 37.5–39.5 psu. Unlike in the Atlantic Ocean, where temperature decreases with depth, there are no thermal boundaries in the deep sea of the Mediterranean [10]. Shelf waters represent 20% of the total Mediterranean waters, compared with the 7.6% of the world oceans, and therefore play a proportionally greater role here than in the world's oceans [4]. Shelves in the south are mainly narrow and steep (e.g., Moroccan, Algerian, and Libyan coasts, with the exception of the Gulf of Gabés), while those in the north are wider (e.g., the north and central Adriatic Sea, the Aegean Sea, and the Gulf of Lions) [4] (Figure 1d). These features influence the morphology and constrain the connections to the Atlantic, the Red Sea, and the Indian Ocean [3], [11].

The enclosed Mediterranean had a varied geological history, including isolation from the world ocean, that led to its near drying out during the Messinian crisis (5.96 million years ago) and to drastic changes in climate, sea level, and salinity [12], [13]. The geological history, biogeography, ecology, and human history have contributed to the Mediterranean's high cultural and biological diversity [14]–[17].

The recent marine biota in the Mediterranean Sea is primarily derived from the Atlantic Ocean, but the wide range of climate and hydrology have contributed to the co-occurrence and survival of both temperate and subtropical organisms [18], [19]. High percentages of Mediterranean marine species are endemic [16], [20]. This sea has as well its own set of emblematic species of conservation concern, such as sea turtles, several cetaceans, and the critically endangered Mediterranean monk seal (*Monachus monachus*). It is the main spawning grounds of the eastern Atlantic bluefin tuna (*Thunnus thynnus*) [e.g., [21]–[25]]. There are several unique and endangered habitats, including the seagrass meadows of the endemic *Posidonia oceanica*, vermetid reefs built by the endemic gastropod *Dendropoma petraeum*, coralligenous assemblages [e.g., [26]–[29]], and deep-sea and pelagic habitats that support unique species and ecosystems [e.g., [30]–[32]]. Many sensitive habitats exist within the coastal ecosystems. There are 150 wetlands of international importance for marine and migrating birds, and some 5,000 islands and islets [33]–[35].

The region has numerous laboratories, universities, and research institutes dedicated to exploring the sea around them [e.g., [36]]. In addition to the unique geologic, biogeographic, physical, and ecological features, our current understanding of the high biodiversity of the Mediterranean Sea is built on the long tradition of study dating from the times of the Greeks and Romans. Historical documentation began with Aristotle, who contributed to the classification and description of marine biodiversity, and was followed by the work of Plinius (Historia naturalis, liber IX) in the first century B.C., Carl von Linné in the eighteenth century, and many others to the middle of the nineteenth century [e.g., [37]–[40]]. The first deep-sea investigations began at the end of the nineteenth century [e.g., [41]–[43]]. The expeditions of the R.V. “Calypso” by Jacques-Yves Cousteau in the Mediterranean during the 1950s and 1960s provided as well valuable material that supported many important publications on the Mediterranean diversity. The history of ecological research and species discovery in the region has been thoroughly reviewed by Riedl [44], Margalef [45], and Hofrichter [46], though mostly confined to the western Mediterranean.

Numerous detailed taxonomic inventories now exist, most of which are specific to sub-regions or to a range of organisms [e.g., [47]–[56], among many others]. Efforts continue to provide complete datasets of taxonomic groups for the entire basin [e.g., [57]–[67]], although they need periodic updates. Freely available databases for macroorganism inventory include the Medifaune database [68], the Food and Agriculture Organization Species Identification Field Guide for Fishery Purposes [69], the FNAM (Fishes of the North-Eastern Atlantic and the Mediterranean) atlas [70], and the ICTIMED database [71].

However, Web-based datasets often lack updates because of limitations in funding or expertise, and in general, the marine biodiversity of the Mediterranean is less known than its terrestrial counterpart [33], [72]. There are still important gaps at population, community, habitat, and sub-region levels, as well as in basic information about taxonomy distribution, abundance, and temporal trends of several groups [72], [73]. In some areas biodiversity data exist, but it is not easily accessible, because the inventories are not publicly available [74]. Data are also lacking to evaluate the conservation status of many species [34].

The Mediterranean region has been inhabited for millennia, and ecosystems have been altered in many ways [e.g., [5],[16],[45],[75]]. Therefore, impacts of human activities are proportionally stronger in the Mediterranean than in any other sea of the world [33].

Therefore, combined natural and anthropogenic events shaped the biodiversity of the Mediterranean Sea in the past and are likely to continue to do so. Within this complex framework, our aims were threefold:

1. Review available estimates of Mediterranean marine biodiversity, including new estimates of less conspicuous organisms, updating previous checklists, and incorporating living organisms from microbes to top predators.
2. Describe the main spatial and temporal patterns of biodiversity, including innovative ways of describing these patterns.
3. Summarize the main drivers of change and threats to marine biodiversity.

We have collated available information, generated coherent patterns, and identified the current state of knowledge and information gaps, challenges, and prospects for future research. We embrace the concept of biodiversity in its broader definition as the variation of life at all levels of biological organization, but we have focused our efforts on documenting species-level diversity.

## Methods

### Diversity estimates

#### Total estimates of biodiversity

We used our updated taxonomic estimates of species diversity to revise the total estimate of Mediterranean marine biodiversity and to compare it with previous studies [16], [19], [68]. We assessed online data availability by comparing these estimates with global and regional datasets that store an important portion of Mediterranean information, including the World Register of Marine Species database (WoRMS), Marbef Data System (European Register of Marine Species, ERMS) and the Ocean Biogeographic Information System (OBIS), FishBase and SeaLifeBase, AquaMaps, and ICTIMED [71], [76]–[81]. We also calculated the percentage that Mediterranean species of macrophytes and metazoans make up of their global counterpart, by comparing our estimates with global number of marine species according to Bouchet [82] and Green and Short [26] for flowering plants, and Groombridge and Jenkins [83] for other Vertebrata species.

#### Estimates by taxonomic group

We combined an extensive literature analysis with expert opinions to update publicly available estimates of major taxa and to revise and update several species lists. While most of this information has been incorporated into the supporting materials (File S2), here we present detailed summaries of the diversity of some specific groups inhabiting either the extreme ends of the food web (microbes and predators) or the deep-sea environment that represents the most prevalent habitat type in the Mediterranean Sea. In addition, we provide an overview of the newly introduced species. We also identified information gaps by taxonomic group and assessed species discoveries over time for several taxa to visualize the rates of diversity description.

Table 1 and File S2 summarize specific information for each taxonomic group for which such analysis is possible, and File S2 lists the experts contributing to this synthesis. File S2 also lists several experts and taxonomic guides by taxa, although it is not an exhaustive list of experts by taxonomic group in the Mediterranean Sea. File S2 provides methodological specifications and the detailed taxonomic review of several groups too, as well as revised checklists, detailed references, and additional information.

**Table 1:** Taxonomic classification of species reported in the Mediterranean Sea (File S2 for details).

Taxonomic group	No. species^1^	State of knowledge	No. introduced species	No. experts^2^	No. identification guides and key references^3^
**Domain Archaea**	Unknown	Very limited		3	
**Domain Bacteria**	Unknown (165 macroscopically identifiable cyanobacteria described)	Very limited/2		5	7
**(including Cyanobacteria)**					
**Domain Eukarya**					
**Protoctista and Chromista**	Unknown, first estimate approx. 4400^4^	Very limited/3–4	23	24	25
Dinomastigota (Dinoflagellata)	673	4			2
Bacillariophyceae	736	4			1
Coccolithophores	166	4			1
Foraminifera	>600	Benthic and planktonic/3			5
Heterokontophyta	277	3	23	19	1+ File S2
**Plantae ^5^**	854	New species being described and reclassified/4	90	35	3+ File S2
Chlorophyta	190 (180^6^)	4	17		File S2
Rhodophyta	657	4	73		File S2
Magnoliophyta	7	5	1		File S2
**Animalia**	11595		512		
Porifera	681	Well known except southern areas and the Levantine Sea/4		6	5
Cnidaria	757	Limited/4	3	11	7+ File S2
Platyhelminthes	1000	Very limited/3		6	1
Mollusca	2113	Well known, but new species being described/4	Approx. 200	19	4+ File S2
Annelida	1172	New species being described/5	70–80	>28	5+ File S2
Crustacea	2239	New species being described/3–4	106	34	25+ File S2
Bryozoa	388	Limited/4	1	7	7+ File S2
Echinodermata	154	Lack of data in southern and deeper areas/5	5	3	2+ File S2
Tunicata (Ascidiacea)	229	Limited/4	15	8	6+ File S2
Other invertebrates	2168	Limited/3–4	2	17	15+ File S2
Vertebrata (Pisces)	650	Well known, except few rare species recorded sporadically/5	116 (91)	13	10+ File S2
Other vertebrates	43	Well known for mammals, reptiles and birds/5		12	12+ File S2
**SUBTOTAL**					
**TOTAL REGIONAL DIVERSITY^3^**	**16848**		**626** *		

***State of knowledge:*** 5 =  very well known (>80% described, identification guides <20 years old, and current taxonomic expertise); 4 =  well known (>70% described, identification guides <50 years old, some taxonomic expertise), 3 =  poorly known (<50% species described, identification guides old or incomplete, no present expertise within region), 2 =  very poorly known (only few species recorded, no identification guides, no expertise), 1 =  unknown (no species recorded, no identification guides, no expertise). ND  =  No data. Number of experts and number of identification guides correspond to the list provided in File S2, listing several experts and taxonomic guides by taxa, although this is not an exhaustive list of experts by taxonomic group in the Mediterranean Sea. (1) Sources: databases, scientific literature, books, field guides, technical reports (see File S2); (2) N° of experts provided in File S2, listing several experts by taxa, although this is not an exhaustive list of experts by taxonomic group in the Mediterranean Sea; (3) Identification guides cited in File S2; (4) This number is highly uncertain (see text section The biodiversity of the “smallest”); (5) corresponding to macrophytobenthos; (6) 10 species reported within the Chlorophyceae (Volvocales) and Prasinophyceae (Chlorodendrales, Pyramimonadales) are unicellular and can be considered to be phytoplanktonic, although they thrive in mediolittoral and supralittoral pools and have been classically included in the checklists of marine macroalgae.

*This estimate is continuously increasing and may be as high as 1,000 species if unicellular aliens and foraminiferans are included [e.g., [206],[207],[208]].

To classify the estimates of organisms, we followed the taxonomic classification by WoRMS [76]. This classification is followed in the other regional syntheses of marine diversity of the Census of Marine Life (Census) and enables comparison between regions. We therefore used a practical division of the Eukarya into Plantae, Animalia, Protists, and Chromists even though the current kingdom division in the eukaryotes ranges between 6 and 12 and few coincide with these traditional divisions [84]–[86]. However, we placed together Archaea and Bacteria because little information exists for either of these divisions.

Our review included only generic information on prokaryotic (Bacteria and Archaea) and eukaryotic (Protists) marine microbes and detailed quantification of diversity of a few groups, such as seaweeds and seagrasses (a phylogenetically heterogeneous group of eukaryotic photosynthetic organisms) and metazoans (invertebrates and vertebrates). Within Animalia, we especially focused on the phyla Porifera, Cnidaria (with emphasis on benthic forms), Mollusca, Annelida (with emphasis on Polychaeta), Arthropoda (with emphasis on Decapoda, Cumacea, and Mysidacea), Bryozoa, Echinodermata, Sipuncula, some other invertebrates forming part of the meiobenthos (Nematoda, benthic Harpacticoida [Crustacea: Copepoda], benthic Foraminifera, and Gastrotricha), Tunicata (with emphasis on Ascidiacea), and the subphylum Vertebrata. We did not include the Fungi occurring in the Mediterranean Sea (which are reported to be approximately 140 species) [87].

### Depiction of patterns

#### Spatial and bathymetric patterns

To describe spatial patterns, we used published available information by region or subregions and by taxonomic group regarding sighting locations, home ranges, or general information on distribution of species in the Mediterranean Sea. We also included information on biodiversity patterns by depth, reviewing data of several taxa available in the literature.

Spatial patterns of benthic primary producers and invertebrate species were explored at the scale of large regions or basins. When available, we used detailed spatial data, mostly available in the form of expert-drawn maps or sighting locations, to map spatial patterns of vertebrate species using GIS (geographical information system) software (ArcView by ESRI). For each 0.1×0.1 degree grid cell within the Mediterranean, we estimated the species richness of different taxonomic groups as the sum of the species co-occurring by overlapping expert-drawn distribution maps. We compiled data about exotic fish species from the CIESM (The Mediterannean Science Commission) atlas [88], [89] and the paper by Quignard and Tomasini [90]. Data for other fish species were available from the FNAM atlas [70] and data compiled by Ben Rais Lasram et al. [91]. We used maps of species occurrence and sighting locations as point data to draw the distributional ranges of resident marine mammals and turtles, but we excluded nonresident or visiting species from the species richness maps. We represented the latter information as point data showing their sighting locations [22], [92]–[99]. The current distribution of Mediterranean monk seal was drawn by integrating information in recent literature [23], [100]–[107]. Information on the distribution of seabird colonies around the Mediterranean, and of Audouin's gull *Larus audouinii* in particular, was collected from different observations [108]–[111].

In our analysis, we considered those regions with uncertain or insufficient data (mainly identified by a question mark in distribution maps) as “no occurrence.” However, we recognize that the absence of data may well reflect a lack of study effort in a given area rather than actual absence of a species, and thus we used the missing data to identify regions that are insufficiently studied. Moreover, available data have been collected mainly from the 1980s to 2000s. Therefore, species richness maps generated in this study should be considered as cumulative distribution maps rather than current distributions.

We also used the global species distribution model AquaMaps [80] to generate standardized range maps of species occurrence. AquaMaps is a modified version of the relative environmental suitability (RES) model developed by Kaschner et al. [112]. This is an environmental envelope model that generates standardized range maps, within which the relative probability of occurrence for marine species is based on the environmental conditions in each 0.5×0.5 degree cell of a global grid (see specifications of Mediterranean AquaMaps in File S2). We produced AquaMaps of predicted patterns of biodiversity for different taxa in the Mediterranean by overlaying the respective subsets of the 685 available distribution maps for Mediterranean species and counting all species predicted to occur in a given cell. We assumed a species to be present in each cell for which the species-specific predicted relative probability of occurrence was greater than zero. For the prediction of marine mammal biodiversity, we used a probability threshold of species occurrence of at least 0.4 to define presence in a given area, since there is some evidence that lower probabilities for species in this taxa often describe a species' potential rather than its occupied niche [112]. We then used these predictions to visualize species richness patterns by selected latitudinal and longitudinal transects. These results were compared with the maps generated using regional distributions and sighting locations.

#### Temporal patterns

The analysis of temporal changes in Mediterranean marine biodiversity requires the integration of diverse data from paleontological, archaeological, historical, and fisheries data, as well as ecological surveys and monitoring data [e.g., [113]–[116]]. We summarized temporal changes of diversity using studies that dealt with this challenge using available data that informed on changes over past centuries and millennia. We integrated historical records of Mediterranean monk seals and sea turtles around the Mediterranean to explore examples of historical spatial changes [22], [23], [101], [106], [117]–[119].

For the north Adriatic Sea, we analyzed data from Lotze et al. [113], who used a multidisciplinary approach to assess the ecological changes and overall shift in diversity over historical time scales in 12 estuaries and coastal seas worldwide, including the north Adriatic Sea. They assessed the number of species that became depleted (>50% decline), rare (>90% decline), or extirpated (locally extinct) in the north Adriatic Sea over past centuries and millennia, based on records for 64 species or species groups that used to be of ecological or economic importance in the Adriatic Sea (File S2). These records included marine mammals, birds, reptiles, fish, invertebrates, and plants and were grouped into ten distinct cultural periods (File S2).

### Threats to biodiversity

Changes in diversity are partially driven by anthropogenic factors, in addition to natural forces. Therefore, our last aim was to identify and quantify the importance of historical and current human-induced drivers and threats to marine biodiversity.

We used the aggregated results presented by Lotze et al. [113] and explicitly separated the data available for the north Adriatic Sea as an example to explore historical threats in the Mediterranean. Those authors evaluated human impacts that caused or contributed to the depletion or extirpation of species in the north Adriatic Sea over historical time scales.

We also identified current human threats to diversity using published data on specific taxa and areas of the Mediterranean (File S2) and the opinion of experts. Each expert was asked to (1) list main threats to diversity for their taxonomic expertise group using data available and experience, and (2) rank those threats from 1 to 5, taking into account the relative importance of each threat to the biodiversity (0: no importance, 1: lowest in importance, 5: highest in importance). The experts repeated the ranking exercise considering data available and projecting their results 10 years into the future (File S2).

In addition and to visualize the impacts of climate warming on species diversity, we documented the mean location of February (the coldest month of the year in the Mediterranean) sea surface isotherms (°C) for the period 1985 to 2006, integrating several data sources. We also generated current and projected future temperature maps, which we compared with sea surface temperature (SST) data from the 1980s. First, we compiled weekly SST data from the National Climatic Data Center (National Operational Model Archive and Distribution System Meteorological Data Server, NOMADS, NOAA Satellite and Information Service), and interpolated maps at 0.1° resolution. Next, we averaged weekly SST values from 1981 to 1984 for each 0.1° grid cell. Last, we used the Mediterranean model OPAMED8 based on the A2 IPCC scenario [120] to visualize the future climate. This model considers main forcing parameters (river runoffs, exchanges with connected seas, and wind regimes) and was used to generate climate data for the middle (2041–2060) and the end of the twenty-first century (2070–2099).

Finally, we visualized potential hot spots for conservation efforts by linking predicted species distributions from the AquaMaps model to status information reported by the International Union for Conservation of Nature [121]–[123]. From the available AquaMaps, a total of 110 maps belonged to vertebrate species that had been classified as critically endangered, endangered, vulnerable, or near threatened in the Mediterranean Sea. This represented the 16% of all species included in the Mediterranean AquaMaps (File S2). We subsequently mapped the richness of these species using a probability threshold of more than 0.4, which usually corresponds to the most frequently used and ecologically most important habitats [112].

## Results

### Diversity estimates in the Mediterranean

Our analysis revealed approximately 17,000 species occurring in the Mediterranean Sea (Table 1 and File S2). Of these, at least 26% were prokaryotic (Bacteria and Archaea) and eukaryotic (Protists) marine microbes. However, the data available for Bacteria, Archaea, and Protists were very limited, so these estimates have to be treated with caution (see next section), as well as data for several invertebrate groups (such as Chelicerata, Myriapoda, and Insecta).

Within the Animalia, the greater proportion of species records were from subphylum Crustacea (13.2%) and phyla Mollusca (12.4%), Annelida (6.6%), Plathyhelminthes (5.9%), Cnidaria (4.5%), the subphylum Vertebrata (4.1%), Porifera (4.0%), Bryozoa (2.3%), the subphylum Tunicata (1.3%), and Echinodermata (0.9%). Other invertebrate groups encompassed 14% of the species, and Plantae included 5%. Detailed biodiversity estimates of main taxonomic groups of benthic macroscopic primary producers and invertebrates are summarized in Table 1 and documented in File S2 in detail.

Available information showed that the highest percentage of endemic species was in Porifera (48%), followed by Mysidacea (36%), Ascidiacea (35%), Cumacea (32%), Echinodermata (24%), Bryozoa (23%), seaweeds and seagrasses (22%), Aves (20%), Polychaeta (19%), Pisces (12%), Cephalopoda (10%), and Decapoda (10%) (File S2). The average of the total endemics was 20.2%. In some groups the percentage of endemics was now lower than in the past, partly due to new finding of Mediterranean species in adjacent Atlantic waters (File S2).

### The biodiversity of the “smallest”

An important bulk of species diversity was attributed to the prokaryotic (Bacteria and Archaea) and eukaryotic (Protists) marine microbes. However, the differences in the methodologies and types of studies and the continuously changing state of our knowledge of marine microbial diversity make it difficult to provide species estimates for the Mediterranean (or from anywhere else) and establish comparisons.

Current methods cannot yet provide reliable estimates of the microbial richness of a system [e.g., [124]] because of (i) our limited capacity to describe morphological variability in these organisms, (ii) the limited development and the biases associated with molecular techniques used to identify them, even with the use of the most powerful of these techniques, and (iii) the uncertainty in determining a “microbial species” and where to draw the line that differentiates one species from another. Morphological variability is used to describe diversity of some groups of microbes, such as ciliates and microphytoplankton [125], but this is not useful for most nano- and almost all picoplanktonic organisms, including all Archaea and most Bacteria. Therefore, until recently, surveys of microbial diversity were mainly limited to those taxa with enough features to be described under an optical microscope. Among phytoplankton, the best-studied groups included thecate dinoflagellates, diatoms, coccolithophores, and silicoflagellates. Among microzooplankton, groups like tintinnids, foraminifers, or radiolarians attracted most attention. Much less information is available on “naked” auto- or heterotrophic flagellates and on small picoplankton species.

However, researchers have made efforts to obtain estimates of the dominant microbial species in Mediterranean waters. The expansion of electron microscopy in the last decades of the twentieth century helped to untangle inconsistencies in the distribution of some described species and to consolidate the establishment of a biogeography of many protist taxa. More recently, molecular techniques (metagenomics) have been used to enumerate the microorganisms present in a given sample and have completely transformed the field by changing ideas and concepts. These advances have highlighted the problems with the species concept when applied to microbial communities, which may be based on morphology, biology, or phylogeny [125]. Furthermore, different methodologies have biases that give different views of microbial diversity [e.g., [126],[127]], and now we know that microdiversity is a general characteristic of microbial communities [128], making the delimitation of “diversity” units difficult. To avoid some of the problems with the “species” delimitation, some authors prefer to use “functional diversity”: the amount and types of microbial proteins (e.g., functions) in the sample [e.g., [129]], rather than “species” diversity.

According to the compilation published in Hofrichter [87], the number of described protist species in the Mediterranean is approximately 4,400 (Table 1). However, this estimate requires cautious interpretation and it is likely that many morphospecies, more or less well described, will include a number of cryptic or pseudocryptic variants [e.g., [125]]. Molecular methods have recently uncovered new sequences that are being associated with the organisms they represent [130]. Fingerprinting techniques [131] have been used to compare microbial communities and establish the scale of variability of these communities. For example, Schauer et al. [132] determined that, along the coastal northwestern Mediterranean, the time of the year was more important than exact location in determining bacterial community structure. Acinas et al. [133] and Ghiglione et al. [134] showed that microbial communities tend to be similar in the horizontal scale and much more variable on the vertical scale, but these techniques are not appropriate to determine the number of species present and usually refer only to the dominant organisms. Recent application of new methodologies (such as metagenomics and 454-tag sequencing) will in the near future provide more accurate estimates.

All studies to date concur in identifying members of the SAR11 group as some of the most abundant Mediterranean bacteria, comprising 25–45% of the reported sequences [e.g., [126],[127]]. These are followed by other Alphaproteobacteria, which tend to be more common in coastal regions and during algal blooms (such as *Roseobacter*-like). Cyanobacteria (*Prochlorococcus* and *Synechococcus*), diverse culturable (Alteromonadales) and unculturable Gammaproteobacteria and Bacteroidetes form the rest of the diversity with some differences with depth and with distance from land. Several studies have concentrated in the diversity of subgroups of these abundant bacteria in the Mediterranean [e.g., [135],[136]].

Additionally, the diversity of deep samples and the communities from which they are taken have received considerable attention in the Mediterranean. Specific and likely unique ecotypes of some bacteria appear at certain depths, [e.g., [137]], free-living communities appear to be as complex as epipelagic communities [138], and appear to vary seasonally, as do surface communities [139]. The deep-sea Mediterranean maintains several extremely peculiar and interesting ecosystems, such as the deep hypersaline anoxic “lakes” in the Ionian Sea that are reported to include several new and little-known microbial lineages [e.g., [140]].

Some studies have shown that bacterial richness peaks in tropical latitudes [e.g., [141]] and concluded that at Mediterranean latitudes the number of detectable “operational taxonomic units” (OTUs) is between 100 and 150. Zaballos et al. [142] arrived at a similar value that, once extrapolated, indicated a value of approximately 360 OTUs for surface waters. A slightly lower value was estimated for the coastal Blanes Bay Microbial Observatory [e.g., [126]] based on a different approach. Archaeal richness is known to be lower than bacterial richness [e.g., [143]], and this has been seen in the Mediterranean and in other oceans. Results of these new sequencing techniques suggest that microbial richness in the sea is much higher because of the presence of a “rare biosphere” composed of very few individuals of many distinct organism types [144], [145]. Application of this technique to data from the northwestern Mediterranean indicates that the numbers should be raised to about 1,000 “bacterial species” per sample [146]. Again, the real magnitude of bacterial richness in the Mediterranean cannot be appreciated with the techniques available.

A similar situation to that with prokaryotes occurs with small eukaryotes, which are photosynthetic, heterotrophic, or mixotrophic organisms. These small eukaryotes are found in abundances of 10^3^–10^4^ ml^−1^ and have low morphological variability [147]. Thus we must rely on molecular techniques to grasp their diversity. Molecular work has allowed the discovery of new groups of eukaryotes present in this smallest size class [148], [149].

The study of Mediterranean protists has benefited from the early establishment of marine laboratories and a number of illustrated books and checklists [e.g., [150]–[155]]. More recent inventories can be found in Velasquez and Cruzado [156] and Velasquez [157] for diatoms, Gómez [158] for dinoflagellates and Cros [159] for coccolithophorids. The compilation of northwestern Mediterranean diatom taxa of Velasquez [157] records 736 species and 96 genera. The checklist of Gómez [158] contains 673 dinoflagellate species in 104 genera.

Cros [159] lists 166 species of coccolithophorids of the northwestern Mediterranean and revised the classification of several important taxa [see also 160]. Recently, the discovery of a number of combination coccospheres bearing holo- and heterococcoliths [161] fostered the recognition that holococcolithophores do not belong to a separate family, as previously accepted, but are part of a life cycle that includes holo- and heterococcolithophore stages. The biodiversity of photosynthetic nano- and picoflagellates other than coccolithophores is poorly known for most groups, as may be expected from the difficulties involved in their identification. However, in the last decade, work using optical and electron microscopy, often in combination with molecular and culturing techniques, has considerably increased the taxonomic knowledge of many of these groups and has highlighted the potential existence of much cryptic or unknown diversity [e.g., [162],[163]].

There are few taxonomic surveys of heterotrophic flagellates [e.g., [164]], although many phytoplankton studies based on microscopy also included taxa from these groups. Massana et al. [165] describes a high diversity of picoeukaryotic sequences, belonging to two groups of novel alveolates (I with 36% and II with 5% of clones), dinoflagellates (17%), novel stramenopiles (10%), prasinophytes (5%), and cryptophytes (4%). Later work has shown that these novel stramenopiles are free-living bacterivorous heterotrophic flagellates [130].

Most of the biodiversity work on ciliates has focused on tintinnids or loricate ciliates, while studies involving naked ciliates tend to use groupings based on ecological morphotypes and only rarely include detailed taxonomical work [e.g., [155],[166]–[168]]. Numbers of species ranging from 40 to 68 were recorded in one to several-year surveys of various Mediterranean sites [among others 154]. Other groups, such as the Foraminifera, which have calcium carbonate tests, and the Radiolaria, which produce siliceous or strontium sulfate skeletons, have been the subject of many stratigraphical and paleoceanographical studies. However, biodiversity work on living Foraminifera and Radiolaria in the Mediterranean is scarce [e.g., [155],[169],[170]]. Hofrichter [87] provided a systematic summary of the main groups and species of both autotrophic and heterotrophic protists found in the Mediterranean.

### The biodiversity at high trophic levels

Species that occupy the upper trophic levels, normally beyond the level of secondary consumers, are classified as predators. They have lower diversity than other taxonomic groups, but information available is usually more detailed (Table 1 and File S2). We reviewed data available for fish, seabirds, marine mammals, and turtles in the Mediterranean Sea.

Ground-breeding species such as seabirds (gulls and terns) are counted using census bands [171] and monitored by satellite tracking. However, procellariiforms reproduce in caves and burrows in cliffs on remote, inaccessible islets, and census methods to estimate population densities are not totally reliable. Population models, based on demographic parameters, allow researchers to estimate extinction probabilities [172]. A census of marine mammals or turtles normally uses transect data collected from aerial or boat-based sighting surveys developed to assess abundance, while movement patterns are tracked with transmitters and monitored by satellite tracking as well. Fish species are mainly studied using scuba diving or fishing techniques.

There is still some discussion about diversity estimates for these taxonomic groups. For fish species, for example, several estimates of Mediterranean diversity exist: Quignard [173] lists a total of 562 fish species occurring in the Mediterranean Sea; Whitehead et al. [70] mention 589; Fredj and Maurin [68] list a total of 612 species (and identified 30 species as uncertain); and Quignard and Tomasini [90] register 664 species. Hofrichter [87] summarizes 648 species, and Golani et al. [89] report a total of 650 fishes (File S2). Fish diversity estimates also change as new species are described or reclassified. The updated list of exotic fish species [88] reveals that the Mediterranean currently contains 116 exotic species, although more species are likely to be cited. There is also a long-standing controversy regarding genetic differentiation among a few fish populations and sub-basins, especially of commercial species due to management implications (for example for the European anchovy *Engraulis encrasicolus*), although results are still under debate [e.g., [174]].

Approximately 80 fish species are elasmobranchs, although the status of some is uncertain because of infrequency or uncertain reporting [e.g., [123],[175],[176]]. According to Cavanagh and Gibson [123], nine of these elasmobranch species may not breed in the Mediterranean, while some are rare because the Mediterranean represents the edge of their distribution ranges. Only four batoid species are Mediterranean endemics: the Maltese skate (*Leucoraja melitensis*), the speckled skate (*Raja polystigma*), the rough ray (*R. radula*), and the giant devilray (*Mobula mobular*) [175].

Nine species of marine mammals are encountered regularly in the Mediterranean (File S2) [92], [93], [94], [97]. Of these species, five belong to the Delphinidae, and one each to the Ziphiidae, Physeteridae, Balaenopteridae, and Phocidae. Other 14 species are sporadically sighted throughout the basin and are considered “visitors” or “non-residents.”

Of the seven living species of sea turtles, two (the green and the loggerhead *Chelonia mydas* and *Caretta caretta* - Cheloniidae) commonly occur and nest in the Mediterranean, and one (leatherback turtle *Dermochelys coriacea* - Dermochelyidae) is regularly sighted but there is no evidence of nesting sites. The other two (hawksbill and Kemp's riddle turtles *Eretmochelys imbricata* and *Lepidochelys kempi* - Cheloniidae) are extremely rare and considered to be vagrants in the Mediterranean (File S2) [22], [95], [96], [98], [99].

Seabirds from the Mediterranean have a low diversity (15 species, File S2) and their population densities are small, consistent with a relatively low-productivity ecosystem compared with open oceans, and particularly with upwelling regions. Ten of the Mediterranean species are gulls and terns (Charadriiformes), four are shearwaters and storm petrels (Procellariiformes), and one is a shag (Pelecaniformes). Three of the ten species are endemics [108]–[110].

### What is hidden in the deep?

Because of the large size of the Mediterranean deep-sea ecosystems (Figure 1d), our knowledge of the benthic deep-sea diversity is incomplete [177]. In the past 20 years, several studies on deep-sea sediment diversity have been undertaken in various oceans [e.g., [178],[179]] but have been limited to a few taxonomic groups. However, due to technological improvements that render the deep waters more accessible, the deep-sea benthos of the Mediterranean has received increased attention and there is progress toward a more comprehensive view of the levels, patterns, and drivers of deep-sea biodiversity in this semienclosed basin [180].

Its paleoecological, topographic, and environmental characteristics suggest that the Mediterranean Sea is a suitable model for investigating deep-sea biodiversity patterns along longitudinal, bathymetric and energetic gradients across its different regions. There are few areas with depths greater than 3,000 m (Figure 1d), and typically bathyal or abyssal taxonomic groups are limited. Cold-water stenothermal species that elsewhere represent the major part of the deep-sea fauna [181] are also unknown in the Mediterranean Sea. The Mediterranean abyssal macrobenthos comprises a large number of eurybathic species and only 20–30 true abyssal species. In the western basin, where the depth does not exceed 3,000 m, the abyssal fauna is less abundant than in the deeper eastern basin, where abyssal species are dominant in the Matapan trench, which is more than 5,050 m deep [182]. The close affinity between Mediterranean and Atlantic congeneric deep-water species suggests that the ancestors of the Mediterranean bathyal endemic species moved from the Atlantic when conditions were favorable (i.e. when larvae of deep Atlantic fauna was able to enter in the Western Mediterranean due to hydrodynamic and physico-chemical conditions allowed it).

According to Pérès [183], the deep-water fauna of the Mediterranean has a lower degree of endemism than that of the Atlantic at similar depths. So while the Mediterranean basin is recognized as one of the most diverse regions on the planet, the deep sea in the Mediterranean may contain a much lower diversity than deep-sea regions of the Atlantic and Pacific oceans [184], [185]. The reasons for such a low diversity may be related to (a) the complex paleoecological history characterized by the Messinian salinity crisis and the almost complete desiccation of the basin [186], and (b) the Gibraltar sill that is, potentially, a physical barrier to the colonization of larvae and deep-sea benthic organisms from the richer Atlantic fauna. These factors may explain the composition of the benthos in the deep sea of the Mediterranean [187]. It may also be that the high deep-sea temperatures (about 10°C higher than in the Atlantic Ocean at the same depth) have led to a Mediterranean deep-sea fauna that consists of reproductively sterile pseudopopulations that are constantly derived through larval inflow. These postulates were based on the analysis of the macrobenthos, characterized by life cycles with meroplanktonic larvae that are spread by currents [188].

However, the populations of the most common benthic mollusks in depths greater than 1,000 m off the Israeli coast are composed of both adult and juvenile specimens, and one species, *Yoldia micrometrica*, the most common and abundant species in the eastern Mediterranean, is unrecorded from the westernmost part of the sea. In addition, and though much reduced in diversity and richness compared with the deep-sea fauna of the western and central basins of the Mediterranean, the Levantine bathybenthos is composed of autochthonous, self-sustaining populations of opportunistic, eurybathic species that have settled there following the last sapropelic event [189]–[191].

Macpherson [192] and Briggs [193] have suggested that within the Atlantic-Mediterranean region, the fauna (including invertebrates and fishes) of the Mediterranean is more diverse than that of the Atlantic and displays considerable endemism. For strictly deep-dwelling species (e.g., the deep-water decapod crustacean family Polychelidae), the Gibraltar sill is not an impenetrable barrier for some deep-waters macrobenthic species [194]. Moreover, available hypotheses did not consider meiofauna diversity, which is characterized by direct development [188] but also by a small size, which allows organisms' resuspension and drifting over wide regions. This is consistent with information on the most abundant deep-sea phylum, the Nematoda, which often accounts for more than 90% of total meiofauna abundance [9], [195]. Nematode diversity has been investigated only in a few areas of the deep sea in the Mediterranean: slopes of the Gulf of Lions, Catalan margin and Corsica, Tyrrhenian basin, and Eastern Mediterranean [e.g., [196]–[198]]. Recent collections from a limited number of sites throughout the Mediterranean basin (at approximately 1,000 m, 3,000 m, and 4,000 m depth), suggest that, conversely to what was expected, the deep-sea nematode fauna of the Mediterranean basin is rather diverse.

At bathyal and abyssal depths, levels of nematode genera and species richness are similar to those reported from other deep-sea areas of the world oceans [198]. In the deep sea of the Mediterranean, small-bodied taxa (e.g., meiofauna) can reach a high diversity, and with the presence of a high prokaryotic diversity in the sediments of the deep-sea Mediterranean [199], this may change the view that the Mediterranean deep-sea biota is impoverished in comparison with its Atlantic counterpart. Endemic macrobenthic species account for approximately 13–15% of total species number at depths from 200 m to 1,000 m, and approximately 20% at 2,000 m [200]. These estimates are similar for each taxon (Table 1) and are further supported by the continuous discovery of new species (both within the highly diverse Nematoda and in rare phyla such as the Loricifera) in different sectors of the deep Mediterranean [180]. Therefore, the general conclusion that the biodiversity is high in coastal systems and low in the deep sea of the Mediterranean might not hold true. Detailed references about the deep Mediterranean can be found in [180].

### New biodiversity

The biodiversity of the Mediterranean is definitively influenced by the introduction of new species [e.g., [88],[201]–[208]]. Since the first review of exotic species in the Mediterranean [209], the studies in this topic have intensified. Now more than 600 metazoan species have been recorded as alien, these representing 3.3% of the total estimates (Table 1, and File S2 for detailed information by taxonomic group). However, this estimate is continuously increasing and may be as high as 1,000 species if unicellular aliens and foraminiferans are included [e.g., [206],[207],[208]].

Most of these introductions are littoral and sublittoral benthic or demersal species (or their symbionts). Because the shallow coastal zone, and especially the benthos, has been extensively studied and is more accessible than deeper waters, new arrivals probably will be encountered and identified in shallow waters. The species most likely to be introduced by the predominant pathways (the Suez Canal, vessels, and mariculture) are shallow-water species.

A taxonomic classification of the alien species showed that the alien phyla most frequently recorded are Mollusca (33%), Arthropoda (18%), Chordata (17%), Rhodophyta (11%), and Annelida (8%). The data are presumably most accurate for large and conspicuous species that are easily distinguished from the native biota and for species that occur along a frequently sampled or fished coast and for which taxonomic expertise is readily available. Data are entirely absent for many of the small members of invertebrate phyla [210]. Thus, the true numbers of alien species are certainly downward biased.

**Figure 2:**
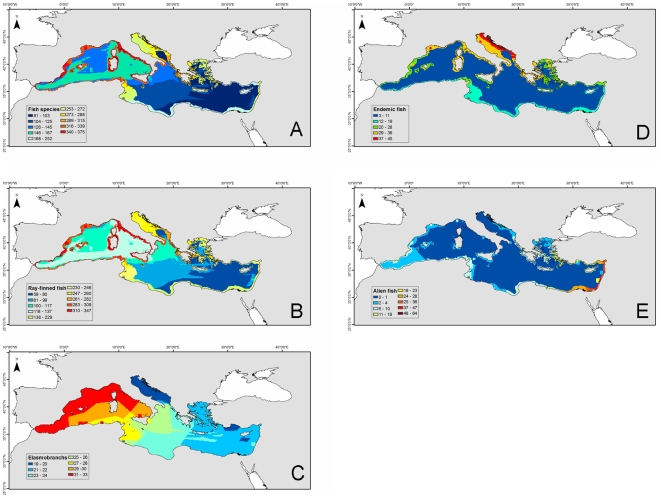
Spatial patterns of fish species richness in the Mediterranean Sea based on superimposed expert-drawn maps. (A) All fish species (n = 625), (B) ray-finned fish species (n = 545), (C) elasmobranchs (n = 80), (D) endemic fish species (n = 79), (E) alien fish species (n = 127) [data modified from 91]. Colors express species occurrence from blue (little or no occurrence) to red (highest occurrence). The size of the cell is 0.1×0.1 degree.

The native range of the alien species in the Mediterranean was most commonly the Indo-Pacific Ocean (41%), followed by the Indian Ocean (16%), and the Red Sea (12%), while some species have a pantropical or circumtropical distribution (19%). The actual origins of the Mediterranean populations of a species widely distributed in the Indo-Pacific Ocean may be their populations in the Red Sea, both from the Indian or Pacific oceans, or a secondary introduction from already established populations in the Mediterranean itself [e.g., [50]]. However, and with few notable exceptions [e.g., [211],[212]], the source populations of alien species in the Mediterranean have not been assessed by molecular means. Even so, it is clear that most alien species in the Mediterranean are thermophilic and therefore originated in tropical seas (see Figure 2). The exceptions are exotic algae, of which the largest numbers are in the Gulf of Lions and the northern Adriatic [213], [214], and a few other examples [e.g., [215]].

As far as can be deduced, the majority of aliens in the Mediterranean entered through the Suez Canal (Erythrean aliens) (53%), and an additional 11% were introduced primarily through the Canal and then dispersed by vessels. Introductions from vessels from other parts of the world account for 22% of introduced species, and aquaculture accounts for 10%. A further 2% arrived with the introduction of aquaculture and were secondarily spread by vessels. The means of introduction differ greatly among the phyla: whereas of the alien macrophytes, 41% and 25% were introduced through mariculture and vessels, respectively, the majority of alien crustaceans, mollusks, and fish are Erythrean aliens (59%, 64%, and 86%, respectively), and mariculture introductions are few (4%, 5%, and 4%, respectively) [[216], B.S. Galil, personal observation].

The numbers of alien species that have been recorded over the past century have increased in recent decades. The increasing role of the Mediterranean as a hub of international commercial shipping, a surge in the development of marine shellfish farming over the last 25 years, and the continued enlargement of the Suez Canal have contributed to the resurgence of introductions since the 1950s. Many introduced species have established permanent populations and extended their range: 214 alien species have been recorded from three or more peri-Mediterranean countries, and 132 have been recorded from four or more countries [[216], B.S. Galil, personal observation].

A comparison of the alien species recorded along the Mediterranean coasts of Spain and France and an equivalent length of coast in the Levantine Sea (from Port Said, Egypt, to Marmaris, Turkey) showed marked differences in their numbers, origin, and means of introduction. There are nearly four times as many alien species along the Levantine coast (456 species) as along the western coast of the Mediterranean (111 species). The majority of aliens in the Eastern Mediterranean entered through the Suez Canal (68% of the total, 14% vessel-transported, 2% mariculture), whereas mariculture (42%), vessels (38%), or both (5%) are the main means of introduction in the Western Mediterranean [[216], B.S. Galil, in preparation]. Climate change favors the introduction of Red Sea species in the southeastern Mediterranean and their rapid spreading northwards and westwards (see section 4.2c and d). It similarly favors species coming from the African Atlantic coasts to enter the western basin [89], [217].

### Spatial patterns of Mediterranean biodiversity

#### Longitudinal and latitudinal patterns

Describing the distribution of marine diversity is as important as quantifying it. In the Mediterranean, a northwestern-to-southeastern gradient of species richness was observed in most groups of invertebrate species analyzed here, with a highly heterogeneous distribution of species in the different regions (Table 2, and File S2 for detailed information). We noticed only a few exceptions. For example, while there was the same number of *Euphausia* species in the western and central basins, estimates for several other invertebrate groups were higher in the Aegean Sea than in central areas of the Mediterranean. These exceptions may be due to different species tolerance to environmental factors (such as temperature and salinity), connectivity between regions, and to the lack of data in some regions.

**Table 2:** Species richness by taxa and regions of the Mediterranean Sea.

	W Med^1^	E Med^2^	NW Med	Alboran Sea	SW Med	Adriatic Sea	Central Med	Ionian Sea	Aegean Sea	Tunisian Pl.^3^/Gulf of Sidra	Levantine Basin^10^	Reference 11
Ceramiales (Rhodophyta)	248					198	211		193			
Phaeophyceae			161		119(4)	160	183(5)	122(6)			74	[16]
Porifera			432	181	123	230		181	200	90	94	
Anthozoa	151					100		58	90		38	
Gastropoda	1148					462	582		622		83	[66]
Cephalopoda	61	55				45						[435]
Polychaeta	946	877										
Harpacticoid copepoda	254								96			
Cumacea	85	74	78	43	42	13	50(5)	28	43	4	48	
Mysidacea	90	55	62	9	2	34	64(5)	7	5	30		
Euphausiacea	13					12	13		12		11	[67]
Isopoda	149					47	26		74		34	[66]
Cirripedia	34					17	17		17		13	[66]
Amphipoda	421					242	160		260		144	[66]
Decapoda (1)	316					228	205		252		59	[66]
Decapoda (2)						293(7)			260		230	
Echinodermata	144(8)					101	98(9)		107		73	
Sipuncula			45	19	15	36	36			16		
Ascidiacea	193	167										

N: North, S: South, W: West, E: East, Med: Mediterranean.

(1) Including NW Med, Alboran Sea, SW Med, Tyrrhenian Sea, and excluding Adriatic Sea;

(2) Including Aegean, Ionian, Levantine, and Central Mediterranean;

(3) Plateau;

(4) North Africa,

(5) Tyrrhenian Sea;

(6) Mediterranean Greece and Turkey,

(7) Italian waters;

(8) Including Thyrrenian Sea, Alboran, and SW Mediterranean;

(9) Including the Ionian Sea,

(10) There are severe gaps in our knowledge of most invertebrate taxa in the Levantine Sea,

(11) This contribution (details in supplementary material), except where noted.

We found similar results for vertebrate species. There was a decreasing gradient from northwest to the southeast, while the sea around Sicily had the highest richness (375 species per 0.1×0.1 degree cell), followed by other northwestern coastal and shelf areas (Figures 2a–b). The distribution of elasmobranch species was not homogenous either, showing a higher concentration of species in the west (Figure 2c). The endemic richness gradient of fish species was more pronounced with latitude, the north side exhibiting a greater richness, and the Adriatic appearing as a hot spot of endemism with 45 species per cell (Figure 2d). Spatial patterns also showed how most of Mediterranean coastal waters have been colonized by exotic species (Figure 2e). The highest richness of exotic species occurred along the Israeli coast.

**Figure 3:**
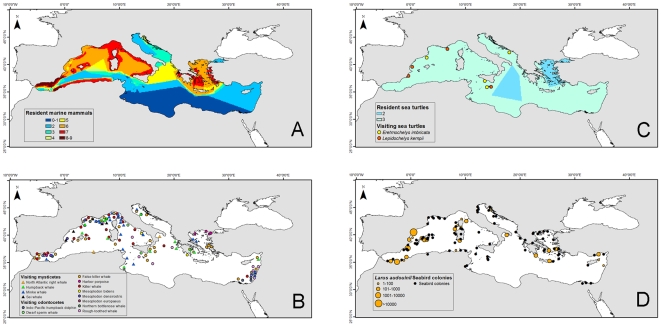
Spatial patterns of vertebrate species richness in the Mediterranean Sea based on superimposed expert-drawn maps (excluding fish species). (A) resident marine mammals (n = 9), (B) nonresident marine mammals (n = 14), and (C) resident sea turtles (n = 3), as well as sighting records (dots) of the two visiting sea turtles. Colors express species occurrence from blue (little or no occurrence) to red (highest occurrence). (D) Seabird colonies (the yellow dots show the distribution and population density of colonies in breeding pairs (bp) of Audouin's gull: Some dots represent the epicenter of several smaller colonies in archipelagos). The size of the cell is 0.1×0.1 degree.

Marine mammals were concentrated in the Western Mediterranean and Aegean seas (Figure 3a). Of the nine resident marine mammals, eight were found in the western part of the basin. This distribution pattern was also observed for the visiting marine mammals (Figure 3b). Two of the three resident sea turtles (loggerhead, green, and leatherback turtles) occurred in the central Mediterranean and Aegean seas, while the two visiting turtles were absent from the eastern side (Figure 3c). There were fewer seabird colonies and seabird density was lower in the southeast than the northwest (Figure 3d).

**Figure 4:**
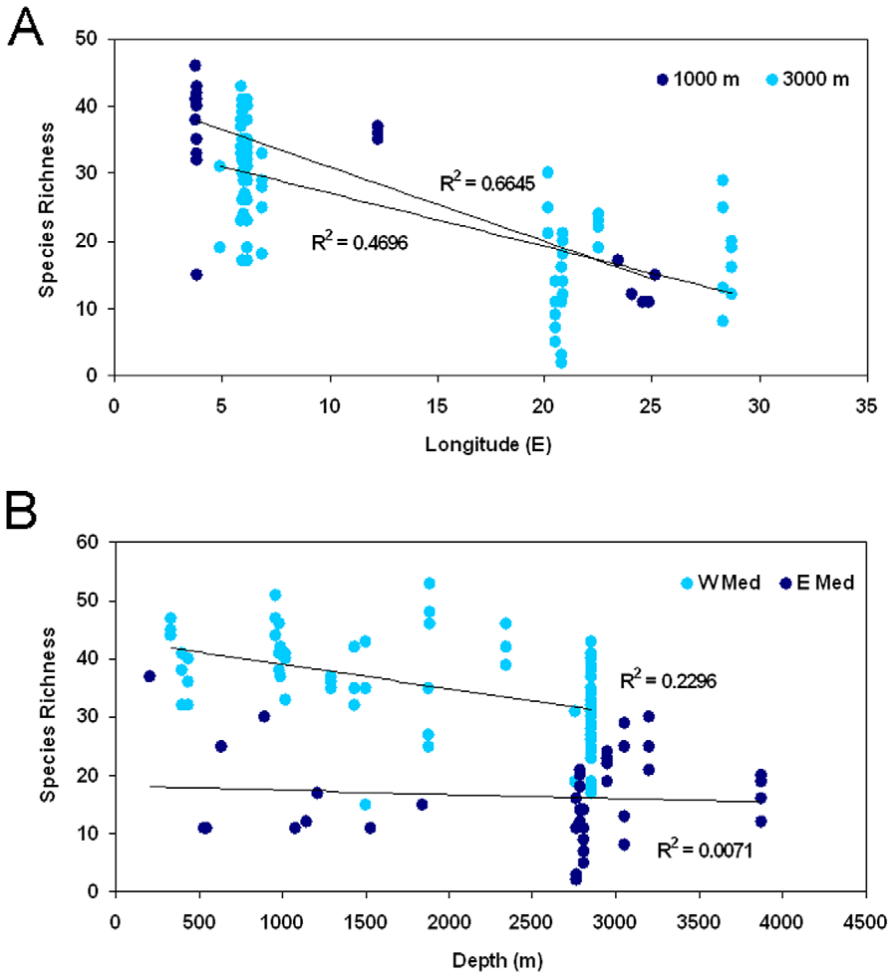
Patterns of benthic biodiversity in the deep sea of the Mediterranean. (A) Longitudinal patterns, and (B) bathymetric patterns of benthic nematodes along the open slopes of the European margins. Benthic biodiversity is estimated as the total number of meiofaunal taxa, and as nematode species richness (expected number of nematode species for a theoretical sample of 51 specimens).

Spatial patterns of benthic biodiversity in the deep sea are poorly known in comparison with other ecosystems. Available information is scarce and our maps and estimates include only approximations for the deep sea. In this context, metazoan meiofauna and, in particular, nematodes can be used to describe the biodiversity patterns in the deep sea. Deep-sea nematode diversity appears to be related to that of other benthic components such as foraminifers [218], macrofauna [219], and the richness of higher meiofauna taxa in the deep sea [220]. Results for the deep sea of the Mediterranean show a clear longitudinal biodiversity gradient that also occurs along the open slopes, where values decrease eastward, from Catalonia to the margins of southern Crete (Figure 4a). The analysis of the Nematoda indicates that at equally deep sites, nematode diversity decreases from the western to the eastern basin and longitudinal gradients are evident when comparing sites at 3,000 m or 1,000 m depth [195]. Complementary information on spatial patterns of the deep Mediterranean fauna can be found in [180].

Additional information from the literature on spatial patterns of Mediterranean marine diversity suggests that the measurement of local *α*-diversity is not sufficient to draw a clear picture for the whole Mediterranean basin. Whittaker [221] defined *α*-diversity as the number of species found in a sample (or within a habitat), *β*-diversity as the extent of species replacement along environmental gradients (termed “turnover diversity” by Gray [222]), and *γ-*diversity as the diversity of the whole region. The analysis of *β-*diversity of Nematoda among different sites in the deep sea of the Mediterranean and across bathymetric and longitudinal gradients reveals an extremely high species turnover. By comparing nematode assemblages at (a) different depths, (b) similar depths in two different basins, and (c) similar depths within the same basin, the dissimilarity of biodiversity among deep-sea samples is always greater than 70% [195], [197], [198], [223]. On average, the dissimilarity of nematode diversity between western and eastern Mediterranean at about 3,000 m depth is greater than 80% and at similar depths the dissimilarity between Atlantic and Western Mediterranean exceeds 90%. These findings indicate that each region is characterized by the presence of a specific assemblage and species composition. This has important implications for estimating the overall regional diversity (*γ*-diversity) but also suggests the presence of high biogeographic complexity in the Mediterranean. However, these patterns may not hold for all the taxonomic groups [224], and a broader comparison is needed.

#### Spatial patterns predicted with AquaMaps

**Figure 5:**
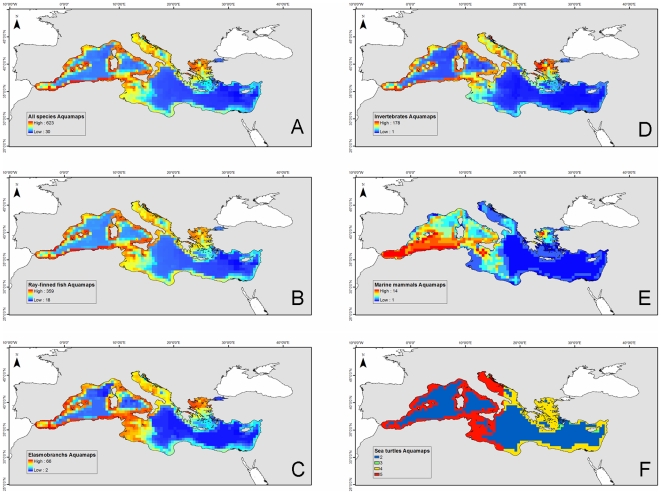
Spatial predicted patterns of species richness in the Mediterranean Sea based on the AquaMaps model [80, and File S2]. (A) All species (n = 693), (B) ray-finned fishes (n = 397), (C) elasmobranchs (n = 74), (D) invertebrates (n = 193), (E) marine mammals (n = 16), (F) sea turtles (n = 5). All maps were generated without imposing a probability threshold except for marine mammals, for which we used a probability threshold of ≥0.4. Colors express species occurrence from blue (little or no occurrence) to red (highest occurrence). The size of the cell is 0.5×0.5 degree.

Predicted patterns of overall species richness based on AquaMaps showed a concentration of species in coastal and continental waters most pronounced in the Western Mediterranean, Adriatic, and Aegean seas (Figure 5). Less than half of the species were predicted to occur in the deeper waters of the central Mediterranean, and biodiversity was particularly low in offshore waters at the eastern end. Given the overall proportion of ray-finned fishes in AquaMaps dataset (File S2), overall biodiversity patterns from these figures were largely dominated by Actinopterygii (Figures 5a and b). The concentration in coastal waters was more pronounced in the map focusing on these taxa (Figure 5b). Predicted species richness of elasmobranchs was similar to that for Actinopterygii, but rays and sharks occurred farther offshore, especially in the waters of Tunisia and Libya (Figure 5c). The Aegean Sea, especially its northern sector, also showed high invertebrate species richness, which was otherwise low in most of the remaining central and eastern basin (Figure 5d). Biodiversity patterns for the marine mammals contrasted with patterns for fishes and invertebrates in that many species were also predicted to occur in the offshore western and central basin waters, and particularly in slope waters (Figure 5e). The biodiversity patterns of sea turtles broadly mimic those of the other more species-rich taxa in that there was a concentration in coastal areas and a decline in species richness from the northwest to the southeast (Figure 5f).

**Figure 6:**
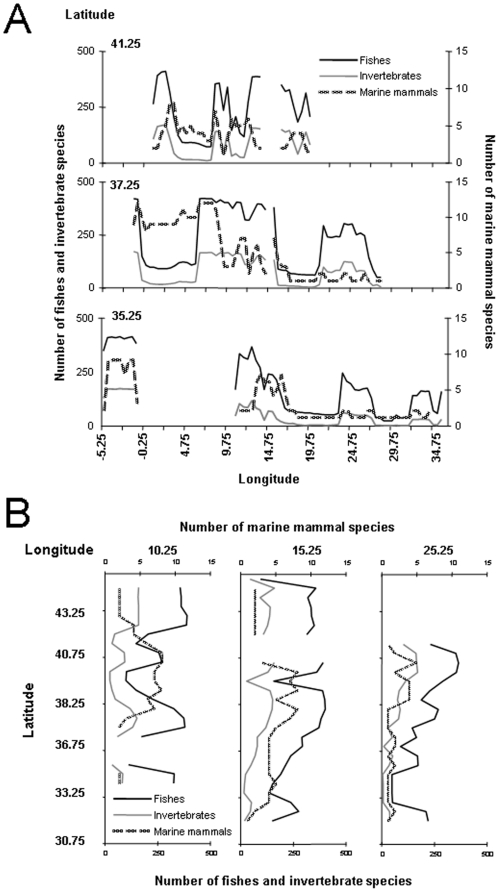
Transects of spatial predicted species richness produced using the AquaMaps model [80, and File S2]. (A) Latitudinal transects, and (B) Longitudinal transects. The contribution of fishes, invertebrates, and marine mammals to geographic gradients in biodiversity is shown.

Therefore, there were similarities and differences between expert-drawn maps (Figures 2 and 4) and modeling results (Figure 5). The pattern describing species richness of ray-finned fish was similar overall (Figures 2b and 5b), but for the elasmobranchs there were some noticeable differences (Figures 2c and 5c). While both methods identified areas around Sicily, the coast of Tunisia, and the Western Mediterranean as high diversity hot spots, the Adriatic and Aegean seas showed up as high in species richness only in the predicted maps. Both types of analyses arrived at similar patterns for marine mammals, although the lack of distinction between resident and visitor species in the AquaMaps analysis hampered the direct comparison of diversity patterns for these taxa. Nevertheless, differences could be seen around the Aegean and Alboran seas (Figures 3c and 6e). Maps of sea turtle diversity showed peaks in the western region based on both types of analysis, but there were a few discrepancies regarding the eastern Mediterranean (Figures 3e and 6f). AquaMaps analysis of predicted species richness of invertebrates also showed a geographical gradient (Figure 5d).

Latitudinal transects corresponding to cross sections through the species richness map (Figure 5a) highlighted the importance of coastal habitats for fishes and invertebrates. These habitats were represented by peaks in species numbers in areas corresponding to shelf waters (Figure 6a). Cross-section gradients followed a similar pattern for fishes and invertebrates; large variations were mostly determined by depth changes along the respective transects. There was also an overarching trend of decreasing species richness from western to eastern waters, a trend that became particularly pronounced in the southern transects. Marine mammal transects diverged from the general trend in that species richness was less directly linked to depth variation. Changes in fish and invertebrate species richness along three different longitudinal cross sections again followed similar depth contours (Figure 6b). Marine mammal longitudinal biodiversity patterns in the Western Mediterranean followed a different trend with highest numbers predicted to occur in deeper waters, such as the southern Tyrrhenian Sea. There appeared to be a general decrease of diversity from northern to southern regions.

#### Bathymetric patterns

Because seaweeds and seagrasses are photosynthetic organisms, their development is limited to shallow areas where there is enough light for growth. They are distributed between the mediolittoral zone and the deepest limit of the circalittoral zone, situated at 110 m in the clearest waters of the western Mediterranean [225] and a bit deeper in the even more oligotrophic waters of the eastern part [27]. Their growth occurs only on the continental shelves and the uppermost parts of seamounts above 150 m depth. Seaweeds, which have a limited distribution across the whole bathymetric gradient, show an increase in species richness from the highest levels of the mediolittoral rocks down to the lower infralittoral and upper circalittoral communities. There they display the highest species richness, as many as 150 species reported in a surface of 1,600 cm^2^ at 18 m depth [226]. Species richness then decreases along the circalittoral zone from the shallowest down to the deepest parts [227], becoming nil at the beginning of the bathyal zone.

**Figure 7:**
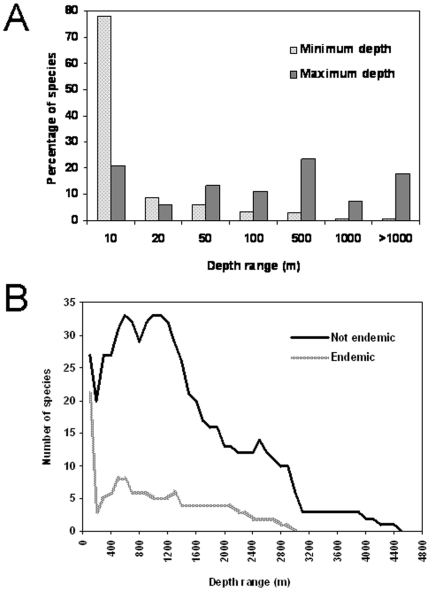
Bathymetric patterns of species richness. (A) Bathymetric ranges of distribution for Mediterranean polychaete species at minimum and maximum depths where they have been reported (File S2), and (B) number of Mediterranean cumaceans recorded in each 100 m depth interval (Endemic species are plotted in gray. For nonendemic species only records from the Mediterranean Sea are considered, File S2).

**Figure 8:**
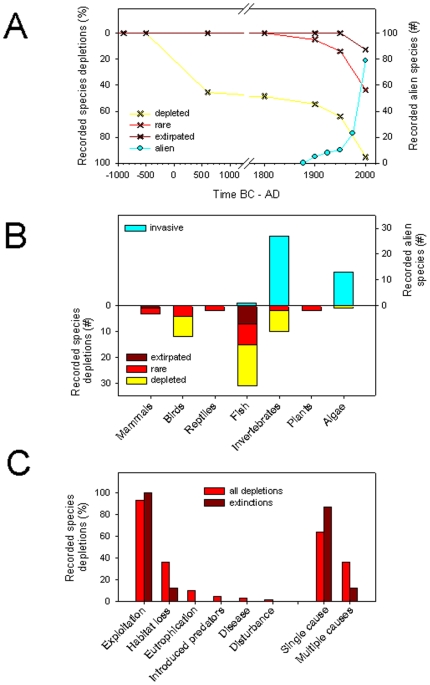
Historical changes and threats of species in the Mediterranean Sea. (A) Historical trends in the proportion of species being depleted (>50% decline), rare (>90% decline), or extirpated (100% decline) in the North Adriatic Sea, based on data for 64 economically and ecologically important species for which long-term records are available. Temporal trends for alien species refer to recorded exotic mollusks in the whole Mediterranean Sea [272]. (B) Shifts in species diversity of the North Adriatic Sea over historical time scales. Species depletions and extirpations occurred mostly in larger species groups, while invasions occurred in smaller and lower trophic-level species [data from 271]. (C) Threats to diversity in the North Adriatic Sea over historical time scales. Shown is the percent of recorded species depletions and extinctions caused by, or attributed to, different human impacts. Also shown is whether human impacts acted as single or multiple causes. Data were adapted from Lotze et al. [113].

The pattern of a generally decreasing diversity with increasing depth was also documented here for invertebrate and fish species (Figures 3, 4, 7, and 8) and is consistent with previous studies [e.g., [31],[228]]. Diversity was concentrated in coastal areas and continental shelves, mainly above 200 m depth. However, patterns did not necessarily show a monotonic decrease with depth. For example, more polychaete species inhabited shallow waters than deep waters, particularly below 1,000 m deep, but this pattern was less clear when looking at maximum ranges of depth (Figure 7a, File S2). It is not clear whether this is a real pattern of lower deep-sea diversity or a result of the lack of proper faunistic studies in the Mediterranean at those depths. Larger numbers of cumacean species were found in shallow waters of 0–99 m depth (48 species) and between 200 m and 1,400 m depth, but species richness decreased below this depth (Figure 7b, references in File S2). The highest endemism (43.8%) was found between 0 and 99 m depth. The largest number of mysidaceans (54 species) was also found in shallow waters less than100 m deep. At depths between 100 m and 1,000 m, 27 species were found, and below 1,000 m, 21 species. The level of endemism was also higher in the 0–100 m depth interval (29 species, 78.4% of total endemism) than in the 100–1,000 m interval (3 species, 8.1%) or below 1,000 m (5 species, 13.5%), in line with results obtained for cumaceans. The circalittoral zone was the region with highest anthozoan species richness (61.8% by numbers of species) followed by the infralittoral (57.6%) and bathyal (40%) zones (File S2). Half of the total number of species were restricted to one of the infra-, circa-, or bathyal zones, and 9.7% were eurybathic, while the remaining species (40%) were intermediate in depth distribution. We also found exceptions to the pattern of decreasing diversity with depth. The bathymetric range of Mediterranean sipunculans was generally quite wide [229]. Most of the Mediterranean records were bathyal, whereas there were few sublittoral records (File S2).

Other studies carried out on depth-related distribution of marine biodiversity in the deep sea of the Mediterranean available form the literature suggest a generally unimodal pattern of species richness, the highest values of which are observed at intermediate depths (about 2,000 m) and lower values at upper bathyal (<2,000 m) and abyssal (>2,000 m) plains [230], [231]. More recent studies, however, have demonstrated that such patterns are not always recognizable [e.g., [223]–[233]]. In open slope systems, bathymetric gradients of species diversity have been widely documented [e.g., [230]–[234]]. In the Mediterranean, nematode diversity also decreases with depth (Figure 4b), but the degree of species decrease is limited and ample ranges of biodiversity are observed at the same depth. These results suggest that the eurybathy of the Mediterranean fauna (3,613 species) could be lower than previously reported [235]. For example, analysis of all the existing nematode diversity data from the Aegean Sea showed that there is a gradual increase of diversity with depth from the littoral zone down to the bathyal areas (2,000 m) (N. Lampadariou, personal observation). Complementary information on bathymetric patterns of the deep Mediterranean fauna are explored with detail in [180].

### Temporal trends

Available data from the literature show that environmental factors have led to profound changes in the abundance, distribution, and composition of Mediterranean marine species in the distant past [e.g., [19],[33],[87]]. For example, during the Cretaceous, the Mediterranean Sea (called Tethys) was connected to the Atlantic on its western side and the Indo-Pacific on its eastern side. The two oceans contributed very different faunas to the Tethys. During the Miocene, the Tethys was isolated from the Indo-Pacific Ocean and at the Messinian stage, the connection with the Atlantic Ocean was also closed. During this Messinian salinity crisis, the Mediterranean underwent severe desiccation that drove most species to extinction. Although some shallow areas remained on the two sides of the Siculo-Tunisian Strait, and there were many allopatric speciations [19], [236], [237], the reopening of the Strait of Gibraltar 5 million years ago led to restocking of the Mediterranean with fauna and flora from the Atlantic. Up to the nineteenth century, the Mediterranean had been connected with the eastern Atlantic Ocean only.

In this section, however, we summarized main changes since the end of the last ice age (approximately 12,000 years ago). During this time there were notable climate-driven fluctuations but also human-induced changes due to the long periods of exploration and exploitation, and more recently the reopening to the Red Sea through the Suez Canal, the globalization of commerce and trade, increasing pollution and eutrophication of coastal areas, habitat modification and loss, and finally the looming climate change.

Early evidence of human interaction with marine fauna in the Mediterranean Sea comes from the Paleolithic period and continues through the Mesolithic and Neolithic periods (approximately 20,000–4000 B.C.). Zooarchaeological remains are found in Franchthi Cave in the southern Argolid, Greece [238], Las Cuevas de Nerja in southern Spain [239], Athlit Yam, a submerged site south of Haifa Bay in Israel [240], Cape Andreas Kastros in Cyprus [241], and the Strait of Gibraltar [242]. In Greece, fish bones of large tuna, Sparidae and Mugillidae, were found. Zooarchaeological remains in Spain include 20 taxa and show changes in mean fish size and range over time that have been considered as indication of overfishing. At Cape Andreas Kastros in Cyprus and in Athilit Yam, 90% of the remains are grey trigger fish (*Balistes capriscus*), which points to intensive fishing regardless of size. In Gibraltar, remains of Mediterranean monk seals and mollusks consumed by humans were found. However, stable isotope analyses of human bones show that between 10,000 and 8000 B.C., the main Mediterranean coastal populations did not rely significantly on marine food [243], [244].

Since the fifth century B.C., humans have exploited marine resources. Aristotle, in his zoological works dating to the fourth century B.C., focuses his scientific interest on fish and invertebrates exploited by humans in various ways [245]. Fisheries in the Aegean communities by that period are characterized by variability both in the nature and abundance of the exploited fish and in the manner of their exploitation [246]. Mollusks and other invertebrates are part of the diet of ancient Greeks, and their consumption is connected with the treatment or prevention of various health problems and diseases [247]. Bath sponges of the genera *Spongia* and *Hippospongia*, collected by skillful divers, are widely exploited for household and personal hygiene purposes, and play a principal role in medical practice [248].

Commercial fishing and fish processing activities play an important role in the Pontic economy. The export of fish and fish products, including salt-fish (*tarichos*) and fish sauce (*garum*) mainly from European anchovy to the Aegean Sea, continue into the Roman period [249]. These products are exported from the western Mediterranean, but g*arum* is forgotten in the west by the tenth century, although it is still prepared in Constantinople in the fifteenth and sixteenth centuries [250]. Naval trade traffic becomes intense, and invasions of islands from the mainland are already common, and they result in the beginning of the introduction of alien species in those ecosystems. Some of these introductions (rats, carnivores) trigger the extirpation of many seabird colonies, and they have shaped the current distribution of several seabird species [251], [252].

Seafood becomes increasingly popular toward the end of Roman domination, probably because of the proximity of, and access to, marine resources. There is historical evidence of overfishing in some parts of the Western Mediterranean in the early Imperial period [253]. Even then, certain fishing techniques are prohibited to manage or counteract the decline in fish stocks (such as fishing by torch lights at night), and efforts are made to boost natural availability with introduced fish and shellfish stocks. For example, the parrot fish (*Sparisoma cretense*) is captured in the Aegean Sea and released in the Tyrrhenian Sea [253], [254]. There are also pictorial remains that show fishing gear and a large variety of targeted species during Roman times. Gastropods [255], the red coral *Corallium rubrum* [256], and several species of sponges [257] were exploited on an industrial scale.

Fishing, fish processing, industrial exploitation of several marine species, and development of improved fishing gear continue during the Byzantine period [253]. Various literary sources point out that targeted species, among them the currently overfished tuna, are conspicuous. There is a 200-year gap between the Moslem conquest of the Near East and northern Africa and the appearance in the ninth century of the first Arabic written sources [250]. In northern Africa, the first written evidence dates from the tenth century and refers to fishing gear used to catch mullets, Atlantic bluefin tuna (with large spears), and fish in shallow waters [258]. Zooarchaeological material from the Israeli coastline dating from the Byzantine through the Moslem Crusader and Mamluk periods (fourteenth century) points to a high consumption of marine and freshwater fish that are still fished in Israel today, such as the thin-lipped grey mullet (*Liza ramada*), Sparidae, and the parrot fish [250]. There is noticeable fishing activity dating from the Byzantine, Moslem (tenth century), and later Norman periods (eleventh to thirteenth centuries) in southern Italy and in Sicily, where Atlantic bluefin tuna is the main target species exploited by traps (*tonnara*) [259].

Harvesting of the gastropods *Hexaplex trunculus* and *Bolinus brandaris* is an example of the successive exploitation of marine resources from the Iron Age until the thirteenth century in the Eastern Mediterranean. These species are specifically harvested for the purple pigments extracted from their shells and used to dye clothes. This harvest disappear from the Levantine area in the late twelfth century, and from Greece a century later, although both species are still abundant to this day [250]. Another example of human exploitation of marine resources from historical times is the hunting of seabirds on islands, particularly of shearwaters, which probably constituted the only source of protein in periods of scarcity especially on small islands. In places such as Formentera (Balearic Islands), humans contribute to the depletion, and partial extinction, of Balearic shearwaters (*Puffinus mauretanicus*), with consequences at the level of the marine trophic web [260].

Human impacts on marine biodiversity grow increasingly stronger as the Mediterranean cities and ports continue to grow and more recent centuries witnessed substantial advances in technology. It is assumed that since the fourteenth century, the adoption of new fishing methods (such as the *tonnara*, a sort of drift net mainly used for tuna fishing) in the Western Mediterranean, their spread to southern Italy [261], [262], and their introduction to the Adriatic in the seventeenth century [261], [263] increase fishing catches. Fishing catches increase to an extent that even the early fishermen organizations (sixteenth century), such as *Cofradias* in Catalonia [262] and the *Prud*'*homies* in Provence [264], are concerned about possible negative effects on exploited stocks. Such effects are further intensified by the increasing industrialization in the nineteenth century, with an increase in the efficiency of existing fishing gear (e.g., otter trawl) and the introduction of new ones (such as midwater pelagic trawls, hydraulic dredges, and iron-toothed dredges). Industrialized fishing had severe impacts on species, habitats, and ecosystems [265]. Several studies also show historical changes in fish communities of different regions of the basin [e.g., [25],[123],[266]–[268]]. These findings point to a general severe depletion of top predators in the basin, including Atlantic bluefin tuna, which is considered critically endangered according to the declining trend observed in the Atlantic and the Mediterranean in the last 50 years. Historical fluctuations in the abundance of this species have been described on the basis of a centuries-long time-series of tuna trap catches, starting in the seventeenth century, and suggested to be linked to climate fluctuations [269].

Despite this comparative wealth of historic information about temporal trends mainly linked to the history of human exploitation of Mediterranean marine biodiversity, many unknowns remain in spatial and chronological gaps from prehistoric periods to the present. Ancient, medieval, and early modern records contain qualitative rather than quantitative data, and it is difficult to depict general diversity trends at either a species or ecosystem level at the scale of the whole Mediterranean.

Interesting results do emerge from analyses of specific regions. The overall trends reported by Lotze et al. [113] for the north Adriatic Sea indicated that prehistoric people had no measurable effect on marine resources around this basin (Figure 8a, see File S2 for species included in the analysis). This changed during the Classical period (500 B.C. to A.D. 600) [270], and especially during Roman times, when reports of species depletion and overexploitation in coastal waters increased. It is possible that marine species recovered from heavy exploitation after the collapse of the Roman Empire, as has been documented for terrestrial resources [33]. However, human population increased during the Medieval period (approximately A.D. 600 to 1500), increasing the pressure on marine resources. With the onset of the industrialization in Europe in the nineteenth century, signs of species depletions and rareness increased and accelerated throughout the twentieth century, when the first extirpations of species were also recorded. Biodiversity did not decrease, however, because some species were newly introduced into the Adriatic Sea [271]. No temporal trend is known for alien species in the Adriatic Sea, so we showed (Figure 8a) a timeline of mollusk invasions in the Mediterranean as a whole [272], which started in the late nineteenth century and accelerated during the twentieth century. The depletion of formerly abundant species and the invasion of new species caused a shift in species composition and diversity in the north Adriatic Sea [113]. Local species depletions and extirpations mostly occurred among large species, including marine mammals, birds, reptiles, and commercial fish and invertebrates, while species invasions were mainly by smaller species at lower trophic levels, such as invertebrates and algae (Figure 8b). Such fundamental changes in species composition had effects on the structure and functioning of food webs and ecosystems [113], [273].

**Figure 9:**
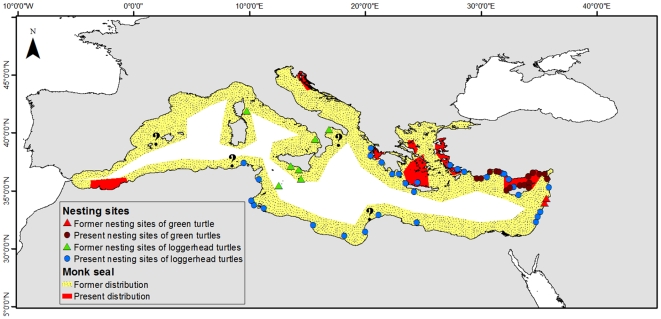
Distribution of monk seals and nesting sites of marine turtles in the Mediterranean. Present (red areas) and historical (yellow areas) distribution of the Mediterranean monk seal [22], [23], [101], [106], [117]–[119], and nesting sites for loggerhead turtle and green turtle [modified from 22]. Green and red triangles, respectively, are the former nesting sites for loggerhead turtle and green turtle; green and red dots are the present sites. Question marks represent sites where one or a few Mediterranean monk seals have been recently seen.

Population declines have also been noted among marine mammals throughout the Mediterranean. These species include sperm whales, which have been declining since the end of the 1980s [274]; short-beaked common dolphins, which began to decline around the 1970s [93], [275]; common bottlenose dolphins, which have decreased by at least 30% over the past 60 years [97], [276]; and striped dolphins, which have been in decline since the early 1990s [277]. The Mediterranean monk seal, in particular, was deliberately hunted during the Roman period [278], and it disappeared in the greatest part of the Mediterranean basin during the early 1900s [279], [280]. Currently, it mainly occurs in small, isolated areas of the Greek and Turkish coasts, and northwest African coastal waters (Figure 9), but the presence of Mediterranean monk seal in some of these areas is uncertain. There are fewer loggerhead and green turtles throughout the Mediterranean, although historical records were available to determine the severity of their population decline [22], [95]. Known nesting sites especially for the loggerhead turtle disappeared in several areas of the basin [22] (Figure 9).

Although the population trends for most seabird species are not well known, all reliable long-term information suggests that most seabird species have recovered on the European coasts during the last three decades. This recovery is due to more restrictive conservation policies at national and international levels. With the exception of shearwaters, seabird species show relatively stable population trends. Gulls and terns, after two decades (1980s and 1990s) of sharp increase in their densities (up to an average 13% annual growth rate in Audouin's gull) [171], are now in dynamic equilibrium [281]. Sparse data on shags suggest a slow recovery in the last two decades. Storm petrel populations are stable at the few long-term monitored sites [282], but many suitable breeding sites have been destroyed since historical times along coastlines. Paleontological records confirm that the distribution of many species was much larger, even occupying habitats in the interior of large islands relatively far from the sea, where recolonization is now impossible [283]. Population recoveries of Mediterranean seabirds must be considered only partial, and only occurring where protection is effective [284].

### Threats to diversity and hot spots

As shown above, anthropogenic factors have influenced the general patterns and temporal trends of Mediterranean marine diversity with varying degrees of intensity. Quantifying the importance of each threat is essential for future analysis.

Lotze et al. [113] provided data to evaluate the human impacts that caused or contributed to the depletion or extirpation of species in the north Adriatic Sea over historical time scales. Exploitation stood out as the most important factor causing or contributing to 93% of depletions and 100% of local extinctions or extirpations (Figure 8c). Habitat loss or destruction was the second-most-important human impact, followed by eutrophication, introduced predators, disease, and general disturbance. While 64% of depletions and 88% of local extinctions were caused by a single human impact, in all other cases the combination of two or several human causes was responsible for the decline or loss. This highlights the importance of cumulative human impacts, especially in coastal ecosystems, with emphasis on species with commercial interest.

**Figure 10:**
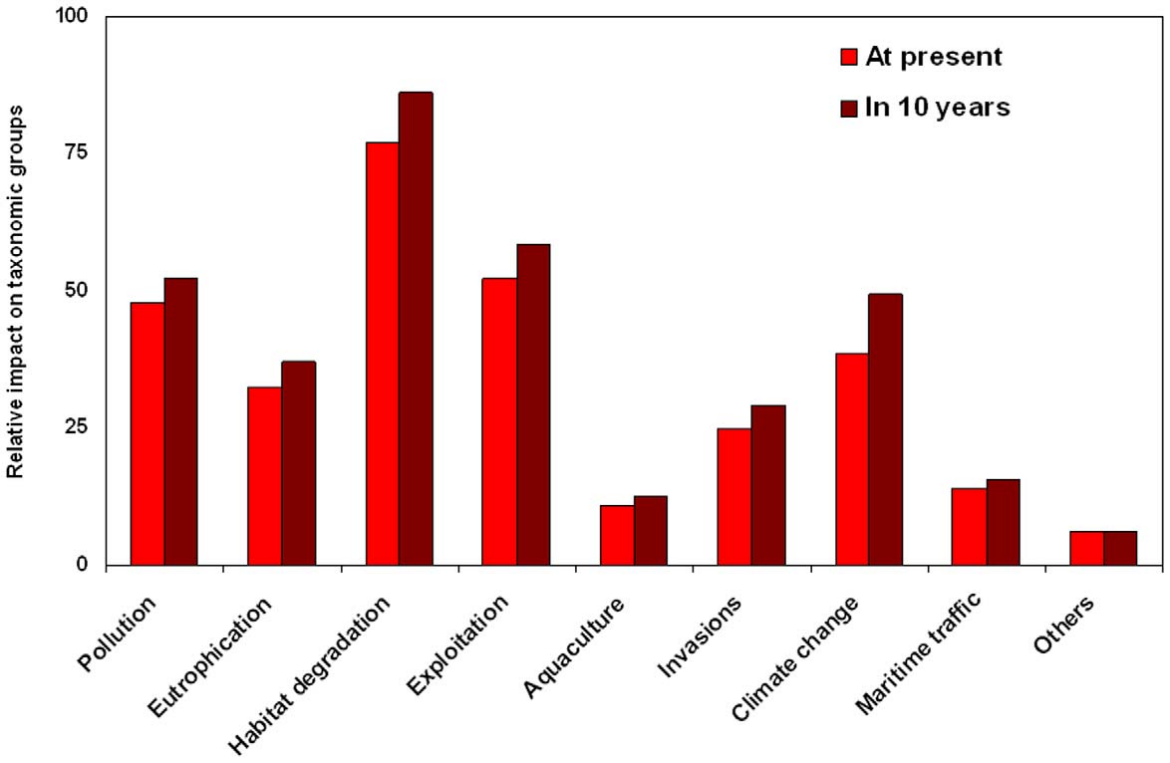
Current and future threats to biodiversity in the Mediterranean Sea. We used published data on specific taxa and expert opinion. Threats to diversity were ranked from 0 to 5 for 13 taxonomic groups and results are shown as the percentage of the ranking to the maximum values (File S2).

Recently, anthropogenic drivers and threats to diversity increased and further diversified in the Mediterranean, as observed elsewhere [285]. Published information and the opinion by experts identified and ranked current threats to diversity in the Mediterranean (Figure 10, and File S2). The sum of the ranking (0–5 for each threat) showed that for 13 large taxonomic groups, habitat loss and degradation are considered the primary impact on diversity, followed by exploitation, pollution, climate change, eutrophication and species invasions. These were the most conspicuous threats and also affect the greatest number of taxonomic groups. Other threats to diversity were maritime traffic (collisions with vessels) and aquaculture. Within 10 years from now, habitat degradation and exploitation were predicted to retain the predominant roles, while pollution and climate change will likely increase in importance, followed by eutrophication. Of all current threats to biodiversity in the Mediterranean, climate change was predicted to show the largest growth in importance within the next 10 years (10.8%), followed by habitat degradation (9.2%), exploitation (6.2%), and pollution, eutrophication, and invasion of species (4.6% each) (Figure 10).

**Figure 11:**
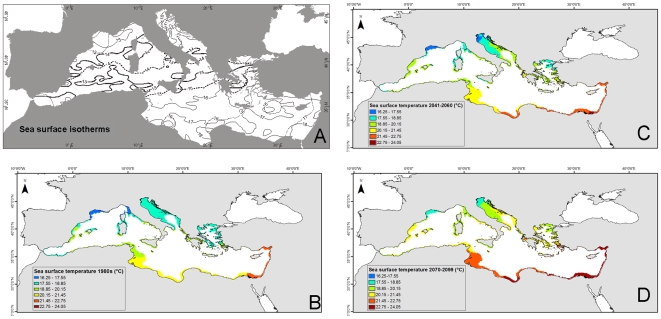
Past changes in seawater temperature and future projections in the Mediterranean Sea. (A) recent northward shifting of February sea surface isotherms (°C) in the Mediterranean Sea (broken lines are the one-century climatological means, solid lines the means for 1985–2006: the 14°C and the 15°C “dividers” are highlighted by a thicker tract. Data compiled from MEDATLAS, GOS-MED, NOAA-AVHRR data and various other sources. Seawater surface temperature on the continental shelves is shown (B) during the 1980s (according to the NOAA data), (C) by 2041–2060, and (D) by 2070–2099 [according to the OPAMED8 model based on the A2 IPCC scenario, 120]. The size of the cell is 0.1×0.1 degree.

Figure 11 shows past changes and projected future increases in sea surface temperature (SST) in the Mediterranean Sea. The 15°C isotherm, whose one-century climatological mean crosses the Straits of Sicily, may have moved northward in recent times (Figure 11a). This can imply that a number of tropical Atlantic species that entered the Mediterranean during the last interglacial (125,000 to 110,000 years ago) will reenter the Western Mediterranean in the near future [286]–[288]. In the meantime, in the Western Mediterranean, the “14°C divide” [289], the one-century climatological mean of the surface isotherm for February that coincides with a frontal system created by mesoscale eddies in the Algerian Basin [290] and that may act as a barrier to dispersal, has apparently moved northward in recent times (Figure 11a). The southern sectors of the Mediterranean harbor many native warm-water species that do not occur or get much rarer in the northern sectors. These “southerners” are apparently confined by the 14°C divide. Perhaps not coincidentally, many of these native but “meridional” warm-water species have colonized the northern sectors, which are thus facing a process of “meridionalization” [e.g., [286],[291],[292]]. In addition, the mean SST made in early 1980s (Figure 11b) revealed that the warmest area of the Mediterranean was the Levantine Basin, with a mean SST of 21.8°C, and the coolest areas were the Gulf of Lions and the Ligurian Sea, with a mean SST of 16.9°C. Climate models predicted that by 2041–2060, the major part of the Mediterranean will become warmer except the northern Adriatic, which is expected to become cooler (OPAMED8 model based on the A2 IPCC scenario, Figure 11c). By 2070–2099, the Mediterranean is projected to warm by 3.1°C (Figure 11d), the last cool enclaves being the Gulf of Lions and the northern Adriatic, with a mean SST of 18°C.

**Figure 12:**
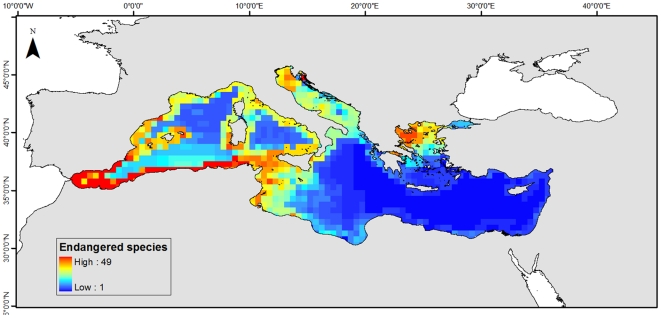
Biodiversity hot spots for Mediterranean vertebrate species of special conservation concern. This figure includes 110 critically endangered, endangered, vulnerable, or near threatened species. Results are predictions based on AquaMaps model [80, and File S2] and generated using a probability threshold of occurrence of ≥0.4 to highlight likely areas of critical habitat for each species. Colors express species occurrence from blue (little occurrence) to red (highest occurrence). The size of the cell is 0.5×0.5 degree.

Taking into account data regarding marine biodiversity and threats, we mapped vertebrate endangered species and have tried to locate potential hot spot areas of special concern for conservation in the Mediterranean (Figure 12). The first attempt included fish, marine mammals, and sea turtles, which are considered important sentinels for ocean health. The identified hot spots highlighted the ecological importance of most of the western Mediterranean shelves. The Strait of Gibraltar and adjacent Alboran Sea and African coast were identified as representing important habitat for many threatened or endangered vertebrate species. The most threatened invertebrate species in the Mediterranean, the limpet *Patella ferruginea*, is also distributed along this area [293]. Both the northern Adriatic and Aegean seas also showed concentrations of endangered, threatened, or vulnerable species. Other equally species-rich waters along the northeast African coast, and the southern Adriatic Sea, were of lesser concern for the protection of endangered species.

## Discussion

### Estimates and patterns of marine diversity in the Mediterranean Sea

Our estimate of 17,000 species for marine biodiversity in the Mediterranean updated and exceeded previous values, which were on the order of 8,000–12,000 species (Table 3). In comparison with the 1992 estimate [15], the total number of recorded species has increased substantially. As a result of recent efforts and improvements in analytical methods and instruments, our estimates of invertebrates and protists, in particular, have undergone an upward revision in recent years. Current estimates of sponges, cnidarians, polychaetes, mollusks, arthropods, echinoderms, ascidians, and other invertebrates all exceed those dating back to the early 1990s. However, since most microbial diversity is basically unknown, global numbers and their evolution are uncertain.

**Table 3:** Group-specific biodiversity estimates for the Mediterranean marine biota through time [16], [19], [68], including the current estimate (estimate 2009), and online free-access global databases [71], [77]–[81].

	Datasets for the Mediterranean Sea				Global datasets**		
	MEDIFAUNA 1992^1^	Bianchi and Morri 2000^2^	Boudouresque 2004^3^	Current estimate 2009^4^	AquaMaps^5^	FishBase & SeaLifeBase^6^	OBIS 2009^7^
Benthic primary producers^8^	0	1086	1034	1131	260	0	0
Invertebrates	6338	6575	7287	10901	3445	2088	193
Vertebrates	694	639	694	693	613	618	493
Bacteria, Protists and Fungi	0	265	2985	Approx. 4400	10	0	0
Total	7032	8565	12000	16848	4328	2706	686

**Queried July 2009.

1 [15];

2 [19];

3 [16];

4 Table 1;

5 [80];

6 [78];

7 [77];

8 Heterokontophyta, Rhodophyta, Chlorophyta and Magnoliophyta.

Estimates from global databases that include Mediterranean information up to September 2009 range from 4% and 25% of the total species diversity estimated in our study (Table 3). They covered vertebrate taxa fairly comprehensively, but other taxonomic groups were underrepresented. WoRMS included 8,562 records of Mediterranean marine species, which represented 50% of species registered in this study. Mediterranean databases such as ICTIMED (specialized in fish diversity) included about 70% of fish diversity reported in our study.

Total estimates of Mediterranean species of macrophytes and metazoans represented 6.4% of their global counterpart (Table 4). Macrophytes showed the highest percentage of shared species with global estimates, and Heterokontophyta and Magnoliophyta scored the highest (17.2% and 11.7%, respectively). Among metazoans, Mediterranean sponges showed the highest percentage (12.4%), followed by polychaetes (9.4%) and cnidarians (7.7%). Other groups represented much lower percentages of the total, such as echinoderms (2.2%), fish species (4%), and mollusks (4%).

**Table 4:** Number of Mediterranean species of macrophytes and metazoans, global number of marine species, and percentage of Mediterranean species with respect to their global counterparts.

Taxa	No. species this work	No. species worldwide*	%
**Macrophytes**			
Phaeophyta	277	1600	17.31
Chlorophyta	190	2500	7.60
Rhodophyta	657	6200	10.60
Magnoliophyta	7	60	11.67
**Metazoans**			
Porifera	681	5500	12.38
Cnidaria	757	9795	7.73
Platyhelminthes	1000	15000	6.67
Mollusca	2113	52525	4.02
Annelida	1172	12000	9.77
Crustacea	2239	44950	4.98
Bryozoa	388	5700	6.81
Echinodermata	154	7000	2.20
Ascidiacea	229	4900	4.67
Other invertebrates	2168	18565	11.68
Vertebrata (Pisces)	650	16475	3.95
Other Vertebrata	43	481	8.94
**Total**	**12725**	**203051**	**6.27**

*Based on Bouchet [82], Green and Short [26], and Groombridge and Jenkins [83].

Previous studies claim the existence of a gradient of species richness from the northwest to the southeast Mediterranean [e.g., [90],[251],[294]–[297]], in agreement with differences in key environmental variables, such as latitude, salinity, temperature, and water circulation, in addition to the distance from the Strait of Gibraltar. Our results confirmed this general decreasing trend and showed that the distribution of marine diversity in the Mediterranean is highly heterogeneous.

The Western Mediterranean displays the highest values of species richness, likely owing to the influx of Atlantic species and the wide range of physicochemical conditions. The central Mediterranean, Adriatic, and Aegean seas are areas of second-highest species richness, although with exceptions. The Adriatic Sea sometimes displays lower species numbers because of restricted exchange with the western basin, decreasing depth toward the north, the presence of fresh water, and the larger amplitude of temperature variations [297], [298]. However, this basin shows a large number of endemics possibly owing to its higher isolation. The Aegean Sea normally follows the western areas, mainly because of its more direct exchange with the western basin and its higher habitat diversity [297], [299], [300]. The Levantine Basin and southeastern side have in general the lowest species richness, which is due to the unfavorable conditions prevailing in the area (such as high salinity) as well as the less intensive sampling effort [297], [301].

In fact, a lack of data is evident in several eastern and southern regions of the Mediterranean basin. This may have strongly influenced some of our results regarding spatial patterns, so generalizations have to be made carefully. Marine research in the Mediterranean has been regionally biased, reflecting sparse efforts along the southern and easternmost rim. It has even been suggested that the relative species richness of different taxa by sector of the Mediterranean is a better indicator of the level of research effort than of true species richness [302]. Therefore, as new species are assessed in the eastern and southern areas, patterns may be modified. Moreover, the diversity in the eastern end is more influenced by species introductions. The Suez Canal, opened in 1869, has restored the connection between the Mediterranean and the Indian Ocean [303], and in recent years we have witnessed an exponential increment in the number of Indo-Pacific species recorded in the Eastern Mediterranean [e.g., [88],[304]]. This trend will continue to influence the biodiversity of the Mediterranean Sea.

In addition, the data used to draw spatial patterns were collected from the 1980s to 2000s, so results may differ from the current situation and may represent potential ranges and values rather than current ones. However, similarities exist between results achieved with distribution maps drawn with expert data and predicted results using AquaMaps models. These similarities indicated that the species richness maps resulting from this study are a useful first attempt to represent comprehensive species richness patterns at the Mediterranean scale. Differences encountered using both methods may be due to limitations of the data. By their nature, expert-drawn maps or sightings often represent underestimates of total species distributions because of the absence or lack of effort in certain areas (in our case the southern shorelines of Mediterranean along the coasts of northern Africa and the eastern sites) and the inability to detect rarer species without sufficient efforts. On the other side, AquaMaps model predictions do not currently factor human impacts or ecological interactions and may be closer to fundamental or historical niche rather than realized niche. Therefore some AquaMaps predictions may represent overestimates (a good example is the Mediterranean monk seal; see www.aquamaps.org). Besides, the relative probability of occurrence calculated from AquaMaps does not distinguish between a rare species that might only have been sighted once in a given cell, and a more abundant species that might be sighted every day. AquaMaps rely exclusively on data accessible through OBIS/GBIF, which currently contains few Mediterranean records. Therefore, for many species, occurrence was inferred from habitat use outside of the Mediterranean. Because the Mediterranean environment represents some environmental extremes (such as salinity and temperature records), occurrences in the eastern part may not have been captured adequately by AquaMaps, and this could partially explain the low values in this region. These limitations are extended to our first attempt to depict hot spot areas in the Mediterranean. The eastern region hosts important populations of elasmobranchs and marine mammals that are currently threatened, but their probability of occurrence estimated by AquaMaps model is lower than 0.4. Further studies should be able to reconcile both mapping sources and confirm or correct patterns.

Explanations for the observed heterogeneity of species richness in the Mediterranean Sea include the threshold of the Siculo-Tunisian Strait that divides the Mediterranean into two basins, and the paleo-biogeographical history of the Mediterranean Sea. The western basin shows more biological similarity with the Atlantic Ocean, hosting a higher number of cold-temperate species, while the eastern basin shows more biological similarities with the Indo-Pacific, and hosts a larger number of subtropical species. The Siculo-Tunisian Strait still partially acts as a barrier to the dispersal of many species between the two basins and constitutes their meeting point.

Diversity differences between areas may also reflect changes in water masses and circulation [305], [306] as well as changes in temperature and salinity [307]. The diversity of some groups is definitively influenced by this temperature gradient. For the sipunculans, richness may be linked to the temperature of the water masses during the year [289], which reflects a physiological barrier between cold and warm water for cold- and warm-water species. For example, *Golfingia margaritacea* is mainly a temperate and boreal species [229], and its presence in the Mediterranean may indicate the prevalence of colder water masses. In contrast, other thermophilic species, such as *Phascolion convestitum* and *Aspidosiphon elegans*, have been proposed as Lessepsian migrants [229], [308].

Diversity distribution in the Mediterranean is also associated with a productivity gradient. Higher productivity areas show higher diversity partially because they are important feeding and reproductive sites for several taxa. Most of these areas occur in the Western Mediterranean and the northern Adriatic that, for example, host many species of fish, seabirds, marine mammals, and turtles [e.g., [91],[110],[309]]. Their distribution is associated with feeding habits [e.g., [92],[93],[97],[276],[280]]. Moreover, some fish, seabirds, sea turtles, and mammals show opportunistic feeding behavior, exploiting discards from trawling and purse seines, and to a lesser extent from artisanal long-lining [e.g., [310]–[312]]. In developed Mediterranean countries, discards from trawl fishing can be up to 400% of the commercially valuable catches, and such amounts of food, which may be predictable in space and time, are scavenged by many species. Most Mediterranean marine mammals are predominantly offshore and prefer deep-water habitats, but a few species can venture to inshore waters and scavenge fishery discards [97], [309], [313].

The three main categories explaining the drivers of biodiversity in the deep Mediterranean are (i) bathymetric gradients, which are associated with increasing pressure and decreasing food availability in deeper sediments; (ii) geographical and physicochemical features, which are responsible for the north-northwest–south-southeast gradient in trophic conditions; and (iii) environmental heterogeneity (e.g., grain size distribution, habitat complexity, distribution of food inputs) [179], [180]. Our understanding of the mechanisms driving deep-sea biodiversity patterns is still limited, but some of the factors frequently invoked are (a) sediment grain size and substrate heterogeneity [231]; (b) productivity, organic content, or microbial activity [314]; (c) food resources [233]; (d) oxygen availability [315]; (e) water currents [185]; and (f) occasional catastrophic disturbances [219]. Thus, the spatial distribution of available energy may influence the distribution of benthic abundance, biomass, and biodiversity [9], [184], [196], [219], [316]–[318]. Food availability depends almost entirely on the supply of energy from the water column and decreases with depth, which may explain most of the variability between the observed spatial patterns of the benthic biodiversity in the deep Mediterranean Sea.

### Threats to diversity

In the past, geological and physical changes lie at the root of the most dramatic changes in biodiversity in the Mediterranean Sea. Today, human activities are essential elements to consider as well, and several of them threaten marine diversity. The most important threats in this region are habitat loss, degradation and pollution, overexploitation of marine resources, invasion of species, and climate change.

#### Habitat degradation, pollution, and eutrophication

Our results show that habitat degradation and loss is currently the most widespread threat and was also important in the past. Human interventions, such as coastal modification, that can be traced back to before the Roman period [75], have important consequences for diversity. Coastal development, sediment loading, and pollution reduced the extent of important habitats for marine diversity, such as seagrass meadows, oyster reefs, maërl, and macroalgal beds, and affected Mediterranean ecosystem functioning well before the 1900s [319]–[321]. Most species depend strongly on their habitats (such as bryozoans, sponges, echinoderms, benthic decapods, and organisms of the suprabenthos and meiobenthos); hence, its loss and degradation have major effects on marine diversity.

Cultural eutrophication, in particular in semienclosed basins such as the Adriatic Sea, can also be traced back for centuries [322], [323]. This phenomenon reached its peak in the late 1980s [323] and, in addition to fishing, may be the cause of the sequence of jellyfish outbreaks, red tides, bottom anoxia events leading to benthic mass mortalities, and mucilage events that have occurred in recent ecological history of the Adriatic Sea [324]. Direct and indirect pollution is generated directly from the coast, or through fluvial contributions, and ends up in the sea [5]. Pollution affects a wide range of marine species [e.g., [110],[252],[325]–[328]] and is of primary concern for the conservation of the deep-sea ecosystems [180].

The main threats for most seabirds and marine turtles in the Mediterranean arise from habitat degradation and loss [110], [252]. The breeding habitat for seabirds is relatively well protected along the northern Mediterranean shore, but the protection of many seabird colonies and hot spots is less effective along the southern shore because of limited resources. Marine wind farms, which are expected to increase in some countries, may represent a new conservation concern for seabird populations [329]. Marine turtles are also affected primarily by degradation of habitats but also by marine pollution, driftnets, gillnet and longline by-catches, and boat strikes [22], [95], [330]. The continuing increase of coastal settlements is important for the region's economic activity, but it is also causing intense environmental degradation through excessive coastal development, further pollution, and consumption of natural resources, all of which add pressure to coastal areas and the marine environment [46].

#### Exploitation of marine species

This study also illustrates that the oldest and one of the most important maritime activities that has become a threat to diversity is human exploitation of marine resources. People around the Mediterranean have exploited marine resources since earliest times. Maybe not surprisingly, negative effects of the exploitation of the Mediterranean marine biodiversity were first reported in the fourth century B.C. by Aristotle. He mentioned that scallops had vanished from their main fishing ground (Gulf of Kalloni, in Lesvos Island) since fishermen began using an instrument that scratched the bottom of the sea [247]. Early records of overfishing and depletion of coastal resources become evident during Roman and medieval times and are driven by human population growth and increasing demand and the increasing commercialization and trade of food and products [113], [115].

The current high demand for marine resources continues and has resulted in high levels of fishing or harvesting intensity. Several fish resources are highly exploited or overexploited [e.g., [25],[331]–[335]]. Other organisms that are exploited or affected by exploitation in the Mediterranean include macrophytes, sponges, cnidarians, echinoderms, mollusks, arthropods, polychaetes, ascidians, and other invertebrates (File S2) [e.g., [257],[336]–[342]].

The threats to currently endangered marine mammals and sea turtles include unwanted by-catch [121], [265] as well as historical exploitation. For sea turtles, the overall mortality rate caused by entanglement in fishing gear and by habitat degradation is poorly known [95], but for marine mammals the major threats clearly derive from human activities: direct or indirect effects of exploitation, such as prey depletion, direct killing, and fishery by-catch [97], [122], [275], [277], [343]–[345]. At sea, threats to seabirds mainly come from fisheries [346]–[347], particularly by-catch in longlining [172], [348].

Fishing is being expanded toward deeper areas and is threatening several ecosystems [e.g., [265],[349],[350]], while management effectiveness in the Mediterranean is low [351], [352]. Fishing activity may also be the cause of ecosystem structural and functional changes and ecosystem degradation [e.g., [273],[353]–[355]].

#### Bioinvasions

A few Mediterranean invasive aliens have drawn the attention of scientists, managers, and media for the conspicuous impacts on the native biota attributed to them. A pair of coenocytic chlorophytes, *Caulerpa taxifolia* [356] and *C. racemosa* var. *cylindracea* [357], are the most notorious invaders due to their high impact on marine benthic ecosystems, thus the best-studied invasive species in the Mediterranean. Other work [216] has traced the impacts of invasive aliens that entered the Mediterranean from the Red Sea through the Suez Canal and displaced native species.

Tropical species have been entering the Mediterranean through either the Suez Canal (Lessepsian migration) or the Strait of Gibraltar for decades, and mainly by ship transportation. The Mediterranean is highly susceptible to ship-transported bioinvasions: one-fifth of the alien species recorded in the Mediterranean were first introduced by vessels [216]. In 2006, 13,000 merchant vessels made 252,000 calls at Mediterranean ports, and an additional 10,000 vessels passed through the sea (REMPEC/WG.29/INF.9). The increase in shipping-related invasions may be attributed to the increase in shipping volume throughout the region, changing trade patterns that result in new shipping routes, improved water quality in port environments, augmented opportunities for overlap with other introduction vectors, and increasing awareness and research effort [358]–[359]. The swarms of the vessel-transported American comb jelly (*Mnemiopsis leidyi*) that spread across the Mediterranean from Israel to Spain in 2009 raise great concern because of their notorious impacts on the ecosystem and fisheries [ansamed.info and 360].

Moreover, with the development of large-scale marine aquaculture (mariculture) in the late twentieth century, the commercially important alien shellfish *Crassostrea gigas* and *Ruditapes philippinarum* were intentionally introduced to the Mediterranean. The high permeability of aquaculture facilities, transport, and transplantation of these species have resulted in many unintentional introductions: oyster farms have become veritable gateways into Mediterranean coastal waters for alien macrophytes [213]. The massive “official” and “unofficial” importation of foreign spat (young bivalves both before and after they become adherent) in the 1970s and 1980s coincided with a marked increase of alien species around oyster farms, and the aliens were considered to have arrived with the oysters [361]. Segments of the industry may still resort to illegal importation: neither the Turkish authorities nor the UN Food and Agricultural Organization were aware of the importation of the bilaterally ablated female banana prawn (*Fenneropenaeus merguiensis*) that was found in the Bay of Iskenderun, Turkey [362].

Although some aliens are responsible for reducing the population of some native species [363], others have become locally valuable fishery resources [364]. Some Erythrean aliens were exploited commercially almost as soon as they entered the Levantine Sea, and their economic importance was quickly acknowledged [365]. Levantine fisheries statistics record the growing prominence of the Erythrean aliens: the Erythrean prawns are highly prized and, beginning in the 1970s, a shrimp fishery developed in the Levantine Sea. Nearly half of the trawl catches along the Levantine coast consist of Erythrean fish, but the commercially exploitable species were accompanied each summer by swarms of the scyphozoan jellyfish *Rhopilema nomadica*, washed ashore along the Levantine coast. The shoals of jellyfish adversely affect tourism, fisheries, and coastal installations, and severe jellyfish envenomations require hospitalization. The recent spread of the silver stripe blaasop (*Lagocephalus sceleratus*) and the striped catfish (*Plotosus lineatus*) pose severe health hazards. Other work [216] has traced the impacts of invasive aliens that entered the Mediterranean from the Red Sea through the Suez Canal and displaced native species.

Pronounced thermal fluctuations and a significant increase in the average temperature of the waters in the Mediterranean during the past two decades have coincided with an enlarged pool of warm-water alien species that have become established and expanded their distributions (see next section). These thermophilic aliens have a distinct advantage over the native Mediterranean biota. Though no extinction of a native species is yet attributable to invasion of new species, sudden declines in abundance, concurrent with proliferation of aliens, have been recorded [216]. Examination of the profound ecological impacts of some of the most conspicuous invasive alien species underscores their role, among many anthropogenic stressors, in altering the infralittoral benthic communities. Local population losses and niche contraction of native species may not induce immediate extirpation, but they may trigger reduction of genetic diversity and loss of ecosystem functions and processes, and habitat structure.

#### Impacts of climate change

Climate change is exerting a major effect on Mediterranean marine biodiversity through seawater warming [e.g., [366]–[372]]. The increase in seawater temperature has affected the distribution and abundance of native and alien species, and has had both direct and indirect effects on invertebrates and fish [e.g., [373]–[379], see File S2]. The increase in water temperature in the Mediterranean also alters jellyfish population dynamics [e.g., [380]] and may act in addition to indirect fishing impacts [e.g., [381]].

Seawater of the Mediterranean Sea has been warming since at least the 1970s [382], [383]. Rising temperature enlarges the pool of alien species that could establish themselves, enables the warm-water species (native and alien) present in the sea to expand beyond their present distributions, and provides the thermophilic aliens with a distinct advantage over the native Mediterranean biota. The appearance of numerous allochthonous species of tropical origin is leading to what is called the “tropicalization” of the Mediterranean Sea [384]. Although tropical invaders have been recorded in the northernmost sectors of the Mediterranean [e.g., [385],[386]], tropicalization is especially obvious in the southern sectors, where species of tropical origin now form a significant portion of the biota.

Tropical species have been entering the Mediterranean through either the Suez Canal (Lessepsian migration) or the Strait of Gibraltar for decades [201], [387], but they used to remain in the eastern or western basin, respectively. Thus it conformed to the traditional physiographic and biogeographic subdivision of the Mediterranean [367]. However, in the last two decades, the number of tropical species that have also spread through the entire basin is growing. Examples of Erythrean aliens that crossed the Strait of Sicily include algae, a seagrass, many invertebrates and fish [e.g., [216],[388]–[390]]. Species coming from the tropical Atlantic have traveled the opposite way to reach the Levantine Sea [e.g., [50],[391]]. The Strait of Sicily is today a crossroad for species of distinct tropical origins (Atlantic and Indo-Pacific), expanding their range longitudinally within the Mediterranean [370], [392].

If the southern sectors of the Mediterranean are being “tropicalized” (higher occurrence of tropical aliens) and the northern sectors “meridionalized” (increased proportion of indigenous thermophilic species), it is uncertain what will happen to those species of boreo-Atlantic origin, which entered the Mediterranean during glacial periods and have been established in the northern and colder areas of the basin. Because they cannot move farther northward, they may dramatically decrease [393] or even be at risk of extirpation. Although the total extinction of flora and fauna from a basin as wide as the Mediterranean may be unrealistic, the signs of increased rarity or even disappearance of cold-water species deserve further investigation [354], [394]–[397]. An example is the deep-water white coral, *Lophelia pertusa*, reefs of which have become rare in the Mediterranean [61]. These coldest parts of the Mediterranean (Gulf of Lions, northern Adriatic) could act as a sanctuary for cold-temperate species, but if warming intensifies, those areas may act as traps without any cooler water for escape [371].

Global warming may cause thermophilic species of the southern Mediterranean to appear more frequently in the northern and colder parts [e.g., [19],[397]–[399]], and an increasing colonization by southern exotic species may be seen [400]. But there may also be habitat fragmentation and local extinction of species unable to undertake migrations. Lack of (evidence of) species extinctions, coupled with establishment of alien species, is apparently leading to an increased species richness of the Mediterranean, a much debated issue [202]. Richness is increasing at the whole-basin scale (*γ*-diversity), but it is difficult to establish what is happening at local scales (α-diversity) in coastal areas. Instances of species replacement [e.g., [202],[396]–[397],[401]], and mass mortalities due to high temperature or pathogens [e.g., [374],[402]–[403]] and perhaps aliens [404] have been observed. Climate warming, moving physiological barriers and inducing the spatial overlap between alien and indigenous species, causes biotic homogenization [400] and hence a depression in β-diversity. Thus, the relationship between tropicalization, meridionalization, and biodiversity is not straightforward.

In general, the establishment of tropical invasive aliens may cause Mediterranean communities to lose their particular character [405] and to become similar to their tropical analogs, especially in the southern portions of the basin [406]. *Cladocora caespitosa*, the most important shallow-water zooxanthellate species living in the Mediterranean, was more abundant and built more conspicuous formations during periods of the Quaternary, when the Mediterranean climate was subtropical [407]. However, warming episodes in recent summers coincided with mass-mortality events of this coral [e.g., [408]]. Hence, it is unlikely that the Mediterranean in the future will contain significant coral constructions. The overwhelming number of Lessepsian immigrants will move the composition of the biota more and more like that of the Red Sea, but Mediterranean communities will probably look like those that today characterize southern Macaronesia and the Cape Verde region, with scanty coral and abundant algae [e.g., [409]], rather than those of the Red Sea and the Indo-Pacific.

Seawater acidification may also be a threat to Mediterranean marine biodiversity [410]. The most obvious consequence of the increased concentration of CO_2_ in seawater is a reduced rate of biogenic calcification in marine organisms [411], [412]. This could affect both planktonic and benthic communities. Calcifying phytoplankton (coccolithophores) play a significant role in the primary productivity of the oligotrophic Mediterranean Sea, whereas many benthic habitats are engineered by sessile organisms that lay down carbonate crusts. Calcareous red algae are the builders of coralligenous reefs, one of the most important Mediterranean ecosystems, and seawater acidification will probably impair their role [413]. However, noncalcifying photosynthetic plants, such as frondose algae and seagrasses, may take advantage of a greater availability of CO_2_. But large, erect species of brown algae as well as Mediterranean seagrass are now in decline because of the environmental degradation, induced primarily by human activities [336], [414].

### The unknowns and limitations

The study of Mediterranean marine diversity over many years has produced a significant amount of information. Yet this information remains incomplete with the discovery and description of new species, especially of smaller, less conspicuous and cryptic biota (Table 1 and File S2). The biodiversity in the Mediterranean Sea may be in fact much higher than is currently known.

We do not have credible measures of microbial richness, but development of new technologies will allow us to decide whether this is knowable or not. The description of microbial diversity is probably better approached through the continued study at selected sites, such as the Microbial Observatories, for which data exist on both identification methodologies and the functioning of the ecosystem. Current Mediterranean observatories are at Blanes Bay, Gulf of Naples, Villefranche's Point B, Dyfamed station, and the MOLA and SOLA stations in Banyuls. Sites in the southern and eastern Mediterranean are still to be added.

Further exploration and taxonomic work on seaweeds and seagrasses is needed in all the African countries (mainly in Libya and Egypt), the Levantine Sea (Israel, Lebanon, Cyprus, Syria), and the Aegean Sea (Greece and Turkey). Phycological surveys are also required in Croatia, because several species (and even genera) described from the Adriatic have never been found again and require taxonomic reevaluation. We do not expect a significant increase in the rate of description of new species, but the description of new macroalgal species continues [e.g., [415],[416]]. A large number of species are poorly known, and our checklist includes several *taxa inquirenda* (see File S2). Accurate morphological studies, and new molecular tools, are required to decipher the taxonomy of several genera, including *Ectocarpus*, *Cystoseira*, *Acrochaetium*, *Polysiphonia*, and *Ulva*.

**Figure 13:**
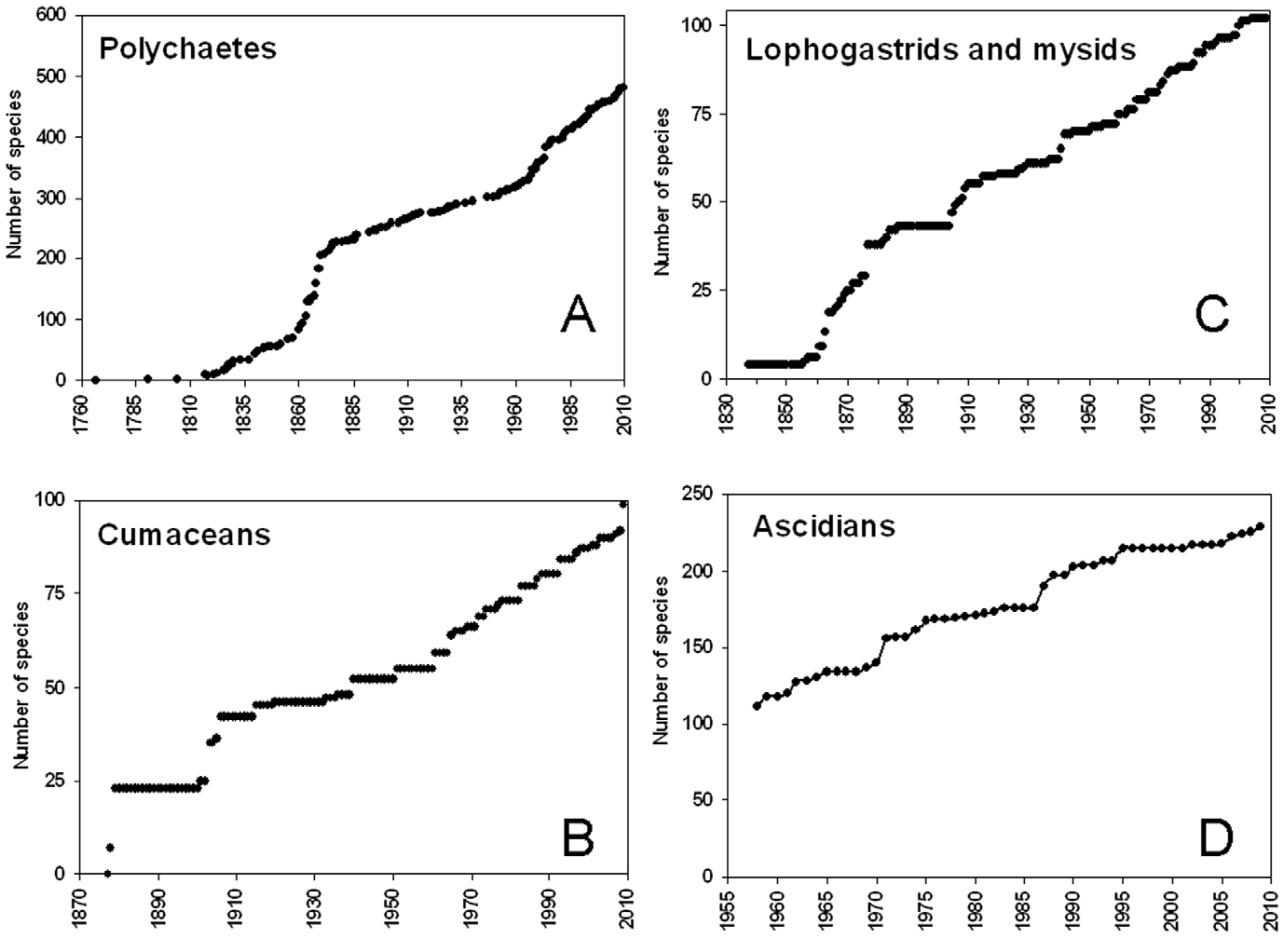
Cumulative numbers of species discovered (described or first recorded) over time in the Mediterranean Sea. (A) polychaetes, (B) cumaceans, (C) lophogastrids and mysids, and (D) ascidians (File S2).

A similar situation exists for the invertebrates (see File S2). Most of the small fauna of the Mediterranean are typical of current scientific knowledge: in one of the best-known geographic areas of the world, there are many regions and habitats that remain insufficiently studied, and several taxonomic groups in deep-sea areas and portions of the southern region are still poorly known. The description of new species is still a high priority. As illustrative examples, the accumulation curves for cumaceans, mysids, polychaetes, and ascidians discovered (described or first recorded) (Figure 13) show that no asymptote has been reached, and there has been no slowing in the rate of discovery for less conspicuous species in the Mediterranean, as it is observed when analyzing accumulation curves in other parts of the world [76].

The shortage of taxonomists for many groups is a particularly serious problem worldwide, and it also applies to the Mediterranean Sea. Several of the main invertebrate specialists have retired or are close to retirement and few are being replaced. Many samples are not being properly identified, which leads to a corresponding underestimation of biodiversity [417], [418]. The current spread of invasive species requires serious taxonomic attention. Many, if not most, taxonomic groups are subject to anthropogenic threats in one way or another, and researchers must work against time to avoid losing valuable biological information. Undescribed invertebrate species may become extinct before we even know of their existence [419], [420]. In addition, and paradoxically, some of the commonest and most accessible ecosystems such as beaches, among other habitats in the Mediterranean, have been poorly studied [421], [422]–[424].

**Figure 14:**
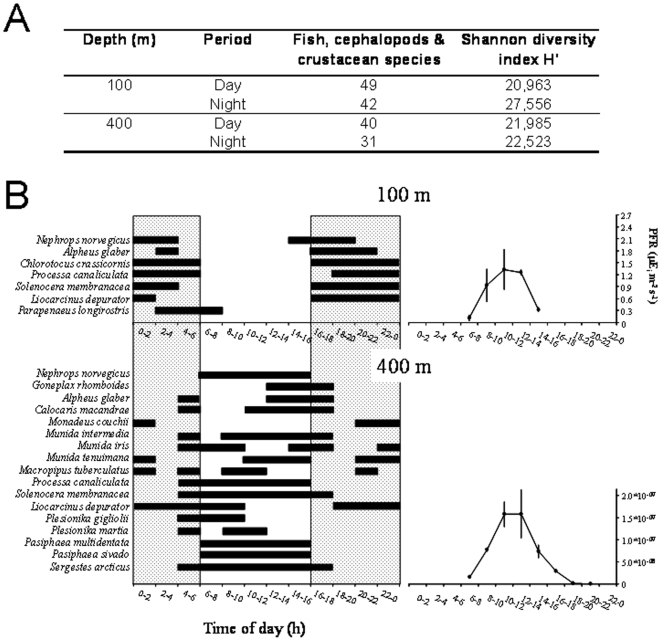
Diel difference in biodiversity estimates obtained with trawling in the Mediterranean Sea. Reported diel differences in estimated biodiversity are obtained by two trawl hauls performed at the autumnal equinox at midday and midnight, in the same sampling location of the western Mediterranean shelf (100 m) and slope (400 m), during October 1999 (NERIT survey). (A) Number of fish, crustaceans, and cephalopod species, and Shannon diversity index (H'), and (B) Waveform analysis of four-day time series of data for catches (left) and light intensity variations as photon fluency rate (PFR; right) for representative decapods. Black rectangles depict the temporal limits of significant increases in catches. Shaded gray rectangles indicate the night duration [adapted from 425].

Sampling biases are another source of uncertainty in the estimation of marine biodiversity. In particular, the three-dimensional character of marine ecosystems requires much more study at depths where light penetration is perceived as important but is poorly understood. Light intensity decreases with increasing depth and species perform extensive migrations within the water column or along the seabed. Endobenthic species display rhythms of emergence, including burying or burrowing within the substrate and sheltering in natural holes [425]. Marine species react to light intensity cycles, which may include movements in and out of our sampling windows [426]. Information gathered without attention to such rhythmicity will affect perceived population distribution, biomass, and estimated biodiversity [425]. These issues have been integral to land ecology since the early twentieth century [427] but have been rarely considered in the marine environment. In the Mediterranean, Sardà et al. [428] considered this problem during day-night sampling at and below the end of the twilight zone (1,000 m depth) and observed day-night fluctuations in their catches. Midday and midnight trawl catches at different depths during October showed great differences in fish, cephalopod, and crustacean species composition and relative abundance in the deeper areas (see Figure 14a). Waveform analysis of crustacean catches showed behavioral rhythms that affected presence or absence from catches made at different times during a 24-hour cycle (Figure 14b). Because trawl surveying is one of the commonest methods of sampling in marine waters [429], and is one of the most used in the Mediterranean Sea, future biodiversity studies should correct for the practice of sampling only during daytime. In addition, observations of important diel variation in the fauna associated with seagrasses include a notable increase of species richness and abundance in nighttime samples [430], [431]. This issue brings together the problem of biodiversity and climate change due to expected changes in species migrations and rhythmicity.

While Mediterranean vertebrate species are better known than the invertebrates, our understanding is still incomplete and often outdated. The FNAM atlas [70], which contains data collected and edited during the 1980s and 1990s, is based on regional data and expert knowledge and is the only record of geographic ranges for all Mediterranean fish species. Several areas of the southern Mediterranean have never been surveyed scientifically. Long-term monitoring programs are absent or unavailable for many countries. Since vertebrate species may be useful indicators of changes in ocean food webs [432], a major challenge that remains is to achieve time-series sampling of species diversity, abundance, and habitat data. These time series should have large spatial and temporal scales to develop useful indicators of changes in Mediterranean marine ecosystems and provide measures of ecological connections and ecosystem services.

A clearer identification of hot spot areas will require the inclusion of new data on macroalgae and seagrasses, invertebrates, and seabirds. Most of the Mediterranean seabird species (with the exception of some large gulls) are protected by European laws because of their small or declining populations or the small number of breeding sites. Nine species are included in Annex II of the EU list of endangered or threatened species. The Balearic shearwater is critically endangered [172], and the monitored colonies of Cory's and Mediterranean shearwaters are slowly declining [433]. Although information is incomplete for macroalgae and invertebrates [293], [434], a total of 11 species of macroalgae, 3 of flowering plants, 9 of sponges, 3 of cnidarians, 17 of mollusks, 2 of crustacean decapods, and 3 of echinoderms are now listed as endangered or threatened in the Annex II of the Barcelona Convention for the Protection of the Marine Environment and the Coastal Region of the Mediterranean (1995). A recent proposal (2009) for amendments in the annex II increased to four the number of flowering plants and to 16 plus all the species of the genus *Cystoseira* (with the exception of *C. compressa*) the number of endangered species of macroalgae.

### Conclusions

The Mediterranean Sea is a region of high biodiversity that ranks among the best known in the world, although much work remains to be done. The description of new species, especially of invertebrates and protists, undergoes upward revision, and new discoveries continually modify previous estimates. Increased efforts are required in taxonomy and sampling of poorly known ecosystems and on long-term monitoring programs of species and habitats. The invasion of alien species will continue to change the biodiversity of the Mediterranean Sea and requires continuous monitoring.

The first attempt to integrate the spatial data and temporal trends presented here enables one to visualize macroecological patterns at the Mediterranean scale. These results depict a region of high diversity and heterogeneity, but they also evidence the need for further study of geographical areas that are largely unexplored, mainly the African coasts and certain zones of the southeastern basin and the deep sea.

Our study illustrates that the Mediterranean is a complex region where ecological and human influences meet and strongly interact, posing a large and growing potential impact to marine biodiversity. Although much is known about individual threats, knowledge is very limited about how multiple impacts will interact. Therefore, there is the need to develop comprehensive analysis of conservation and management initiatives to preserve Mediterranean biodiversity. Owing to the Mediterranean physically, ecologically, and socioeconomically steep gradients, this region may be seen as a model of the world's oceans and a suitable laboratory to study marine ecosystems and decipher future trends.

In addition to further sampling and taxonomic efforts, much of what remains to be done requires free distribution of publicly available data from national and regional research initiatives. This will facilitate database updates and enable scientific discussion. Marine surveys are not always accessible at the regional level and, when available, data coverage is often incomplete. Regional initiatives (such as MedObis) provide promising platforms for the integration of efforts devoted to marine biodiversity within the Mediterranean region, but they must be kept up to date. Individual and collaborative research efforts must continue to advance our knowledge of marine biodiversity in the Mediterranean Sea and narrow down the unknowns.

## Supporting Information

File S1 Abstract translations
(0.08 MB DOC)

File S2 Supplementary material
(12.38 MB DOC)
