# Supplementary material for: The Biodiversity of the Mediterranean Sea: Estimates, Patterns, and Threats
Source: PLoS One. 2010 Aug 2;5(8):e11842. doi: 10.1371/journal.pone.0011842 (PMC2914016; doi:10.1371/journal.pone.0011842)
Supplement: File S1 — Abstract translations (0.08 MB DOC) [file pone.0011842.s001.doc]

**Supporting information 1^st^ file: abstracts translations**

# The Biodiversity of the Mediterranean Sea: Estimates, Patterns, and Threats

Marta Coll^1,2^, Chiara Piroddi^3^, Jeroen Steenbeek^3^, Kristin Kaschner^4^, Frida Ben Rais Lasram^5,6^, Jacopo Aguzzi^1^, Enric Ballesteros^7^, Carlo Nike Bianchi^8^, Jordi Corbera^9^, Thanos Dailianis^10,11^, Roberto Danovaro^12^, Marta Estrada^1^, Carlo Froglia^13^, Bella S. Galil^14^, Josep M. Gasol^1^, Ruthy Gertwagen^15^, João Gil^7^, François Guilhaumon^5^, Kathleen Kesner-Reyes^16^, Miltiadis-Spyridon Kitsos^10^, Athanasios Koukouras^10^, Nikolaos Lampadariou^17^, Elijah Laxamana^16^, Carlos M. López-Fé de la Cuadra^18^, Heike K. Lotze^2^, Daniel Martin^7^, David Mouillot^5^, Daniel Oro^19^, Saša Raicevich^20^, Josephine Rius-Barile^16^, Jose Ignacio Saiz-Salinas^21^, Carles San Vicente^22^, Samuel Somot^23^, José Templado^24^, Xavier Turon^7^, Dimitris Vafidis ^25^, Roger Villanueva^1^, and Eleni Voultsiadou^10^

1 - Institut de Ciències del Mar, Scientific Spanish Council (ICM-CSIC), Barcelona, Spain;

2 - Biology Department, Dalhousie University, Halifax, Canada;

3 - Fisheries Center - Aquatic Ecosystems Research Laboratory, University of British Columbia, Vancouver, Canada;

4 - Evolutionary Biology & Ecology Lab, Albert-Ludwigs-University, Freiburg, Germany;

5 - Laboratoire Ecosystèmes Lagunaires UMR 5119, Université Montpellier 2, Montpellier Cedex 5, France;

6- Laboratoire Ecosystèmes & Ressources Aquatiques UR03AGRO1, Institut National Agronomique de Tunisie, Tunis, Tunisia;

7- Centre d’Estudis Avançats de Blanes, Scientific Spanish Council (CEAB-CSIC), Blanes, Spain;

8- Dipartimento per lo studio del Territorio e delle sue Risorse, Università di Genova, Genova, Italy;

9- Carrer Gran, Argentona, Spain;

10 - Department of Zoology, Aristoteleio University of Thessaloniki, Thessaloniki, Greece;

11 - Hellenic Centre for Marine Research, Institute of Marine Biology and Genetics, Heraclion, Greece;

12 - Dipartimento Scienze del Mare, Polytechnic University of Marche, Ancona, Italy;

13 - Istituto di Scienze Marine, Consiglio Nazionale dell Ricerche, Ancona, Italy;

14 - National Institute of Oceanography, Israel Oceanographic & Limnological Research, Haifa, Israel;

15 - Haifa University & Oranim Academic College, Haifa, Israel;

16 - The WorldFish Center, Philippine Office, Los Baños, Philippines;

17 - Hellenic Centre for Marine Research, Institute of Oceanography, Heraklion, Greece;

18 - Laboratorio de Biología Marina - Departamento de Fisiología y Zoología, Universidad de Sevilla, Sevilla, Spain;

19 - Mediterranean Institute for Advanced Studies, Scientific Spanish Council (IMEDEA-CSIC), Esporles, Spain;

20 - Istituto Superiore per la Ricerca e la Protezione Ambientale, Chioggia, Italy;

21 - Zoology Department, University of the Basque Country, Bilbao, Spain;

22 - Carrer Nou, Creixell, Spain;

23 - Météo-France, Centre National de Recherches Météorologiques, Toulouse Cedex, France;

24 - Museo Nacional de Ciencias Naturales, Scientific Spanish Council (MNCN-CSIC), Madrid, Spain;

25 - Department of Ichthyology & Aquatic Environment, University of Thessaly, Nea Ionia, Greece.

**Address for correspondence:** [**mcoll@icm.csic.es,**](mailto:r.danovaro@univpm.it) [**martacoll@dal.ca**](mailto:martacoll@dal.ca)

**Abstrac translated to Arabic:**

**التنوع البيولوجي في البحر الأبيض المتوسط : التقدير والأنماط والتهديدات**

**الملخص**

يعتبر البحر الأبيض المتوسط نقطة ساخنة للتنوع البيولوجي البحري. قمنا في هذه الدراسة بدمج تحليل مستفيض للأدب مع معلومات الخبراء قصد تحديث تقديرات الأصناف الرئيسية في ذلك النظام الإيكولوجي البحري كما قمنا بمراجعة وتحديث قوائم الأنواع المختلفة. قمنا كذلك بتقييم الأنماط الزمانية والمكانية للتنوع البيولوجي وتحديد التغيرات الكبرى والتهديدات.

أحصينا ما يقرب من 000 17 كائنة بحرية في البحر الأبيض المتوسط. أظهرت الأنماط المكانية تواجد انخفاض تدريجي عام في التنوع البيولوجي من الشمال الغربي في اتجاه الجنوب حسب الإنتاج مع بعض الاستثناءات والاحتياطات اللازمة نظرا لوجود نقائص في معرفة الكائنات الحية على طول السواحل الجنوبية والشرقية.

بصفة عامّة يكون التنوع البيولوجي أعلى في المناطق الساحلية والجرف القاري ويتناقص مع العمق. أظهرت الاتجاهات الزمنية أن الإفراط في استغلال وخسارة الموائل هما السببان الرئيسيان للتغيرات التاريخية في التنوع البيولوجي. حاليا ، يمثل ضياع وتدهور الموائل، يليها الاستغلال المفرط والتلوث وتغير المناخ ، والتخثث وانتشار الأنواع الغريبة التهديدات الرئيسية التي تؤثر على أكبر عدد من المجموعات التصنيفية . كل هذه التأثيرات من المرجح أن تزداد في المستقبل ، خاصة تغير المناخ وتدهور الموائل.

تحديد النقاط الساخنة يكشف الأهمية الإيكولوجية لمعظم جروف غرب البحر الأبيض المتوسط خاصة مناطق مضيق جبل طارق ، وبحر البوران والساحل الأفريقي وشمال البحر الأدرياتيكي وبحر ايجه التي تتميز بتواجد كثيف للكائنات المهددة بالانقراض. أما الحوض الشرقي فهو مهدد بانتشار الكائنات الغريبة.

تقدير أنماط التنوع البيولوجي البحري لا يزال ناقصا بسبب بعض الأنواع التي لم توصف بعد والتي ستضاف في المستقبل. كما يوجد نقص في تقديرات التنوع الميكروبي وكائنات المناطق العميقة والمناطق الشرقية والجنوبية التي لا تزال غير معروفة. إن غزوات الأنواع الغريبة هي من العوامل الحاسمة التي سوف تستمر والتي ستحدث تغييرا رئيسيا للتنوع البيولوجي في الحوض الشرقي للبحر الأبيض المتوسط.

**Abstrac translated to Catalan:**

**La biodiversidat de la mar Mediterrània: estimacions, patrons i amenaces**

**Resum**

La mar Mediterrània és un “punt calent” de diversitat marina. En aquest treball combinem una extensa anàlisi de la literatura científica amb opinions d’experts per tal de posar al dia les estimacions publicades de la diversitat dels principals tàxons existents en aquest ecosistema marí, i per a revisar i actualitzar les diverses llistes d’espècies disponibles. També s’avaluen els patrons espacio-temporals globals de la diversitat específica i s’identifiquen els principals canvis i amenaces que afecten la diversitat de la Mediterrània. Hem quantificat aproximadament 17.000 espècies que habiten la Mediterrània i hem constatat una disminució general de la biodiversitat des de les regions noroccidentals cap a les sud-orientals, de forma paral·lela al gradient de producció primària, però amb algunes excepcions. Cal interpretar aquesta observació amb precaució degut a llacunes en el coneixement de la biota a les vores sud i est. La diversitat és generalment més alta a les àrees costaneres i a les plataformes continentals, i disminueix amb la profunditat. Les tendències temporals indiquen que la sobre-explotació i la pèrdua d’hàbitat han estat els principals factors antropogènics que han generat canvis històrics de biodiversitat. Actualment la pèrdua i degradació d’hàbitat, seguides per l’explotació, la contaminació, el canvi climàtic, l’eutrofització i l’establiment d’espècies invasores són les amenaces més importants i afecten un nombre elevat de grups taxonòmics. Es preveu que tots els impactes augmentin en el futur, especialment el canvi climàtic i la degradació d’hàbitat. La identificació de "punts calents" subratlla la importància ecològica de la major part de les plataformes de la Mediterrània occidental i, en particular, de l’estret de Gibraltar amb el mar d'Alboran i les costes africanes adjacents, conjuntament amb el mar Egeu i el mar Adriàtic, que mostren altes concentracions d’espècies en perill, amenaçades o vulnerables. La biodiversitat de la conca Llevantina, greument afectada per la invasió d’espècies, es troba també amenaçada. De tota manera, les estimacions presents de la riquesa i els patrons de la diversitat marina són incompletes i cal esperar que en el futur s’afegiran noves espècies encara no descrites. La diversitat microbiana està substancialment infravalorada i les zones marines profundes i parts de les regions meridionals i orientals són encara molt poc conegudes. La invasió d’espècies alienes és un factor crucial que continuarà canviant la biodiversitat de la Mediterrània, sobretot a la conca oriental.

**Abstrac translated to Dutch:**

**De biodiversiteit van de Middellandse Zee: Schattingen, patronen en bedreigingen**

**Samenvatting**

De Middellandse Zee is een hotspot voor mariene biodiversiteit. In dit paper combineren we een uitgebreide literatuurstudie met meningen van experts teneinde huidige schatting van prominente taxa in dit ecosysteem te herzien, en verschillende soortenlijsten bij te werken. We beschouwen ruimtelijke trends in soortenrijkdommen, en identificeren grote veranderingen en bedreigingen. Wij kwantificeerden ongeveer 17.000 mariene soorten die voorkomen in de Middellandse Zee. Ruimtelijke trends toonde een algemene daling in biodiversiteit van de noordwestelijke naar zuidoostelijke regio's, wat grotendeels overeen komt met een verloop van productie, maar met inachtname van lacunes in feitelijke kennis van flora en fauna langs de zuidelijke en oostelijke kusten van de Middellandse Zee. Biodiversiteit is in het algemeen hoger in de kustgebieden en het continentaal plat, en neemt af met diepte. Van oudsherre zijn overbevissing en habitatverlies de voornaamste menselijke factoren geweest die leidden tot veranderingen in biodiversiteit. Op dit moment zijn verlies en afbraak van habitat, gevolgd door overbevissing, vervuiling, klimaatverandering, eutrofiëring en de vestiging van uitheemse soorten de belangrijkste bedreigingen die het grootste aantal taxonomische groepen beinvloedden. Van al deze effecten, maar met name klimaatverandering en aantasting van habitat, is voorspeld dat zij in de toekomst in belang zullen toenemen. De identificatie van "hot spots" benadrukt het ecologische belang van het merendeel van de plateaus in de westelijke Middellandse Zee: de Straat van Gibraltar en de aangrenzende Alboran Zee en de Afrikaanse kust, de noordelijke Adriatische Zee en de Egeïsche Zee, waar hoge concentraties van ernstig bedreigde, bedreigde of kwetsbare soorten zijn aangetroffen. Het Levantijnse Basin, sterk beïnvloed door de invasie van uitheemse soorten, wordt ook bedreigd. Toch zijn onze schatting en patronen van mariene diversiteit onvolledig aangezien vele nog onbeschreven soorten in de nabije toekomst zullen worden toegevoegd. Schattingen van diversiteit voor microben zijn wel degelijk ondervertegenwoordigd, en de diepzee en delen van de zuidelijke en oostelijke regio zijn nog steeds slecht bekend. De invasie van uitheemse soorten is een cruciale factor met blijvende invloed op de biodiversiteit van de Middellandse Zee, met name in haar oostelijk bekken.

**Abstrac translated to French:**

**Biodiversité de la mer Méditerranée: estimations, patrons et menaces**

**Résumé**

La mer Méditerranée est un *hot spot* de biodiversité marine. Dans cette étude, nous combinons une analyse extensive de la littérature avec une connaissance d’expert afin de mettre à jour les estimations publiques disponibles des principaux taxons dans cet écosystème marin, et afin de réviser et de mettre à jour diverses listes d’espèces. De même, nous évaluons les patrons spatiaux et temporaux de la diversité spécifique et nous identifions les principaux changements et menaces. Nous avons dénombré approximativement 17 000 espèces marines en mer Méditerranée. Les patrons spatiaux ont montré une diminution générale de la biodiversité du nord ouest au sud est suivant un gradient de production avec toutefois quelques exceptions et des précautions dues aux failles de notre connaissance des biotes le long des rives sud et est. La biodiversité est généralement plus élevée au niveau des zones côtières et du plateau continental, et elle décroît avec la profondeur. Les tendances temporelles ont montré que la sur-exploitation et la perte des habitats sont les principaux drivers humains des changements historiques de la biodiversité. Actuellement, la perte des habitats et leur dégradation, suivies par la sur-exploitation, la pollution, les changements climatiques, l’eutrophisation et l’établissement des espèces exotiques sont les principales menaces qui ont un impact sur le plus grand nombre de groupes taxonomiques. Tous ces impacts sont susceptibles d’augmenter dans le futur, particulièrement les changements climatiques et la dégradation des habitats. L’identification des *hot spots* révèle l’importance écologique de la plupart des plateaux ouest méditerranéens et particulièrement le détroit de Gibraltar, la mer d’Alboran, les côtes africaines, le nord de l’Adriatique et la mer Egée qui montrent une forte concentration d’espèces en danger, menacées et vulnérables. Le bassin Levantin, sévèrement touché par les invasions d’espèces, est aussi menacé. Cependant, nos estimations et les patrons de diversité marine demeurent incomplets puisque certaines espèces non encore décrites s’y ajouteront dans le futur. Les estimations de la diversité microbienne sont substantiellement sous représentées et les zones profondes ainsi que les régions est et sud dont encore peu connues. Les invasions d’espèces exotiques sont un facteur crucial qui continuera de modifier la biodiversité méditerranéenne principalement dans le bassin est.

**Abstrac translated to German:**

**Die Artenvielfalt des Mittelmeers: Artenanzahl,-verteilung und Gefährdung/Bedrohung**

**Zusammenfassung**

Das Mittelmeer ist ein bekannter Hotspot für marine Biodiversität. Basierend auf einer umfassenden Literaturrecherche und unter Berücksichtigung von Expertenwissen stellen wir hier neu berechnete Schätzungen über die Artenanzahl verschiedener Taxa, sowie einige aktualisierte Artenlisten vor. Des weiteren haben wir die allgemeinen zeitlichen und räumlichen Verteilungsmuster der Biodiversität im Mittelmeer untersucht und hier dargestellt, sowie die Hauptursachen für mögliche vergangene und zukünftige Veränderungen identifiziert. Nach unseren Ergebnissen kommen schätzungsweise 17,000 marine Arten im Mittelmeer vor. In Hinblick auf Verteilungsmuster zeigen unsere zusammengetragenen Ergebnisse einen klaren abnehmenden Trend, der – dem Hauptprimärproduktionsgradienten folgendend - von nordwestlicher in südöstlicher Richung verläuft. Im allgemeinen ist die Artenvielfalt im Küstenbereich und entlang des Kontinentalshelfs grösser und nimmt mit zunehmender Tiefe ab. Überfischung und Habitatzerstörung waren bisher die hauptsächlichen anthropogenen Ursachen für Veränderungen und Abnahme der Artenvielfalt. Heutzutage spielen neben Habitatverlust, der die Hauptbedrohung mit den größten Auswirkungen auf die meisten Artengruppen darstellt, auch Fischerei, Umweltverschmutzung, Klimawandel, Überdüngung und das Einschleppen invasiver Arten eine Rolle. Nach unseren Vorhersagen wird die Auswirkung aller hier aufgeführten Faktoren in Zukunft noch zunehmen, dies gilt vor allem für Klimawandel und Habitatverlust. Unsere Untersuchungen zeigten eine besondere Konzentration bedrohter Arten entlang der westlichen Shelfgebiete, wobei hier die Strasse von Gibraltar und die daneben liegenden Gebiete des Alboranischen Meer sowie der afrikanische Küstenstreifen und auch die Adria und das Ägäische Meer besonders hervorstechen. Das Levantinische Becken, in welchem die Anzahl invasiver Arten besonders hoch ist, ist ebenfalls bedroht. Die hier gezeigten Daten hinsichtlich der Gesamtartenzahl und Verbreitungsmuster können nur vorläufige Schätzungen darstellen, da vermutlich noch weitere bisher unbekannte Arten dazu kommen werden. Besonders lückenhaft ist unser Wissen über die Mikrobenvielfalt und die mediterranen Tiefseegebiete, sowie über einige Gebiete in den südöstlichen Bereichen des Mittelmeers. Das Einschleppen invasiver Arten ist ein kritischer Faktor, der auch weiterhin grossen Einfluss auf die Artenvielfalt des Mittelmeers haben wird, besonders in den östlichen Becken.

**Abstrac translated to Greek:**

**Η βιοποικιλότητα στη Μεσόγειο θάλασσα: εκτιμήσεις, πρότυπα και απειλές**

**Περίληψη**

Η Μεσόγειος Θάλασσα αποτελεί πυρήνα θαλάσσιας βιοποικιλότητας. Στην εργασία αυτή γίνεται εκτεταμένη ανάλυση της βιβλιογραφίας σε συνδυασμό με τις απόψεις ειδικών με σκοπό την αναθεώρηση των διαθέσιμων εκτιμήσεων μεγέθους της ποικιλότητας των κύριων ομάδων οργανισμών στο θαλάσσιο αυτό οικοσύστημα, αλλά και την παρουσίαση ενημερωμένων καταλόγων ειδών. Γίνεται ακόμη εκτίμηση γενικών χωρικών και χρονικών προτύπων της ποικιλότητας των ειδών και προσδιορίζονται σημαντικές μεταβολές και απειλές. Καταγράφηκε η παρουσία 17.000 θαλάσσιων ειδών στη Μεσόγειο κατά προσέγγιση. Τα πρότυπα χωροκατανομής έδειξαν μια γενικευμένη μείωση της βιοποικιλότητας από τις βορειοδυτικές προς τις νοτιοανατολικές περιοχές, η οποία ακολουθεί μια αντίστοιχη διαβάθμιση της πρωτογενούς παραγωγής. Το πρότυπο αυτό γίνεται δεκτό με ορισμένες εξαιρέσεις κι επιφυλάξεις λόγω ελλιπούς γνώσης για το βιόκοσμο των ανατολικότερων και νοτιότερων περιοχών. Η βιοποικιλότητα είναι γενικά μεγαλύτερη στις παράκτιες περιοχές και την υφαλοκρηπίδα, ενώ μειώνεται αυξανομένου του βάθους. Οι τάσεις μέσα στο χρόνο δείχνουν ότι η υπεραλίευση και η καταστροφή των ενδιαιτημάτων αποτελούν τα σημαντικότερα ανθρωπογενή αίτια των ιστορικών αλλαγών στη βιοποικιλότητα. Σήμερα, η απώλεια και υποβάθμιση των ενδιαιτημάτων και ακολούθως η εκμετάλλευση, η ρύπανση, η κλιματική αλλαγή, ο ευτροφισμός και η εγκατάσταση αλλόχθονων ειδών αποτελούν τις σημαντικότερες απειλές και επηρεάζουν τις περισσότερες ταξινομικές ομάδες οργανισμών. Όλες αυτές οι επιδράσεις προβλέπεται ότι θα εμφανιστούν περισσότερο έντονες στο μέλλον, κυρίως όσον αφορά την κλιματική αλλαγή και την υποβάθμιση των ενδιαιτημάτων. Ιδιαίτερη οικολογική σημασία ως πυρήνες βιοποικιλότητας μέσα στη Μεσόγειο φαίνεται να έχουν η υφαλοκρηπίδα της Δυτικής λεκάνης στη μεγαλύτερη έκτασή της, με έμφαση στο στενό του Γιβραλτάρ, τη γειτονική θάλασσα του Αλμποράν και τις αφρικανικές ακτές, η βόρεια Αδριατική θάλασσα και το Αιγαίο πέλαγος. Οι περιοχές αυτές παρουσιάζουν μεγάλες συγκεντρώσεις ειδών που κινδυνεύουν, απειλούνται ή είναι τρωτά. Η Λεβαντίνη, που έχει δεχθεί έντονη εισβολή αλλόχθονων ειδών, βρίσκεται επίσης σε κίνδυνο. Πάντως, η θαλάσσια βιοποικιλότητα και τα πρότυπά της στη Μεσόγειο είναι ακόμη ελλιπώς γνωστά και αναμένεται ότι πρόκειται να γίνουν περιγραφές και προσθήκες νέων ειδών στο μέλλον. Η γνώση για την ποικιλότητα των μικροοργανισμών, καθώς και το βιόκοσμο των μεγάλων βαθών και των νότιων και ανατολικότερων περιοχών αναμένεται να αυξηθεί. Τέλος, η εισβολή αλλόχθονων ειδών αποτελεί αποφασιστικό παράγοντα ο οποίος θα συνεχίσει να μεταβάλλει τη βιοποικιλότητα της Μεσογείου, ιδιαίτερα στην ανατολική λεκάνη.

**Abstrac translated to Hebrew:**

המגוון הביולוגי של הים התיכון: אמדן, מגמות, ואיומים

תקציר

הים התיכון הוא בעל מגוון ביולוגי רב. במאמר זה אנו משלבים בחינה מפורטת של הספרות עם דעות מומחים במטרה לעדכן את האומדנים המצויים של הקבוצות הטקסונומיות הראשיות במערכת הימית ולתקן מספר רשימות מינים. בנוסף אנו אומדים מגמות במרחב ובזמן של שונות מינים ומזהים שינויים חשובים ואיומים על המגוון. כ 17,000 מינים מצויים בים התיכון. דגמים מרחביים מצביעים על פחיתה במגוון מצפון-מערב הים לדרום-מזרחו, בעקבות דעיכה הדרגתית ביצור. אך יש להתריע שקביעה זו עשויה להשתנות אם יצומצמו הפערים במידע על הביוטה לאורך החופים הדרומיים והמזרחיים של הים.

המגוון הביולוגי גבוה יותר בדרך כלל בקרבת החופים ואדני היבשת, ופוחת בעומק. המגמות לאורך ציר הזמן מצביעות שניצול לא מבוקר ואובדן בתי גידול הם הסיבות העיקריות לשינויים מעשי אדם במגוון.

בהווה, השחתה והרס בתי גידול, ניצול משאבים, זיהום, שינוי אקלימי, העשרה אורגנית והתבססות מינים זרים הם האיומים העיקריים המשפיעים על המספר הגדול ביותר של קבוצות טקסונומיות. השפעות אלו צפויות להחמיר בעתיד, במיוחד שינוי אקלימי והרס בתי גידול. זיהוי אתרים בעלי מגוון מינים גבוה במיוחד מבליט את החשיבות האקולוגית של אדני היבשת במערב הים התיכון, במיוחד מיצר גיברלטר, ים אלבורן והחוף האפריקאי הסמוך; צפון הים האדריאטי והאגאי –אשר בהם ריכוז של מינים רגישים ובסכנת הכחדה. אגן הלבנט, המושפע באורח חמור מפלישות מינים, נמצא בסכנה גם הוא. האומדנים והדגמים של מגוון ימי אינם שלמים – אנו צופים שמינים נוספים יתוארו ויתווספו למניין בעתיד. אומדני מינים חד תאיים לוקים בחסר, ואזורים בים העמוק, ובדרום ומזרח הים התיכון ידועים אך מעט. פלישה של מינים זרים מהווה גורם מכריע אשר יוסיף וישנה את מגוון המינים, בעיקר באגן המזרחי.

**Abstrac translated to Italian:**

**La biodiversità del Mar Mediterraneo: stime, distribuzione e minacce**

**Riassunto**

Il Mar Mediterraneo è un *hotspot* di biodiversità marina. Questo studio integra un’ampia analisi della letteratura insieme a opinioni di esperti con l’intento di aggiornare pubblicamente le stime disponibili dei maggiori *taxa* di questo ecosistema marino, e di rivedere e aggiornare le liste delle diverse specie. La nostra ricerca ha valutato anche la distribuzione spaziale e temporale complessiva della diversità delle specie, identificandone i cambiamenti e le minacce più significative: nel Mediterraneo.sono state quantificate circa 17.000 specie marine. Le distribuzioni spaziali hanno mostrato una generale diminuzione della biodiversità dalle regioni nord-ovest a quelle sud-est, seguendo un gradiente di produzione, con qualche eccezione e cautela dovuta a mancanza di dati sul biota lungo le coste sudorientali. La biodiversità è generalmente più elevata nelle aree costiere e nelle scarpate continentali e diminuisce con la profondità. I trend temporali hanno mostrato come lo sfruttamento eccessivo delle risorse e la perdita degli habitat siano stati i fattori principali di origine umana alla base dei cambiamenti storici nella biodiversità. Attualmente, le minacce più significative e quelle che impattano il maggior numero di gruppi tassonomici sono la perdita di habitat e la sua degradazione, seguiti da sfruttamento, inquinamento, cambiamenti climatici, eutrofizzazione e introduzione di specie aliene. Si prevede che tutte queste minacce diventino sempre più importanti in futuro, specialmente il cambiamento climatico e la degradazione dell’habitat. L’identificazione di *hotspot* mette in evidenza l’importanza ecologica della maggior parte delle coste occidentali del Mediterraneo, in particolare dello stretto di Gibilterra e delle adiacenti coste del Mare di Alboran e dell’Africa, dell’Adriatico settentrionale e dell’Egeo, aree che mostrano elevate concentrazioni di specie in pericolo, minacciate o vulnerabili. Anche il bacino levantino, gravemente colpito dall’invasione di specie, è minacciato. Tuttavia, le nostre stime e la distribuzione della biodiversità marina sono ancora incomplete, essendoci specie non descritte che verranno aggiunte in futuro. Le stime sulla biodiversità dei microbi sono sostanzialmente ancora sottorappresentate, così come sono poco conosciute le aree marine profonde e alcune parti delle regioni meridionali e orientali. L’invasione di specie aliene è un fattore cruciale che continuerà a modificare la biodiversità mediterranea, soprattutto nel bacino orientale.

**Abstrac translated to Spanish:**

**La biodiversidad del mar Mediterráneo: estimaciones, patrones y amenazas**

**Resumen**

El mar Mediterráneo es un *hotspot* marino de biodiversidad. En este trabajo hemos combinado el análisis de datos bibliográficos con la opinión y el conocimiento de expertos para actualizar las estimaciones de la biodiversidad de especies marinas de los principales taxones en este ecosistema, al mismo tiempo que hemos revisado numerosos listados de especies. También hemos analizado los patrones espaciales y temporales de la diversidad, así como identificado las principales amenazas. Se han cuantificado un total aproximado de 17.000 especies marinas en el mar Mediterráneo. Los patrones espaciales muestran como esta diversidad decrece desde el noroeste al sudeste siguiendo un gradiente de producción teniendo en cuenta algunas excepciones y precauciones, ya que existen aun áreas con gran escasez de datos, sobretodo en las zonas del sur y del este. La biodiversidad marina es generalmente mayor en las zonas costeras y en las plataformas continentales y desciende con la profundidad. Los patrones temporales indican que la sobreexplotación y la degradación y la pérdida del hábitat han sido en el pasado las actividades humanas con mayor impacto sobre la biodiversidad marina. Actualmente, la degradación y la pérdida del hábitat, seguidas por la explotación, la polución, el cambio climático, la eutrofización y la introducción de especies foráneas, son las principales amenazas que afectan al mayor número de grupos taxonómicos. La importancia de estos impactos muy probablemente crecerá en un futuro, especialmente la de los asociados al cambio climático y a la degradación del hábitat. En este trabajo se identifican una serie de *hotspots* o zonas con una elevada concentración de especies de gran importancia ecológica, en la parte occidental del mar Mediterráneo, y en particular en el estrecho de Gibraltar, el mar de Alborán y la costa africana asociada, y también en el norte del mar Adriático y del mar Egeo. Estas regiones muestran una alta concentración de especies en peligro, amenazadas o vulnerables. El mar de Levante, impactado notablemente por la invasión de especies foráneas, también se encuentra amenazado. Sin embargo, nuestro conocimiento sobre el número total de especies marinas y los patrones de biodiversidad son aun incompletos, ya que existen numerosas especies aún sin describir. Las estimaciones relativas a la comunidad bacteriana están substancialmente infravaloradas, y gran parte del mar profundo y regiones del sur y del este del mar Mediterráneo se encuentran todavía prácticamente inexploradas. La invasión de especies foráneas es también un factor crucial que continuará influyendo en la biodiversidad marina del Mediterráneo, sobretodo en las zonas situadas en la cuenca este.
