# Supplementary material for: The Biodiversity of the Mediterranean Sea: Estimates, Patterns, and Threats
Source: PLoS One. 2010 Aug 2;5(8):e11842. doi: 10.1371/journal.pone.0011842 (PMC2914016; doi:10.1371/journal.pone.0011842)
Supplement: File S2 — Supplementary material (12.38 MB DOC) [file pone.0011842.s002.doc]

**Supporting information 2^nd^ file: supplementary material**

# The Biodiversity of the Mediterranean Sea: Estimates, Patterns, and Threats

Marta Coll^1,2^, Chiara Piroddi^3^, Jeroen Steenbeek^3^, Kristin Kaschner^4^, Frida Ben Rais Lasram^5,6^, Jacopo Aguzzi^1^, Enric Ballesteros^7^, Carlo Nike Bianchi^8^, Jordi Corbera^9^, Thanos Dailianis^10,11^, Roberto Danovaro^12^, Marta Estrada^1^, Carlo Froglia^13^, Bella S. Galil^14^, Josep M. Gasol^1^, Ruthy Gertwagen^15^, João Gil^7^, François Guilhaumon^5^, Kathleen Kesner-Reyes^16^, Miltiadis-Spyridon Kitsos^10^, Athanasios Koukouras^10^, Nikolaos Lampadariou^17^, Elijah Laxamana^16^, Carlos M. López-Fé de la Cuadra^18^, Heike K. Lotze^2^, Daniel Martin^7^, David Mouillot^5^, Daniel Oro^19^, Saša Raicevich^20^, Josephine Rius-Barile^16^, Jose Ignacio Saiz-Salinas^21^, Carles San Vicente^22^, Samuel Somot^23^, José Templado^24^, Xavier Turon^7^, Dimitris Vafidis ^25^, Roger Villanueva^1^, and Eleni Voultsiadou^10^

1 - Institut de Ciències del Mar, Scientific Spanish Council (ICM-CSIC), Barcelona, Spain;

2 - Biology Department, Dalhousie University, Halifax, Canada;

3 - Fisheries Center - Aquatic Ecosystems Research Laboratory, University of British Columbia, Vancouver, Canada;

4 - Evolutionary Biology & Ecology Lab, Albert-Ludwigs-University, Freiburg, Germany;

5 - Laboratoire Ecosystèmes Lagunaires UMR 5119, Université Montpellier 2, Montpellier Cedex 5, France;

6- Laboratoire Ecosystèmes & Ressources Aquatiques UR03AGRO1, Institut National Agronomique de Tunisie, Tunis, Tunisia;

7- Centre d’Estudis Avançats de Blanes, Scientific Spanish Council (CEAB-CSIC), Blanes, Spain;

8- Dipartimento per lo studio del Territorio e delle sue Risorse, Università di Genova, Genova, Italy;

9- Carrer Gran, Argentona, Spain;

10 - Department of Zoology, Aristoteleio University of Thessaloniki, Thessaloniki, Greece;

11 - Hellenic Centre for Marine Research, Institute of Marine Biology and Genetics, Heraclion, Greece;

12 - Dipartimento Scienze del Mare, Polytechnic University of Marche, Ancona, Italy;

13 - Istituto di Scienze Marine, Consiglio Nazionale dell Ricerche, Ancona, Italy;

14 - National Institute of Oceanography, Israel Oceanographic & Limnological Research, Haifa, Israel;

15 - Haifa University & Oranim Academic College, Haifa, Israel;

16 - The WorldFish Center, Philippine Office, Los Baños, Philippines;

17 - Hellenic Centre for Marine Research, Institute of Oceanography, Heraklion, Greece;

18 - Laboratorio de Biología Marina - Departamento de Fisiología y Zoología, Universidad de Sevilla, Sevilla, Spain;

19 - Mediterranean Institute for Advanced Studies, Scientific Spanish Council (IMEDEA-CSIC), Esporles, Spain;

20 - Istituto Superiore per la Ricerca e la Protezione Ambientale, Chioggia, Italy;

21 - Zoology Department, University of the Basque Country, Bilbao, Spain;

22 - Carrer Nou, Creixell, Spain;

23 - Météo-France, Centre National de Recherches Météorologiques, Toulouse Cedex, France;

24 - Museo Nacional de Ciencias Naturales, Scientific Spanish Council (MNCN-CSIC), Madrid, Spain;

25 - Department of Ichthyology & Aquatic Environment, University of Thessaly, Nea Ionia, Greece.

**Address for correspondence:** [**mcoll@icm.csic.es,**](mailto:r.danovaro@univpm.it) [**martacoll@dal.ca**](mailto:martacoll@dal.ca)

**Outline**

**Table S1.** Taxonomic classification of species known from the Mediterranean Sea.

**Table S2.** List of co-authors with expertise, specific contribution to the synthesis, and contact information.

**Text S1.** Taxonomic revision and methodological specifications.

**Mediterranean AquaMaps (by Kristin Kaschner, Kathy Kesner-Reyes, Josephine Barile & Elijah Laxamana)**

**Text S2.** Mediterranean AquaMaps specifications.

**Table S3.** Mediterranean native and endemic marine species reported by FishBase/SeaLifeBase by different taxa and current extent of coverage of AquaMaps.

**Table S4.** Proportion of checked or expert-reviewed AquaMaps for different taxa.

**Table S5.** Mediterranean species of special conservation concern covered by AquaMaps.

**Table S6.** Checklist of species included in AquaMaps of the Mediterranean Sea.

**Seaweeds and Seagrasses (by Enric Ballesteros)**

**Table S7.** Mediterranean biodiversity (species/infraspecific taxa, families, orders, classes) for the phyla Heterokontophyta, Rhodophyta, Chlorophyta and Magnoliophyta and all the macrophytobenthos. Total number of species/intraspecific taxa is also split into introduced, endemics and others.

**Table S8.** Percentage of introduced, endemics and other macrophytobenthic species/infraspecific taxa by phylum and totals.

**Table S9.** Checklist of the phylum Heterokontophyta and comments to the checklist.

**Table S10.** Checklist of the phylum Rhodophyta and comments to the checklist.

**Table S11.** Checklist of the phylum Chlorophyta and comments to the checklist.

**Table S12.** Checklist of the phylum Magnoliophyta and comments to the checklist.

**Sponges (by Eleni Voultsiadou & Thanos Dailianis)**

**Figure S1.** Mediterranean percentages of the world sponge families and genera for each demosponge order.

**Figure S2.** Distribution of the recorded demosponge species (outer circle) and genera (inner circle) richness in distinct zoogeographic areas of the Mediterranean.

**Anthozoans (by Dimitris Vafidis)**

**Table S13.** Checklist of the class Anthozoa (Phylum Cnidaria).

**Mollusks (by José Templado & Roger Villanueva)**

**Table S14.** Checklist of the phylum Mollusca.

**Table S15.**  Number of Mediterranean species of each of the eight mollusks classes.

**Polychaetes (by Daniel Martín & João Gil)**

**Table S16.** Checklist of the class Polychaeta (Phylum Annelida).

**Cumaceans (by Jordi Corbera)**

**Table S17.** Checklist of the order Cumacea (Phylum Arthropoda), with enumeration of references in each region of the Mediterranean Sea.

**Table S18.** Species number of cumaceans known in different Mediterranean regions.

**Mysidaceans (by Carles San Vicente)**

**Table S19.** Checklist of the orders Mysida and Lophogastrida (Phylum Arthropoda) known in the different Mediterranean regions considered.

**Table S20.** Species number of mysidaceans known in the world and in the Mediterranean Sea. Endemics and its percentage for each family are also indicated.

**Figure S3.** Number of mysidacean species recorded in each of the main biogeographical zones of the Mediterranean Sea.

**Decapods (by Carlo Froglia)**

**Table S21.** Checklist of the Mediterranean endemic species of the Order Decapoda (Phylum Arthropoda), known geographic distribution and bathymetric range.

**Bryozoans (Carlos Mª López-Fé de la Cuadra)**

**Text S3.** References for Mediterranean bryozoan species.

**Table S22.** Checklist of the phylum Bryozoa.

**Echinoderms (by Athanasios Koukouras & Miltiadis-Spyridon Kitsos)**

**Text S4.** References for Mediterranean Echinoderms diversity.

**Table S23.** Checklist of the phylum Echinodermata, and their distribution in the geographical areas of the Mediterranean with reference to their presence in the Atlantic and the Indo-Pacific Oceans.

**Figure S4.** Distribution of the known species of echinoderms in the main geographical areas of the Mediterranean, as real numbers (parentheses) and percentages of the total Mediterranean species.

**Figure S5.** Percentages of the four zoogeographical categories in the Mediterranean territorial areas and the Black Sea.

**Sipunculans (by José Ignacio Saiz-Salinas)**

**Table S24.** Checklist of the phylum Sipuncula. Asterisks indicate unpublished identifications from the author J.I. Saiz. *N. sp. cf. flagriferum* (43º02.83’N; 9º41.06’E; depth: 454 m). *P. turnerae* (41º07’N; 2º25’E; depth: 1100 m).

**Figure S6.** Cluster analysis of sipunculan species of the Mediterranean Sea by biogeographic sectors as proposed by Bianchi and Morri (2000).

**Meiobenthos (by Nikolaos Lampadariou)**

**Text S5.** References for Mediterranean Meiobenthos.

**Ascidians (by Xavier Turon)**

**Table S25.** Check-list of the class Ascidiacea (Subphylum Tunicata, Phylum Chordata).

**Fishes (by Frida Ben Rais Lasram)**

**Table S26.** Checklist of the class Chondrichthyes, Actinopterygii, Myxini and Hyperoartia (subphylum Vertebrata, phylum Chordata).

**Seabirds, marine mammals, and turtles (by Daniel Oro & Chiara Piroddi)**

**Table S27.** Checklist of the class Aves (subphylum Vertebrata, phylum Chordata).

**Table S28.** Checklist of the class Mammalia (subphylum Vertebrata, phylum Chordata).

**Table S29.** Checklist of the class Reptilia (subphylum Vertebrata, phylum Chordata).

**The diversity of the past and temporal patterns (by Heike Lotze)**

**Table S30.** Ecologically and economically important species of the Adriatic Sea for which long-term data were available and that were included in historical diversity trends.

**Table S31.** Timing of different cultural periods around the Adriatic Sea (after Haywood 1997; Lotze et al. 2006).

**Current threats to diversity**

**Table S32.** Ranking of current threats for various taxonomic groups in the Mediterranean Sea.

**Table S33.** Ranking of future threats to diversity (considering 10 years from now) for various taxonomic groups in the Mediterranean Sea.

## **Table S1. Taxonomic classification of species known from the Mediterranean Sea**

The total number of species described, as well as the number of endemic and introduced species are given. Here we also provide a list of several experts and taxonomic guides by taxa, although this is not an exhaustive list of experts by taxonomic group in the Mediterranean Sea.

|  |  |  | |  |  |  |  |  |  |  | |  |
| --- | --- | --- | --- | --- | --- | --- | --- | --- | --- | --- | --- | --- |
| **Division/Kingdom** | **Phylum or Class** | **Class or Order** | | **Order or Family** | **Described species** | **State of Knowledge** | **No. endemic species** | **No. undescribed species** | **No. introducespecies** | **Taxonomic experts (name)^1^** | | **Identification guides and references^2^** |
| **Animalia** |  |  | |  |  |  |  |  |  |  | |  |
|  | **Chordata** |  | |  | **954** |  |  |  | **131** |  | |  |
|  | Vertebrata |  | |  | 693 |  |  |  |  |  | |  |
|  |  | Mammalia | |  | 23 | 9 common, 14 visiting. Few data on trends | | |  | G. Bearzi, G. Notabartolo di Sciara, A. Aguilar, C. Piroddi | | [1,2,3,4, Table S28] |
|  |  | Aves | |  | 15 | Well known, trends less known | 3 |  |  | D. Oro, J.S. Aguilar, N. Baccetti, J.M. Arcos | | [5,6,7, Table S27] |
|  |  | Reptilia | |  | 5 | 3 common, 2 visiting. Few data on trends | | | | J.A. Camiñas, J. Tomás, J.A. Raga | | [8,9,10,11,12, Table S29 ] |
|  |  | Pisces* (Myxinida, Petromyzontida, Chondrichthyes,Actinopterygia) | | | 650 | Good resolution, except few rare species recorded sporadically | Approx. 80 |  | 116 (91*) *marine aliens only | F. Ben Rais Lasram, D. Lloris, L.J.V. Compagno, D. Golani, E. Massutí, J. Moranta, F. Serena, J.P. Quignard, J.A. Tomasini, I.K. Fergusson, S.P. Iglésias, E. Macpherson, M. Goren | | [13,14,15,16,17,18,19,20,21,22, Table S26] |
|  | Tunicata |  | |  | 229 |  |  |  |  |  | |  |
|  |  | Ascidiacea | |  | 229 |  | 80 |  | 15 | X. Turon, R. Brunetti, F. Monniot , A. Koukouras, F. Mastrototaro, A. Tursi, N. Shenkar, A.A. Ramos-Esplà | | [23,24,25,26,27,28, Table S25] |
|  |  | Thaliacea | |  | Unknown |  |  |  |  |  | |  |
|  |  | Appendicularia | |  | Unknown |  |  |  |  |  | |  |
|  | Cephalocordata | | |  | 1 |  |  |  |  |  | | [19] |
|  | Mesozoa |  | |  | 31 |  |  |  |  |  | | [19] |
|  |  | Dicyemida, Rhomobozoa, Monoblastozoa | | | 17 |  |  |  |  |  | |  |
|  |  | Orthonectida | |  | 14 |  |  |  |  |  | | [19] |
|  | **Echinodermata** | | |  | **154** | Lack of data in the south and the deep | 37 |  | 5 | A. Koukouras, M.-S. Kitsos, C. Palacin | | [29,30, Table S23] |
|  |  | | Crinoidea |  | 5 |  | 2 |  | 0 |  | |  |
|  |  | | Asteroidea |  | 33 |  | 5 |  | 1 |  | |  |
|  |  | | Ophiuroidea |  | 34 |  | 6 |  | 2 |  | |  |
|  |  | | Echinoidea |  | 28 |  | 5 |  | 1 |  | |  |
|  |  | | Holothuroidea |  | 54 |  | 19 |  | 1 |  | |  |
|  | **Hemichordata** | | |  | **5** |  |  |  |  |  | | [19] |
|  | **Xenoterubellida** | | |  | Unknown |  |  |  |  |  | |  |
|  | **Platyhelminthes** | | | Mainly benthic | **800-1000** |  | 57 |  |  | R.A. Patzner, R. Hofrichter, A. Schmidt-Rhaesa, A. Faubel, C. Noreña Janssen, M. Curini Galletti | | [19] |
|  | **Acanthocephala** | | | Mainly terrestrial | Unknown | Poor |  |  |  |  | | [19] |
|  | **Rotifera (Rotatoria)** | | |  | **59** |  |  |  |  |  | | [19] |
|  | **Nematomorpha** | | |  | **1** |  |  |  |  |  | | [19] |
|  | **Nematoda** |  | | Meiofauna | **>703** | Poor |  |  |  | N.Lampadariou, R. Danovaro, R. Sandulli | | [19, 31,32,33,34] |
|  | **Priapulida** |  | |  | **4 - 5** |  |  |  |  |  | | [19,35] |
|  | **Loricifera** |  | | Recently discovered | **2 - 10** |  |  |  |  |  | | [19,35] |
|  | **Kinorhyncha** |  | |  | **28** |  |  |  |  |  | | [19] |
|  | **Tardigrada** |  | |  | **>77** |  |  |  |  | M. Gallo d’Addabbo | | [36] |
|  | **Onychophora** | | |  | Unknown |  |  |  |  |  | | [19] |
|  | **Arthropoda** |  | |  | **3014** |  |  |  | **106** |  | | [19,37] |
|  | **Crustacea** |  | |  | 2239 |  |  |  |  |  | |  |
|  | Anostraca |  | |  | Unknown |  |  |  |  |  | |  |
|  | Phyllopoda |  | |  | 7 |  |  |  |  |  | | [19] |
|  | Copepoda |  | |  |  |  |  |  |  | G. Gorsky, M. Alcaraz, E. Saiz | |  |
|  |  | Harpacticoida | | Benthic, meiofauna | >254 |  |  |  |  | K. Sevastou, A. Eleftheriou | | [38,39,40,41,42] |
|  |  | Calanoida, Cyclopoida | | Pelagic | >150 |  |  |  |  |  | | [19] |
|  | Mystacocarida |  | |  | 1? |  |  |  |  |  | | [19] |
|  | Ostracoda |  | |  | 500 |  |  |  |  | N. Pugliese | |  |
|  | Ascothoracida |  | |  | 1 |  |  |  |  |  | |  |
|  | Cirripeda |  | |  | >40 |  |  |  |  | A. Koukouras, A. Matsa, G. Relini | | [43,44] |
|  | Malacostraca |  | |  |  |  |  |  |  |  | |  |
|  |  | Leptostraca | |  | 6 |  |  |  |  | J. Moreira | | [36,45] |
|  |  | Stomatopoda | |  | 11 |  |  |  | 2 | P. Abelló, C. Froglia, B.S. Galil | | [46,47,48]. |
|  |  | Bathynellacea | |  | 10? |  |  |  |  |  | | [19] |
|  |  | Euphausiacea | |  | 13 |  |  |  |  | M. Mavidis, L. Guglielmo | | [49] |
|  |  | Decapoda | |  | 383 |  | 40 |  | 74 (69*) *marine aliens | C. Froglia, A. Koukouras, J. E. Cartes, B. Companys, B.S. Galil, P. Noël, P. Dworschak, C. d’Udekem d’Acoz, T. Katagan | | [44,50,51,52,53, Table S21] |
|  |  | Thermosbaenacea | | | 5 |  |  |  |  |  | | [19] |
|  |  | Mysidacea | |  | 102 | African coasts and eastern sector less studied | 37 | 0.6% annual increase of new species | 2 | C.Sanvicente, C. Barberá Cebrián, J.E. Cartes, T. Munilla, P. Ariani, K. Wittman | | [54,55, Table S19] |
|  |  | Cumacea | | Water-sediment interface | 99 | Adriatic Sea or the Gulf of Gabes unknown | 32 | 0.5% annual increase of new species | 1 | J. Corbera, J.E. Cartes, J.C. Sorbe | | [56, Table S17] |
|  |  | Isopoda | |  | 165 |  |  |  |  |  | | [44] |
|  |  | Amphipoda | |  | 449 |  |  |  |  | D. Bellan-Santini, S. Ruffo, T. Krapp-Schickel | | [44,57,58,59] |
|  |  | Tanaidacea | |  | 43 |  |  |  |  |  | | [19] |
|  | **Arachnida** |  | | Only species related to marine environments | 13/450 |  |  |  |  |  | | [19,37] |
|  | **Pantopoda** |  | |  | >45 |  |  |  |  | T. Murilla, F. Krapp | | [19] |
|  | **Chilopoda** |  | |  | 6 |  |  |  |  |  | | [19] |
|  | **Myriapoda** |  | | Only species related to marine environments | 2/10 |  |  |  |  |  | | [19,37] |
|  | **Apterygota** |  | |  | 14 |  |  |  |  |  | | [19] |
|  | **Insecta** |  | | Only species related to marine environments | 54/250 |  |  |  |  |  | | [19,37] |
|  | **Chaetognatha** | | |  | **>20** |  |  |  |  |  | | [19] |
|  | **Nemertea** |  | |  | **172** |  |  |  |  |  | | [19] |
|  | **Annelida** |  | |  | **1172** |  |  |  |  | D. Martin, J. Gil, C. Arvanitidis, G. San Martín, E. López, R. Capaccioni, M. Aguado, R. Sardá, J. Parapar, F. Aguirrezabalaga, J. Moreira, J.M. Amoureaux, A. Giangrande, C. Gambi, G. Cantone, C. Lardicci, B. Mikac, N. Simboura, M.E. Çinar, Z. Ergen, N. Ben Eliahu, R. Barnich, D. Fiege, F. Pleijel, A. Mackie, A. Castelli | | |
|  |  | Polychaeta | |  | 1122 |  | 210 | 4.2 species being discovered per year | 70-80 |  | [60,61,62,63, Table S16] | |
|  |  | Clitellata | |  | 44 |  |  |  |  | E. Rota, A. Minelli | [36,37] | |
|  |  | Echiura | |  | 6 |  |  |  |  |  | [35] | |
|  | **Sipuncula** |  | |  | **34** | Poor in southern and deep areas |  | Unknown, but large | **2** | J.I. Saiz | [64,65,66,67,68,69, Table S24] | |
|  | **Phoronida** |  | |  | **3/5** |  |  |  |  | C.C. Emig | [19,70] | |
|  | **Brachiopoda** |  | |  | **14/15** |  | **2** |  |  | C.C. Emig, F. Álvarez | [19,71,72] | |
|  | **Mollusca** |  | |  | **2113** | Poor in southern and deep areas | | 5-10 species being discovered every year | **154-203** | J. Templado, H. Mienis, B. Cesare | [73,74,75,76, Table S14] | |
|  |  | Aplacophora (Caudofoveata) | | | 9 |  |  |  | 0 | L. Salvini Plaven |  | |
|  |  | Aplacophora (Solenogastres) | | | 29 |  |  |  | 0 | L. Salvini Plaven |  | |
|  |  | Polyplacophora | |  | 31 |  |  |  | 1 | B. Dell’Angelo |  | |
|  |  | Monoplacophora | |  | 1 |  |  |  | 0 |  |  | |
|  |  | Gastropoda | |  | 1564 |  |  |  | 92 | B. Sabelli, M. Oliverio, S. Gofass, R. Cattaneo Vietti, M. Ballesteros | | |
|  |  | Bivalvia | |  | 400 |  |  |  | 57 |  |  | |
|  |  | Scaphopoda | |  | 14 |  |  |  | 0 | G. Steiner |  | |
|  |  | Cephalopoda | |  | 65 |  | 3 |  | 4 | G. Bello, P. Belcari, P. Jereb, E. Lefkaditou, A. Quetglas, A. Salman, P. Sánchez, R. Villanueva | [73] | |
|  | **Gnathostomulida** | | |  | **10** |  |  |  |  |  | [19] | |
|  | **Gastrotricha** |  | | Meiofauna | **165** | Poor |  |  |  | W.D. Hummon, M.A. Todaro, M. Balsamo | [19,77] | |
|  | **Cycliophora** |  | |  | **1** | Poor |  |  |  |  | [78,79] | |
|  | **Entoprocta** |  | |  | **19** |  |  |  |  |  | [19,35] | |
|  | **Bryozoa** |  | | Mainly benthic | **388** |  | 88 |  | **1??** | C.M. López de la Cuadra, JA. Álvarez, Y.V. Gautier, J.G. Harmelin, J.C. García Gómez, M. Zabala, A. Rosso | [80,81,82,83,84,85,86, Table S22] | |
|  |  | Cyclostomata | |  | 53 | Poor | 17 |  |  |  |  | |
|  |  | Ctenostomata | |  | 44 | Poor | 5 |  |  |  |  | |
|  |  | Cheilostomata | |  | 292 | Poor | 66 |  |  |  |  | |
|  | **Placozoa** |  | |  | **1/2?** |  |  |  |  |  | [19] | |
|  | **Ctenophora** |  | | Mainly pelagic | **>30** |  |  |  |  |  | [19] | |
|  | **Cnidaria (Coelenterates)** | | |  | **757** |  |  |  |  |  | [19,87, and references within] | |
|  |  | Anthozoa | |  | 164 |  | 37 |  | 3 | D. Vafidis, H. Zibrowius  C. Morri, A. Altuna Prados, P.J. López González | [44,88,89,90, Table S13] | |
|  |  | Scyphozoa | |  | 20 |  |  |  |  | F. Boero, B.S. Galil, M. Avian | [19] | |
|  |  | Cubozoa | |  | 1 |  |  |  |  | M. Avian |  | |
|  |  | Hydrozoa | |  | 457 |  |  |  |  | J.M.Gili, F. Boero, J. Bouillon, Á.L. Peña Cantero | [91] | |
|  |  | Myxozoa | |  | 115 |  |  |  |  |  | [19] | |
|  | **Porifera** |  | |  | **681** | Well known except southern areas and the Levantine Sea | | | | J. Vacelet, N. Boury-Esnault, M. Pansini, M.J. Uriz, M. Maldonado, E. Voultsiadou | [92,93,94,95,96] | |
|  |  | Hexactinellida | |  | 8 |  |  |  |  |  |  | |
|  |  | Calcarea | |  | 44 |  |  |  |  |  |  | |
|  |  | Demospongiae | |  | 629 |  | Approx. 302 |  |  |  |  | |
| **Plantae^3^** | **seaweeds (part) and seagrasses** | | | Only benthic | **854** | Limited | **171** |  | **91** | A. Athanasiadis, A. Gómez-Garreta, A. Meinesz, A. Sfriso, A. Vergés, C. Perrone,  C. Rodriguez-Prieto, C.F. Boudouresque, CI Delle Foglie, D. Balata, D. Serio, E. Ballesteros, E. Cecere, F. Boisset, F. Cinelli, F. De Masi, F. Rindi, F. Tripodi, G. Alongi, G. Bressan, G. Furnari, G. Giaccone, G. Sartoni, G.M. Gargiulo, I. Pérez-Ruzafa, J. Rull, L. Babbini-Benussi, L. Piazzi, M. Altamirano, M. Cormaci, M. Verlaque, M.A. Ribera, M.C. Barceló, N. Sánchez, S. Benhissoune | [97] | |
|  | Rhodophyta |  | |  | 657 |  | 150 |  | 73 |  | [98, Table S10] | |
|  | Chlorophyta^4^ |  | | Macroalgae | 190 (180^4^) |  | 20 |  | 17 |  | [99, Table S11] | |
|  | Magnoliophyta or angiospermae | | | | 7 |  | 1 |  | 1 |  | Table S12 | |
| **Protoctista (Protozoa) & Chromista** | | | | Mainly marine and freshwater | **Unknown, first estimate approx. 4400^5^** | Very limited |  |  |  | M. Estrada, J.M. Gasol | [19,100,101,102,103,104,105,106,107,108,109,110,111,112,113,114,115,116,117,118,119,120,121,122] | |
|  | Chlorophyta |  | | Microalgae |  |  |  |  |  |  |  | |
|  |  | Bacillariophyceae | | | 736 |  |  |  |  | Z.R. Velasquez | [113] | |
|  |  | Coccolithophores | | | 166 |  |  |  |  | L. Cros | [114] | |
|  | Dinoflagellata |  | |  | 673 |  |  |  |  | F. Gomez | [19,120] | |
|  | Foraminifera |  | | Benthic and pelagic | >600 |  |  |  |  |  | [110,122,123,124,125]. | |
|  | Heterokontophyta | | |  | 277 |  | 81 |  | 23 | A. Athanasiadis, A. Gómez-Garreta, A. Meinesz, A. Sfriso, A. Vergés, C. Perrone,  C. Rodriguez-Prieto, C.F. Boudouresque, C.I. Delle Foglie, D. Balata, D. Serio, E. Ballesteros, E. Cecere, F. Boisset, F. Cinelli, F. De Masi, F. Rindi, F. Tripodi, G. Alongi | [126, Table S9] | |
| **Prokaryotes** | |  | |  | Not Available | Very limited |  |  |  |  |  | |
| **Bacteria and Archaea** | |  | |  | Not Available | Very limited |  |  |  | J. Gasol, C. Pedrós-Alió, R. Massana, K. Linke, W. Petz | [19,127,128,129,130,131,132] | |
|  | Large cyanobacteria | | |  | 165 |  |  |  |  |  | [19,35] | |
| (1) Not an exhaustive list; (2) Not an exhaustive list; (3) Includes a part of the Macrophytobenthos (red and green seaweeds and seagrasses); (4) 10 species reported within the Chlorophyceae (Volvocales) and Prasinophyceae (Chlorodendrales, Pyramimonadales) are unicellular and can be considered to be phytoplanktonic although they thrive in mediolittoral and supralittoral pools and have been classically included in the checklists of marine macroalgae; (5) This number is highly uncertain (see text section heading “The biodiversity of the ‘smallest’”). | | | | | | | | | | | | |

**References**

1. Notarbartolo di Sciara G (2002) Cetaceans of the Mediterranean and Black Seas: state of knowledge and conservation strategies. A report to the ACCOBAMS Secretariat, Monaco.

2. Bearzi G, Reeves RR, Notarbartolo di Sciara G, Politi E, Canadas A, et al. (2003) Ecology, status and conservation of short-beaked common dolphins *Delphinus delphis* in the Mediterranean Sea. Mammal Review 33: 224-252.

3. Frantzis A, Alexiadou P, Paximadis G, Politi E, Gannier A, et al. (2003) Current knowledge of the cetacean fauna of the Greek Seas. Journal of Cetacean Research and Management 5: 219-232.

4. Reeves R, Notarbartolo di Sciara G (2006) The status and distribution of cetaceans in the Black Sea and Mediterranean Sea. Málaga, Spain: UCN Centre for Mediterranean Cooperation. 142 p.

5. Aguilar JS, Monbailliu X, Paterson A (1993) Status and Conservation of Seabirds: Proceedings of the 2nd Pan-Mediterranean Seabird Symposium. Calvià, Mallorca, 1989. S.E.O., Madrid. 386 p.

6. Yésou P, Sultana J (2000) Monitoring and Conservation of Birds, Mammals and Sea Turtles of the Mediterranean and Black Seas: Proceedings of the 5th MEDMARAVIS Pan-Mediterranean Seabird Symposium, Gozo, Malta, October 1998. Environmental Protection Dpt, Malta. 320 p.

7. Mínguez E, Oro D, de Juana E, Martinez-Abrain A (2003) Mediterranean sea birds and their conservation: Scientia Marina. Vol. 67 ( Suppl.2): 1-148.

8. Groombridge B (1990) Marine turtles in the Mediterranean: distribution, population status, conservation: a report to the Council of Europe, Environment Conservation and Management Division.

9. Camiñas JA (2004) Sea turtles of the Mediterranean Sea: population dynamics, sources of mortality and relative importance of fisheries impacts. Papers presented at the Expert Consultation on Interactions between Sea Turtles and Fisheries within an Ecosystem Context, Rome, 9-12 March 2004: 27.

10. Venizelos L, Papapavlou K, Dunais MA, Lagonika C (2005) A review and reappraisal of research in some previously unsurveyed Mediterranean marine turtle nesting sites. Belgian Journal of Zoology 135: 271-277.

11. NOAA (2007) Hawksbill Sea Turtle (*Eretmochelys imbricata*). Five year Review: Summary and Evaluation: NOAA and U.S. Fish and Wildlife Service. 90 p.

12. Tomás J, Raga JA (2009) Occurrence of Kemp's ridley sea turtle (*Lepidochelys kempii*) in the Mediterranean. Marine Biodiversity Records Marine Biological Association of the United Kingdom 1: 1-2.

13. Fischer W, Bauchot M, Schneider M (1987b) Fiches FAO d’identification des espèces pour les besoins de la pêche. Méditerranée et Mer Noire, zone de pêche 37. Rome, Italie: Service des ressources marines, département des pêches de la FAO.Vol II. Vertébrés.

14. Whitehead P, Bauchot L, Hureau J, Nielsen J, Tortonese E (1986) Fishes of the North-Eastern Atlantic and the Mediterranean. Paris: UNESCO.

15. Fergusson IK. Checklist of sharks frequenting the Mediterranean Sea; 1994. pp. 15-16.

16. Quignard JP, Tomasini JA (2000) Mediterranean fish biodiversity. Biologia Marina Mediterranea 7: 1-66.

17. Compagno LJV (2001) Sharks of the World. An annotated and illustrated catalogue of the shark species known to date. Volume 2. Bullhead, mackerel and carpet sharks (Heterodontiformes, Lamniformes and Orectolobiformes).

18. Golani D, Orsi Relini L, Massuti E, Quignard J (2002) The CIESM Atlas of exotic species in the Mediterranean. Volume I. Fishes. Monaco.

19. Hofrichter Rc (2002) El Mar Mediterráneo. Fauna, Flora, Ecología. II/1. Guía Sistemática y de Identificación. Barcelona: Ediciones Omega. 849 p.

20. Serena F (2005) Field identification Guide to the Sharks and Rays of the Mediterranean and Black Sea. Rome. 97 p.

21. Cavanagh RD, Gibson C (2007) Overview of the conservation status of cartilaginous fishes (Chondrichthyans) in the Mediterranean Sea; IUCN, editor. Gland and Malága.

22. Iglésias SP, Clique A (2009) Identification et Classification des Chondrichtyens et des Actinoptérygiens du Nord-est Atlantique et de la Méditerranée. http://www.mnhn.fr/iccanam/.

23. Salfi M (1931) Gli Ascidiacei dei Golfo di Napoli. Publicazione della Stazione Zoologica di Napoli 11: 293-360.

24. Harant H, Vernières P (1933) Faune de France. Tuniciers. I: Ascidies. Paris, France: P. Lechevalier.

25. Tursi A (1980) Guide por il riconoscimiento delle specie animali delle acque lagunari e costiere italiane. Ascidiacei. Consiglio Nazionale delle Ricerche 4: 7-88.

26. Turon X (1987) Estudio de las ascidias de las costas de Cataluña e Islas Baleares. Barcelona: University of Barcelona. Spain.

27. Ramos-Esplá AA (1988) Ascidias litorales del Mediterráneo ibérico. Faunística, Ecología y Biogeografía. Barcelona: University of Barcelona, Spain.

28. Naranjo S (1995) Taxonomía, zoogeografía y ecología de las ascidias del Estrecho de Gibraltar. Implicaciones de su distribución bionómica en la caracterización de áreas costeras. Sevilla: University of Sevilla, Spain.

29. Tortonese E (1979) Review of present status of knowledge of the Mediterranean echinoderms. Proceedings of the European colloquium on echinoderms Brussels: 141-149.

30. Koukouras A, Sinis AI, Bobori D, Kazantzidis S, Kitsos MS (2007) The echinoderm (Deuterostomia) fauna of the Aegean Sea, and comparison with those of the neighbouring seas. Journal of Biological Research-Thessaloniki 7: 67-92.

31. Allgen CA (1942) Die freilebenden Nematoden des mittelmeeres. Eine zusammenstellung sÃmtlicher aus dem Mittelmeer bekannten Nematoden. Zoologische Jb (Systematik) 76: 1-102.

32. Schuurmans Stekhoven Jr JH (1950) The free living marine nemas of the Mediterranean. I. The Bay of Villefranche. Memoires Institut Royal des Sciences Naturelles de Belgique 37: 1-220.

33. Boucher G (1970b) Deux especes nouvelles de Nematodes de la vase terrigene cotiere de Banyuls-Sur-Mer. Bulletin du Museum National d'Histoire Naturelle, Serie 2 42: 975-983.

34. Vitiello P (1974) Marine nematodes associations in the muddy bottoms of Provence. I. Muddy sediments in sheltered areas and terrigenous coastal muds. Annales de l'Institut Oceaanographique, Paris 50: 145-172.

35. Boudouresque CF (2004) Marine biodiversity in the Mediterranean: status of species, populations and communities. Scientific Reports of Port-Cros National Park, France 20: 97-146.

36. SIBM (2009) Checklist of marine fauna of the Italian seas: www.sibm.it/CHECKLIST/principalechecklistfauna.htm.

37. Riedl R (1983) Fauna and Flora of the Mediterranean: a Systematic Marine Guide for Biologists and Nature Lovers. Hamburg: Verlag Paul Parey. 836 p.

38. Soyer J (1970) Bionomie benthique du plateau continental de la cote catalane franssaise. III. Les peuplements de Copepodes harpacticoides (Crustacea). Vie et Milieu 21: 337-511.

39. Stobbe K (2001) Temporal and spatial variabilities of harpacticoid copepods in the eulittoral zone of a sandy beach on Crete, Greece. Rostock: MSc Thesis, Faculty of Biological Sciences. University of Rostock, Rostock. 63 p.

40. Mitwally H, Montagna PA (2001) Egyptian interstitial Copepoda Harpacticoida with the description of two new species and one new subspecies. Crustaceana: 513-544.

41. Zenetos A, Meric E, Verlaque M, Galli P, Boudouresque CF, et al. (2008) Additions to the annotated list of marine alien biota in the Mediterranean with special emphasis on Foraminifera and Parasites. Mediterranean Marine Science 9: 119-165.

42. Sevastou K, Eleftheriou A (submitted) Meiobenthic diversity in space and time: the case of harpacticoid copepods in two Mediterranean microtidal sandy beaches.

43. Koukouras A, Matsa A (1998) The thoracican cirriped fauna of the Aegean Sea: New information, check list of the Mediterranean species, faunal comparisons. Marine Biodiversity 28: 133-142.

44. Koukouras A, Voultsiadou E, Kitsos MS, Doulgeraki S (2001) Macrobenthic fauna diversity in the Aegean Sea, affinities with other Mediterranean regions and the Black Sea. Bios (Macedonia, Greece) 6: 61-76.

45. Moreira J, Kocak C, Katagan T (2007) *Nebalia kocatasi* sp. nov., a new species of leptostracan (Crustacea: Phyllocarida) from Izmir Bay (Aegean Sea, eastern Mediterranean). Journal of the Marine Biological Association of the UK 87: 1247-1254.

46. Fischer W, Bauchot M, Schneider M (1987a) Fiches FAO d’identification des espèces pour les besoins de la pêche. Méditerranée et Mer Noire, zone de pêche 37. Rome, Italie: Service des ressources marines, département des pêches de la FAO.Vol I. Vegetaux et invertebres.

47. Froglia C, Manning RB (1989) Checklist and key to adult mediterranean Stomatopod Crustacea. In: Ferrero EA, editor. Biology of Stomatopods: Selected Symposia and Monographs, U.Z.I., 3: 265-273.

48. Abelló P, Pretus JL, Corbera J (1994) Occurrence and distribution of some stomatopod crustaceans in the western Mediterranean. Miscel lània Zoològica 17: 107-113.

49. Mavidis M, Aplikioti M, Kirmitzoglou I, Koukouras A (2005) The Euphausiacean fauna (Malacostraca) of the Aegean sea, and comparison with those of the neighbouring seas. Crustaceana 78: 19-27.

50. Heller C (1863) Crustaceen des Südlichen Europa. Wien. 10 pls, 336 p.

51. Koukouras A, Dounas C, Türkay M, Voultsiadou-Koukoura E (1992) Decapod crustacean fauna of the Aegean Sea: new information, check list, affinities. Senckenbergiana Maritima 22: 217-244.

52. Stevcic Z, Galil B (1993) Checklist of the Mediterranean brachyuran crabs. Acta Adriatica 34: 65-76.

53. Udekem d'Acoz Cd (1999) Inventaire et distribution des crustacés décapodes de l'Atlantique nord-oriental, de la Méditerranée et des eaux continentales adjacentes au nord de 25° N. 383. Patrimoines Naturels (MNHN/PN) 40: 1-383.

54. Bacescu M (1941) Les Mysidacés des eaux méditerranéennes de la France (spécialment de Banyuls) et des eaux de Monaco. Bulletin de l´Institut Océanographique 795: 1-46.

55. Wittmann KJ (2001) Centennial changes in the near-shore mysid fauna of the Gulf of Naples (Mediterranean Sea), with description of *Heteromysis riedli* sp. n. (Crustacea, Mysidacea). PSZNI Marine Ecology 22: 85-109.

56. Fage L (1951) Cumacés. Faune de France 54: P. Lechevalier, Paris.

57. Bellan-Santini D, Karaman G, Krapp-Schickel G, Ledoyer M, Myers AA, et al. (1982) The Amphipoda of the Mediterranean. Part 1: Gammaridae (Acanthonotozomatidae to Gammaridae). Mémoires de l'Institut Océanographique de Monaco 13: 364.

58. Bellan-Santini D, Diviacco G, Krapp-Schickel G, Myers AA, Ruffo S (1989) The Amphipoda of the Mediterranean. Part 2. Gammaridea (Haustoriidae to Lysianassidae). Mémoires de l’Institut Océanographique, Monaco 13: 365-576.

59. Ruffo S (1998) The Amphipoda of the Mediterranean. 13: 958.

60. Bellan G (2001) European register of marine species: a check-list of the marine species in Europe and a bibliography of guides to their identification. In: Costello MJ, Emblow C, White RJ, editors. European register of marine species: a check-list of the marine species in Europe and a bibliography of guides to their identification Collection Patrimoines Naturels. Paris, France: Muséum national d'Histoire Naturelle. pp. 214-231.

61. Arvanitidis C, Bellan G, Drakopoulos P, Valavanis V, Dounas C, et al. (2002) Seascape biodiversity patterns along the Mediterranean and the Black Sea: lessons from the biogeography of benthic polychaetes. Marine Ecology Progress Series 244: 139-152.

62. Viéitez J, Alós C, Parapar J, Besteiro C, Moreira J (2004) Annelida Polychaeta I. In: Ramos M, Alba J, Bellés X, Gosálbez J, Guerra A, editors. Fauna Ibérica Vol 23. Madrid: CSIC. pp. 530.

63. San Martín G (2003) Annelida, Polychaeta II: Syllidade. In: Ramos M, Alba J, Bellés X, Gosálbez J, Guerra A, editors. Fauna Ibérica Vol 21. Madrid: CSIC. pp. 554.

64. Pancucci-Papadopoulou MA, Murina GV, Zenetos A (1999) The phylum Sipuncula in the Mediterranean Sea. Monographs on Marine Sciences 2: 1-109.

65. Murina VV (2002) Novyi podvid morskikh chervei (Sipuncula, Golfingiidae) iz Egeiskogo morya. Vestnik Zoologii 36: 87-88.

66. Açik S (2009) Soft-bottom sipunculans in Izmir Bay (Aegean Sea, eastern Mediterranean). Zootaxa 2136: 40-48.

67. Açik S, Murina GV, Çinar ME, Ergen Z (2005) Sipunculans from the coast of northern Cyprus (eastern Mediterranean Sea). Zootaxa 1077: 1-23.

68. Açik S (2007) Observations on the population characteristics of *Apionsoma (Apionsoma) misakianum* (Sipuncula: Phascolosomatidae), a new species for the Mediterranean fauna. Scientia Marina 71: 571-577.

69. Açik S (2008) Occurrence of the alien species *Aspidosiphon (Aspidosiphon) elegans* (Sipuncula) on the Levantine and Aegean Coasts of Turkey. Turkish Journal of Zoology 32: 443-448.

70. Emig CC (2007) Phoronida world database. Available online at http://www.marinespecies.org/phoronida. Consulted on 2010-01-09.

71. Logan A, Bianchi CN, Morri C, Zibrowius H (2004) The present-day Mediterranean brachiopod fauna: diversity, life habits, biogeography and paleobiogeography. Scientia marina 68 (Suppl. 1): 163-170.

72. Emig CC (2006) Brachiopoda world database. Available online at http://www.marinespecies.org/brachiopoda. Consulted on 2010-01-09.

73. Bello G (2004) The biogeography of Mediterranean cephalopods. Biogeographia 24: 201–217.

74. Sabelli B, Giannuzzi-Savelli R, Bedulli D (1990) Catalogo annotato dei molluschi marini del Mediterraneo. Bologna: Libreria Naturalistica Bolognese. 348 p.

75. San Martín G (2003) Annelida, Polychaeta II: Syllidade; Ramos MA, Alba J, Bellés X, Gosálbez J, Guerra A et al., editors. Madrid: CSIC. 554 p.

76. CLEMAM (2009) Check List of the European Marine Mollusca: http://www.somali.asso.fr/clemam/index.

77. Hummon WD, Roidou E (1995) Marine Gastrotricha of Greece. A preliminary report. Biologia Gallo-Helenica 22: 279-289.

78. Matthias O, Peter F, Gonzalo G (2005) Hidden diversity and host specificity in cycliophorans: a phylogeographic analysis along the North Atlantic and Mediterranean Sea. Molecular Ecology 14: 4427-4440.

79. Nedved O (2004) Occurrence of the phylum Cycliophora in the Mediterranean. Marine Ecology Progress Series 277: 297-299.

80. Gautier YV (1962) Recherches écologiques sur les Bryozoaires Chilostomes en Méditerranée occidentale. Recueil des Travaux de la Station marine d'Endoume 38: 1-434.

81. Zabala M (1986) Fauna dels Briozous dels Paisos Catalans. Barcelona: Arxius de la Secció de Ciències 84. Institut d'Estudis Catalans. 833 p.

82. López de la Cuadra CM, Garcia Gomez JC (1988) Briozoos queilostomados del Estrecho de Gibraltar y áreas próximas. Cahiers de Biologie Marine 29: 21-36.

83. Zabala M, Maluquer P (1988) Illustrated keys for the classification of Mediterranean Bryozoa. Treballs del Museu de Zoologia de Barcelona 4: 1-294.

84. Álvarez JA (1992) Briozoos de la Campaña Fauna I. Parte Primera: Ctenostomida, Cheilostomida y Anascina. Cahiers de Biologie Marine 33: 273-297.

85. Harmelin JG, d'Hondt JL (1992) Bryozoaires del parages de Gibraltar (campagne océanographique BALGIM, 1984). 2 - Ctenostomes et Cyclostomes. Bulletin du Museum National d'Histoire Naturelle, Paris Serie 2 14: 605-621.

86. Hayward PJ, McKinney FK (2002) Northern Adriatic Bryozoa from the vicinity of Rovinj, Croatia. Bulletin of the American Museum of Natural History 270: 1-139.

87. Altuna Prados A (2008) Bibliografía de los Cnidarios de la Península Ibérica e Islas Baleares. Documento electrónico disponible en http://www.fauna-iberica.mncn.csic.es/faunaib/Altuna5.pdf, Proyecto Fauna Ibérica, Museo Nacional de Ciencias Naturales, Madrid.

88. Vafidis D, Koukouras A, Voultsiadou-Koukoura E (1994) Octocoral fauna of the Aegean Sea with check list of the Mediterranean species: New information, faunal comparisons. Annales de l’Institut Océanographique 70: 217-229.

89. Chintiroglou CC, Doumenc D, Zamponi M (1997) Commented list of the Mediterranean Actiniaria and Corallimorpharia(Anthozoa). Acta Adriatica 38: 65-70.

90. Voultsiadou E, Vafidis D (2007) Marine invertebrate diversity in Aristotle's zoology. Contributions to Zoology 76: 103-120.

91. Bouillon J, Medel MD, Pagès F, Gili JM, Boero F, et al. (2004) Fauna of the Mediterranean Hydrozoa. Scientia Marina 68 (Suppl. 2): 1-454.

92. Pansini M, Longo C (2003) A review of the Mediterranean Sea sponge biogeography with, in appendix, a list of the demosponges hitherto recorded from this sea. Biogeographia 24: 59-90.

93. Voultsiadou E (2009) Reevaluating sponge diversity and distribution in the Mediterranean Sea. Hydrobiologia 628: 1-12.

94. Van Soest R, Boury-Esnault N, Hooper J, Rützler K, de Voogd N, et al. (2008) World Porifera database: www.marinespecies.org/porifera.

95. SMEBD (2009) World Register of Marine Species: Accessed htttp://www.marinespecies.org on [2009-09-29].

96. Hooper JNA, van Soest RWM (2002) Systema Porifera. A guide to the classification of sponges. New York: Kluwer Academic/Plenum Publishers.

97. Cabioc'h J, Le Floc'h JY, Le Toquin A, Boudouresque CF, Meinesz A, et al. (2006) Guide des algues des mers d'Europe: Délachaux et Niestlé. 272 p.

98. Gomez Garreta A, Gallardo T, Ribera MA, Cormaci M, Furnari G, et al. (2001) Checklist of Mediterranean seaweeds. III. Rhodophyceae Rabenh. 1. Ceramiales Oltm. Botanica Marina 44: 425-460.

99. Gallardo T, Gómez Garreta A, Ribera MA, Cormaci M, Furnari G, et al. (1993) Check-list of Mediterranean seaweeds. II: Chlorophyceae Wille s. l. Botanica Marina 36: 399-421.

100. Navarro M, Massutí M (1940) Composición y ciclo anual del plancton superficial de la bahía de Palma de Mallorca. Notas y Resúmenes Instituto Español de Oceanografia 97: 1-62.

101. Massutí M, Margalef R (1950) Introducción al estudio del plancton marino. Barcelona: Patronato Juan de La Cierva. Sección de Biología Marina. 183 p.

102. Trégouboff G, Rose M (1957) Manuel de planctonologie méditerranéenne. Paris: C.N.R.

103. Balech E (1959) Tintinnoidea del Mediterráneo. Trabajos del Instituto Español de Oceanografía 28: 1-88.

104. Margalef R (1969) Composición específica del fitoplancton de la costa catalana levantina (Mediterráneo Occidental) en 1962-67. Investigación Pesquera 33: 345-380.

105. Kimor B, Wood EJF (1975) A plankton study in the eastern Mediterranean Sea. Marine Biology 29: 321-333.

106. Travers M (1975) Inventaire des protistes du Golfe de Marseille et de ses parages. Annales de l'Institut Océanographique, Paris 51(1): 51-75.

107. Laval-Peuto M, Brownlee DC (1986) Identification and systematics of the Tintinnina (Ciliophora): evaluation and suggestions for improvement. Annuaire de l'Institut Océanographie de Paris 62: 69-84.

108. Margalef R, Estrada M (1987) Synoptic distribution of summer microplankton (Algae and Protozoa) across the principal front in the western Mediterranean. Investigación Pesquera 51: 121-140.

109. Bernard C, Rassoulzadegan F (1994) Seasonal variations of mixotrophic ciliates in the northwest Mediterranean Sea. Marine Ecology Progress Series 108: 295-295.

110. Pujol C, Grazzini CV (1995) Distribution patterns of live planktic foraminifers as related to regional hydrography and productive systems of the Mediterranean Sea. Marine Micropaleontology 25: 187-217.

111. Velasquez ZR, Cruzado A (1995) Inventory of the diatom flora of the NW Mediterranean Sea. Vie et Milieu 45: 249-263.

112. Vaqué D, Blough HA, Duarte CM (1997) Dynamics of ciliate abundance, biomass and community composition in an oligotrophic coastal environment (NW Mediterranean). Aquatic Microbial Ecology 12: 71-83.

113. Velasquez ZR (1997) Fitoplancton en el Mediterráneo Noroccidental: Ph. D. Thesis. Universitat Politècnica de Catalunya. Spain.

114. Cros L, Kleijne A, Zeltner A, Billard C, Young JR (2000) New examples of holococcolith-heterococcolith combination coccospheres and their implications for coccolithophorid biology. Marine Micropaleontology 39: 1-34.

115. Cros L, Fortuño JM (2002) Atlas of northwestern Mediterranean coccolithophores. Scientia Marina 66 (Suppl. 1): 1-182.

116. Cros L (2002) Planktonic coccolithophores of the NW Mediterranean. Barcelona: Universitat de Barcelona, Spain.

117. Dolan JR, Claustre H, Carlotti F, Plounevez S, Moutin T (2002) Microzooplankton diversity: relationships of tintinnid ciliates with resources, competitors and predators from the Atlantic Coast of Morocco to the Eastern Mediterranean. Deep-Sea Research Part I 49: 1217-1232.

118. Modigh M, Castaldo S (2002) Variability and persistence in tintinnid assemblages at a Mediterranean coastal site. Aquatic Microbial Ecology 28: 299-311.

119. Arndt H, Hausmann K, Wolf M (2003) Deep-sea heterotrophic nanoflagellates of the Eastern Mediterranean Sea: qualitative and quantitative aspects of their pelagic and benthic occurrence. Marine Ecology Progress Series 256: 45-56.

120. Gómez F (2003) Checklist of Mediterranean free-living dinoflagellates. Botanica Marina 46: 215-242.

121. Massana R, Balagué V, Guillou L, Pedrós-Alió C (2004) Picoeukaryotic diversity in an oligotrophic coastal site studied by molecular and culturing approaches. FEMS Microbiology Ecology 50: 231-244.

122. Fontanier C, Jorissen FJ, Lansard B, Mouret A, Buscail R, et al. (2008) Live foraminifera from the open slope between Grand Rhône and Petit Rhône Canyons (Gulf of Lions, NW Mediterranean). Deep-Sea Research Part I 55: 1532-1553.

123. Samir AM, Abdou HF, Zazou SM, El-Menhawey WH (2003) Cluster analysis of recent benthic foraminifera from the northwestern Mediterranean coast of Egypt. Revue de Micropaléontologie 46: 111-130.

124. Hyams O, Almogi-Labin A, Benjaminia C (2002) Larger foraminifera of the southeastern Mediterranean shallow continental shelf off Israel. Israel Journal of Earth Sciences 51: 169-179.

125. Mojtahid M, Jorissen F, Lansard B, Fontanier C, Bombled B, et al. (2009) Spatial distribution of live benthic foraminifera in the Rhône prodelta: Faunal response to a continental–marine organic matter gradient. Marine Micropaleontology 70: 177-200.

126. Ribera MA, Gómez A, Gallardo T, Cormaci M, Furnari G, et al. (1992) Check-list of Mediterranean seaweeds. I: Fucophyceae (Warming, 1884). Botanica Marina 35: 109-130.

127. Zaballos M, López-López A, Ovreas L, Bartual SG, D'Auria G, et al. (2006) Comparison of prokaryotic diversity at offshore oceanic locations reveals a different microbiota in the Mediterranean Sea. FEMS Microbiology Ecology 56: 389-405.

128. Alonso-Sáez L, Balagué V, Sà E, Sánchez O, González JM, et al. (2007) Seasonality in bacterial diversity in north-west Mediterranean coastal waters: assessment through clone libraries, fingerprinting and FISH. FEMS Microbiology Ecology 60: 98-112.

129. Feingersch R, Suzuki MT, Shmoish M, Sharon I, Sabehi G, et al. (2009) Microbial community genomics in eastern Mediterranean Sea surface waters. The ISME Journal: doi:101038/ismej200992.

130. Garczarek L, Dufresne A, Rousvoal S, West NJ, Mazard S, et al. (2007) High vertical and low horizontal diversity of *Prochlorococcus* ecotypes in the Mediterranean Sea in summer. FEMS Microbiology Ecology 60: 189-206.

131. Blumel M, Suling J, Imhoff JF (2007) Depth-specific distribution of Bacteroidetes in the oligotrophic Eastern Mediterranean Sea. Aquatic Microbial Ecology 46: 209.

132. Pedrós-Alió C, Pommier T, Acinas SG, Gasol JM (Submitted) Patterns of spatial bacterial diversity in NW Mediterranean by 454.

## **Table S2. List of co-authors with expertise, specific contribution to the synthesis,** and contact information

|  |  |  |
| --- | --- | --- |
| **Experts** | **Main contribution to the synthesis** | **Contact information** |
| Aguzzi, Jacopo | Methodology aspects of sampling biodiversity | Institut de Ciencies del Mar (CSIC). Passeig Maritim 37-49. E-08003 Barcelona. Catalunya. Spain |
| Ballesteros, Enric | Macroalgae | Centre d’Estudis Avançats de Blanes (CEAB), Consejo Superior de Investigaciones Científicas (CSIC), Carrer d’accés a la Cala Sant Francesc 14, 17300-Blanes (Girona), Catalunya, Spain |
| Barile, Josephine | Aquamaps model | The WorldFish Center-Philippine Office, Khush Hall, IRRI, College, Los Baños, Laguna 4031, Philippines |
| Ben Rais Lasram, Frida | Fishes and climate change | Laboratoire Ecosystèmes Lagunaires UMR 5119, Université Montpellier 2, cc 093, Place E. Bataillon, 34095 Montpellier Cedex 5, France & Laboratoire Ecosystèmes et Ressources Aquatiques UR03AGRO1, Institut National Agronomique de Tunisie, 43 avenue Charles. Tunisie |
| Bianchi, Carlo Nike | Climate change and global threats | Dipartimento per lo studio del Territorio e delle sue Risorse). Università di Genova, Corso Europa 26. I-16132 Genova, Italy |
| Coll, Marta | Global estimates, patterns, and drivers | Institut de Ciencies del Mar (CSIC). Passeig Maritim 37-49. E-08003 Barcelona. Catalunya. Spain & Dalhousie University, Biology Department. 1355 Oxford Street, Halifax, Nova Scotia. B3H 4J1. Canada |
| Corbera, Jordi | Cumaceans | Carrer Gran, 90, 08310 Argentona, Catalonia, Spain |
| Dailianis, Thanos | Sponges | Department of Zoology, School of Biology, Aristotle University of Thessaloniki, 54124 Thessaloniki, Greece & Hellenic Centre for Marine Research, Institute of Marine Biology and Genetics, Thalassocosmos, Herakleio, Crete, Greece |
| Danovaro, Roberto | Deep-sea biodiversity, patterns, and drivers | Dipartimento Scienze del Mare, Polytechnic University of Marche, Via Brecce Bianche 0131, Ancona, Italy |
| Estrada, Marta | Phytoplankton | Institut de Ciencies del Mar (CSIC). Passeig Maritim 37-49. E-08003 Barcelona. Catalunya. Spain |
| Froglia, Carlo | Decapods | Istituto di Scienze Marine—CNR, Sede di Ancona, Largo Fiera della Pesca, 60125 Ancona, Italy |
| Galil, Bella S. | Aliens | National Institute of Oceanography, Israel Oceanographic & Limnological Research, POB 8030, Haifa 31080, Israel |
| Gasol, Josep Mª | Bacteria and Archaea | Institut de Ciencies del Mar (CSIC). Passeig Maritim 37-49. E-08003 Barcelona. Catalunya. Spain |
| Gertwagen, Ruthy | Historical information | HMAP Mediterranean and the Black Sea Project coordinator and Teams Leader; Haifa University and Oranim Academic College, Mount Carmel, Haifa 31905. Israel |
| Gil, João | Polychaetes | Centre d’Estudis Avançats de Blanes (CEAB), Consejo Superor de Investigaciones Científicas (CSIC), Carrer d’accés a la Cala Sant Francesc 14, 17300-Blanes (Girona), Catalunya. Spain |
| Guilhaumon, François | Fishes and climate change | Laboratoire Ecosystèmes Lagunaires UMR 5119, Université Montpellier 2, cc 093, Place E. Bataillon, 34095 Montpellier Cedex 5, France |
| Kaschner, Kristine | Aquamaps model | Evolutionary Biology & Ecology Lab, Institute of Biology I (Zoology), Albert-Ludwigs-University, 79104 Freiburg, Germany |
| Kesner-Reyes, Kathleen | Aquamaps model | The WorldFish Center-Philippine Office, Khush Hall, IRRI, College, Los Baños, Laguna 4031, Philippines |
| Kitsos, Miltiadis-Spyridon | Echinoderms | Department of Zoology, School of Biology, Aristoteleio University of Thessaloniki, 541-24 Thessaloniki, Greece |
| Koukouras, Athanasios | Echinoderms | Department of Zoology, School of Biology, Aristoteleio University of Thessaloniki, 541-24 Thessaloniki, Greece |
| Lampadariou, Nikolaos | Meiobenthos | Hellenic Centre for Marine Research, PO Box 2214, 710 03 Heraklion, Crete, Greece |
| Laxamana, Elijah | Aquamaps model | The WorldFish Center-Philippine Office, Khush Hall, IRRI, College, Los Baños, Laguna 4031, Philippines |
| López-Fé de la Cuadra, Carlos Mª | Bryozoans | Laboratorio de Biología Marina, Departamento de Fisiología y Zoología. Facultad de Biología (Universidad de Sevilla). Avda. Reina Mercedes, 6. 41012 Sevilla Spain. |
| Lotze, Heike K. | Historical information | Dalhousie University, Biology Department. 1355 Oxford Street, Halifax, Nova Scotia. B3H 4J1. Canada |
| Martin, Daniel | Polichaetes | Centre d’Estudis Avançats de Blanes (CEAB), Consejo Superor de Investigaciones Científicas (CSIC), Carrer d’accés a la Cala Sant Francesc 14, 17300-Blanes (Girona), Catalunya. Spain |
| Mouillot, David | Fishes and climate change | Laboratoire Ecosystèmes Lagunaires UMR 5119, Université Montpellier 2, cc 093, Place E. Bataillon, 34095 Montpellier Cedex 5, France |
| Oro, Dani | Seabird diversity and patterns | IMEDEA (CSIC-UIB) Miquel Marques 21. 07190 Esporles, Spain |
| Piroddi, Chiara | Top predators. Patterns and drivers | Fisheries Center, Aquatic Ecosystems Research Laboratory (AERL). 2202 Main Mall. The University of British Columbia. Vancouver, BC. Canada V6T 1Z4 |
| Raicevich, Saša | Historical information | ISPRA -Istituto Superiore per la Ricerca e la Protezione Ambientale, Loc. Brondolo, 30015 Chioggia (Italy); member of the steering committee HMAP (History of Marine Animal Population) of the Mediterranean and the Black Sea projects |
| Saiz-Salinas, José Ignacio | Sipunculans | University of the Basque Country. E-48080 Bilbao, P.O.Box 644, Spain |
| San Vicente, Carles | Mysidacean | C/Nou núm. 8. 43839 Creixell-Tarragona. Spain |
| Somot, Samuel | Climate change | Météo-France, Centre National de Recherches Météorologiques, 42 avenue Gaspard Coriolis, 31057, Toulouse Cedex, France |
| Steenbeek, Jeroen | GIS and spatial patterns | Fisheries Center, Aquatic Ecosystems Research Laboratory (AERL). 2202 Main Mall. The University of British Columbia. Vancouver, V6T 1Z4 BC. Canada |
| Templado, José | Mollusks | Biodiversidad y Biologia Evolutiva. Museo Nacional de Ciencias Naturales. C/ José Gutiérrez Abascal 2. Madrid 28006. Spain |
| Turon, Xavier | Tunicates | Centre d’Estudis Avançats de Blanes (CEAB), Consejo Superior de Investigaciones Científicas (CSIC), Accés a la Cala Sant Francesc 14, 17300-Blanes (Girona), Spain |
| Vafidis, Dimitris | Anthozoans | University of Thessaly, School of Agricultural Sciences, Department of Ichthyology and Aquatic Environment, Nea Ionia, Magnesia, Greece |
| Villanueva, Roger | Cephalopods | Institut de Ciencies del Mar (CSIC). Passeig Maritim 37-49. E-08003 Barcelona. Catalunya. Spain |
| Voultsiadou, Eleni | Sponges | Department of Zoology, School of Biology, Aristotle University of Thessaloniki, 54124 Thessaloniki, Greece |
|  |  |  |

## **Text S1. Taxonomic revision and methodological specifications**

Here we provide the detailed information for the taxonomic revision of macrophytes (seaweeds and seagrasses), invertebrates and Ascidiacea (subphylum Tunicata, phylum Chordata) in the Mediterranean Sea. This information is summarized in Table 1 in the main body of the study and Table S1, while checklists and specific figures are included in Tables S7-S29 and Figures S1-S6.

**Seaweeds and seagrasses**

Here we include a wide array of organisms usually known as seaweeds, and a reduced group of aquatic flowering plants known as seagrasses. They are a phylogenetic heterogeneous group of eukaryotic photosynthetic organisms. The application of molecular tools to the classification of living organisms has led to a better understanding of the phylogenetic relationships between them and has been demonstrated that brown seaweeds are included in the group Chromista while red seaweeds, green seaweeds, and flowering plants belong to Plantae. Here we do not include blue green algae (phylum Cyanobacteria), which, although classically considered in the textbooks of botany, they are prokaryotic. Most of the brown, red and green algae grow usually attached in the sea bottom or the intertidal zone, while blue greens both thrive as a component of the benthos and the plankton. Their taxonomy is being reshaped continuously, and it is hard to know the real diversity of this group at the "species" level.

The Mediterranean Sea was probably the first area in the world where SCUBA diving techniques were used in the study and collection of macroalgae [1], which is the main and best methodology currently in use. However, deep waters below 50 meters depth are still usually sampled by indirect methods such as dredging or bottom trawling. Plants thriving in the mediolittoral and upper infralittoral zone can be easily collected by hand with no special equipment or simply with the provision of a face mask and a snorkel. See Tsuda and Abbott [2] for a more detailed description of collection, handling, preservation and identification of macroalgae.

Athanasiadis [3] provides a thorough revision of the history of Mediterranean phycology (or algae studies), which began long time ago with the descriptions of several algal species by Theophrastus three centuries B.C., increased during the early Linnean period (Gmelin, Lamarck, Lamouroux, Roth, Bory de Saint Vincent and others), was maintained in the 19^th^ century (C. Agardh, J. Agardh, Kützing, Meneghini, Ardissone, Montagne, Schmitz, Berthold, Foslie and others) and continued until the end of the 19^th^ century, when Zanardini published the first Mediterranean illustrated flora, and the start of the 20^th^ century when Preda published a red algal flora of Italy, Sauvageau his monograph on *Cystoseira*, and De Toni his monumental "Sylloge Algarum". Afterwards, Hamel, Funk, J. Feldmann, Feldmann-Mazoyer, Dangeard, and Ercegovic made important contributions to the phycology in the mid of the 20^th^ century. At present, there are several teams working in the taxonomy of Mediterranean seaweeds, mainly in Italy, France and Spain. Several countries or regions have checklists of marine macroalgae: Morocco, Algeria, Tunis, Libya, Malta, Alexandria (Egypt), Turkey, Aegean Sea, Adriatic Sea, Italy, Sicily, Corsica, eastern Pyrenees (France), and Catalonia and Andalusia (Spain). Moreover, partial checklists of Mediterranean seaweeds have been published since 1992 [4,5,6]. There are also studies focused in introduced species [e.g. 7,8,9,10], a subject that is in constant revision [11].

The total number of taxa currently present in the Mediterranean basin is 1131 (see the annotated checklist of every phyla and for the criteria used in making the lists, Table S7). Phaeophyceae (268 taxa), Pelagophyceae (two species), and Xanthophyceae (seven species) are the three classes within the phylum Heterokontophyta with benthic representatives (this class also hosts the Chrysophyceae, Raphidophyceae, Dictyochophyceae, and others with planktonic representatives). The Mediterranean representatives of the phylum Rhodophyta belong to five classes: Bangiophyceae (eight species), Compsopogonophyceae (17 species), Porphyridiophyceae (1 species), Stylonematophyceae (five species), and Florideophyceae (626 taxa). The highly diverse phylum Chlorophyta hosts five classes with Mediterranean benthic representatives: Chlorophyceae (12 species), Prasinophyceae (six species), Trebouxiophyceae (three species), Ulvophyceae (73 taxa) and Bryopsidophyceae (52 taxa). Some (ten) of the species reported within the Chlorophyceae (Volvocales) and Prasinophyceae (Chlorodendrales, Pyramimonadales) are unicellular and can be considered to be phytoplanktonic, although they thrive in mediolittoral and supralittoral pools and have been classically included in the checklists of marine macroalgae. Thus, strictly benthic chlorophytes number 180 instead of the 190 reported in Table 1. We also include in the list of the flowering plants (phylum Magnoliophyta) those that are usually considered as seagrasses (four autochthonous species and one introduced) as well as two other species typical from brackish waters that they can also be collected in extremely shallow lagoons and sheltered bays (*Ruppia* spp.).

The percentage of endemic species at a basin level ranges from approximately 10% in the phylum Chlorophyta to 30% in the phylum Heterokontophyta, with an average of 22.3% for all the macrophytobenthos (see Table S8). The higher number of endemics is found within the Rhodophyceae, which hosts a large number of deep water red algae considered to be endemic. However, this number is decreasing as increasing studies of macroalgae are made in Macaronesian islands and warm-temperate eastern Atlantic Ocean (coasts of Portugal and Spain) using SCUBA. They are reporting a high number of Mediterranean supposed endemics [e.g. 12,13,14,15]. Within the red algae the highest numbers of endemics are found in *Polysiphonia* (12) and *Acrochaetium* (nine), but both genus need nomenclatural and taxonomical reinvestigations, and these numbers could decrease significantly. Other genera with a high number of endemics include *Peyssonnelia* (six), *Rodriguezella* (four), *Osmundea* (four), and *Kallymenia* (three). *Ptilophora mediterranea* is an interesting endemic of the eastern Mediterranean basin with its closest relatives being found in the Indian Ocean, suggesting that it is a paleoendemic that overcame the Messinian salinity crisis. Within the brown algae, the genus *Cystoseira* alone accounts for 37 endemic taxa (23 species, and 14 infraspecific entities), which makes this genus a landmark in the marine Mediterranean flora. Most *Cystoseira* endemic species are considered neoendemic as they have probably evolved from Atlantic taxa entering the Mediterranean Sea from the Atlantic Ocean starting after the Messinian salinity crisis [16]. Moreover species of the genus *Cystoseira* act as ecosystem engineers in sublitoral Mediterranean communities and are of paramount ecological importance, similar to that played by species of the order Laminariales in other temperate seas and oceans. Also a member of the order Fucales, *Fucus virsoides,* is a neoendemic restricted to the northern Adriatic and the only true Mediterranean representative of this genus. Another important endemic brown alga is *Laminaria rodriguezii*, known from the Adriatic and the western basin, with apparently no relationships with other European species of this genus, and considered to be a Tethys relic [17]. Stands of *Laminaria rodriguezii* are restricted to deep waters, usually below 70 meters depth. Other canopy-forming Laminariales and Tilopteridales (e.g. *Laminaria ochroleuca*, *Saccorhiza polyschides*) are very uncommon in the Mediterranean and they are only found in the Messinian strait, between Italy and Sicily, and in places subjected to surface water inflow from the Atlantic ocean (southern Spain and Moroccan and Algerian coasts). Amongst the green algae, the highest number of endemics is found in *Ulva* but, again, this is a genus where nomenclatural and taxonomical reinvestigations are required. Within the flowering plants, *Posidonia oceanica* is the only endemic, with its closest relatives found again in the Indian Ocean (coasts of southern Australia), suggesting that *P. oceanica* is a Tethys relic [17]. Moreover, *P. oceanica* forms extensive meadows in the infralittoral zone, from the surface in sheltered areas to more than 40 m depth in the crystal-clear waters of the eastern Mediterranean, which makes it the most important Mediterranean shallow-water ecosystem. It is only absent in the easternmost area of the Mediterranean, the Moroccan coast and areas of southern Spain situated close to the Strait of Gibraltar.

The Mediterranean Sea is a hot spot for marine introduced species and up to 114 introduced macrophytes have been reported, representing the 10% of the known marine flora. At present, the main vector of introduction of marine macrophytes is aquaculture which surpasses the Suez Canal [7]. The highest number of exotic algae is currently found in coastal lagoons from the Gulf of Lions and northern Adriatic and not in the eastern basin, unlike other groups [18]. According to Boudouresque and Verlaque [8], at least eight introduced species merit the category of invasive as they play a conspicuous role in the recipient ecosystems: *Sargassum muticum, Stypopodium schimperi, Acrothamnion preissii, Asparagopsis armata, Lophocladia lallemandii, Womersleyella setacea, Caulerpa racemosa var. cylindracea, Caulerpa taxifolia* and *Halophila stipulacea*. However, sometimes the invasive capacity of these species is not the same in the different sub-basins, geographical areas or environments and we still do not know which are the features of the species and the environment that allow a species to become invasive.

There is a gradient of species richness between the western to the eastern basin as it has been observed in most groups of organisms (Table 2). For example of the total number of 263 species of the order Ceramiales (Rhodophyta) reported in the checklist, 94% appear in the western basin, 80% in the border between eastern and western basins (Sicily, Tunisia, Ionian Sea), 75% in the Adriatic, and 73% in the eastern basin.

Some seagrass meadows, algal stands, and algal-dominated communities and landscapes are known to be threatened in the Mediterranean [19]. Airoldi and Beck [20] reported coastal development and water quality, followed by destructive fishing, and diseases, pests and predators as the main drivers of the loss of seagrass meadows and macroalgal stands along European coasts, including the Mediterranean. Coastal management, and chemical pollution, as well as trawling, invasive species, increased epiphytism and increased herbivory by sea-urchins are amongst the main causes of the decline of *Posidonia oceanica* meadows [21]. Overgrazing by sea urchins, out-competition by mussels, habitat destruction related to coastal management, chemical pollution, increased water turbidity, human trampling and direct plant destruction attributed to net fishing and even scientific sampling have been blamed for the local extinction of up to 11 taxa belonging to the genera *Cystoseira* and *Sargassum* in the eastern Pyrenees (France) [22]. Colonization by turf algae [23] and global change [24] have also been considered as factors explaining the decrease in Fucales (i.e. brown algae). Increasing abundance of turf-forming, filamentous, or ephemeral algae are also reported as the main cause for the decline of macroalgal stands [25,26]. Trawling, alien invasions, waste waters, diving activities and large scale events involving mass-mortalities are reported as the main causes of disturbance affecting deep-water coralline algae-dominated environments (coralligenous and maërl beds) [27]. At present there is no species of macroalgae or seagrass that has became extinct at the basin scale, but there are some reports of extinctions at local scales [e.g. 22], which can result in a total extinction for some endemics with reduced geographical distribution (e.g. some *Cystoseira* spp.).

The only text regarding habitat and species protection at a regional scale that has been signed by all the Mediterranean countries is the Barcelona Convention. Two Action Plans ("Marine Vegetation" and "Coralligenous and other calcareous bioconcretions") specifically deal with the protection of macrophytobenthos and the habitats they constitute. Another Action Plan ("Introduced and Invasive Species") and the Protocol concerning Specially Protected Areas and Biological Diversity ("SPA Protocol") deal also in part with marine macroalgae and seagrasses. At present, there are 14 species of macrophytes that are listed in the Annex II of the SPA Protocol but in 2009 a list with 44 species was proposed to the National Focal Points for approval. Engineering species, both fleshy (most *Cystoseira* spp., *Sargassum* spp., *Laminaria* spp., *Posidonia oceanica*, *Zostera* spp.) and calcareous (*Titanoderma ramosissimum*, *T. trochanter*, *Tenarea tortuosa*, *Lithophyllum byssoides*) will all be hopefully included in the Annex II in the near future, as well as some rare and highly threatened deep-water endemics.

At a European level there are several EEC Directives that protect marine vegetation: Habitats Directive (92/43/EEC), Water Framework Directive (2000/60/EC) and Marine Strategy Directive (2008/56/EC). Within the Habitats Directive, although no marine macrophytes are listed in the Annex II - species whose conservation requires the designation of special areas of conservation -, *Posidonia oceanica* meadows are a priority natural habitat type and Member States have designated special areas of conservation to ensure its protection. The Water Framework Directive indirectly protects macroalgae and seagrasses as its purpose is to prevent further deterioration and to protect/enhance the ecological status of aquatic coastal (and freshwater) ecosystems, in a similar way that it is stated in the Marine Strategy Directive for all marine ecosystems. Moreover, a Council Regulation (EC 1967/2006, 21 December 2006) concerning management measures for the sustainable exploitation of fishery resources in the Mediterranean Sea, prohibits fishing with trawl nets, dredges, seines or similar nets above seagrass meadows, coralligenous concretions and maërl beds, which, if correctly implemented in all Member States, should be an important measure for protection of these habitats.

However, even with all these regulations, and as far as the protection of marine macrophytobenthic diversity is concerned, habitat destruction and degradation, changes in water quality and turbidity associated to pollution, habitat modification due to changes in the food web of anthropogenic origin, and invasive species will continue to be the major threats in the short and mid-term. We already know that the impact of increasing temperatures and decreasing pH associated to climate change will enhance the development of warm water species (and doing so the expansion of Lessepsian migrants), will cause the rarefaction or even extinction of some species of cold water affinities, will enhance the production of fleshy algae, and will inhibit the growth of calcareous algae [e.g. 28,29].

**Animalia**

All animal phyla with marine representatives are present in the Mediterranean Sea, including the puzzling phyla discovered in recent times, Loricifera and Cycliphora, by Reinhardt Kristensen in 1983, and R. Kristensen and Peter Funch in 1995, respectively. Loricifera was first recorded in the Mediterranean Sea by Todaro and Kristensen [30] with the description of the new species *Nanoloricus khaitatus*. Larvae of two additional undescribed species of this phylum have been mentioned by Hofrichter [31]. On the other hand, Baker et al. [32] and Baker and Giribet [33] pointed out the presence of at least two species of Cycliphora in the Mediterraean, *Symbium pandora* and a undescribed species found in Croatia.

In this synthesis, we especially focused our efforts on diversity of the phyla a) Porifera, b) Cnidaria, with emphasis on Anthozoa, c) Mollusca, d) Annelida, with emphasis on Polychaeta, e) Arthropoda, with emphasis on Decapoda, Cumacea and Mysidacea, f) Bryozoa, g) Echinodermata, h) Sipuncula, i) other invertebrates such as nematodes, and less conspicuous species of the meiobenthos, j) Tunicata with emphasis on Ascidiacea, and k) the subphylum Vertebrata (including fish, marine mammals, sea turtles and seabirds).

**- Porifera**

Mediterranean sponges are an important component of the sublittoral and circalittoral hard substrata communities. They thrive in sciaphilic habitats such as the coralligenous environment [34], submerged or semi-submerged caves [35,36], living also in the proximity of hydrothermal vents [37]. This phylum has been a subject of interest for humans in the littoral of the Mediterranean Sea since the time of Aristotle (4^th^ century BC), who reported five different sponge species in his zoological writings [38]. However, systematic investigation of sponge species occurred after the 17^th^ century. Mediterranean sponge faunal list (at least demosponges which contain 85% of all living sponges) has been recently reviewed and updated by Pansini and Longo [39] and Voultsiadou [40]. Here we updated previous efforts using literature references.

The total number of species from the Mediterranean Sea is to date 681, including 629 Demospongiae, 44 Calcarea, and 8 Hexactinellida. Lists of the Porifera species recorded from the Mediterranean are available in the World Porifera Database [41] and the World Register of Marine species [42], and corresponding percentages (calculated according to the *Systema Porifera*, [43] for each demosponge order separately are illustrated in Figure S1. Due to their sessile habit and their short-lived, current dependant larvae [44], a large number of the Mediterranean demosponges (~48%) are endemic and only 11.5% are cosmopolitan or circumtropical [39]. The strong zoogeographical affinity of the Mediterranean sponge fauna with that of the Atlantic [45] is reflected in species composition, with 37.5% of the Mediterranean sponges being of Atlantic origin, while only few (~3%) are Indo-Pacific [39]. Although several species had been assumed to be Lessepsian migrants [46], recent studies [47] support that their identity with known Red Sea or Indo-Pacific species can be either rejected or remains highly doubtful, and that they are rather thermophilous remnants from an ancient warm period of the Mediterranean. Deep-sea sponges have been studied mostly in the western Mediterranean [48,49]; although bathyal species have been considered as widely distributed [50] and eurybathic [51] recent research revealed several species endemic in the bathyal zone of the eastern Basin [52].

The distribution of demosponge species and genera richness in the different regions of the Mediterranean is also uneven (Figure S2). Four major zoogeographic sub-areas have been identified in the Mediterranean according to the affinities of their sponge fauna: the north-western, north-eastern, the central zone, and the south-eastern areas [40]. A clear prevalence of the north-western basin, which hosts 78% of the total Mediterranean demosponge species and a decline pattern in sponge diversity from the north-western to the south-eastern is observed. The 14 demosponge orders are represented all over the Mediterranean; nevertheless, a gradual decrease in species numbers of Poecillosclerida and an increase of Dictyoceratida, Halichondrida, and Homosclerophorida is observed from the northwest to the southeast Mediterranean [53].

The Mediterranean sponge fauna can be characterized as well studied. However, the knowledge of the distribution of the sponge fauna is far from uniform. While substantial effort has been invested in the study of the northern coastline, investigation in the southern areas and the Levantine has been arguably sporadic and limited [39]. This is especially crucial for environments where sponge diversity is acknowledged to be high, such as sciaphilic habitats, or those which constitute a promising field for discovering new biodiversity, such as deep-sea and extreme environments [37,52,54]. Lately there has been an additional source of new sponge diversity in the form of cryptic species. The difficulties that sponges manifest to the taxonomist are notorious, mostly due to the lack or plasticity of distinguishing morphologic characters that is inherent in several taxa or groups [55]. Molecular data from recent studies have highlighted this concealed biodiversity [56,57,58,59,60].

Sponges demonstrate a wide variety of roles related to the functioning of marine ecosystems, including creation and modification of substrate, benthic-pelagic coupling, as well as a spectrum of interactions and associations with marine organisms [61,62,63]. Although sponges are present in most aquatic environments, the preponderance of their biodiversity occurs in specific habitats, such as coastal hard substrate formations, caves and overhangs, reefs and seamounts, as well as detritic and coralligenous seabeds. These ecosystems are highly vulnerable to degradation both by localized factors such as coastal pollution or industrial fishing [64,65] and global climate change phenomena [66]. The latter can also have a direct effect on sponge diversity, promoting mortality events on several species of the littoral community [67,68]. Thus, the protection of Mediterranean sponge biodiversity is utterly dependant on the protection of those sensitive aquatic environments. Initiations on protection and management plans both in local and global scale will thus have critical effect on the preservation of evidenced or yet unspecified sponge diversity. Sponges also frequently act as ecosystem engineers offering a living substratum to numerous other organisms [69]. Thus, they constitute important biodiversity hotspots in the Mediterranean environment deserving special attention and protection.

Sponges can be commercial products, with a commercial interest that has been traditionally restricted to the common high quality ‘bath sponges’ of the Mediterranean. Five species, *Spongia officinalis*, *S. mollisima*, *S. zimocca*, *S. lamella,* and *Hippospongia communis* have been harvested since early historical times and intensively throughout the 20^th^ century, especially at the eastern and southern part of the basin [70]. The economic and social importance of bath sponges has been significant, as they were, and still are, exported and utilized worldwide [71]. Moreover, the Mediterranean is rich in sponge species with potential bioactive components [72] producing secondary metabolites with anticancer, antibiotic, anti-inflammatory, or antifouling activity [73]. Since production requirements can not be sustainably met by harvesting wild populations, *in situ* sponge culture is being investigated [74]. Mariculture has been investigated for the production of Mediterranean bath sponges [75], especially after the natural populations faced a dramatic decline due to the combined effect of uncontrolled harvesting and several mass mortality events [76], as well as climate warming [68,77].

Progress towards management and protection measures aiming at species is critical, regarding sponges utilized as biological resources. Those include the Mediterranean bath sponges, as well as species with proved or potential value for the pharmaceutical industry. The lack of control characterizing intensive bath sponge harvesting throughout the 20^th^ century in the Mediterranean is acknowledged as an apparent cause for the recent decline of natural populations. Sixteen Mediterranean sponge species have been included in the Barcelona (Annex II and III) the Bern Convention (Appendices II and III) as worthy of protection or exploitation regulation.

**- Cnidaria**

We summarized available information for the Classes hydrozoans, scyphozoans and cubozoan [31,78], while a special emphasis is put on anthozoans. Anthozoans are common in most marine habitats from shallow to deep oceanic waters. Although most anthozoans occur on hard substrata (Gorgonacea, Scleractinia, etc.), a large number are adapted to life in mud, sand or gravel (Pennatulacea, Ceriantharia) in big range of current velocities but favoring for strong ones [e.g. 79,80]. They feed on nanoeukaryotes, dinoflagellates, diatoms, ciliates, as well as detrital POC and are generally considered microplankton suspension feeder [81]. For some the nutrition comes via their symbiotic relationship with small algae living in their gastrodermis and other are carnivorous [e.g. 82,83,84]. Although Aristotle (4^th^ century BC) was the first to mention the presence of four anthozoans in the Aegean Sea [38], the knowledge on biodiversity of this group in the Mediterranean Sea rose after 17^th^ century. Here we revise previous estimates of anthozoan diversity.

A total of 757 species of Cnidaria are currently quantified (Table 1 and Table S1). Anthozoa species recorded so far are 164 species including 51 Octocorallia and 113 Hexacorallia (Table S13). Two primary faunal components are recognized: Atlantic-Mediterranean (62.20% of the fauna by numbers of species) and endemic (21.95%). The boreal component contributes only minimally (1.83%), while the remaining fauna is made up of cosmopolitan (8.54%) and Amphi-Atlantic species (4.88%). There is only one confirmed Indo-Pacific component (*A. erythraea*) in the Mediterranean Sea. Introduced corals into the Mediterranean have been cited due to shipping via the Atlantic: *Oculina patagonica* and *Diadumene cincta* [85,86], and in the Red Sea: *Acabaria erythraea* [87].

The geographical and bathymetric distribution of anthozoan species and genera richness in the different regions of the Mediterranean is rather uneven (Table 2, and Table S13). Three major zoogeographical zones have been identified in the Mediterranean according to the affinities of their anthozoan fauna: a zone of high diversity (Alboran Sea, Western basin, Tyrrhenian basin), a zone of moderate diversity (Adriatic Sea, Aegean Sea), and a zone of impoverished diversity (Ionian basin, Levantine basin). The higher species diversity in the western basin can be explained by the occurrence of a number of Atlantic species with eastern boundaries in the Mediterranean, i.e. in various areas of the western basin [88,89,90]. Intensive research effort in the western Mediterranean may also be responsible for this difference in numbers. Another reason is the general impoverishment in the diversity of the eastern part biota due to the oligotrophic conditions in the area [91].

Corals have been used since antiquity for jewellery, costume accessories and souvenirs [92], and also produce diverse compounds useful in medicine [93]. Harvesting and ripping out of colonies, as well as chemical pollution, burial and sediments deposition, are harming too many species, particularly permanently attached, erect forms such as gorgonians, antipatharians and scleractinians. Red coral (*Corallium rubrum*) is a precious coral and has been widely sought after since ancient times in the Mediterranean. Nowadays it is one of the most over-exploited invertebrates in this sea [e.g. 92,94].

Less than 20% of the corals living in the Mediterranean have been included under the annexes of the conventions for the protection of animal life. The most of them (85%) are only protected under Annex II of CITES, which does not extend full protection but rather regulates their commercial trade. Six of them are protected under EC legislation or international conventions and two are included in the lists of maximum protection [95].

**- Mollusca**

Mollusks are important components of marine communities worldwide, making up to 15-25% of the benthic macrofauna. This is one of the few phyla that are routinely taken into consideration in marine biodiversity surveys and are considered to be an ‘appropriate indicator group’ for rapid assessment of diversity inhabiting a particular area [96]. Their importance did not escape early human settlers who clearly appreciated their value as seafood or the beauty of their shells. The Mediterranean molluscan fauna is one of the most anciently studied. Most malacologist, professional or amateur, are aware of the seminal works of Forbes (1844) for the Aegean Sea, Philippi (1836) for Sicily, or Bucquoy, Dautzenberg and Dollfus (1882-1898) from the French Mediterranean coast, among others. In the late 19^th^ century, syntheses of available knowledge on the Mediterranean mollusks were provided by Weinkauff, Monterosato, Carus, among others.

General collecting procedures, techniques and gears are used to sample mollusks in the Mediterranean (towed nets to sample planktonic species, fishing gears to sample cephalopods, bottom sledges, dredges, grabs, box samplers or corers to collect members of the infauna, scuba diving sampling for littoral species living of rocky bottoms, and many other specialized methods). A useful and rapid method to give preliminary background information of the mollusks’ diversity of a particular area is the study of small samples of bioclastic sediments (a sediment type composed of fragments of organic skeletal materials and shells of micromollusks). Empty shells are a biodiversity indicator that points the difficulty of estimating the real magnitude of species richness for taxonomical groups that do not have *post mortem* remains, such as flatworms, polychaetes, meiofauna, or peracarid crustaceans [96]. One small sample of bioclastic sediment in the Mediterranean may contain more than 100 species of mollusks [97].

In the Mediterranean, 2113 species of marine mollusks are known. Table S14 shows the completed species list of mollusks, including cephalopods, based on Sabelli et al. [98] and Bello [99] and updated from CLEMAM database (Check List of the European Marine Mollusca: http://www.somali.asso.fr/clemam/index). The class Gastropoda is the one with higher number of species (74% of all known species in the Mediterranean), followed by Bivalvia (19%), Cephalopoda (3%), Polyplacophora (1.5%), Solenogastres (1.4%), Scaphopoda (0.7%), Aplacophora (0.4%), and Monoplacophora (0.05%). In general, mollusks show a decreasing biodiversity from the west to the east, with about 45% of the species in the eastern basin (Table 2 shows data regarding cephalopods and gastropods). Two main ‘hot spots’ for mollusks are observed in the Alboran Sea and the area around Sicily. A mixture of Mediterranean and Atlantic species are present in the former, where Penas et al. [100] recorded up to 655 species of mollusks in a small area surrounding the Alboran Island. On the other hand, the central zone of the Mediterranean Sea around Sicily is an area with a high degree of endemic species. Moreover, the Mediterranean Sea currently hosts more than 150 exotic species of mollusks, of which about 90 forms established populations [101,102]. The bulk of the introduced species of mollusks in the Mediterranean (about 154) are species of Indo-Pacific origin, mostly as ‘Lessepsian immigrant’.

Mediterranean mollusks are highly diverse from a morphological and ecological point of view, ranging from minute wormlike interstitial animals (smaller than 1 mm) to giant squids (*Architeuthis*), and from minute snails (0.7 mm in *Retrotortina fuscata*) to giant fan shells (up to 90 cm in *Pinna nobilis*). Within gastropods (the most diverse class of mollusks) almost 35% of the species have an adult size smaller than 5 mm (micro-mollusks). By contrast, species larger than 50 mm account only less than 3%. Nevertheless, faunal surveys and inventories have a tendency to focus on the large species of ‘seashells’ and neglect the smaller species.

While members of most classes of mollusks are adapted to a particular environment or life-stile (caudofoveates, scaphopods, and most of bivalves are members of the infauna of sedimentary bottoms, monoplacophorans and polyplacophorans live on rocky surfaces, solenogastres are epifaunal members that prey upon cnidarians, cephalopods are highly specialized predators), gastropods are present in any marine environment (from hydrothermal vents to the pelagic realm), and they show great disparity in external form and behaviour. All kind of feeding habits (micrograzers, deposit feeders, herbivores, filter feeders, parasites, generalist or specialized predators, and scavengers) can be found within gastropods. All species of the order Acochlidiacea are interstitial animals, and all members of the orders Thecosomata and Gymnosomata and few others are holoplanktonic animals. Within the pelagic realm, cephalopods are the only group of mollusks that have nektonic species, reaching large adult size, are placed as predators competing with fishes. Most of the benthonic mollusks live in littoral areas or in the continental shelf (about 96% of the species) and only about 4% belong to the bathyal fauna. Some groups of mollusks evidence significant degree of endemics, for example, nearly 10% of the Mediterranean cephalopods are considered endemic or quasi-endemic, a characteristic observed in the Family Sepiolidae [99].

Although mollusks have been more intensely studied in the Mediterranean than almost in any other sea since the antiquity, an average of five to ten new marine species of mollusks are still being described each year in this sea. As an example, Penas et al. [100] recently described eight new species of gastropods near the Alboran Island. Currently, large-scale DNA sequencing provides the view of biodiversity being underestimated in all parts of the tree of life due the existence of cryptic species. Therefore, probably many more Mediterranean new species of mollusks will be recognized in the next years [e.g. 103]. Besides, many exotic species are being added to the native species because the global process of ‘bioinvasions’ is affecting notably the Mediterranean Sea, as it has been commented before.

Habitat loss and degradation is the main threat that impacts the molluscan diversity in the Mediterranean coasts, following by over-exploitation and fisheries (due to cephalopods and bivalves are important fishing resources for Mediterranean countries), pollution, introduction of new species, climate change and others. These threats are frequently cumulative and cause biotic homogenization and impoverishment. Mass mortalities have also known on some species, for instance the bivalve *Spondylus gaederopus* suffered widespread mortality in 1981 and 1982 [104] probably because a viral, bacterial or fungal infection. Besides, anchor causes notably damage the populations of the large fan shell *Pinna nobilis* [105]. In some cases, fishing periods coincide with reproductive seasons for cephalopod species, increasing the impact on their populations [106,107].

Seventeen Mediterranean species of mollusks have been included in the Anex II of the Barcelona Convention (Annex II and III) and Appendix II of the Bern Convention as worthy of protection. One of the species included in these lists is the data mussel *Lithophaga lithophaga*, because its overfishing by scuba divers causes serious damage along calcareous coasts of the Mediterranean Sea [108,109]. This boring bivalve is harvested by scuba divers, who smash the rocks with chisels or pneumatic hammers to detach the specimens from the walls into which their live. The major consequence is the removal of the biological cover (macroalgae and zoobenthos), which ranges from bare patches to complete desertification of the bottom communities [110].

**- Polychaeta**

Polychaeta are truly segmented worms belonging to the phylum Annelida, among which they represent the class with the largest number of described Mediterranean species. The Mediterranean Polychaeta fauna has always been among the most intensively studied. A wide panoply of sampling and analytical methods are involved in the study of polychaetes [111,112,113,114], while major insights in revealing cryptic species have been obtained, and more are still expected, from the use of fine morphological [e.g. 115], and molecular techniques [e.g. 116]. Based on an extensive taxonomic revision of all known European polychaetes, in which all existing literature up to 2008 has been checked and contrasted, we updated the total estimates for the Mediterranean polychaete species (see Table S16), which is then compared with the respective estimates for geographically restricted areas (western and eastern basins, and the Iberian Mediterranean coasts).

Polychaetes have a wide size range, extending from small meiofauna (less than 1 mm long) to big megafaunal (reaching about three m long) organisms, and are among the most common inhabitants of marine benthic bottoms, from shallow-waters to deep-sea and from brackish to hypersaline waters, with well-consolidated incursions into the plankton, as well as in continental environments [117]. They inhabit all types of substrata, from rocky bottoms to muddy sediments, where they may be among the most dominant organisms, both in terms of abundance and biomass, and also often in diversity [118]. Most of them are free-living, showing a variety of feeding strategies [119], but there are numerous cases of more or less specialized symbionts, from parasites to mutualists, living in association with many marine taxa, including other polychaetes [120].

There are numerous works in the Mediterranean Sea comprising different geographical ambits, which include more or less complete taxonomic compilations of polychaete species (see Table S16). A first attempt to estimate the total number of benthic Mediterranean polychaetes was done by Bellan [121] who reported 950 species. More recently, Arvanitidis et al. [122] extensively analysed the Mediterranean and Black Sea polychaete biodiversity patterns, reporting 1036 species as valid (based on a 1999 inventory). Our analysis reveals that the whole Mediterranean polychaete fauna currently includes 1122 species, grouped in 452 genera belonging to 72 families.

According to Arvanitidis et al. [122], the Mediterranean polychaetes are dominated by cosmopolitan species (more than 30% of the total), while the Mediterranean endemics represented 18.8%. However, the former trend (often attributed to the presence of opportunistic species), may be confusing, as many supposed cosmopolitan species revealed to have, after recent accurate morphological and/or genetic studies, more geographically restricted distributions, and the Mediterranean cosmopolites (e.g. *Haplosyllis spongicola*) are certainly not an exception [see 123]. In turn, a few Mediterranean endemics have to be removed from this category after looking at the adequate habitats in other biogeographical regions, or as a consequence of more precise taxonomic revisions. *Haplosyllis chamaeleon* (a symbiotic species associated to the gorgonian *Paramuricea clavata*, another Mediterranean endemics), has been recently found to live in association with *Paramuricea grayii* in the Atlantic coasts of Galicia, NE Iberian Peninsula [123], while *Acanthicolepis costeaui* was shown to be a junior synonym of *Acanthicolepis asperrima*, a species with a wider distribution along the European Atlantic coast [124]. Thus, the number of species known only from the Mediterranean Sea may be currently estimated as 210, taking into account that it is not yet possible to assess how many of them are truly Mediterranean endemics.

The western Mediterranean is the richest basin for polychaetes, and most likely the best studied too (Table 2). It is harbouring 85% of the species. The Central Basin, Adriatic Sea and Aegean Sea harboured a 50%, respectively, while the Levantine Basin harboured less than 45%. A total of 946 polychaete species are known from the western Mediterranean, 877 (78%) from the eastern Mediterranean, and 601 (54%) from the Iberian Mediterranean coasts. Therefore, the western basin is a 6% richer than the eastern one, but we have to consider the spectacular increase of the diversity of the polychaete fauna from the eastern basin lately, probably associated with the increasing number of studies in this biogeographic region. The Iberian Mediterranean has a reduced set of species, but its variation in taxonomic distinctness (283.4), shows a significant departure (p = 0.08) from the upper limit of the simulated distribution [125,126,127], indicating that some higher taxa include more species than those expected at random. A possible explanation may be the recent faunistic efforts concentrated in a few families carried out within the frame of the Fauna Iberica project [128,129].

New species are still being described either as a result of studying common habitats using new approaches [e.g. 130], or unexplored environments or regions in any way [e.g. 114], while an additional source of diversity may be invasive species [131] including Lessepsian migrants [e.g. 132], which were estimated to be around 6-7% the known Mediterranean species (i.e. 70 - 80 known invaders nowadays). The same occurs with a significant part of the southern coasts, and major advances in terms of the knowledge of the diversity of the group may be expected from the exploration of these regions. The average number of polychaete species that have been newly described from Mediterranean waters is 3.8 per year, since the first one described by Linné [133]. This rhythm of descriptions was almost four times higher (12.4 new species per year) during the 1860s, with the concurrent works of R. E. Claparède, A. E. Grube and E. H. Ehlers, among others, and was almost twice as high (5.7 new species per year) during the late 1960’s and early 1970’s. In the last five years (2005-2009), there has been an average of 4.2 newly described Mediterranean polychaete species per year (Figure 13a). Although the deep-sea Mediterranean bottoms seem to be less rich that those from the nearby seas (e.g. the Atlantic ones), they are still largely unexplored. The same occurs with a significant part of the southern coasts, both of the western and eastern basins. Major advances in terms of the knowledge of the diversity of the group may be expected from the exploration of these regions.

Major threats to polychaete diversity in the Mediterranean Sea may be linked to the increasing anthropogenization of the coasts, the global increase of temperatures, changes in the water quality (e.g. acidification, turbidity), overfishing, or the presence of introduced/invasive species, sometimes in a massive way like in the case of the reef-building serpulid *Ficopomatus enigmaticus* [134]. Polychaetes can also play a harmful role as fouling organisms in ship-hulls or harbours and other marine structures, as pests in natural and cultured oyster populations, or as introduced species, with the subsequent economical losses [e.g. 135].

Moreover, some polychaetes are eaten by humans, mainly those known as “palolo” worms [136], while others have been traditionally used in local pharmacopoeia [e.g. 137]. The presence of toxins or venom glands in some groups (e.g, Amphionomidae, *Glycera*, *Metaxypsamma*), and the fact that other groups are chemically defended [e.g. 138], opens the possibility of new investigations and applications in pharmacology and medicine. Polychaetes have also a significant economical relevance, as revealed by the growing commercial activities and the international market for polychaete species that are dug up or farmed, mainly for being used as fishing bait and as a food item in aquaculture, with the implied risk of introducing foreign species and associated pathogens or other non-native organisms in the wild [e.g. 139,140]. Threats to polychaete diversity may be also linked to overfishing. However, polychaetes are ubiquitous and, unless major perturbations occur, the only expected consequences would be the replacement of some species by others locally or regionally. An exception may be species associated with restricted habitats, like coastal lagoons, or with specialized life habits, like the symbiotic *Ichthyotomus sanguinarius* [an external parasite of eels never found again since its original description, 141].

**- Arthropoda**

This phylum is highly diverse and species can be found all along the Mediterranean Sea and among the most common habitats. The estimation of total number of species is difficult because species vary enormously in size and habitats, as well as they go through metamorphic changes that have confused taxonomists for centuries. Information for this synthesis came from several sources (Table 1 and Table S1). Crustaceans are the dominant group in terms of biodiversity and they include some commercial groups that have been better studied (such as decapods or stomatopods). Estimates for several arthropod groups of the Mediterranean Sea have been listed in regional lists along the basins, and several checklists exist (Table 1 and Table S1). Here we summarize main species estimates and we revised in detail the estimates available for i) cumaceans, ii) mysidaceans and iii) decapods.

A total of 2244 species of crustaceans have been registered so far in the Mediterranean Sea (Table 1 and Table S1). Cumaceans and mysidaceans are important groups belonging to the suprabenthos communities. Studies in the western Mediterranean showed that they can comprise from 42% of total suprabenthos in deep waters [862-1808 m, 163] to 61.8% in shallower areas [1-3 m, 164]. A total of 871 species are listed here to be the principal components of the Mediterranean suprabenthos communities, including Cumacea, Mysida and Lophogastrida, Isopoda, Amphipoda, Tanaidacea, and Euhpausiacea.

**(i) Cumaceans:** Accurately sampling the near-bottom invertebrate swimming species (in the suprabenthic habitat) is a difficult task due to the size of the species and their behaviour. Cumaceans, for example, were considered during long time larval stages of other crustaceans. Adult cumaceans range from 1.5 to 35 mm total length but they grow into small development instars before reaching the adult form. Fractions of a same population are lost if they are sampled or sieved with standard mesh size such in studies of macrofauna [142]. Cumaceans live in the water-sediment interface, they burrow into the sediment, and some littoral species migrate to the water surface during night-time [143]. Studies to quantify their biodiversity use dredges, and when densities are low, as frequently it happens in deep water, suprabenthic or epibenthic sledges that collect animals from the nearest sea floor water layer are used.

It was only in 1870 when cumaceans were recognized as an independent order. Since then, the knowledge of this crustacean group has grown slowly and taxonomic effort has varied greatly among biogeographical regions. The Mediterranean Sea, together with the northeast Atlantic Ocean, is likely one of the best-studied regions in the world. Our estimates show that cumaceans from the Mediterranean are composed of 99 species (Table S17). Six of the eight cumacean families are present in the Mediterranean Sea, lacking the family Gynodiastylidae, distributed in the southern Hemisphere and the family Ceratocumidae, the species of which are restricted to deep waters. Nannastacidae is the most specious family (28 species), followed by Bodotriidae and Diastylidae (27 and 23 species respectively). Leuconidae is represented by 13 species while Lampropidae and Pseudocumatidae have only four species each. The level of endemics is relatively high reaching to 32.3% for the whole Mediterranean Sea. In addition to the still unknown cumaceans Mediterranean species, it is also very probable that other foreigners could invade this sea. This fact has been already observed in the Levantine Sea where a Red Sea species was recently found [165].

In the NW Mediterranean there are 78 cumacean species known that account the 91.8% of those recorded for the western basin, and the 78.8% of those in the whole Mediterranean Sea. Conversely, in the Tunisian Plateau/Gulf of Sintra and the Adriatic Sea only few species have been recorded (four and 13, respectively). A total of 85 species are recorded in the western basin *versus* 74 in the eastern (Table S18). However, high differences in species richness observed in the four regions considered of the western basin are mostly due to the different taxonomical effort instead to an actual difference between the fauna of these seas. Both basins have 15 endemic species in common, while there are 11 endemic species exclusives from the western basin, and seven from the eastern. In terms of percentage, endemics are very similar in both basins (30.6% in the western and 29.7% in the eastern).

The taxonomic effort to describe cumaceans diversity varied also greatly among regions and areas such as the Adriatic Sea or the Gulf of Gabés are practically unknown. The accumulation curve of cumaceans species discovered (described or first recorded) in the Mediterranean Sea (Figure 13b) shows that no asymptote has been reached so far, and therefore, there has been no slowing in the rate of discovery of Mediterranean cumaceans since late 19^th^ century. This represents an annual increase of about 0.5% of the known Mediterranean cumaceans. This rate of discovery, despite it seems low, is very similar to observed in most groups of crustaceans [172].

Human activities manly focused on littoral waters such as fishing, coastal engineering, sandy beach restoration, fish farming, etc., are affecting and will affect the area where cumacean diversity and level of endemics of these crustaceans are highest. Cumaceans, and especially those living in shallowest bottoms, are very sensitive with the mean size grain of the sediment [173] and small changes in its composition may lead to the disappearance of certain species. Changes in the cumacean assemblage structure were already observed in the Spanish coast induced by massive influxes of waste water and sludge discharges [174]. Increment in shipping, as well as in water temperature because the global warming, will favour the establishment of aliens that could threaten the autochthonous cumaceans fauna.

**(ii) Mysidaceans:** They are regularly represented in the Mediterranean Sea, from the Alboran Sea to the Marmara Sea and from the coastal lagoons and beaches to the bathyal environments. The majority of the species live as well in the suprabenthic habitat, but some can be found in hard bottoms (or caves) or performing vertical migrations between the bottom and the surface, mainly at night. The studies of Mediterranean mysidacean began in 1837, when the first two species were described by Milne-Edwards. During the decades of 1860-1941 several species were described as a result of intensive sampling by diverse authors (G.O. Sars, W. Tattersall, V. Czerniavsky, M. Bacescu). From Bacescu [144] contribution, the knowledge of the mysidacean biodiversity has grown regularly until now.

Several methods are used to sample the majority of lophogastrid and mysid species [145,146]. The majority of world species [74%, 147] live in the suprabenthic habitat (or hyperbenthic, composed of near-bottom swimming species), which need specific gears to sample. The most efficient mysidacean samplers, and in general for suprabenthos, are nets mounted on suprabenthic sledges that are towed over the surface of the sediment [148,149]. The choice of a suprabenthos sampling equipment depends largely on local conditions (e.g. depth of the samples, size of the ship, power and capabilities of the lifting gears, bottom conditions, etc.). Coastal mysids, especially in hard bottoms (or caves) can also be sampled by Scuba using diver-operated specially-designed suction bottles or plankton nets. Many mysidaceans species exhibit pelagic phases and perform vertical migrations between the bottom and the surface, mainly at night. Pelagic samples can be taken with plankton nets (such as rectangular mid-water trawl, obliquely Tucker trawl, or vertically plummet net). However, coastal and oceanic strictly pelagic species are relatively few in number.

Mediterranean mysidaceans are composed of 38 genera and 102 species. Four of the seven mysidacean families are present in the Mediterranean Sea, lacking the family Petalophthalmidae (order Mysida) whose species are restricted to deep waters. The two families of the order Stygiomysida are a small group of cavernicoulus mysids endemic of caves in Central America and Mediterranean fresh waters caves but without known representation in the Mediterranean marine environment. A complete list of genera and species of the Orders Lophogastrida and Mysida at present known from the Mediterranean marine waters, their geographical distribution, and their habitat based on current information are provided in Tables S19-20. The Mediterranean mysidacean fauna consist of 37 endemic species, in addition to 48 species that also occur in the north-eastern Atlantic, and 18 cosmopolitan species. At present there are few invasive species in the Mediterranean Sea: the Ponto-Caspian mysid *Hemimysis anomala,* and the Atlantic species *Neomysis integer*, both recently detected and confirmed, respectively, in the estuary of the Grand Rhône [166]. There is a decrease in mysidacean species richness from the west to the east basins (90 known species in the western basin *versus* 55 in the eastern) (Figure S3). Isopoda, Cirripeda, Amphipoda and Decapoda also show a general higher species richness in the western basin (Table 2).

The mysidacean fauna of the Mediterranean Sea is also considered one of the best known faunas of the world [145]. However, while the marine mysid fauna of the north-western Mediterranean and the Tyrrhenian Sea (especially the Gulf of Naples) are the best known, various parts of the African coasts and of the eastern sector of the Mediterranean Sea have been little or no studied (Figure S3). The temporal trend of new species descriptions show a climbing curve that suggest that there is still a large number of unknown species to describe in this sea (Figure 13c).

The impact of anthropogenic activities in the Mediterranean mysidacean is poorly documented. There are some evidences of significant changes in the mysidacean fauna in areas under strong anthropogenic pressure, thus most Mediterranean coasts. In some areas of the north-western basin there are indications of species substitutions of the genus *Hemimysis,* probably caused by global climate warming [175]. In the gulf of Naples, as a result of the eutrophication of waters and other anthropogenic coastal pressures, local population extinctions or strong area regressions have been described [176]. Unfortunately, many of the supposed new species could likely become extinct before we even know of their existence [147,176]. Biodiversity of Mediterranean lophogastrids and mysids is therefore immersed in a critical point of knowledge and scientists must work against time with the aim of not loosing such valuable biological information.

**(iii) Decapods:** regarding their diversity, several early regional studies included substantial information [e.g. 150,151,152,153,154,155,156,157]. The first treatise on the whole Mediterranean decapod fauna was published by Heller [158] and listed 154 marine species. In the 20^th^ century important monographs updated the knowledge of regional faunas [159,160,161]. In the last twenty years our knowledge of the decapod fauna has progressed further, often as a by-product of the fishery surveys carried out to monitor the status of Mediterranean fishery resources that include high valued shrimps. Currently, there is a complete compilation on decapods of the Mediterranean Sea and north east Atlantic Ocean [162].

Overall 383 species, of those 309 autochthonous species in 63 families, have been reported in the Mediterranean Sea for decapods (against 480 species in 79 families in the Ibero-Mauritanian sectors of the Atlantic Ocean). The present autochthonous Mediterranean decapod fauna mirrors that of the temperate east Atlantic, even if it is significantly impoverished. This is due to the main connection of the Mediterranean to the eastern Atlantic Ocean through the Gibraltar Strait and that Mediterranean decapod fauna, after the Messinian salinity crisis, was rebuilt mainly from the east Atlantic stock of species. Forty Mediterranean autochthonous decapod species are endemic, and 12 of these are still known only for the type locality and the original description (Table S21). The cut of the Suez Canal in 1869 has restored the connection with the Indian Ocean, and since then, we have witnessed an exponential increment in the number of Indo-pacific decapod species recorded in the eastern Mediterranean [167]. Some of them have become locally valuable fishery resources [168], others are deemed responsible for the reduction of the populations of some autochthonous species [169]. The arrival of a large number of Lessepsian immigrants in the Levantine Sea has significantly modified the biodiversity of its decapod fauna. Currently, the Mediterranean is hosting 74 non indigenous decapod species. The autochthonous decapod species recorded in the Levantine Sea are only 60% of those reported for the whole Mediterranean Sea, even if the intensified research effort in the Aegean and Levantine Seas in the last thirty years has proved the species richness of the autochthonous decapods fauna is higher than previously supposed.

Even if decapods of the Mediterranean Sea are also regarded among the best known, new taxa continue to be discovered as a result of revisions of “difficult” genera, as *Anapagurus* by García-Gómez [170], or of the use of more appropriate techniques to collect burrowing species that are seldom obtained with traditional sampling gears [171].

Multiple anthropogenic causes, such as over-exploitation of fishery resources, coastal pollution, maritime traffic, etc., threaten the Mediterranean decapod fauna. The joint research effort of taxonomists and ecologists may help to mitigate the losses of the original Mediterranean biodiversity. Global warming may in the next future significantly affect both the distribution of these species living in coastal waters and the arrival of new Lessepsian migrants in the eastern basin. Actually a small population of *Ocypode cursor* has already been recorded on a Sicilian beach and the stock of *Crangon crangon*, a fishery commodity, in the northern Adriatic collapsed in the last twenty years. The dramatic decrease of this boreal species has been in parallel by the increment of the penaeid shrimp *Melicertus kerathurus* a thermophilic species unknown in the area until a century ago [see 158,159]. The changes we witness may be explained also by other factors that act synergistically, and highlight the importance of a continuous monitoring of the biodiversity in the Mediterranean Sea.

**- Bryozoa**

Species of this phylum are abundant and diverse in the sea bottom of the Mediterranean Sea, mainly on hard substrata. However, due to its comparatively small size, they are very often overlooked or misidentified. Bryozoans live in different substratum on which the colonies are settled: rocks, algae or other animals like mollusk shells or corals. Few species live on soft bottoms, where they usually settle on small hard pieces of substratum (small rocks or shells) that lie on the bottom. The relatively well studied western European coast of the Mediterranean has produced an abundant bibliography (Table S22).

Bryozoans have been sampled by extraction of the substratum on which the colonies are settled: rocks, algae or other animals like mollusk shells or corals, because the detaching of the colonies often destroy or severely damage them to the point of becoming unrecognizable. The observation and annotation of colonies without extraction, directly or by photography, is suitable for middle to large sized colonies of well known and easily recognizable species, which are not the most. Therefore, the sampling by trawls or dredges that do not include hard substrata tends to underestimate the bryozoans diversity of an area. Most species are determined in the laboratory with a stereomicroscope, because their usually well developed calcareous skeleton is very distinctive, but the use of a Scanning Electron Microscope (SEM) is necessary in many cases due to the small size of the zooids and the overall similarity of some species.

A total of 389 species of bryozoans have been cited in the Mediterranean and 88 species (23%) are endemic (Table S22). Of these ones, 53 species belong to the order Cyclostomata (with 17 endemic species), 44 species belong to Ctenostomata (with five endemic species), and 292 species are of the order Cheilostomata (with 66 endemic species). However, some citations may be doubtful and the taxonomic status of some of the species cited may need updating. In addition, the qualification of endemic is provisional, given that the neighbouring Atlantic waters and a great part of the Mediterranean ones are not well studied.

The most extensive works about Spanish bryozoan are those by Zabala [177] and Zabala and Maluquer [178], which, although mainly devoted to Catalonia, suppose a revision and updating of most previous information about the Mediterranean bryozoan. The Straits of Gibraltar and Alboran Sea have been studied in the last 20 years by several authors [179,180,181,182,183,184,185,186,187,188,189,190]. The French coasts are also well studied, with continuous new descriptions since the 19^th^ century [191,192,193,194,195,196,197]. The Italian coast has been less studied but there is some noticeable work [198,199,200]. Moreover, the bryozoans of the Adriatic Sea have been recently revised [201]. On the contrary, the African coasts have been scarcely studied, although some works, mainly from French authors were done along these coasts in the first half of the 20^th^ century [e.g. 202]. The eastern basin has been poorly studied as well, except from some occasional works [203,204].

However, not only a geographical bias exists, but also a taxonomic one, which is not exclusive of the Mediterranean Sea, but extensive to the general studies about bryozoans. The order Cheilostomata is the best studied due to it is the most diverse one, with about 80% of the known species, and species are easier to identify due to their high polymorphy of zooids, which are modified for different uses, and differ greatly between species even when they are closely related. On the contrary, the other two marine orders, Ctenostomata and Cyclostomata, have technical difficulties that make them harder to identify. Ctenostomata species are soft and give few external characters. Cyclostomata are well calcified, but the colonies are usually very small and cryptic, and often, if they are not reproductive, they cannot be morphologically identified because some distinctive characters occur only in the gonozooid, which is not present in young specimens or no reproductive colonies. The works on these two orders are thus very valuable, like the worldwide revision of Ctenostomata [205] and the works on Cyclostomes [185,187,188,195]. In addition, some general works such as Zabala [177], Zabala and Maluquer [178], Álvarez [186]; Álvarez [189], and Hayward and McKinney [201] include interesting revisions.

Little is known about threats to bryozoans populations in the Mediterranean Sea because many species have been seldom found. The cryptic habitats and small size of most colonies make an evaluation of risks difficult. But they share the fate of their habitats and where the coast is environmentally degraded, the populations of bryozoans may suffer the same effect. A case of massive mortality of invertebrates, including bryozoans, has been documented in the Provence related with a month of high temperature [206]. Besides, some species (i.e. *Pentapora fascialis*, *Retepora* spp) are also impacted by diver frequentation [e.g. 207]. However, there are also some species that are favoured by human activities, mainly the fouling ones like *Bugula neritina*, *Schizoporella errata* or *Watersipora subovoidea*. These and other ones are usually intertidal or shalow sublitoral species that may settle on artificial substrata and be dispersed by ships and debris. *Bugula neritina*, for instance, is a currently cosmopolitan species of Indo-Pacific origin which was spread by ships during the 19^th^ and 20^th^ centuries, and is now among the most abundant bryozoans in European and Mediterranean shallow-waters, especially well installed in harbours. One species of bryozoan, *Hornera lichenoides* is included in the Annex II of the Barcelona Convention.

**- Echinodermata**

A detailed revision of the relevant literature was carried out and information regarding the taxonomy and geographical distribution of the Mediterranean species of echinoderms was collected (Text S4). Tortonese [208] and Koukouras et al. [209] reviewed the Mediterranean echinoderm fauna. Since then, some additional information on the taxonomy and the geographical distribution of the Mediterranean echinoderm species has been published [210,211,212,213,214]. We also used the data collected from 190 stations in the Aegean Sea and Cyprus (0-1,250 m depth). Samples were obtained using fishing nets, dredges, grabs and by free or SCUBA diving. All echinoderm specimens were identified to species level and deposited at the Museum of the Department of Zoology, Aristotle University of Thessaloniki (Greece). Based on the literature and sampling data we updated the available checklist and their general distribution in the Mediterranean Sea.

Tortonese [208] reviewed the Mediterranean echinoderm fauna and reported 143 species (five Crinoidea, 30 Asteroidea, 34 Ophiuroidea, 26 Echinoidea and 48 Holothuroidea). Koukouras et al. [209], based on new information, raised the number and updated their distribution in the Mediterranean Sea. We estimated that the echinoderm fauna of the Mediterranean Sea is currently composed of 154 valid species (five Crinoidea, 33 Asteroidea, 34 Ophiuroidea, 28 Echinoidea, 54 Holothuroidea) (Table S23 provides with information on their depth distribution and detailed references). Species richness in each one of the main geographical areas of the Mediterranean Sea is given in Table 2 and Figure S4. Differences between areas can be discussed in terms of water masses and circulation [215,216] along with data on temperature and salinity variations [217], and geographical aspects [218,219]. Most of the Mediterranean echinoderm species (67.7% of the total Mediterranean species number) have an Atlantic-Mediterranean distribution, while 37 species (24%) are Mediterranean endemics. The echinoderm fauna of the Levantine is enriched by 5 Lessepsian migrant species, one of which *Synaptula reciprocans* has expanded its distribution in the Aegean Sea (Figure S4 and S5).

Echinoderms richness showed that the western Mediterranean hosted 93.5% of the known Mediterranean species and displayed the highest species richness among all other areas (Figure S5). The Central Mediterranean came fourth in echinoderm species richness among the Mediterranean areas (63.6%). However, it should have had a higher species number compared to the Aegean Sea and the Adriatic due to its direct neighbouring with the western Mediterranean. The rather low number of echinoderm species from this area is attributed to the limited sampling effort. The Adriatic Sea hosts 101 echinoderm species (65.5%). The Aegean Sea, although more distant from Gibraltar, displayed a higher echinoderm species richness in relation to the Adriatic and the Central Mediterranean (69.4% of the species). The Levantine Basin displayed the lowest richness (47.4% of the species).

In general terms, the Mediterranean echinoderm fauna is well studied, however there is a lack of relevant information from the southern Mediterranean coast due to less intensive sampling effort [219], as well as there is a lack of knowledge concerning the Mediterranean deep-sea echinoderm fauna [212]. The acquisition of a concise, detailed view on the Mediterranean echinoderm fauna is often limited by certain taxonomical problems. The descriptions of certain rare endemic species, such as the ophiuroid *Pectinura vestita*, are old and incomplete and these species have not been re-collected ever since. Thus, various records of echinoderm species from the Mediterranean should be considered doubtful since the respective identifications have been carried out in the framework of benthic ecological studies and have not been checked by taxonomy experts. In addition, many records in the literature are given under older, invalid names [209].

Climate change is considered a major threat for the Mediterranean marine biodiversity. Recently, it has been demonstrated that the entrance rate of the Lessepsian decapod, mollusk and fish species in the Mediterranean has been accelerating as a result of the increase in the mean temperature of the Mediterranean waters, a reflection of the global climate change [220,221,222]. Furthermore, for the same reason, the dispersal rates of the Lessepsian species towards higher geographical latitudes are also increasing. In this context, the Lessepsian holothurians species *Synaptula reciprocans*  seems to be quickly expanding its distribution in the Mediterranean since it has been recently reported from the Dodecanese and Cyclades Islands (south Aegean Sea) [223], while till 2007 it was known up to Rhodos I. [209]. Future research may be focused to study the entrance and dispersal rates of the Lessepsian echinoderms in the Mediterranean and their potential impact on the native fauna.

Certain echinoderm species constitute an important fishery resource. The sea urchins *Paracentrotus lividus* and *Sphaerechinus granularis* can be usually found in Mediterranean fish markets since their gonads are regularly consumed. Certain holothurians, such as *Holothuria tubulosa,* are commonly used as fishing bait, while different species of asteroids and echinoids are used for decoration. However, there is no enough information about threats to echinoderm species, although they can have important ecological roles: for example, sea urchins are important in structuring the assemblages in shallow hard-substrate areas through grazing, and they may drive the transition from erect macroalgal assemblages to coralline barrens [224,225, and references therein].

**- Sipuncula**

The phylum Sipuncula is one of the minor worm phyla, closely related to annelids. The last comprehensive revision of the Mediterranean sipunculan fauna was by Pancucci-Papadopoulou et al. [226]. Since then, only a few additions have been published [227,228,229,230,231]. Here we reviewed available information to update the list of sipunculans in the Mediterranean with published and unpublished identifications.

A total of 34 species and 4 subspecies of sipunculans arranged in 9 genera and 5 families were recorded in the Mediterranean Sea (Table S24). The more ubiquitous species are *Sipunculus nudus, Golfingia elongata, G. vulgaris, Onchnesoma steenstrupii, Phascolosoma granulatum* and *Aspidosiphon muelleri.* By contrast, very rare species are *Nephasoma constricticervix, Phascolosoma perlucens, Apionsoma trichocephalus, N.* sp. cf. *flagriferum, P. turnerae* and the subspecies *G. vulgaris antonellae.* The last subspecies is endemic of the Mediterranean Sea, whereas the last species is recorded for the first time for the investigated area by using unpublished material (J.I. Saiz, personal communication, Table S24).

Cluster analysis (Figure S6) shows that Mediterranean biogeographical sectors can be placed together into two main groups with a similarity level of 61%. The smallest group includes the three Adriatic areas plus the ‘Gulf of Lyon and Ligurian Sea’ sector. By contrast, the largest group of the dendrogram comprises the remaining 6 sectors located both at the western and eastern Mediterranean. The main species responsible for this dichotomy are *N. diaphanes diaphanes, A. murinae bilobatae,* and *Thysanocardia procera,* which are well represented in the largest group of the dendrogram, whereas *G. margaritacea* is almost the only species well represented in the smallest group.

Mediterranean sipunculans represent almost the 25% of the global sipunculan diversity. This percentage is relatively low, since the large diversity of the phylum corresponds to warm shallow tropical areas [232]. At the level of families, almost all sipunculan families are represented in the Mediterranean, with the exception of Themistidae. Concerning genera, nine of the 17 genera of sipunculans are represented in the Mediterranean. Some of the absent genera are monotypic or very restricted in their global distribution to warm waters [233]. Only two exotic species have been described so far, and there is only one subspecies considered to be endemic: *Golfingia (Golfingia) vulgaris antonellae* [227].

Regarding species data by region (Table 2) we observed a latitudinal gradient from the north to the south of the Mediterranean and west to east, linked to the temperature of the water masses along the year [234]. Thus, the dendrogram obtained (Figure S6) may be reflecting a physiological barrier for sipunculans with cold *versus* warm species. In fact, *G. margaritacea* is mainly a temperate and boreal species [226] and its presence in the Mediterranean may be indicating the prevalence of colder water masses. By contrast, other termophilic species, such as *Ph. convestitum* and *A. elegans* have been proposed as Lessepian migrants [226,230]. In this way, some other rare records of *Phascolosoma* and *Apionsoma* could be further candidates to migrants.

The spatial analysis of sipunculan diversity shows that African coast is especially undersampled. This also applies to the abyssal zone (> 3,000 m) where only three single records (*N. diaphanes corrugatum,* *Ph. tuberculosum* and *A. murinae murinae*) are published [235,236].

The only reported endangered sipunculan species is *Sipunculus nudus,* which is collected massively along the Spanish littoral as bait for fishing [237].

**- Meiobenthos**

Marine sediments hold an abundance of microscopic life, the smallest of which attach to individual sand grains or live in the interstices between grains. A variety of bacteria, archaea, and protists share this habitat with minute metazoans, the meiofauna, a major component of seabed ecosystems, particularly in the deep-sea. About half of the animal phyla are represented in the meiofauna, and some (e.g., Loricifera, Kinorhyncha) are confined to it. Nematodes are typically the most numerous component, with harpacticoid copepods, and foraminiferans also important.

The study of the diversity of main species in the large meiobenthos group (mainly composed of small benthic invertebrates that that can pass through a 0.5 mm mesh but will be retained by a 32 μm mesh and live mainly in the sediment) is a difficult and time consuming tack and therefore most studies have been dealt with higher taxonomic levels [238]. The Mediterranean Sea is not an exception, and species or genus level ecological data are scarce. In this study, nematodes and benthic (Harpacticoida) copepods were investigated, since these two groups are the dominant ones in the most of the cases (Text S5). In addition, among the various meiobenthic organisms, living soft and hard shelled benthic Foraminifera are usually equally important with nematodes, and these two taxa together usually account for over 90% of the meiobenthic community [239]. We therefore include some sparse information available about Foraminifera, as well as about Gastrotricha.

Most of the early qualitative work on free-living marine nematodes in the Mediterranean was summarized by Allgen [240] and Schuurmans Stekhoven Jr [241]. Schuurmans Stekhoven Jr [242] compiled a list of all the species found to the date from the Mediterranean and reported 143 species, 106 of which were new to science. Significant work in the past provided a series of taxonomic works on nematodes, which contributed to the knowledge of nematode biodiversity [e.g. 243,244,245]. De Bovée [246] studied the nematode populations in sublittoral terrigeneous muds off Banyuls-sur-Mer during an annual cycle, and reported a much higher number of species (184). More recently, Danovaro and Gambi [247] investigated the nematode assemblages in a *Posidonia oceanica* bed of the north-western Mediterranean over an annual cycle. High diversity values were correlated with high concentration and high heterogeneity of the food sources indicating that biodiversity is closely coupled with changes in food availability. In the eastern Mediterranean, only few studies have dealt with the lower taxonomic composition of nematodes (family, genus, or species), and several are yet to be published. Wieser [248] studied the meiobenthic nematodes of the Piraeus harbour area (south Aegean Sea) and identified 44 different species of which nine were new to science. Lampadariou [249] studied the nematodes of the continental shelf of the Cretan Sea and some deep-sea areas of the north and south Aegean Sea and found approximately 280 different species, many of which were undescribed. Nematodes currently listed more than 700 species in the Mediterranean Sea [31], and the most important taxonomic papers that contributed to the knowledge of nematode biodiversity are listed in Text S5.

Regarding harpacticoid copepods, there is no clear picture on their actual biodiversity since most studies have been mainly of taxonomic nature [e.g. 250,251,252]. However, the taxonomic studies from the Mediterranean suggest that copepod diversity might actually be high and that many new species are yet to be described. Steuer [253] investigated five different stations near El Shatby in Alexandria. Mitwally and Montagna [254] also studied the harpacticoid copepods along three sandy beaches in Alexandria and found nine species, among which, two members of the Ectinosomatidae, were new to science (*Arenosetella bassantae* and *Noodtiella toukae*). In the western Mediterranean, Soyer [255] studied the populations of harpacticoid copepods on the continental shelf off the coast of Albères between t and 130 m depth and found 254 species. In a study on the harpacticoid copepod populations from the eulittoral zone of a sandy beach on Crete, Stobbe [256] found 12 species. The community was dominated by *Psammotopa phyllosetosa*. Sevastou [257] also studied the harpacticoid copepod populations from two geographically spaced sandy beaches in Crete applying a 13-month sampling design and found 96 species, which outreached by far any species richness recorded in previous studies of comparable habitats.

Until the late 1990s, the study of benthic foraminifera in the Mediterranean has been restricted to a small number of samples collected from specific sites [258,259]. More recently, a larger number of investigations have considered the importance of living benthic foraminifera in ecological studies [e.g. 260,261,262], however they are still far from being well studied. The knowledge recently gained indicated that in the Mediterranean the foraminifera are highly diverse and consist with more than 600 species. A large number of these species belong to Lessepsian invaders [11], which is however still a matter under debate since data on the biogeography, diversity and ecology of autochthonous shallow-water faunas from various carbonate environments of the Mediterranean Sea are limited [263].

In addition, the studies on marine gastrotrichs in the Mediterranean were conducted mainly through a programme of faunistic and taxonomic research of the Italian seas [264,265,266,267,268,269,270] and in Crete [271]. All these studies revealed approximately 150 species from the Mediterranean from a total of 280 and 580 which are known from Europe and world wide, respectively. Hofrichter [31] mentioned 165 species in the Mediterranean Sea.

A small number of pollution studies in the Mediterranean Sea considered the use of meiofauna as potential indicators of anthropogenic disturbance. These studies concerned mostly domestic sewage discharges, pollution in harbours, and fish farm impacts. Marcotte and Coull [272] identified copepod species from five stations placed on transect in the northern Adriatic Sea along a gradient of municipal raw sewage discharge and showed that diversity was very low near the outfall and increased with increasing distance from the source of pollution. Keller [273] investigated the meiofauna communities in a marine area which was highly polluted by the sewage outfall of Marseille. Lampadariou et al. [274] investigated the nematode and copepod community structure along a grid of seventeen stations covering an area from the innermost polluted to the outer clean area of Heraklion harbour, and showed that the nematode community showed a clear zonation according to the degree of pollution and physical disturbance from shipping activities. They concluded that, besides physical factors such as depth, the high level of organic carbon or pollutants such as copper and cadmium played an important role in structuring the nematode communities. A decline in diversity as a result of disturbance caused by fish farming activities was also reported by Mirto et al. [275] from the Gulf of Gaeta in the NW Mediterranean. *Setosabatieria*, which was the dominant genus, was found to be highly sensitive to organic disturbance as it disappeared completely three months after the deployment of cages. In contrast, other nematode genera, such as *Dorylaimopsis*, *Sabatieria*, and *Oxystomina*, proved to be tolerant and benefited from the new organically enriched conditions.

Information on other marine invertebrates is summarized in Table 1 and Table S1. Total updated registries for the other invertebrate species are of 2168 species (1393 species, excluding Arthropoda).

**- Ascidiacea**

Ascidiacea comprises the largest Class of the Subphylum Tunicata, a part of the phylum Chordata. Although most abundant at sublittoral rocky communities, they are also adapted to live in abyssal plains [276], and few species are intertidal [277]. The Mediterranean fauna is an important part of the global ascidian fauna of the Atlantic and Mediterranean coasts, which totals approximately 500 species [278]. The ascidians of the Mediterranean Sea have been explored since the second half of the 19^th^ century [e.g. 279,280]. In the first half of the 20^th^ century, important taxonomic works were published [e.g. 281,282,283] that improved the knowledge of Mediterranean ascidian diversity. Pérès [284] listed 130 species in the Mediterranean Sea (although since then some species have been synonymised or have split in several species). Later faunistic and taxonomic studies analysed the diversity of the ascidian fauna in particular areas, especially in the western Mediterranean. In this revision we build on Pérès’ work and updated the records of ascidian species in the Mediterranean Sea, as well as their distribution and affinities.

Fiala-Medioni [285] reported 77 species from the SE of France. Turon [286] found 107 species in the NE Spanish littoral. Ramos-Esplá [287], similarly, listed 117 species in the Mediterranean shores of Spain. Naranjo [288] found 84 species in the area around the Gibraltar Straits, 79 of which were present in the Mediterranean side. Koukouras et al. [289] first reviewed the long-neglected ascidian fauna of the eastern Mediterranean, providing a check-list of 86 species in this basin, out of an estimate number of 187 for the whole Mediterranean. Mastrototaro and Tursi [290] provided a check-list of the Italian fauna of ascidians, including 128 species. Aside from these authors, most recent work on ascidian diversity in the Mediterranean has been done by specialists such as Jean-Marie Pérès, Claude and Françoise Monniot, Françoise Lafargue, and Riccardo Brunetti, among others. Building on the work by Pérès [284], and exhaustively searching all posterior reports, we listed 229 ascidian species for the Mediterranean Sea. Although some of the records are dubious, we have eliminated from our list only those species that were clearly invalid or synonyms of other species (Table S25). Overall, the number of new citations incorporated to the database since 1960 is 22.4 species per decade (Figure 13d). Of these, 52 species (46.8% of additions) corresponded to newly described species, not just new reports.

From a biogeographic point of view, Pérès [284] reported a major contribution of endemic Mediterranean species (50%). The second major component consisted of species of Atlantic-Mediterranean distribution (37.7%). A further 11.5% of the species were cosmopolitan or had a circumtropical range. Only one species (*Herdmania momus*) was detected as possible Lessepsian migrant at that time. In the area close to the Straits of Gibraltar, the number of Mediterranean endemics falls to 22% of species, while those of Atlantic-Mediterranean distribution rise to 60% [278]. We currently estimated that the endemic ascidians accounted for 34.9% of the species (Table S25). The species with an Atlantic-Mediterranean distribution accounted for another 46.7% (of which 12.2% were found in the Mediterranean only in the western basin); cosmopolitan and circumtropical species made up 14.4% of the total, and nine species (3.9%) were identified as probable Lessepsian migrants. Izquierdo-Muñoz et al. [291] reviewed introduced ascidian species in the Mediterranean, listing 14 species, and Shenkar and Loya [292] reported the occurrence of seven non indigenous ascidian species in the Mediterranean coasts of Israel, many of which are most likely Lessepsian migrants. Particularly worrisome is the recent report of the clubbed tunicate *Styela clava* in the lagoon of Thau, France [293], given the problems that this species has posed to shellfish industry elsewhere [294]. Although not generally recognized as such, many forms adapted to live in man-made structures, even if they have been present for long times in the Mediterranean, are probably introduced, e.g. *Styela plicata* and *Botryllus schlosseri* [295,296].

The composition of the ascidian fauna of the Mediterranean, with species of temperate and subtropical affinities, makes it sensitive to ongoing global warming [77], and a displacement of species of cold-water affinities in favour of termophilic species [297] is expectable. The arrival of introduced ascidian species to the Mediterranean has received considerable attention in recent years. Many introduced species are confined to artificial environments, but other spread outside and colonize natural substratum, turning into invasive forms. One clear instance is the solitary ascidian *Microcosmus squamiger*, which can reach high densities and carpet natural substrates [298]. This species is now common in western Mediterranean [299], and Streftaris and Zenetos [300] listed this species (as *M. exasperatus*) within the 100 “worst” marine invasive in the Mediterranean.

The trends observed in the known distribution of ascidians from the sixties on indicate that the percentage of endemics is now considerably lower (from 50% to ca. 35%), which is partly caused by the finding of Mediterranean species in adjacent Atlantic waters [e.g. 301]. The number of species known only from one or another basin, which was high and highly skewed towards the western basin, is now much lower and more balanced between basins (Table 2). This is the result of increased knowledge of intra-Mediterranean distribution of many species and, particularly, the rise of studies performed in the eastern basin, which is compensating the previous neglect of this area [e.g. 289,290,302]. Endemic ascidians present at both basins represented 16.9% of Mediterranean ascidians, while 11.3% and 7.4% were endemics reported only in the western and eastern basin, respectively (Table S25).

Ascidian diversity is underreported due to sampling effects, the lack of taxonomists, and inherent difficulties in the taxonomy of the group. Rocky shores in the northern Mediterranean can be considered reasonably well studied, but the Mediterranean African shores still present few studies available [e.g. 303], and may be a hotspot of ascidian biodiversity. This is because this region acts as a refuge for Atlantic-Mediterranean species of tropical affinities, while the northern region of the Mediterranean harbours species with temperate and even boreal affinities. Moreover, much remains to be known about the ascidian fauna in deep waters, or in soft-bottom communities. The ascidians themselves pose important problems for identification, due to the lack of distinctive features in some groups, overlapping of characters between species and, particularly, because observation of characters requires specimens adequately preserved in a relaxed state and ripe, which are hardly available. It is a group where molecular tools can substantially contribute to help taxonomic work. Available studies that have incorporated genetic markers to the research on Mediterranean ascidians showed that cryptic speciation may be much commoner than usually recognized [e.g. 304,305,306].

Ascidians are subjected to different threats such as habitat destruction, degradation, and pollution, as well as global warming, arrival of invasive species, exploitation and other factors [e.g. 307,308]. Mass mortalities have also occurred at some occasions; for instance, an undescribed illness devastated the populations of *Microcosmus sabatieri* in the early 1990s at least along Spanish and French shores. This species was once the most abundant solitary form in the shallow sublittoral and reached abundances of approximately one individual per square meter in NE Spain (X. Turon, personal communication). It is nowadays extremely rare. This is significant because in the Mediterranean, large pyurid species such as *M. polymorphus*, *M. sabatieri* and *M. vulgaris* have been consumed since the 1^st^ century AD [309]. *M. sabatieri* is still abundant in the eastern Mediterranean, where it sustains a small-scale fishery [309,310]. Threats to ascidian biodiversity have also biotechnological implications, as ascidians are producers of some of the most promising anticancer compounds found to date in marine invertebrates [311]. They have originated one drug already marketed (Trabectedin, sold under the brand Yondelis®), obtained from *Ecteinascidia turbinata*, a circumtropical species that used to be farmed in the Balearic Islands before a synthetic production of the drug was achieved in the 1990s). A second compound, Aplidin®, obtained from the Mediterranean species *Aplidium albicans* is in advanced clinical trials.

**References**

## **Mediterranean AquaMaps (by Kristin Kaschner, Kathy Kesner-Reyes, Josephine Barile & Elijah Laxamana)**

## **Text S2. Mediterranean AquaMaps specifications**

AquaMaps model represents a modified version of the relative environmental suitability model (RES) developed by Kaschner et al. [1]. It is an environmental envelope model that generates standardized range maps and the relative probability of occurrence within that range for marine species based on the environmental conditions in each 0.5 degree latitude by 0.5 degree longitude cell of global grid. The model was developed specifically to deal with the sampling biases, such as heterogeneous sampling effort and species misidentifications, affecting most of the large-scale data sets that are currently available for species distribution modelling in the marine realm [2,3]. The model currently includes more than 9,000 fishes, marine mammals, reptiles and invertebrate species. Predictions are generated based on species-specific environmental tolerances computed using available point occurrences, which are obtained from online databases such as OBIS, or the Global Biodiversity Information Facility [4], but which are then supplemented with other types of habitat usage information obtained directly from online species databases such as FishBase and SeaLifeBase [2,3]. Moreover, an expert-review function in the AquaMaps algorithm explicitly allows for the incorporation of expert knowledge about species occurrence to counteract or compensate known sampling biases. AquaMaps outputs have been successfully validated using independent, effort-corrected survey data and, in the face of the existing sub-optimal input data sets, AquaMaps model performance compares well with that of other presence-only habitat prediction models, such as GARP, Maxent or GAMs [3].

Species richness maps for the Mediterranean Sea were generated by superimposing generated range maps of all individual species and then counting the number of species predicted to be present in each half degree cell.

A summary of the number of species, by different taxa, which are known to occur in and/or are endemic to the Mediterranean Sea based on the data provided by online species databases such as FishBase and SeaLifeBase can be found in Table S3 and Table S4. The appendix also shows the proportion of species currently covered by AquaMaps. Based on the comparison with FishBase and SeaLifeBase data, AquaMaps coverage of higher vertebrate and fish taxa is either complete or relatively comprehensive (Table S5-S6). In contrast, most invertebrate groups are much less well represented and other groups, such as plants, fungi, bacteria, but also seabirds, have not yet been incorporated at all. Of those species covered by AquaMaps and included in this analysis, more than half are ray-finned fishes (Actinopterygii), and roughly a third are invertebrates, and only a very small percentage are higher vertebrates such as cetaceans, pinnipeds or marine turtles. Based on the information available from FishBase and SeaLifeBase, only a relatively small proportion of all species that are native in the Mediterranean Sea are also endemic. At the moment, most AquaMaps available for the Mediterranean species represent the computer-generated default maps, which have not been checked explicitly for consistency with published range maps or fully reviewed by experts. In general, though, a relative high proportion of species in taxa that are often the focus of conservation efforts, such as marine mammals, marine turtles and elasmobranch, have already been reviewed. On average, approximately 16% of all species included in this analysis are listed by the IUCN as endangered, vulnerable or threatened, thus requiring special protection and conservation measures. However, while less than one percent of the ray-finned fishes is considered endangered, 30% and more of the Mediterranean marine mammal species and elasmobranches, and all marine turtles and Holocephali species occurring in this ocean basin are threatened or endangered.

The World Register of Marine Species (WoRMS) lists 8,470 species records for the Mediterranean Sea [5] which is surpassed by an even higher estimate of up to 17,000 marine Mediterranean species based on the information provided in the main part of this paper. Thus, the approximately 700 Mediterranean species currently covered by AquaMaps, which formed the basis for our biodiversity maps, represent only a fraction of all species known to occur in this ocean basin. However, the species list provided by WoRMS also includes all non-Animalia species that are, at the moment, mostly not represented by any of the existing online species databases and for which there are mostly few if any occurrence records available [6]. In terms of predicted patterns of species richness, AquaMaps outputs are probably relatively representative for fishes and marine mammals, although increasing the number of expert-reviews for Mediterranean Actinopterygii species would increase confidence in biodiversity maps for this taxonomic group. Similarly, while the selected presence threshold and the resulting species richness predictions for marine mammals have successfully been validated [7], this type of analysis still remains to be carried out for other taxa. For most taxa, the maps showing biodiversity patterns of endangered species in the Mediterranean Sea, however, can be regarded as reliable and representative, since more than 70% of all underlying species maps have been reviewed by experts.

**References**

## **Table S3. Mediterranean native and endemic marine species reported by FishBase/SeaLifeBase by different taxa and current extent of coverage of AquaMaps**

|  | **Native and Endemic Species Reported from the Mediterranean Sea** | | | | |  |  |  |  |  |
| --- | --- | --- | --- | --- | --- | --- | --- | --- | --- | --- |
|  |  | **Native** | | | **Endemic** | | | **Native & Endemic** | | |
|  |  | FB/SLB | AquaMaps | % | FB/SLB | AquaMaps | % | FB/SLB | AquaMaps | % |
| **Fishes** | Actinopterygii | 469 | 373 | 80% | 42 | 20 | 48% | 511 | 393 | 77% |
|  | Elasmobranchii | 76 | 68 | 89% | 4 | 4 | 100% | 80 | 74 | 93% |
|  | Holocephali | 1 | 1 | 100% |  |  |  | 1 | 1 | 100% |
|  | Myxini | 1 | 1 | 100% |  |  |  | 1 | 1 | 100% |
|  | Cephalaspidomorphi | 2 | 2 | 100% |  |  |  | 2 | 2 | 100% |
|  | **Subtotal Fish** | **549** | **445** | **81%** | **46** | **24** |  | **595** | **471** | **79%** |
|  |  |  |  |  |  |  |  |  |  |  |
| **Mammals** | Balaenopteridae | 4 | 4 | 100% |  |  |  | 4 | 4 | 100% |
|  | Delphinidae | 8 | 8 | 100% |  |  |  | 8 | 8 | 100% |
|  | Kogiidae | 1 | 1 | 100% |  |  |  | 1 | 1 | 100% |
|  | Phocidae | 1 | 1 | 100% |  |  |  | 1 | 1 | 100% |
|  | Phocoenidae |  |  |  | 1 | 1 | 100% | 1 | 1 | 100% |
|  | Physeteridae | 1 | 1 | 100% |  |  |  | 1 | 1 | 100% |
|  | Ziphiidae | 4 | 4 | 100% |  |  |  | 4 | 4 | 100% |
|  | **Subtotal Mammals** | **19** | **19** | **100%** | **1** | **1** | 100% | **20** | **20** | **100%** |
|  |  |  |  |  |  |  |  |  |  |  |
|  |  |  |  |  |  |  |  |  |  |  |
| **Turtles** | Cheloniidae | 4 | 4 | 100% |  |  |  | 4 | 4 | 100% |
|  | Dermochelyidae | 1 | 1 | 100% |  |  |  | 1 | 1 | 100% |
|  | **Subtotal Turtles** | **5** | **5** | **100%** | **0** | **0** |  | **5** | **5** | **100%** |
|  |  |  |  |  |  |  |  |  |  |  |
|  |  |  |  |  |  |  |  |  |  |  |
| **Invertebrates** | Anopla | 6 |  | 0% |  |  |  | 6 | 0 | 0% |
|  | Anthozoa | 23 | 3 | 13% | 1 |  | 0% | 24 | 3 | 13% |
|  | Aplacophora | 30 |  | 0% |  |  |  | 30 | 0 | 0% |
|  | Appendicularia | 39 |  | 0% |  |  |  | 39 | 0 | 0% |
|  | Articulata | 6 | 2 | 33% |  |  |  | 6 | 2 | 33% |
|  | Ascidiacea | 141 | 22 | 16% |  |  |  | 141 | 22 | 16% |
|  | Asteroidea | 2 |  | 0% |  |  |  | 2 | 0 | 0% |
|  | Bivalvia | 311 | 62 | 20% | 1 |  | 0% | 312 | 62 | 20% |
|  | Bryopsidophyceae | 2 |  | 0% |  |  |  | 2 | 0 | 0% |
|  | Calcarea | 33 |  | 0% |  |  |  | 33 | 0 | 0% |
|  | Cephalopoda | 39 | 15 | 38% | 2 |  | 0% | 41 | 15 | 37% |
|  | Demospongiae | 22 | 1 | 5% |  |  |  | 22 | 1 | 5% |
|  | Echinoidea | 3 |  | 0% |  |  |  | 3 | 0 | 0% |
|  | Enopla | 4 |  | 0% |  |  |  | 4 | 0 | 0% |
|  | Enteropneusta | 7 |  | 0% |  |  |  | 7 | 0 | 0% |
|  | Eoacanthocephala | 1 |  | 0% |  |  |  | 1 | 0 | 0% |
|  | Gastropoda | 233 | 11 | 5% |  |  |  | 233 | 11 | 5% |
|  | Gymnolaemata | 69 | 4 | 6% |  |  |  | 69 | 4 | 6% |
|  | Holothuroidea | 4 | 2 | 50% |  |  |  | 4 | 2 | 50% |
|  | Hydrozoa | 196 | 4 | 2% | 10 |  | 0% | 206 | 4 | 2% |
|  | Inarticulata | 1 |  | 0% |  |  |  | 1 | 0 | 0% |
|  | Kinorhyncha | 19 |  | 0% |  |  |  | 19 | 0 | 0% |
|  | Loricifera | 2 |  | 0% |  |  |  | 2 | 0 | 0% |
|  | Malacostraca | 209 | 33 | 16% |  |  |  | 209 | 33 | 16% |
|  | Maxillopoda | 32 | 5 | 16% |  |  |  | 32 | 5 | 16% |
|  | Nematomorpha | 1 |  | 0% |  |  |  | 1 | 0 | 0% |
|  | Not assigned | 213 |  | 0% |  |  |  | 213 | 0 | 0% |
|  | Nuda | 3 |  | 0% |  |  |  | 3 | 0 | 0% |
|  | Orthonectida | 1 |  | 0% |  |  |  | 1 | 0 | 0% |
|  | Ostracoda | 2 |  | 0% |  |  |  | 2 | 0 | 0% |
|  | Palaeacanthocephala | 6 |  | 0% |  |  |  | 6 | 0 | 0% |
|  | Phascolosomatidea | 10 | 1 | 10% |  |  |  | 10 | 1 | 10% |
|  | Pogonophora | 1 |  | 0% |  |  |  | 1 | 0 | 0% |
|  | Polychaeta | 211 | 4 | 2% |  |  |  | 211 | 4 | 2% |
|  | Polyplacophora | 14 | 3 | 21% |  |  |  | 14 | 3 | 21% |
|  | Priapulida | 3 |  | 0% |  |  |  | 3 | 0 | 0% |
|  | Pterobranchia | 1 |  | 0% |  |  |  | 1 | 0 | 0% |
|  | Pycnogonida | 26 | 2 | 8% |  |  |  | 26 | 2 | 8% |
|  | Rhombozoa | 17 |  | 0% |  |  |  | 17 | 0 | 0% |
|  | Scaphopoda | 17 | 7 | 41% |  |  |  | 17 | 7 | 41% |
|  | Scyphozoa | 17 | 1 | 6% | 1 |  | 0% | 18 | 1 | 6% |
|  | Sipunculidae | 18 | 10 | 56% |  |  |  | 18 | 10 | 56% |
|  | Stenolaemata | 3 |  | 0% |  |  |  | 3 | 0 | 0% |
|  | Tentaculata | 23 | 1 | 4% |  |  |  | 23 | 1 | 4% |
|  | Thaliacea | 23 |  | 0% |  |  |  | 23 | 0 | 0% |
|  | Trematoda | 4 |  | 0% |  |  |  | 4 | 0 | 0% |
|  | Turbellaria | 23 |  | 0% |  |  |  | 23 | 0 | 0% |
|  | Ulvophyceae | 2 |  | 0% |  |  |  | 2 | 0 | 0% |
|  | **Subtotal Inverts** | **2073** | **193** | **9%** | **15** | **0** | **0%** | **2088** | **193** | **9%** |
|  | **Total** | **2646** | **662** | **25%** | **62** | **25** | **40%** | **2708** | **689** | **25%** |

## **Table S4. Proportion of checked or expert-reviewed AquaMaps for different taxa**

|  |  | **AquaMaps** | **Expert-reviewed/ checked** | **% checked** |
| --- | --- | --- | --- | --- |
| **Fishes** | Actinopterygii | 393 | 54 | 13.74% |
|  | Elasmobranchii | 74 | 27 | 36.49% |
|  | Holocephali | 1 | 1 | 100.00% |
|  | Myxini | 1 | 0 | 0.00% |
|  | Cephalaspidomorphi | 2 | 0 | 0.00% |
|  | **Subtotal Fish** | **471** | **82** | **17.41%** |
|  |  |  |  |  |
| **Mammals** | Balaenopteridae | 4 | 3 | 75.00% |
|  | Delphinidae | 8 | 8 | 100.00% |
|  | Kogiidae | 1 | 1 | 100.00% |
|  | Phocidae | 1 | 1 | 100.00% |
|  | Phocoenidae | 1 | 1 | 100.00% |
|  | Physeteridae | 1 | 1 | 100.00% |
|  | Ziphiidae | 4 | 1 | 25.00% |
|  | **Subtotal Mammals** | **20** | **16** | **80.00%** |
|  |  |  |  |  |
| **Turtles** | Cheloniidae | 4 | 4 | 100.00% |
|  | Dermochelyidae | 1 | 1 | 100.00% |
|  | **Subtotal Turtles** | **5** | **5** | **100.00%** |
|  |  |  |  |  |
| **Invertebrates** | Anopla | 0 | 0 | 0.00% |
|  | Anthozoa | 3 | 0 | 0.00% |
|  | Aplacophora | 0 | 0 | 0.00% |
|  | Appendicularia | 0 | 0 | 0.00% |
|  | Articulata | 2 | 0 | 0.00% |
|  | Ascidiacea | 22 | 0 | 0.00% |
|  | Asteroidea | 0 | 0 | 0.00% |
|  | Bivalvia | 62 | 1 | 1.61% |
|  | Bryopsidophyceae | 0 | 0 | 0.00% |
|  | Calcarea | 0 | 0 | 0.00% |
|  | Cephalopoda | 15 | 3 | 20.00% |
|  | Demospongiae | 1 | 0 | 0.00% |
|  | Echinoidea | 0 | 0 | 0.00% |
|  | Enopla | 0 | 0 | 0.00% |
|  | Enteropneusta | 0 | 0 | 0.00% |
|  | Eoacanthocephala | 0 | 0 | 0.00% |
|  | Gastropoda | 11 | 0 | 0.00% |
|  | Gymnolaemata | 4 | 0 | 0.00% |
|  | Holothuroidea | 2 | 0 | 0.00% |
|  | Hydrozoa | 4 | 0 | 0.00% |
|  | Inarticulata | 0 | 0 | 0.00% |
|  | Kinorhyncha | 0 | 0 | 0.00% |
|  | Loricifera | 0 | 0 | 0.00% |
|  | Malacostraca | 33 | 1 | 3.03% |
|  | Maxillopoda | 5 | 0 | 0.00% |
|  | Nematomorpha | 0 | 0 | 0.00% |
|  | Not assigned | 0 | 0 | 0.00% |
|  | Nuda | 0 | 0 | 0.00% |
|  | Orthonectida | 0 | 0 | 0.00% |
|  | Ostracoda | 0 | 0 | 0.00% |
|  | Palaeacanthocephala | 0 | 0 | 0.00% |
|  | Phascolosomatidea | 1 | 0 | 0.00% |
|  | Pogonophora | 0 | 0 | 0.00% |
|  | Polychaeta | 4 | 0 | 0.00% |
|  | Polyplacophora | 3 | 0 | 0.00% |
|  | Priapulida | 0 | 0 | 0.00% |
|  | Pterobranchia | 0 | 0 | 0.00% |
|  | Pycnogonida | 2 | 0 | 0.00% |
|  | Rhombozoa | 0 | 0 | 0.00% |
|  | Scaphopoda | 7 | 0 | 0.00% |
|  | Scyphozoa | 1 | 0 | 0.00% |
|  | Sipunculidae | 10 | 0 | 0.00% |
|  | Stenolaemata | 0 | 0 | 0.00% |
|  | Tentaculata | 1 | 0 | 0.00% |
|  | Thaliacea | 0 | 0 | 0.00% |
|  | Trematoda | 0 | 0 | 0.00% |
|  | Turbellaria | 0 | 0 | 0.00% |
|  | Ulvophyceae | 0 | 0 | 0.00% |
|  | **Subtotal Invert** | **193** | **5** | **2.59%** |
|  | **Total** | **689** | **108** | **15.67%** |

## **Table S5. Mediterranean species of special conservation concern covered by AquaMaps**

|  |  | **IUCN status** | **No of species** | **% Endangered** | **AquaMaps** |
| --- | --- | --- | --- | --- | --- |
| **Fishes** | Actinopterygii | CR | 2 |  | 393 |
|  | Actinopterygii | EN | 2 |  |  |
|  | Actinopterygii | VU | 2 |  |  |
|  | Actinopterygii | NT | 1 |  |  |
|  | **Subtotal Actinopterygii** |  | 7 | 1.78% |  |
|  | Elasmobranchii | CR | 12 |  | 74 |
|  | Elasmobranchii | EN | 8 |  |  |
|  | Elasmobranchii | VU | 10 |  |  |
|  | Elasmobranchii | NT | 11 |  |  |
|  | **Subtotal Elasmobranchii** |  | 41 | 55.41% |  |
|  | Holocephali | NT | 1 | 100.00% | 1 |
|  | **Subtotal Fish** |  | **97** | **20.73%** | **468** |
|  |  |  |  |  |  |
| **Mammals** | Balaenopteridae | EN | 2 |  | 20 |
|  | Balaenopteridae | VU | 1 |  |  |
|  | Delphinidae | EN | 1 |  |  |
|  | Delphinidae | VU | 1 |  |  |
|  | Phocidae | CR | 1 |  |  |
|  | Phocoenidae | VU | 1 |  |  |
|  | Physeteridae | VU | 1 |  |  |
|  | **Subtotal Mammals** |  | **8** | **40.00%** |  |
|  |  |  |  |  |  |
| **Turtles** | Cheloniidae | EN | 2 |  | **5** |
|  | Cheloniidae | CR | 2 |  |  |
|  | Dermochelyidae | CR | 1 |  |  |
|  | **Subtotal Turtles** |  | **5** |  |  |
| **Total** |  |  | **110** | **100.00%** |  |

## **Table S6. Checklist of species included in AquaMaps of the Mediterranean Sea**

| **Phylum** | **Class** | **Order** | **Family** | **Genus** | **Species** | **Mean probability of Occurrence** | **IUCN Code** | **Expert Reviewed** |
| --- | --- | --- | --- | --- | --- | --- | --- | --- |
| Annelida | Polychaeta | Canalipalpata | Oweniidae | Owenia | fusiformis | 0.16 | NL | 0 |
| Annelida | Polychaeta | Canalipalpata | Serpulidae | Pomatoceros | triqueter | 0.45 | NL | 0 |
| Annelida | Polychaeta | Canalipalpata | Trichobranchidae | Trichobranchus | glacialis | 0.35 | NL | 0 |
| Annelida | Polychaeta | Not assigned | Capitellidae | Heteromastus | filiformis | 0.2 | NL | 0 |
| Arthropoda | Malacostraca | Amphipoda | Ampeliscidae | Ampelisca | brevicornis | 0.21 | NL | 0 |
| Arthropoda | Malacostraca | Amphipoda | Dexaminidae | Atylus | vedlomensis | 0.02 | NL | 0 |
| Arthropoda | Malacostraca | Decapoda | Aristeidae | Aristaeomorpha | foliacea | 0.28 | NL | 0 |
| Arthropoda | Malacostraca | Decapoda | Aristeidae | Aristeus | antennatus | 0.65 | NL | 0 |
| Arthropoda | Malacostraca | Decapoda | Cancridae | Cancer | pagurus | 0.05 | NL | 0 |
| Arthropoda | Malacostraca | Decapoda | Diogenidae | Dardanus | arrosor | 0.62 | NL | 0 |
| Arthropoda | Malacostraca | Decapoda | Majidae | Maja | squinado | 0.29 | NL | 0 |
| Arthropoda | Malacostraca | Decapoda | Nephropidae | Homarus | gammarus | 0.05 | NL | 1 |
| Arthropoda | Malacostraca | Decapoda | Nephropidae | Nephrops | norvegicus | 0.53 | NL | 0 |
| Arthropoda | Malacostraca | Decapoda | Palinuridae | Palinurus | elephas | 0.65 | NL | 0 |
| Arthropoda | Malacostraca | Decapoda | Pandalidae | Chlorotocus | crassicornis | 0.9 | NL | 0 |
| Arthropoda | Malacostraca | Decapoda | Pandalidae | Plesionika | martia | 0.79 | NL | 0 |
| Arthropoda | Malacostraca | Decapoda | Pasiphaeidae | Pasiphaea | multidentata | 0.19 | NL | 0 |
| Arthropoda | Malacostraca | Decapoda | Penaeidae | Funchalia | villosa | 0.61 | NL | 0 |
| Arthropoda | Malacostraca | Decapoda | Penaeidae | Funchalia | woodwardi | 0.32 | NL | 0 |
| Arthropoda | Malacostraca | Decapoda | Penaeidae | Parapenaeus | longirostris | 0.59 | NL | 0 |
| Arthropoda | Malacostraca | Decapoda | Portunidae | Callinectes | sapidus | 0.3 | NL | 0 |
| Arthropoda | Malacostraca | Decapoda | Portunidae | Carcinus | aestuarii | 0.73 | NL | 0 |
| Arthropoda | Malacostraca | Decapoda | Portunidae | Liocarcinus | depurator | 0.04 | NL | 0 |
| Arthropoda | Malacostraca | Decapoda | Portunidae | Necora | puber | 0.03 | NL | 0 |
| Arthropoda | Malacostraca | Decapoda | Sergestidae | Sergestes | atlanticus | 0.24 | NL | 0 |
| Arthropoda | Malacostraca | Decapoda | Solenoceridae | Solenocera | membranacea | 0.75 | NL | 0 |
| Arthropoda | Malacostraca | Isopoda | Anthuridae | Cyathura | carinata | 0.5 | NL | 0 |
| Arthropoda | Malacostraca | Isopoda | Cirolanidae | Natatolana | borealis | 0.23 | NL | 0 |
| Arthropoda | Malacostraca | Isopoda | Cirolanidae | Natatolana | neglecta | 0.48 | NL | 0 |
| Arthropoda | Malacostraca | Isopoda | Gnathiidae | Gnathia | vorax | 0.43 | NL | 0 |
| Arthropoda | Malacostraca | Isopoda | Idoteidae | Synisoma | capito | 0.75 | NL | 0 |
| Arthropoda | Malacostraca | Isopoda | Janiridae | Janira | maculosa | 0.19 | NL | 0 |
| Arthropoda | Malacostraca | Isopoda | Sphaeromatidae | Dynamene | torelliae | 0.54 | NL | 0 |
| Arthropoda | Malacostraca | Isopoda | Sphaeromatidae | Sphaeroma | serratum | 0.71 | NL | 0 |
| Arthropoda | Malacostraca | Mysida | Mysidae | Gastrosaccus | sanctus | 0.18 | NL | 0 |
| Arthropoda | Malacostraca | Stomatopoda | Squillidae | Squilla | mantis | 0.55 | NL | 0 |
| Arthropoda | Malacostraca | Tanaidacea | Tanaidae | Tanais | dulongii | 0.85 | NL | 0 |
| Arthropoda | Maxillopoda | Pedunculata | Scalpellidae | Scalpellum | scalpellum | 0.06 | NL | 0 |
| Arthropoda | Maxillopoda | Sessilia | Balanidae | Balanus | crenatus | 0.04 | NL | 0 |
| Arthropoda | Maxillopoda | Sessilia | Balanidae | Balanus | improvisus | 0.91 | NL | 0 |
| Arthropoda | Maxillopoda | Sessilia | Chthamalidae | Chthamalus | stellatus | 0.83 | NL | 0 |
| Arthropoda | Maxillopoda | Sessilia | Verrucidae | Verruca | stroemia | 0.01 | NL | 0 |
| Arthropoda | Pycnogonida | Pantopoda | Endeididae | Endeis | spinosa | 0.39 | NL | 0 |
| Arthropoda | Pycnogonida | Pantopoda | Phoxichilidiidae | Anoplodactylus | petiolatus | 0.14 | NL | 0 |
| Brachiopoda | Articulata | Terebratulida | Cancellothyrididae | Terebratulina | retusa | 0.43 | NL | 0 |
| Brachiopoda | Articulata | Terebratulida | Platidiidae | Platidia | anomioides | 0.21 | NL | 0 |
| Bryozoa | Gymnolaemata | Cheilostomata | Hippothoidae | Chorizopora | brongniartii | 0.03 | NL | 0 |
| Bryozoa | Gymnolaemata | Cheilostomata | Microporellidae | Microporella | ciliata | 0.35 | NL | 0 |
| Bryozoa | Gymnolaemata | Cheilostomata | Scrupocellariidae | Scrupocellaria | scrupea | 0.9 | NL | 0 |
| Bryozoa | Gymnolaemata | Cheilostomata | Scrupocellariidae | Scrupocellaria | scruposa | 0.79 | NL | 0 |
| Chordata | Actinopterygii | Acipenseriformes | Acipenseridae | Acipenser | naccarii | 0.49 | VU | 0 |
| Chordata | Actinopterygii | Acipenseriformes | Acipenseridae | Acipenser | sturio | 0.27 | CR | 1 |
| Chordata | Actinopterygii | Anguilliformes | Anguillidae | Anguilla | anguilla | 0.52 | CR | 1 |
| Chordata | Actinopterygii | Anguilliformes | Chlopsidae | Chlopsis | bicolor | 0.55 | NL | 0 |
| Chordata | Actinopterygii | Anguilliformes | Congridae | Ariosoma | balearicum | 0.38 | NL | 0 |
| Chordata | Actinopterygii | Anguilliformes | Congridae | Conger | conger | 0.48 | NL | 1 |
| Chordata | Actinopterygii | Anguilliformes | Congridae | Gnathophis | mystax | 0.76 | NL | 0 |
| Chordata | Actinopterygii | Anguilliformes | Heterenchelyidae | Panturichthys | fowleri | 0.68 | NL | 0 |
| Chordata | Actinopterygii | Anguilliformes | Muraenidae | Gymnothorax | unicolor | 0.84 | NL | 0 |
| Chordata | Actinopterygii | Anguilliformes | Muraenidae | Muraena | helena | 0.86 | NL | 0 |
| Chordata | Actinopterygii | Anguilliformes | Nemichthyidae | Nemichthys | scolopaceus | 0.37 | NL | 0 |
| Chordata | Actinopterygii | Anguilliformes | Nettastomatidae | Facciolella | oxyrhyncha | 0.55 | NL | 0 |
| Chordata | Actinopterygii | Anguilliformes | Nettastomatidae | Nettastoma | melanurum | 0.54 | NL | 0 |
| Chordata | Actinopterygii | Anguilliformes | Ophichthidae | Dalophis | imberbis | 0.71 | NL | 0 |
| Chordata | Actinopterygii | Anguilliformes | Ophichthidae | Echelus | myrus | 0.72 | NL | 0 |
| Chordata | Actinopterygii | Anguilliformes | Ophichthidae | Mystriophis | crosnieri | 0.01 | NL | 0 |
| Chordata | Actinopterygii | Anguilliformes | Ophichthidae | Ophichthus | ophis | 0.32 | NL | 0 |
| Chordata | Actinopterygii | Anguilliformes | Ophichthidae | Ophichthus | rufus | 0.71 | NL | 0 |
| Chordata | Actinopterygii | Anguilliformes | Ophichthidae | Ophisurus | serpens | 0.75 | NL | 0 |
| Chordata | Actinopterygii | Anguilliformes | Serrivomeridae | Serrivomer | brevidentatus | 0.35 | NL | 0 |
| Chordata | Actinopterygii | Atheriniformes | Atherinidae | Atherina | boyeri | 0.86 | LC | 0 |
| Chordata | Actinopterygii | Atheriniformes | Atherinidae | Atherina | hepsetus | 0.77 | NL | 0 |
| Chordata | Actinopterygii | Atheriniformes | Atherinidae | Atherina | presbyter | 0.86 | NL | 0 |
| Chordata | Actinopterygii | Aulopiformes | Alepisauridae | Alepisaurus | ferox | 0.39 | NL | 0 |
| Chordata | Actinopterygii | Aulopiformes | Aulopidae | Aulopus | filamentosus | 0.36 | NL | 0 |
| Chordata | Actinopterygii | Aulopiformes | Chlorophthalmidae | Chlorophthalmus | agassizi | 0.46 | NL | 0 |
| Chordata | Actinopterygii | Aulopiformes | Evermannellidae | Evermannella | balbo | 0.37 | NL | 0 |
| Chordata | Actinopterygii | Aulopiformes | Ipnopidae | Bathypterois | dubius | 0.43 | NL | 0 |
| Chordata | Actinopterygii | Aulopiformes | Paralepididae | Arctozenus | risso | 0.33 | NL | 0 |
| Chordata | Actinopterygii | Aulopiformes | Paralepididae | Lestidiops | jayakari jayakari | 0.18 | NL | 0 |
| Chordata | Actinopterygii | Aulopiformes | Paralepididae | Lestidiops | jayakari pseudosphyraenoides | 0.91 | NL | 0 |
| Chordata | Actinopterygii | Aulopiformes | Paralepididae | Lestidiops | sphyrenoides | 0.59 | NL | 0 |
| Chordata | Actinopterygii | Aulopiformes | Paralepididae | Lestidium | atlanticum | 0.26 | NL | 0 |
| Chordata | Actinopterygii | Aulopiformes | Paralepididae | Paralepis | coregonoides | 0.82 | NL | 0 |
| Chordata | Actinopterygii | Aulopiformes | Paralepididae | Sudis | hyalina | 0.45 | NL | 0 |
| Chordata | Actinopterygii | Batrachoidiformes | Batrachoididae | Halobatrachus | didactylus | 0.02 | NL | 0 |
| Chordata | Actinopterygii | Beloniformes | Belonidae | Belone | belone | 0.79 | NL | 0 |
| Chordata | Actinopterygii | Beloniformes | Belonidae | Tylosurus | acus acus | 0.07 | NL | 0 |
| Chordata | Actinopterygii | Beloniformes | Exocoetidae | Cheilopogon | heterurus | 0.36 | NL | 0 |
| Chordata | Actinopterygii | Beloniformes | Exocoetidae | Exocoetus | obtusirostris | 0.33 | NL | 0 |
| Chordata | Actinopterygii | Beloniformes | Exocoetidae | Exocoetus | volitans | 0.26 | NL | 0 |
| Chordata | Actinopterygii | Beloniformes | Exocoetidae | Hirundichthys | rondeletii | 0.48 | NL | 0 |
| Chordata | Actinopterygii | Beloniformes | Exocoetidae | Hirundichthys | speculiger | 0.29 | NL | 0 |
| Chordata | Actinopterygii | Beloniformes | Hemiramphidae | Hyporhamphus | picarti | 0.83 | NL | 0 |
| Chordata | Actinopterygii | Beloniformes | Scomberesocidae | Scomberesox | saurus saurus | 0.51 | NL | 0 |
| Chordata | Actinopterygii | Beryciformes | Berycidae | Beryx | decadactylus | 0.2 | NL | 0 |
| Chordata | Actinopterygii | Beryciformes | Trachichthyidae | Hoplostethus | mediterraneus mediterraneus | 0.42 | NL | 0 |
| Chordata | Actinopterygii | Clupeiformes | Clupeidae | Alosa | alosa | 0.58 | LC | 1 |
| Chordata | Actinopterygii | Clupeiformes | Clupeidae | Alosa | fallax | 0.58 | LC | 1 |
| Chordata | Actinopterygii | Clupeiformes | Clupeidae | Sardina | pilchardus | 0.75 | NL | 1 |
| Chordata | Actinopterygii | Clupeiformes | Clupeidae | Sardinella | aurita | 0.53 | NL | 1 |
| Chordata | Actinopterygii | Clupeiformes | Clupeidae | Sardinella | maderensis | 0.05 | NL | 0 |
| Chordata | Actinopterygii | Clupeiformes | Clupeidae | Sprattus | sprattus sprattus | 0.44 | NL | 1 |
| Chordata | Actinopterygii | Clupeiformes | Engraulidae | Engraulis | encrasicolus | 0.55 | NL | 1 |
| Chordata | Actinopterygii | Cyprinodontiformes | Cyprinodontidae | Aphanius | fasciatus | 0.86 | LC | 0 |
| Chordata | Actinopterygii | Gadiformes | Gadidae | Gadiculus | argenteus argenteus | 0.7 | NL | 1 |
| Chordata | Actinopterygii | Gadiformes | Gadidae | Merlangius | merlangus | 0.05 | NL | 1 |
| Chordata | Actinopterygii | Gadiformes | Gadidae | Micromesistius | poutassou | 0.58 | NL | 1 |
| Chordata | Actinopterygii | Gadiformes | Gadidae | Trisopterus | luscus | 0.07 | NL | 0 |
| Chordata | Actinopterygii | Gadiformes | Gadidae | Trisopterus | minutus | 0.5 | NL | 0 |
| Chordata | Actinopterygii | Gadiformes | Lotidae | Gaidropsarus | biscayensis | 0.58 | NL | 1 |
| Chordata | Actinopterygii | Gadiformes | Lotidae | Gaidropsarus | mediterraneus | 0.61 | NL | 0 |
| Chordata | Actinopterygii | Gadiformes | Lotidae | Gaidropsarus | vulgaris | 0.38 | NL | 1 |
| Chordata | Actinopterygii | Gadiformes | Lotidae | Molva | dypterygia | 0.62 | NL | 0 |
| Chordata | Actinopterygii | Gadiformes | Lotidae | Molva | molva | 0.04 | NL | 0 |
| Chordata | Actinopterygii | Gadiformes | Macrouridae | Hymenocephalus | italicus | 0.46 | NL | 0 |
| Chordata | Actinopterygii | Gadiformes | Macrouridae | Nezumia | aequalis | 0.36 | NL | 0 |
| Chordata | Actinopterygii | Gadiformes | Macrouridae | Nezumia | sclerorhynchus | 0.34 | NL | 0 |
| Chordata | Actinopterygii | Gadiformes | Macrouridae | Trachyrincus | scabrus | 0.48 | NL | 0 |
| Chordata | Actinopterygii | Gadiformes | Merlucciidae | Merluccius | merluccius | 0.65 | NL | 1 |
| Chordata | Actinopterygii | Gadiformes | Moridae | Gadella | maraldi | 0.27 | NL | 0 |
| Chordata | Actinopterygii | Gadiformes | Moridae | Guttigadus | latifrons | 0.01 | NL | 0 |
| Chordata | Actinopterygii | Gadiformes | Moridae | Lepidion | lepidion | 0.78 | NL | 0 |
| Chordata | Actinopterygii | Gadiformes | Moridae | Mora | moro | 0.07 | NL | 0 |
| Chordata | Actinopterygii | Gadiformes | Moridae | Physiculus | dalwigki | 0.15 | NL | 0 |
| Chordata | Actinopterygii | Gadiformes | Moridae | Rhynchogadus | hepaticus | 0.6 | NL | 0 |
| Chordata | Actinopterygii | Gadiformes | Phycidae | Phycis | blennoides | 0.68 | NL | 0 |
| Chordata | Actinopterygii | Gadiformes | Phycidae | Phycis | phycis | 0.66 | NL | 0 |
| Chordata | Actinopterygii | Gasterosteiformes | Gasterosteidae | Gasterosteus | aculeatus aculeatus | 0.11 | LC | 1 |
| Chordata | Actinopterygii | Gobiesociformes | Gobiesocidae | Diplecogaster | bimaculata bimaculata | 0.14 | NL | 0 |
| Chordata | Actinopterygii | Gobiesociformes | Gobiesocidae | Lepadogaster | candolii | 0.98 | NL | 0 |
| Chordata | Actinopterygii | Gobiesociformes | Gobiesocidae | Lepadogaster | lepadogaster | 0.67 | NL | 0 |
| Chordata | Actinopterygii | Gobiesociformes | Gobiesocidae | Opeatogenys | gracilis | 1 | NL | 0 |
| Chordata | Actinopterygii | Lampriformes | Lampridae | Lampris | guttatus | 0.18 | NL | 0 |
| Chordata | Actinopterygii | Lampriformes | Lophotidae | Lophotus | lacepede | 0.68 | NL | 0 |
| Chordata | Actinopterygii | Lampriformes | Regalecidae | Regalecus | glesne | 0.33 | NL | 0 |
| Chordata | Actinopterygii | Lampriformes | Trachipteridae | Trachipterus | arcticus | 0.35 | NL | 0 |
| Chordata | Actinopterygii | Lampriformes | Trachipteridae | Trachipterus | trachypterus | 0.54 | NL | 0 |
| Chordata | Actinopterygii | Lophiiformes | Lophiidae | Lophius | budegassa | 0.51 | NL | 0 |
| Chordata | Actinopterygii | Lophiiformes | Lophiidae | Lophius | piscatorius | 0.49 | NL | 1 |
| Chordata | Actinopterygii | Mugiliformes | Mugilidae | Chelon | labrosus | 0.69 | LC | 0 |
| Chordata | Actinopterygii | Mugiliformes | Mugilidae | Liza | aurata | 0.83 | LC | 0 |
| Chordata | Actinopterygii | Mugiliformes | Mugilidae | Liza | ramado | 0.84 | LC | 0 |
| Chordata | Actinopterygii | Mugiliformes | Mugilidae | Liza | saliens | 0.86 | LC | 0 |
| Chordata | Actinopterygii | Mugiliformes | Mugilidae | Mugil | cephalus | 0.37 | LC | 1 |
| Chordata | Actinopterygii | Mugiliformes | Mugilidae | Oedalechilus | labeo | 0.83 | NL | 0 |
| Chordata | Actinopterygii | Myctophiformes | Myctophidae | Benthosema | glaciale | 0.4 | NL | 0 |
| Chordata | Actinopterygii | Myctophiformes | Myctophidae | Ceratoscopelus | maderensis | 0.61 | NL | 0 |
| Chordata | Actinopterygii | Myctophiformes | Myctophidae | Diaphus | holti | 0.68 | NL | 0 |
| Chordata | Actinopterygii | Myctophiformes | Myctophidae | Diaphus | metopoclampus | 0.37 | NL | 0 |
| Chordata | Actinopterygii | Myctophiformes | Myctophidae | Diaphus | rafinesquii | 0.53 | NL | 0 |
| Chordata | Actinopterygii | Myctophiformes | Myctophidae | Diogenichthys | atlanticus | 0.31 | NL | 0 |
| Chordata | Actinopterygii | Myctophiformes | Myctophidae | Electrona | risso | 0.37 | NL | 0 |
| Chordata | Actinopterygii | Myctophiformes | Myctophidae | Gonichthys | cocco | 0.38 | NL | 0 |
| Chordata | Actinopterygii | Myctophiformes | Myctophidae | Hygophum | benoiti | 0.49 | NL | 0 |
| Chordata | Actinopterygii | Myctophiformes | Myctophidae | Hygophum | hygomii | 0.42 | NL | 0 |
| Chordata | Actinopterygii | Myctophiformes | Myctophidae | Lampanyctus | crocodilus | 0.56 | NL | 0 |
| Chordata | Actinopterygii | Myctophiformes | Myctophidae | Lampanyctus | pusillus | 0.39 | NL | 0 |
| Chordata | Actinopterygii | Myctophiformes | Myctophidae | Lobianchia | dofleini | 0.37 | NL | 0 |
| Chordata | Actinopterygii | Myctophiformes | Myctophidae | Lobianchia | gemellarii | 0.38 | NL | 0 |
| Chordata | Actinopterygii | Myctophiformes | Myctophidae | Myctophum | punctatum | 0.58 | NL | 0 |
| Chordata | Actinopterygii | Myctophiformes | Myctophidae | Notoscopelus | bolini | 0.66 | NL | 0 |
| Chordata | Actinopterygii | Myctophiformes | Myctophidae | Notoscopelus | elongatus | 0.78 | NL | 0 |
| Chordata | Actinopterygii | Myctophiformes | Myctophidae | Notoscopelus | kroyeri | 0.26 | NL | 0 |
| Chordata | Actinopterygii | Myctophiformes | Myctophidae | Symbolophorus | veranyi | 0.6 | NL | 0 |
| Chordata | Actinopterygii | Notacanthiformes | Notacanthidae | Notacanthus | bonaparte | 0.8 | NL | 0 |
| Chordata | Actinopterygii | Notacanthiformes | Notacanthidae | Polyacanthonotus | rissoanus | 0.01 | NL | 0 |
| Chordata | Actinopterygii | Ophidiiformes | Bythitidae | Cataetyx | alleni | 0.87 | NL | 0 |
| Chordata | Actinopterygii | Ophidiiformes | Bythitidae | Cataetyx | laticeps | 0.07 | NL | 0 |
| Chordata | Actinopterygii | Ophidiiformes | Carapidae | Carapus | acus | 0.68 | NL | 0 |
| Chordata | Actinopterygii | Ophidiiformes | Carapidae | Echiodon | dentatus | 0.6 | NL | 0 |
| Chordata | Actinopterygii | Ophidiiformes | Ophidiidae | Benthocometes | robustus | 0.54 | NL | 0 |
| Chordata | Actinopterygii | Ophidiiformes | Ophidiidae | Ophidion | barbatum | 0.56 | NL | 0 |
| Chordata | Actinopterygii | Osmeriformes | Alepocephalidae | Alepocephalus | rostratus | 0.62 | NL | 0 |
| Chordata | Actinopterygii | Osmeriformes | Argentinidae | Argentina | sphyraena | 0.58 | NL | 0 |
| Chordata | Actinopterygii | Osmeriformes | Argentinidae | Glossanodon | leioglossus | 0.77 | NL | 0 |
| Chordata | Actinopterygii | Osmeriformes | Microstomatidae | Microstoma | microstoma | 0.36 | NL | 0 |
| Chordata | Actinopterygii | Osmeriformes | Microstomatidae | Nansenia | oblita | 0.96 | NL | 0 |
| Chordata | Actinopterygii | Perciformes | Ammodytidae | Ammodytes | tobianus | 0.07 | NL | 0 |
| Chordata | Actinopterygii | Perciformes | Ammodytidae | Gymnammodytes | cicerelus | 0.67 | NL | 0 |
| Chordata | Actinopterygii | Perciformes | Apogonidae | Apogon | imberbis | 0.69 | NL | 0 |
| Chordata | Actinopterygii | Perciformes | Blenniidae | Aidablennius | sphynx | 0.85 | NL | 0 |
| Chordata | Actinopterygii | Perciformes | Blenniidae | Blennius | ocellaris | 0.65 | NL | 0 |
| Chordata | Actinopterygii | Perciformes | Blenniidae | Coryphoblennius | galerita | 0.83 | NL | 0 |
| Chordata | Actinopterygii | Perciformes | Blenniidae | Lipophrys | canevae | 0.62 | NL | 0 |
| Chordata | Actinopterygii | Perciformes | Blenniidae | Lipophrys | pholis | 0.39 | NL | 0 |
| Chordata | Actinopterygii | Perciformes | Blenniidae | Parablennius | gattorugine | 0.63 | NL | 0 |
| Chordata | Actinopterygii | Perciformes | Blenniidae | Parablennius | incognitus | 0.7 | NL | 0 |
| Chordata | Actinopterygii | Perciformes | Blenniidae | Parablennius | pilicornis | 0.57 | NL | 0 |
| Chordata | Actinopterygii | Perciformes | Blenniidae | Parablennius | rouxi | 0.7 | NL | 0 |
| Chordata | Actinopterygii | Perciformes | Blenniidae | Parablennius | sanguinolentus | 0.78 | NL | 0 |
| Chordata | Actinopterygii | Perciformes | Blenniidae | Parablennius | tentacularis | 0.89 | NL | 0 |
| Chordata | Actinopterygii | Perciformes | Blenniidae | Parablennius | zvonimiri | 0.76 | NL | 0 |
| Chordata | Actinopterygii | Perciformes | Blenniidae | Paralipophrys | trigloides | 0.76 | NL | 0 |
| Chordata | Actinopterygii | Perciformes | Blenniidae | Salaria | pavo | 0.88 | NL | 0 |
| Chordata | Actinopterygii | Perciformes | Blenniidae | Scartella | cristata | 0.67 | NL | 0 |
| Chordata | Actinopterygii | Perciformes | Bramidae | Brama | brama | 0.27 | NL | 0 |
| Chordata | Actinopterygii | Perciformes | Callanthiidae | Callanthias | ruber | 0.82 | NL | 0 |
| Chordata | Actinopterygii | Perciformes | Callionymidae | Callionymus | lyra | 0.22 | NL | 1 |
| Chordata | Actinopterygii | Perciformes | Callionymidae | Callionymus | maculatus | 0.53 | NL | 0 |
| Chordata | Actinopterygii | Perciformes | Callionymidae | Callionymus | pusillus | 0.81 | NL | 0 |
| Chordata | Actinopterygii | Perciformes | Callionymidae | Callionymus | reticulatus | 0.83 | NL | 0 |
| Chordata | Actinopterygii | Perciformes | Callionymidae | Callionymus | risso | 0.84 | NL | 0 |
| Chordata | Actinopterygii | Perciformes | Callionymidae | Synchiropus | phaeton | 0.6 | NL | 0 |
| Chordata | Actinopterygii | Perciformes | Caproidae | Capros | aper | 0.6 | NL | 0 |
| Chordata | Actinopterygii | Perciformes | Carangidae | Campogramma | glaycos | 0.78 | NL | 0 |
| Chordata | Actinopterygii | Perciformes | Carangidae | Caranx | crysos | 0.17 | NL | 1 |
| Chordata | Actinopterygii | Perciformes | Carangidae | Caranx | hippos | 0.31 | NL | 1 |
| Chordata | Actinopterygii | Perciformes | Carangidae | Caranx | rhonchus | 0.19 | NL | 0 |
| Chordata | Actinopterygii | Perciformes | Carangidae | Decapterus | macarellus | 0.38 | NL | 0 |
| Chordata | Actinopterygii | Perciformes | Carangidae | Decapterus | punctatus | 0.34 | NL | 0 |
| Chordata | Actinopterygii | Perciformes | Carangidae | Elagatis | bipinnulata | 0.11 | NL | 0 |
| Chordata | Actinopterygii | Perciformes | Carangidae | Lichia | amia | 0.58 | NL | 0 |
| Chordata | Actinopterygii | Perciformes | Carangidae | Naucrates | ductor | 0.28 | NL | 0 |
| Chordata | Actinopterygii | Perciformes | Carangidae | Pseudocaranx | dentex | 0.32 | NL | 0 |
| Chordata | Actinopterygii | Perciformes | Carangidae | Seriola | dumerili | 0.36 | NL | 0 |
| Chordata | Actinopterygii | Perciformes | Carangidae | Trachinotus | ovatus | 0.57 | NL | 0 |
| Chordata | Actinopterygii | Perciformes | Carangidae | Trachurus | mediterraneus | 0.68 | NL | 0 |
| Chordata | Actinopterygii | Perciformes | Carangidae | Trachurus | picturatus | 0.89 | NL | 0 |
| Chordata | Actinopterygii | Perciformes | Carangidae | Trachurus | trachurus | 0.42 | NL | 1 |
| Chordata | Actinopterygii | Perciformes | Centracanthidae | Spicara | maena | 0.74 | NL | 0 |
| Chordata | Actinopterygii | Perciformes | Centracanthidae | Spicara | smaris | 0.73 | NL | 0 |
| Chordata | Actinopterygii | Perciformes | Centrolophidae | Centrolophus | niger | 0.2 | NL | 0 |
| Chordata | Actinopterygii | Perciformes | Centrolophidae | Hyperoglyphe | perciformis | 0.74 | NL | 0 |
| Chordata | Actinopterygii | Perciformes | Centrolophidae | Schedophilus | medusophagus | 0.33 | NL | 0 |
| Chordata | Actinopterygii | Perciformes | Centrolophidae | Schedophilus | ovalis | 0.49 | NL | 0 |
| Chordata | Actinopterygii | Perciformes | Cepolidae | Cepola | macrophthalma | 0.69 | NL | 0 |
| Chordata | Actinopterygii | Perciformes | Chaetodontidae | Chaetodon | melannotus | 0.01 | NL | 0 |
| Chordata | Actinopterygii | Perciformes | Clinidae | Clinitrachus | argentatus | 0.71 | NL | 0 |
| Chordata | Actinopterygii | Perciformes | Coryphaenidae | Coryphaena | equiselis | 0.25 | NL | 0 |
| Chordata | Actinopterygii | Perciformes | Coryphaenidae | Coryphaena | hippurus | 0.19 | NL | 1 |
| Chordata | Actinopterygii | Perciformes | Echeneidae | Echeneis | naucrates | 0.26 | NL | 1 |
| Chordata | Actinopterygii | Perciformes | Echeneidae | Remora | australis | 0.13 | NL | 0 |
| Chordata | Actinopterygii | Perciformes | Echeneidae | Remora | brachyptera | 0.32 | NL | 0 |
| Chordata | Actinopterygii | Perciformes | Echeneidae | Remora | osteochir | 0.35 | NL | 0 |
| Chordata | Actinopterygii | Perciformes | Echeneidae | Remora | remora | 0.33 | NL | 0 |
| Chordata | Actinopterygii | Perciformes | Epigonidae | Epigonus | constanciae | 0.28 | NL | 0 |
| Chordata | Actinopterygii | Perciformes | Epigonidae | Epigonus | denticulatus | 0.58 | NL | 0 |
| Chordata | Actinopterygii | Perciformes | Epigonidae | Epigonus | telescopus | 0.26 | NL | 0 |
| Chordata | Actinopterygii | Perciformes | Epigonidae | Microichthys | coccoi | 1 | NL | 0 |
| Chordata | Actinopterygii | Perciformes | Gempylidae | Ruvettus | pretiosus | 0.2 | NL | 1 |
| Chordata | Actinopterygii | Perciformes | Gobiidae | Aphia | minuta | 0.64 | NL | 0 |
| Chordata | Actinopterygii | Perciformes | Gobiidae | Corcyrogobius | liechtensteini | 1 | NL | 0 |
| Chordata | Actinopterygii | Perciformes | Gobiidae | Crystallogobius | linearis | 0.78 | NL | 0 |
| Chordata | Actinopterygii | Perciformes | Gobiidae | Deltentosteus | quadrimaculatus | 0.7 | NL | 0 |
| Chordata | Actinopterygii | Perciformes | Gobiidae | Didogobius | bentuvii | 1 | NL | 0 |
| Chordata | Actinopterygii | Perciformes | Gobiidae | Gammogobius | steinitzi | 1 | NL | 0 |
| Chordata | Actinopterygii | Perciformes | Gobiidae | Gobius | cobitis | 0.74 | NL | 1 |
| Chordata | Actinopterygii | Perciformes | Gobiidae | Gobius | cruentatus | 0.77 | NL | 0 |
| Chordata | Actinopterygii | Perciformes | Gobiidae | Gobius | geniporus | 0.8 | NL | 0 |
| Chordata | Actinopterygii | Perciformes | Gobiidae | Gobius | niger | 0.68 | NL | 0 |
| Chordata | Actinopterygii | Perciformes | Gobiidae | Gobius | paganellus | 0.74 | NL | 0 |
| Chordata | Actinopterygii | Perciformes | Gobiidae | Lesueurigobius | friesii | 0.66 | NL | 0 |
| Chordata | Actinopterygii | Perciformes | Gobiidae | Lesueurigobius | sanzi | 0.94 | NL | 0 |
| Chordata | Actinopterygii | Perciformes | Gobiidae | Lesueurigobius | suerii | 0.71 | NL | 0 |
| Chordata | Actinopterygii | Perciformes | Gobiidae | Millerigobius | macrocephalus | 1 | NL | 0 |
| Chordata | Actinopterygii | Perciformes | Gobiidae | Odondebuenia | balearica | 1 | NL | 0 |
| Chordata | Actinopterygii | Perciformes | Gobiidae | Pomatoschistus | bathi | 1 | NL | 0 |
| Chordata | Actinopterygii | Perciformes | Gobiidae | Pomatoschistus | marmoratus | 0.8 | NL | 0 |
| Chordata | Actinopterygii | Perciformes | Gobiidae | Pomatoschistus | microps | 0.27 | LC | 0 |
| Chordata | Actinopterygii | Perciformes | Gobiidae | Pomatoschistus | minutus | 0.06 | NL | 0 |
| Chordata | Actinopterygii | Perciformes | Gobiidae | Pomatoschistus | pictus | 0.08 | NL | 1 |
| Chordata | Actinopterygii | Perciformes | Gobiidae | Thorogobius | ephippiatus | 0.03 | NL | 0 |
| Chordata | Actinopterygii | Perciformes | Gobiidae | Zosterisessor | ophiocephalus | 0.78 | DD | 0 |
| Chordata | Actinopterygii | Perciformes | Haemulidae | Plectorhinchus | mediterraneus | 0.06 | NL | 0 |
| Chordata | Actinopterygii | Perciformes | Haemulidae | Pomadasys | incisus | 0.18 | NL | 0 |
| Chordata | Actinopterygii | Perciformes | Istiophoridae | Makaira | nigricans | 0.32 | NL | 0 |
| Chordata | Actinopterygii | Perciformes | Istiophoridae | Tetrapturus | albidus | 0.47 | NL | 0 |
| Chordata | Actinopterygii | Perciformes | Istiophoridae | Tetrapturus | belone | 1 | NL | 0 |
| Chordata | Actinopterygii | Perciformes | Kyphosidae | Kyphosus | sectator | 0.41 | NL | 0 |
| Chordata | Actinopterygii | Perciformes | Labridae | Acantholabrus | palloni | 0.88 | NL | 0 |
| Chordata | Actinopterygii | Perciformes | Labridae | Coris | julis | 0.81 | NL | 0 |
| Chordata | Actinopterygii | Perciformes | Labridae | Ctenolabrus | rupestris | 0.06 | NL | 0 |
| Chordata | Actinopterygii | Perciformes | Labridae | Labrus | merula | 0.77 | NL | 0 |
| Chordata | Actinopterygii | Perciformes | Labridae | Labrus | mixtus | 0.08 | NL | 0 |
| Chordata | Actinopterygii | Perciformes | Labridae | Labrus | viridis | 0.77 | NL | 0 |
| Chordata | Actinopterygii | Perciformes | Labridae | Symphodus | bailloni | 0.5 | NL | 0 |
| Chordata | Actinopterygii | Perciformes | Labridae | Symphodus | cinereus | 0.77 | NL | 0 |
| Chordata | Actinopterygii | Perciformes | Labridae | Symphodus | doderleini | 0.75 | NL | 0 |
| Chordata | Actinopterygii | Perciformes | Labridae | Symphodus | mediterraneus | 0.79 | NL | 0 |
| Chordata | Actinopterygii | Perciformes | Labridae | Symphodus | melanocercus | 0.59 | NL | 0 |
| Chordata | Actinopterygii | Perciformes | Labridae | Symphodus | ocellatus | 0.75 | NL | 0 |
| Chordata | Actinopterygii | Perciformes | Labridae | Symphodus | roissali | 0.74 | NL | 0 |
| Chordata | Actinopterygii | Perciformes | Labridae | Symphodus | rostratus | 0.77 | NL | 0 |
| Chordata | Actinopterygii | Perciformes | Labridae | Symphodus | tinca | 0.9 | NL | 0 |
| Chordata | Actinopterygii | Perciformes | Labridae | Thalassoma | pavo | 0.76 | NL | 0 |
| Chordata | Actinopterygii | Perciformes | Labridae | Xyrichtys | novacula | 0.28 | NL | 0 |
| Chordata | Actinopterygii | Perciformes | Lobotidae | Lobotes | surinamensis | 0.36 | NL | 0 |
| Chordata | Actinopterygii | Perciformes | Luvaridae | Luvarus | imperialis | 0.32 | NL | 0 |
| Chordata | Actinopterygii | Perciformes | Moronidae | Dicentrarchus | labrax | 0.35 | LC | 1 |
| Chordata | Actinopterygii | Perciformes | Moronidae | Dicentrarchus | punctatus | 0.79 | NL | 0 |
| Chordata | Actinopterygii | Perciformes | Mullidae | Mullus | barbatus barbatus | 0.94 | NL | 0 |
| Chordata | Actinopterygii | Perciformes | Mullidae | Mullus | surmuletus | 0.73 | NL | 0 |
| Chordata | Actinopterygii | Perciformes | Mullidae | Pseudupeneus | prayensis | 0.12 | NL | 0 |
| Chordata | Actinopterygii | Perciformes | Nomeidae | Cubiceps | gracilis | 0.51 | NL | 0 |
| Chordata | Actinopterygii | Perciformes | Polynemidae | Galeoides | decadactylus | 0.12 | NL | 1 |
| Chordata | Actinopterygii | Perciformes | Polyprionidae | Polyprion | americanus | 0.24 | DD | 0 |
| Chordata | Actinopterygii | Perciformes | Pomacentridae | Chromis | chromis | 0.78 | NL | 0 |
| Chordata | Actinopterygii | Perciformes | Pomatomidae | Pomatomus | saltatrix | 0.28 | NL | 0 |
| Chordata | Actinopterygii | Perciformes | Priacanthidae | Priacanthus | arenatus | 0.36 | NL | 0 |
| Chordata | Actinopterygii | Perciformes | Scaridae | Sparisoma | cretense | 0.25 | NL | 0 |
| Chordata | Actinopterygii | Perciformes | Sciaenidae | Argyrosomus | regius | 0.58 | NL | 1 |
| Chordata | Actinopterygii | Perciformes | Sciaenidae | Sciaena | umbra | 0.54 | NL | 0 |
| Chordata | Actinopterygii | Perciformes | Sciaenidae | Umbrina | canariensis | 0.38 | NL | 0 |
| Chordata | Actinopterygii | Perciformes | Sciaenidae | Umbrina | cirrosa | 0.49 | NL | 0 |
| Chordata | Actinopterygii | Perciformes | Sciaenidae | Umbrina | ronchus | 0.65 | NL | 0 |
| Chordata | Actinopterygii | Perciformes | Scombridae | Acanthocybium | solandri | 0.28 | NL | 1 |
| Chordata | Actinopterygii | Perciformes | Scombridae | Auxis | rochei rochei | 0.65 | NL | 0 |
| Chordata | Actinopterygii | Perciformes | Scombridae | Auxis | thazard thazard | 0.28 | NL | 0 |
| Chordata | Actinopterygii | Perciformes | Scombridae | Euthynnus | alletteratus | 0.38 | NL | 0 |
| Chordata | Actinopterygii | Perciformes | Scombridae | Katsuwonus | pelamis | 0.43 | NL | 1 |
| Chordata | Actinopterygii | Perciformes | Scombridae | Orcynopsis | unicolor | 0.15 | NL | 0 |
| Chordata | Actinopterygii | Perciformes | Scombridae | Sarda | sarda | 0.43 | NL | 0 |
| Chordata | Actinopterygii | Perciformes | Scombridae | Scomber | scombrus | 0.35 | NL | 1 |
| Chordata | Actinopterygii | Perciformes | Scombridae | Thunnus | alalunga | 0.31 | DD | 0 |
| Chordata | Actinopterygii | Perciformes | Scombridae | Thunnus | thynnus | 0.66 | DD | 1 |
| Chordata | Actinopterygii | Perciformes | Serranidae | Anthias | anthias | 0.74 | NL | 0 |
| Chordata | Actinopterygii | Perciformes | Serranidae | Epinephelus | aeneus | 0.09 | NT | 0 |
| Chordata | Actinopterygii | Perciformes | Serranidae | Epinephelus | caninus | 0.03 | DD | 0 |
| Chordata | Actinopterygii | Perciformes | Serranidae | Epinephelus | marginatus | 0.41 | EN | 1 |
| Chordata | Actinopterygii | Perciformes | Serranidae | Mycteroperca | rubra | 0.89 | LC | 0 |
| Chordata | Actinopterygii | Perciformes | Serranidae | Serranus | atricauda | 0.58 | NL | 0 |
| Chordata | Actinopterygii | Perciformes | Serranidae | Serranus | cabrilla | 0.7 | NL | 0 |
| Chordata | Actinopterygii | Perciformes | Serranidae | Serranus | hepatus | 0.74 | NL | 0 |
| Chordata | Actinopterygii | Perciformes | Serranidae | Serranus | scriba | 0.77 | NL | 0 |
| Chordata | Actinopterygii | Perciformes | Sparidae | Boops | boops | 0.64 | NL | 1 |
| Chordata | Actinopterygii | Perciformes | Sparidae | Dentex | dentex | 0.82 | NL | 1 |
| Chordata | Actinopterygii | Perciformes | Sparidae | Dentex | gibbosus | 0.06 | NL | 1 |
| Chordata | Actinopterygii | Perciformes | Sparidae | Dentex | macrophthalmus | 0.45 | NL | 0 |
| Chordata | Actinopterygii | Perciformes | Sparidae | Dentex | maroccanus | 0.62 | NL | 0 |
| Chordata | Actinopterygii | Perciformes | Sparidae | Diplodus | annularis | 0.69 | NL | 0 |
| Chordata | Actinopterygii | Perciformes | Sparidae | Diplodus | cervinus cervinus | 0.64 | NL | 0 |
| Chordata | Actinopterygii | Perciformes | Sparidae | Diplodus | puntazzo | 0.84 | NL | 0 |
| Chordata | Actinopterygii | Perciformes | Sparidae | Diplodus | sargus sargus | 0.77 | NL | 1 |
| Chordata | Actinopterygii | Perciformes | Sparidae | Diplodus | vulgaris | 0.77 | NL | 0 |
| Chordata | Actinopterygii | Perciformes | Sparidae | Lithognathus | mormyrus | 0.69 | NL | 0 |
| Chordata | Actinopterygii | Perciformes | Sparidae | Oblada | melanura | 0.79 | NL | 0 |
| Chordata | Actinopterygii | Perciformes | Sparidae | Pagellus | acarne | 0.7 | NL | 0 |
| Chordata | Actinopterygii | Perciformes | Sparidae | Pagellus | bellottii | 0.18 | NL | 0 |
| Chordata | Actinopterygii | Perciformes | Sparidae | Pagellus | bogaraveo | 0.7 | NL | 0 |
| Chordata | Actinopterygii | Perciformes | Sparidae | Pagellus | erythrinus | 0.73 | NL | 0 |
| Chordata | Actinopterygii | Perciformes | Sparidae | Pagrus | auriga | 0.07 | NL | 0 |
| Chordata | Actinopterygii | Perciformes | Sparidae | Pagrus | caeruleostictus | 0.23 | NL | 0 |
| Chordata | Actinopterygii | Perciformes | Sparidae | Pagrus | pagrus | 0.58 | EN | 1 |
| Chordata | Actinopterygii | Perciformes | Sparidae | Sarpa | salpa | 0.79 | NL | 0 |
| Chordata | Actinopterygii | Perciformes | Sparidae | Sparus | aurata | 0.78 | NL | 1 |
| Chordata | Actinopterygii | Perciformes | Sparidae | Spondyliosoma | cantharus | 0.72 | NL | 0 |
| Chordata | Actinopterygii | Perciformes | Sphyraenidae | Sphyraena | sphyraena | 0.66 | NL | 0 |
| Chordata | Actinopterygii | Perciformes | Stromateidae | Pampus | argenteus | 0.13 | NL | 0 |
| Chordata | Actinopterygii | Perciformes | Stromateidae | Stromateus | fiatola | 0.44 | NL | 0 |
| Chordata | Actinopterygii | Perciformes | Tetragonuridae | Tetragonurus | cuvieri | 0.32 | NL | 0 |
| Chordata | Actinopterygii | Perciformes | Trachinidae | Echiichthys | vipera | 0.15 | NL | 0 |
| Chordata | Actinopterygii | Perciformes | Trachinidae | Trachinus | draco | 0.7 | NL | 0 |
| Chordata | Actinopterygii | Perciformes | Trachinidae | Trachinus | radiatus | 0.35 | NL | 0 |
| Chordata | Actinopterygii | Perciformes | Trichiuridae | Lepidopus | caudatus | 0.37 | NL | 0 |
| Chordata | Actinopterygii | Perciformes | Trichiuridae | Trichiurus | lepturus | 0.3 | NL | 1 |
| Chordata | Actinopterygii | Perciformes | Tripterygiidae | Tripterygion | delaisi | 0.86 | NL | 0 |
| Chordata | Actinopterygii | Perciformes | Tripterygiidae | Tripterygion | tripteronotus | 0.81 | NL | 0 |
| Chordata | Actinopterygii | Perciformes | Uranoscopidae | Uranoscopus | scaber | 0.79 | NL | 0 |
| Chordata | Actinopterygii | Perciformes | Xiphiidae | Xiphias | gladius | 0.33 | DD | 1 |
| Chordata | Actinopterygii | Perciformes | Zoarcidae | Melanostigma | atlanticum | 0.01 | NL | 0 |
| Chordata | Actinopterygii | Pleuronectiformes | Bothidae | Arnoglossus | imperialis | 0.29 | NL | 1 |
| Chordata | Actinopterygii | Pleuronectiformes | Bothidae | Arnoglossus | laterna | 0.67 | NL | 0 |
| Chordata | Actinopterygii | Pleuronectiformes | Bothidae | Arnoglossus | rueppelii | 0.87 | NL | 0 |
| Chordata | Actinopterygii | Pleuronectiformes | Bothidae | Arnoglossus | thori | 0.73 | NL | 0 |
| Chordata | Actinopterygii | Pleuronectiformes | Bothidae | Bothus | podas | 0.6 | NL | 1 |
| Chordata | Actinopterygii | Pleuronectiformes | Citharidae | Citharus | linguatula | 0.62 | NL | 0 |
| Chordata | Actinopterygii | Pleuronectiformes | Cynoglossidae | Symphurus | nigrescens | 0.56 | NL | 0 |
| Chordata | Actinopterygii | Pleuronectiformes | Pleuronectidae | Platichthys | flesus | 0.05 | LC | 1 |
| Chordata | Actinopterygii | Pleuronectiformes | Pleuronectidae | Pleuronectes | platessa | 0.19 | LC | 0 |
| Chordata | Actinopterygii | Pleuronectiformes | Scophthalmidae | Lepidorhombus | boscii | 0.61 | NL | 0 |
| Chordata | Actinopterygii | Pleuronectiformes | Scophthalmidae | Lepidorhombus | whiffiagonis | 0.32 | NL | 0 |
| Chordata | Actinopterygii | Pleuronectiformes | Scophthalmidae | Psetta | maxima | 0.57 | NL | 0 |
| Chordata | Actinopterygii | Pleuronectiformes | Scophthalmidae | Scophthalmus | rhombus | 0.66 | NL | 0 |
| Chordata | Actinopterygii | Pleuronectiformes | Scophthalmidae | Zeugopterus | regius | 0.45 | NL | 0 |
| Chordata | Actinopterygii | Pleuronectiformes | Soleidae | Bathysolea | profundicola | 0.63 | NL | 0 |
| Chordata | Actinopterygii | Pleuronectiformes | Soleidae | Buglossidium | luteum | 0.43 | NL | 1 |
| Chordata | Actinopterygii | Pleuronectiformes | Soleidae | Dicologlossa | cuneata | 0.52 | NL | 0 |
| Chordata | Actinopterygii | Pleuronectiformes | Soleidae | Microchirus | azevia | 0.65 | NL | 0 |
| Chordata | Actinopterygii | Pleuronectiformes | Soleidae | Microchirus | boscanion | 0.24 | NL | 0 |
| Chordata | Actinopterygii | Pleuronectiformes | Soleidae | Microchirus | ocellatus | 0.41 | NL | 0 |
| Chordata | Actinopterygii | Pleuronectiformes | Soleidae | Microchirus | variegatus | 0.59 | NL | 0 |
| Chordata | Actinopterygii | Pleuronectiformes | Soleidae | Monochirus | hispidus | 0.5 | NL | 0 |
| Chordata | Actinopterygii | Pleuronectiformes | Soleidae | Pegusa | lascaris | 0.24 | NL | 0 |
| Chordata | Actinopterygii | Pleuronectiformes | Soleidae | Solea | solea | 0.37 | NL | 1 |
| Chordata | Actinopterygii | Pleuronectiformes | Soleidae | Synapturichthys | kleinii | 0.77 | NL | 0 |
| Chordata | Actinopterygii | Salmoniformes | Salmonidae | Salmo | salar | 0.32 | LR/lc | 1 |
| Chordata | Actinopterygii | Salmoniformes | Salmonidae | Salmo | trutta trutta | 0.04 | LC | 1 |
| Chordata | Actinopterygii | Scorpaeniformes | Cottidae | Taurulus | bubalis | 0.08 | NL | 0 |
| Chordata | Actinopterygii | Scorpaeniformes | Dactylopteridae | Dactylopterus | volitans | 0.37 | NL | 0 |
| Chordata | Actinopterygii | Scorpaeniformes | Liparidae | Eutelichthys | leptochirus | 0.66 | NL | 0 |
| Chordata | Actinopterygii | Scorpaeniformes | Peristediidae | Peristedion | cataphractum | 0.58 | NL | 0 |
| Chordata | Actinopterygii | Scorpaeniformes | Scorpaenidae | Pontinus | kuhlii | 0.24 | NL | 0 |
| Chordata | Actinopterygii | Scorpaeniformes | Scorpaenidae | Scorpaena | elongata | 0.43 | NL | 0 |
| Chordata | Actinopterygii | Scorpaeniformes | Scorpaenidae | Scorpaena | loppei | 0.8 | NL | 0 |
| Chordata | Actinopterygii | Scorpaeniformes | Scorpaenidae | Scorpaena | maderensis | 0.81 | NL | 0 |
| Chordata | Actinopterygii | Scorpaeniformes | Scorpaenidae | Scorpaena | notata | 0.69 | NL | 0 |
| Chordata | Actinopterygii | Scorpaeniformes | Scorpaenidae | Scorpaena | porcus | 0.75 | NL | 0 |
| Chordata | Actinopterygii | Scorpaeniformes | Scorpaenidae | Scorpaena | scrofa | 0.7 | NL | 0 |
| Chordata | Actinopterygii | Scorpaeniformes | Scorpaenidae | Scorpaena | stephanica | 0.56 | NL | 0 |
| Chordata | Actinopterygii | Scorpaeniformes | Sebastidae | Helicolenus | dactylopterus dactylopterus | 0.42 | NL | 0 |
| Chordata | Actinopterygii | Scorpaeniformes | Triglidae | Aspitrigla | cuculus | 0.69 | NL | 0 |
| Chordata | Actinopterygii | Scorpaeniformes | Triglidae | Chelidonichthys | lucerna | 0.66 | NL | 0 |
| Chordata | Actinopterygii | Scorpaeniformes | Triglidae | Chelidonichthys | obscurus | 0.66 | NL | 0 |
| Chordata | Actinopterygii | Scorpaeniformes | Triglidae | Eutrigla | gurnardus | 0.57 | NL | 0 |
| Chordata | Actinopterygii | Scorpaeniformes | Triglidae | Lepidotrigla | cavillone | 0.71 | NL | 0 |
| Chordata | Actinopterygii | Scorpaeniformes | Triglidae | Lepidotrigla | dieuzeidei | 0.76 | NL | 1 |
| Chordata | Actinopterygii | Scorpaeniformes | Triglidae | Trigla | lyra | 0.54 | NL | 0 |
| Chordata | Actinopterygii | Scorpaeniformes | Triglidae | Trigloporus | lastoviza | 0.66 | NL | 0 |
| Chordata | Actinopterygii | Stomiiformes | Gonostomatidae | Cyclothone | braueri | 0.57 | NL | 0 |
| Chordata | Actinopterygii | Stomiiformes | Gonostomatidae | Cyclothone | microdon | 0.35 | NL | 0 |
| Chordata | Actinopterygii | Stomiiformes | Gonostomatidae | Cyclothone | pygmaea | 0.9 | NL | 0 |
| Chordata | Actinopterygii | Stomiiformes | Gonostomatidae | Gonostoma | denudatum | 0.73 | NL | 0 |
| Chordata | Actinopterygii | Stomiiformes | Phosichthyidae | Ichthyococcus | ovatus | 0.44 | NL | 0 |
| Chordata | Actinopterygii | Stomiiformes | Phosichthyidae | Vinciguerria | attenuata | 0.45 | NL | 0 |
| Chordata | Actinopterygii | Stomiiformes | Phosichthyidae | Vinciguerria | poweriae | 0.47 | NL | 0 |
| Chordata | Actinopterygii | Stomiiformes | Sternoptychidae | Argyropelecus | hemigymnus | 0.38 | NL | 0 |
| Chordata | Actinopterygii | Stomiiformes | Sternoptychidae | Argyropelecus | olfersii | 0.24 | NL | 0 |
| Chordata | Actinopterygii | Stomiiformes | Sternoptychidae | Maurolicus | muelleri | 0.29 | NL | 0 |
| Chordata | Actinopterygii | Stomiiformes | Sternoptychidae | Valenciennellus | tripunctulatus | 0.35 | NL | 0 |
| Chordata | Actinopterygii | Stomiiformes | Stomiidae | Bathophilus | nigerrimus | 0.6 | NL | 0 |
| Chordata | Actinopterygii | Stomiiformes | Stomiidae | Bathophilus | vaillanti | 0.33 | NL | 0 |
| Chordata | Actinopterygii | Stomiiformes | Stomiidae | Borostomias | antarcticus | 0.01 | NL | 0 |
| Chordata | Actinopterygii | Stomiiformes | Stomiidae | Chauliodus | sloani | 0.35 | NL | 0 |
| Chordata | Actinopterygii | Stomiiformes | Stomiidae | Stomias | boa boa | 0.39 | NL | 0 |
| Chordata | Actinopterygii | Syngnathiformes | Centriscidae | Macroramphosus | scolopax | 0.63 | NL | 0 |
| Chordata | Actinopterygii | Syngnathiformes | Syngnathidae | Entelurus | aequoreus | 0.01 | NL | 0 |
| Chordata | Actinopterygii | Syngnathiformes | Syngnathidae | Hippocampus | guttulatus | 0.86 | DD | 0 |
| Chordata | Actinopterygii | Syngnathiformes | Syngnathidae | Hippocampus | hippocampus | 0.83 | DD | 0 |
| Chordata | Actinopterygii | Syngnathiformes | Syngnathidae | Nerophis | ophidion | 0.41 | NL | 0 |
| Chordata | Actinopterygii | Syngnathiformes | Syngnathidae | Syngnathus | abaster | 0.84 | LC | 0 |
| Chordata | Actinopterygii | Syngnathiformes | Syngnathidae | Syngnathus | acus | 0.68 | NL | 0 |
| Chordata | Actinopterygii | Syngnathiformes | Syngnathidae | Syngnathus | typhle | 0.85 | NL | 0 |
| Chordata | Actinopterygii | Tetraodontiformes | Balistidae | Balistes | capriscus | 0.18 | NL | 1 |
| Chordata | Actinopterygii | Tetraodontiformes | Molidae | Mola | mola | 0.41 | NL | 1 |
| Chordata | Actinopterygii | Tetraodontiformes | Molidae | Ranzania | laevis | 0.46 | NL | 1 |
| Chordata | Actinopterygii | Tetraodontiformes | Ostraciidae | Acanthostracion | quadricornis | 0.24 | NL | 0 |
| Chordata | Actinopterygii | Tetraodontiformes | Ostraciidae | Lactophrys | trigonus | 0.31 | NL | 0 |
| Chordata | Actinopterygii | Tetraodontiformes | Tetraodontidae | Arothron | hispidus | 0.17 | NL | 0 |
| Chordata | Actinopterygii | Tetraodontiformes | Tetraodontidae | Ephippion | guttifer | 0.02 | NL | 0 |
| Chordata | Actinopterygii | Tetraodontiformes | Tetraodontidae | Sphoeroides | pachygaster | 0.23 | VU | 0 |
| Chordata | Actinopterygii | Tetraodontiformes | Tetraodontidae | Torquigener | flavimaculosus | 0.69 | NL | 0 |
| Chordata | Actinopterygii | Zeiformes | Zeidae | Zeus | faber | 0.5 | NL | 0 |
| Chordata | Ascidiacea | Enterogona | Ascidiidae | Ascidia | conchilega | 0.05 | NL | 0 |
| Chordata | Ascidiacea | Enterogona | Ascidiidae | Ascidia | mentula | 0.62 | NL | 0 |
| Chordata | Ascidiacea | Enterogona | Ascidiidae | Ascidia | virginea | 0.46 | NL | 0 |
| Chordata | Ascidiacea | Enterogona | Ascidiidae | Ascidiella | aspersa | 0.49 | NL | 0 |
| Chordata | Ascidiacea | Enterogona | Ascidiidae | Phallusia | mammillata | 0.8 | NL | 0 |
| Chordata | Ascidiacea | Enterogona | Cionidae | Ciona | intestinalis | 0.06 | NL | 0 |
| Chordata | Ascidiacea | Enterogona | Clavelinidae | Clavelina | lepadiformis | 0.04 | NL | 0 |
| Chordata | Ascidiacea | Enterogona | Corellidae | Corella | parallelogramma | 0.04 | NL | 0 |
| Chordata | Ascidiacea | Enterogona | Didemnidae | Didemnum | maculosum | 0.48 | NL | 0 |
| Chordata | Ascidiacea | Enterogona | Didemnidae | Diplosoma | listerianum | 0.17 | NL | 0 |
| Chordata | Ascidiacea | Enterogona | Polycitoridae | Cystodytes | dellechiajei | 0.24 | NL | 0 |
| Chordata | Ascidiacea | Enterogona | Polyclinidae | Polyclinum | aurantium | 0.25 | NL | 0 |
| Chordata | Ascidiacea | Enterogona | Polyclinidae | Sidnyum | turbinatum | 0.17 | NL | 0 |
| Chordata | Ascidiacea | Pleurogona | Molgulidae | Molgula | manhattensis | 0.13 | NL | 0 |
| Chordata | Ascidiacea | Pleurogona | Molgulidae | Molgula | occulta | 0.18 | NL | 0 |
| Chordata | Ascidiacea | Pleurogona | Pyuridae | Pyura | microcosmus | 0.72 | NL | 0 |
| Chordata | Ascidiacea | Pleurogona | Pyuridae | Pyura | tessellata | 0.84 | NL | 0 |
| Chordata | Ascidiacea | Pleurogona | Styelidae | Botrylloides | violaceus | 0.03 | NL | 0 |
| Chordata | Ascidiacea | Pleurogona | Styelidae | Botryllus | schlosseri | 0.15 | NL | 0 |
| Chordata | Ascidiacea | Pleurogona | Styelidae | Polycarpa | pomaria | 0.1 | NL | 0 |
| Chordata | Ascidiacea | Pleurogona | Styelidae | Styela | canopus | 0.41 | NL | 0 |
| Chordata | Ascidiacea | Pleurogona | Styelidae | Styela | plicata | 0.41 | NL | 0 |
| Chordata | Cephalaspidomorphi | Petromyzontiformes | Petromyzontidae | Lampetra | fluviatilis | 0.06 | LC | 0 |
| Chordata | Cephalaspidomorphi | Petromyzontiformes | Petromyzontidae | Petromyzon | marinus | 0.24 | LC | 0 |
| Chordata | Elasmobranchii | Carcharhiniformes | Carcharhinidae | Carcharhinus | brachyurus | 0.23 | DD | 0 |
| Chordata | Elasmobranchii | Carcharhiniformes | Carcharhinidae | Carcharhinus | brevipinna | 0.03 | DD | 0 |
| Chordata | Elasmobranchii | Carcharhiniformes | Carcharhinidae | Carcharhinus | falciformis | 0.13 | LR/lc | 0 |
| Chordata | Elasmobranchii | Carcharhiniformes | Carcharhinidae | Carcharhinus | limbatus | 0.27 | DD | 0 |
| Chordata | Elasmobranchii | Carcharhiniformes | Carcharhinidae | Carcharhinus | longimanus | 0.39 | VU | 1 |
| Chordata | Elasmobranchii | Carcharhiniformes | Carcharhinidae | Carcharhinus | melanopterus | 0.22 | LR/nt | 1 |
| Chordata | Elasmobranchii | Carcharhiniformes | Carcharhinidae | Carcharhinus | obscurus | 0.28 | DD | 0 |
| Chordata | Elasmobranchii | Carcharhiniformes | Carcharhinidae | Carcharhinus | plumbeus | 0.31 | EN | 0 |
| Chordata | Elasmobranchii | Carcharhiniformes | Carcharhinidae | Prionace | glauca | 0.23 | VU | 0 |
| Chordata | Elasmobranchii | Carcharhiniformes | Scyliorhinidae | Galeus | melastomus | 0.63 | LC | 0 |
| Chordata | Elasmobranchii | Carcharhiniformes | Scyliorhinidae | Scyliorhinus | canicula | 0.57 | LC | 1 |
| Chordata | Elasmobranchii | Carcharhiniformes | Scyliorhinidae | Scyliorhinus | stellaris | 0.34 | NT | 0 |
| Chordata | Elasmobranchii | Carcharhiniformes | Sphyrnidae | Sphyrna | lewini | 0.36 | LR/nt | 0 |
| Chordata | Elasmobranchii | Carcharhiniformes | Sphyrnidae | Sphyrna | mokarran | 0.15 | EN | 1 |
| Chordata | Elasmobranchii | Carcharhiniformes | Sphyrnidae | Sphyrna | tudes | 0.15 | VU | 1 |
| Chordata | Elasmobranchii | Carcharhiniformes | Sphyrnidae | Sphyrna | zygaena | 0.23 | VU | 0 |
| Chordata | Elasmobranchii | Carcharhiniformes | Triakidae | Galeorhinus | galeus | 0.39 | VU | 1 |
| Chordata | Elasmobranchii | Carcharhiniformes | Triakidae | Mustelus | asterias | 0.16 | LR/lc | 0 |
| Chordata | Elasmobranchii | Carcharhiniformes | Triakidae | Mustelus | mustelus | 0.49 | VU | 0 |
| Chordata | Elasmobranchii | Hexanchiformes | Hexanchidae | Heptranchias | perlo | 0.26 | VU | 0 |
| Chordata | Elasmobranchii | Hexanchiformes | Hexanchidae | Hexanchus | griseus | 0.19 | NT | 0 |
| Chordata | Elasmobranchii | Hexanchiformes | Hexanchidae | Hexanchus | nakamurai | 0.46 | DD | 0 |
| Chordata | Elasmobranchii | Lamniformes | Alopiidae | Alopias | superciliosus | 0.15 | DD | 1 |
| Chordata | Elasmobranchii | Lamniformes | Cetorhinidae | Cetorhinus | maximus | 0.28 | VU | 1 |
| Chordata | Elasmobranchii | Lamniformes | Lamnidae | Carcharodon | carcharias | 0.32 | EN | 1 |
| Chordata | Elasmobranchii | Lamniformes | Lamnidae | Isurus | oxyrinchus | 0.12 | CR | 1 |
| Chordata | Elasmobranchii | Lamniformes | Lamnidae | Isurus | paucus | 0.32 | VU | 1 |
| Chordata | Elasmobranchii | Lamniformes | Lamnidae | Lamna | nasus | 0.46 | CR | 1 |
| Chordata | Elasmobranchii | Lamniformes | Odontaspididae | Carcharias | taurus | 0.21 | CR | 1 |
| Chordata | Elasmobranchii | Lamniformes | Odontaspididae | Odontaspis | ferox | 0.25 | EN | 1 |
| Chordata | Elasmobranchii | Pristiformes | Pristidae | Pristis | pectinata | 0.21 | CR | 1 |
| Chordata | Elasmobranchii | Rajiformes | Dasyatidae | Dasyatis | centroura | 0.45 | NT | 0 |
| Chordata | Elasmobranchii | Rajiformes | Dasyatidae | Dasyatis | pastinaca | 0.79 | NT | 1 |
| Chordata | Elasmobranchii | Rajiformes | Dasyatidae | Dasyatis | tortonesei | 1 | N.E. | 0 |
| Chordata | Elasmobranchii | Rajiformes | Dasyatidae | Pteroplatytrygon | violacea | 0.22 | NT | 0 |
| Chordata | Elasmobranchii | Rajiformes | Dasyatidae | Taeniura | grabata | 0.01 | DD | 0 |
| Chordata | Elasmobranchii | Rajiformes | Gymnuridae | Gymnura | altavela | 0.21 | CR | 0 |
| Chordata | Elasmobranchii | Rajiformes | Myliobatidae | Myliobatis | aquila | 0.93 | NT | 0 |
| Chordata | Elasmobranchii | Rajiformes | Myliobatidae | Pteromylaeus | bovinus | 0.19 | DD | 0 |
| Chordata | Elasmobranchii | Rajiformes | Myliobatidae | Rhinoptera | marginata | 0.03 | NT | 0 |
| Chordata | Elasmobranchii | Rajiformes | Rajidae | Dipturus | batis | 0.42 | CR | 1 |
| Chordata | Elasmobranchii | Rajiformes | Rajidae | Dipturus | oxyrinchus | 0.59 | NT | 0 |
| Chordata | Elasmobranchii | Rajiformes | Rajidae | Leucoraja | circularis | 0.82 | EN | 0 |
| Chordata | Elasmobranchii | Rajiformes | Rajidae | Leucoraja | fullonica | 0.44 | DD | 0 |
| Chordata | Elasmobranchii | Rajiformes | Rajidae | Leucoraja | melitensis | 1 | CR | 0 |
| Chordata | Elasmobranchii | Rajiformes | Rajidae | Leucoraja | naevus | 0.59 | NT | 0 |
| Chordata | Elasmobranchii | Rajiformes | Rajidae | Raja | asterias | 0.75 | LC | 0 |
| Chordata | Elasmobranchii | Rajiformes | Rajidae | Raja | brachyura | 0.36 | DD | 0 |
| Chordata | Elasmobranchii | Rajiformes | Rajidae | Raja | clavata | 0.63 | NT | 1 |
| Chordata | Elasmobranchii | Rajiformes | Rajidae | Raja | miraletus | 0.55 | LC | 0 |
| Chordata | Elasmobranchii | Rajiformes | Rajidae | Raja | montagui | 0.62 | LC | 1 |
| Chordata | Elasmobranchii | Rajiformes | Rajidae | Raja | polystigma | 0.63 | NT | 0 |
| Chordata | Elasmobranchii | Rajiformes | Rajidae | Raja | radula | 0.42 | DD | 0 |
| Chordata | Elasmobranchii | Rajiformes | Rajidae | Raja | rondeleti | 1 | N.E. | 0 |
| Chordata | Elasmobranchii | Rajiformes | Rajidae | Raja | undulata | 0.69 | DD | 0 |
| Chordata | Elasmobranchii | Rajiformes | Rajidae | Rostroraja | alba | 0.78 | CR | 1 |
| Chordata | Elasmobranchii | Rajiformes | Rhinobatidae | Rhinobatos | cemiculus | 0.11 | EN | 1 |
| Chordata | Elasmobranchii | Rajiformes | Rhinobatidae | Rhinobatos | rhinobatos | 0.11 | EN | 1 |
| Chordata | Elasmobranchii | Squaliformes | Centrophoridae | Centrophorus | granulosus | 0.07 | VU | 1 |
| Chordata | Elasmobranchii | Squaliformes | Dalatiidae | Dalatias | licha | 0.18 | DD | 0 |
| Chordata | Elasmobranchii | Squaliformes | Echinorhinidae | Echinorhinus | brucus | 0.22 | DD | 0 |
| Chordata | Elasmobranchii | Squaliformes | Etmopteridae | Etmopterus | spinax | 0.54 | LC | 0 |
| Chordata | Elasmobranchii | Squaliformes | Oxynotidae | Oxynotus | centrina | 0.23 | CR | 0 |
| Chordata | Elasmobranchii | Squaliformes | Somniosidae | Centroscymnus | coelolepis | 0.25 | LC | 0 |
| Chordata | Elasmobranchii | Squaliformes | Somniosidae | Somniosus | rostratus | 0.76 | LC | 0 |
| Chordata | Elasmobranchii | Squaliformes | Squalidae | Squalus | acanthias | 0.15 | EN | 1 |
| Chordata | Elasmobranchii | Squaliformes | Squalidae | Squalus | blainville | 0.53 | N.E. | 0 |
| Chordata | Elasmobranchii | Squaliformes | Squalidae | Squalus | uyato | 0.17 | N.E. | 0 |
| Chordata | Elasmobranchii | Squatiniformes | Squatinidae | Squatina | aculeata | 0.04 | CR | 1 |
| Chordata | Elasmobranchii | Squatiniformes | Squatinidae | Squatina | oculata | 0.05 | CR | 1 |
| Chordata | Elasmobranchii | Squatiniformes | Squatinidae | Squatina | squatina | 0.67 | CR | 1 |
| Chordata | Elasmobranchii | Torpediniformes | Torpedinidae | Torpedo | marmorata | 0.56 | LC | 0 |
| Chordata | Elasmobranchii | Torpediniformes | Torpedinidae | Torpedo | nobiliana | 0.22 | DD | 0 |
| Chordata | Elasmobranchii | Torpediniformes | Torpedinidae | Torpedo | torpedo | 0.37 | LC | 0 |
| Chordata | Holocephali | Chimaeriformes | Chimaeridae | Chimaera | monstrosa | 0.44 | NT | 1 |
| Chordata | Mammalia | Carnivora | Phocidae | Monachus | monachus | 0.81 | CR | 1 |
| Chordata | Mammalia | Cetacea | Balaenopteridae | Balaenoptera | acutorostrata | 0.24 | LR/nt | 1 |
| Chordata | Mammalia | Cetacea | Balaenopteridae | Balaenoptera | borealis | 0 | EN | 1 |
| Chordata | Mammalia | Cetacea | Balaenopteridae | Balaenoptera | physalus | 0.17 | EN | 1 |
| Chordata | Mammalia | Cetacea | Balaenopteridae | Megaptera | novaeangliae | 0.2 | VU | 1 |
| Chordata | Mammalia | Cetacea | Delphinidae | Delphinus | delphis | 0.22 | LR/lc | 1 |
| Chordata | Mammalia | Cetacea | Delphinidae | Globicephala | melas | 0.16 | LR/lc | 1 |
| Chordata | Mammalia | Cetacea | Delphinidae | Grampus | griseus | 0.32 | DD | 1 |
| Chordata | Mammalia | Cetacea | Delphinidae | Orcinus | orca | 0.3 | LR/cd | 1 |
| Chordata | Mammalia | Cetacea | Delphinidae | Pseudorca | crassidens | 0.25 | LR/lc | 1 |
| Chordata | Mammalia | Cetacea | Delphinidae | Stenella | coeruleoalba | 0.64 | LR/cd | 1 |
| Chordata | Mammalia | Cetacea | Delphinidae | Steno | bredanensis | 0.19 | DD | 1 |
| Chordata | Mammalia | Cetacea | Delphinidae | Tursiops | truncatus | 0.39 | DD | 1 |
| Chordata | Mammalia | Cetacea | Kogiidae | Kogia | sima | 0.09 | LR/lc | 1 |
| Chordata | Mammalia | Cetacea | Phocoenidae | Phocoena | phocoena | 0.05 | VU | 1 |
| Chordata | Mammalia | Cetacea | Physeteridae | Physeter | catodon | 0.26 | VU | 1 |
| Chordata | Mammalia | Cetacea | Ziphiidae | Hyperoodon | ampullatus | 0 | LR/cd | 1 |
| Chordata | Mammalia | Cetacea | Ziphiidae | Mesoplodon | bidens | 0.02 | DD | 0 |
| Chordata | Mammalia | Cetacea | Ziphiidae | Mesoplodon | densirostris | 0.09 | DD | 0 |
| Chordata | Mammalia | Cetacea | Ziphiidae | Ziphius | cavirostris | 0.24 | DD | 1 |
| Chordata | Myxini | Myxiniformes | Myxinidae | Myxine | glutinosa | 0.36 | NL | 0 |
| Chordata | Reptilia | Testudines | Cheloniidae | Caretta | caretta | 0.49 | EN | 1 |
| Chordata | Reptilia | Testudines | Cheloniidae | Chelonia | mydas | 0.36 | EN | 1 |
| Chordata | Reptilia | Testudines | Cheloniidae | Eretmochelys | imbricata | 0.18 | CR | 1 |
| Chordata | Reptilia | Testudines | Cheloniidae | Lepidochelys | kempii | 0.22 | CR | 1 |
| Chordata | Reptilia | Testudines | Dermochelyidae | Dermochelys | coriacea | 0.26 | CR | 1 |
| Cnidaria | Anthozoa | Actiniaria | Aiptasiidae | Aiptasia | mutabilis | 0.53 | NL | 0 |
| Cnidaria | Anthozoa | Scleractinia | Caryophylliidae | Cladocora | caespitosa | 0.61 | NL | 0 |
| Cnidaria | Anthozoa | Scleractinia | Caryophylliidae | Lophelia | pertusa | 0.25 | NL | 0 |
| Cnidaria | Hydrozoa | Anthoathecata | Eudendriidae | Eudendrium | capillare | 0.51 | NL | 0 |
| Cnidaria | Hydrozoa | Anthoathecata | Eudendriidae | Eudendrium | carneum | 0.33 | NL | 0 |
| Cnidaria | Hydrozoa | Anthoathecata | Eudendriidae | Eudendrium | rameum | 0.21 | NL | 0 |
| Cnidaria | Hydrozoa | Leptothecata | Lafoeidae | Lafoea | dumosa | 0.07 | NL | 0 |
| Cnidaria | Scyphozoa | Semaeostomeae | Ulmaridae | Aurelia | aurita | 0.17 | NL | 0 |
| Ctenophora | Tentaculata | Cydippida | Pleurobrachiidae | Pleurobrachia | pileus | 0.07 | NL | 0 |
| Echinodermata | Holothuroidea | Aspidochirotida | Stichopodidae | Stichopus | regalis | 0.55 | NL | 0 |
| Echinodermata | Holothuroidea | Dendrochirotida | Cucumariidae | Leptopentacta | elongata | 0.58 | NL | 0 |
| Mollusca | Bivalvia | Arcoida | Arcidae | Arca | noae | 0.94 | NL | 0 |
| Mollusca | Bivalvia | Arcoida | Arcidae | Arca | tetragona | 0.79 | NL | 0 |
| Mollusca | Bivalvia | Arcoida | Arcidae | Barbatia | barbata | 0.85 | NL | 0 |
| Mollusca | Bivalvia | Arcoida | Glycymerididae | Glycymeris | bimaculata | 0.88 | NL | 0 |
| Mollusca | Bivalvia | Arcoida | Noetiidae | Striarca | lactea | 0.78 | NL | 0 |
| Mollusca | Bivalvia | Limoida | Limidae | Lima | lima | 0.81 | NL | 0 |
| Mollusca | Bivalvia | Limoida | Limidae | Limatula | subauriculata | 0.31 | NL | 0 |
| Mollusca | Bivalvia | Myoida | Corbulidae | Corbula | gibba | 0.21 | NL | 0 |
| Mollusca | Bivalvia | Myoida | Teredinidae | Teredo | navalis | 0.47 | NL | 0 |
| Mollusca | Bivalvia | Mytiloida | Mytilidae | Gregariella | petagnae | 0.81 | NL | 0 |
| Mollusca | Bivalvia | Mytiloida | Mytilidae | Lithophaga | lithophaga | 0.83 | NL | 0 |
| Mollusca | Bivalvia | Mytiloida | Mytilidae | Modiolula | phaseolina | 0.75 | NL | 0 |
| Mollusca | Bivalvia | Mytiloida | Mytilidae | Modiolus | adriaticus | 0.78 | NL | 0 |
| Mollusca | Bivalvia | Mytiloida | Mytilidae | Modiolus | barbatus | 0.81 | NL | 0 |
| Mollusca | Bivalvia | Mytiloida | Mytilidae | Musculus | costulatus | 0.92 | NL | 0 |
| Mollusca | Bivalvia | Mytiloida | Mytilidae | Mytilus | galloprovincialis | 0.77 | NL | 0 |
| Mollusca | Bivalvia | Nuculoida | Nuculidae | Nucula | hanleyi | 0.17 | NL | 0 |
| Mollusca | Bivalvia | Nuculoida | Nuculidae | Nucula | nucleus | 0.32 | NL | 0 |
| Mollusca | Bivalvia | Nuculoida | Nuculidae | Nucula | sulcata | 0.43 | NL | 0 |
| Mollusca | Bivalvia | Ostreoida | Anomiidae | Anomia | ephippium | 0.69 | NL | 0 |
| Mollusca | Bivalvia | Ostreoida | Anomiidae | Pododesmus | patelliformis | 0.42 | NL | 0 |
| Mollusca | Bivalvia | Ostreoida | Ostreidae | Crassostrea | gigas | 0.21 | NL | 0 |
| Mollusca | Bivalvia | Ostreoida | Ostreidae | Ostrea | edulis | 0.28 | NL | 1 |
| Mollusca | Bivalvia | Ostreoida | Pectinidae | Aequipecten | opercularis | 0.51 | NL | 0 |
| Mollusca | Bivalvia | Ostreoida | Pectinidae | Chlamys | glabra | 0.88 | NL | 0 |
| Mollusca | Bivalvia | Ostreoida | Pectinidae | Chlamys | pesfelis | 0.73 | NL | 0 |
| Mollusca | Bivalvia | Ostreoida | Pectinidae | Chlamys | varia | 0.66 | NL | 0 |
| Mollusca | Bivalvia | Ostreoida | Pectinidae | Delectopecten | vitreus | 0.04 | NL | 0 |
| Mollusca | Bivalvia | Ostreoida | Spondylidae | Spondylus | gaederopus | 0.79 | NL | 0 |
| Mollusca | Bivalvia | Pholadomyoida | Pandoridae | Pandora | inaequivalvis | 0.66 | NL | 0 |
| Mollusca | Bivalvia | Pterioida | Pinnidae | Atrina | pectinata | 0.32 | NL | 0 |
| Mollusca | Bivalvia | Pterioida | Pinnidae | Pinna | nobilis | 0.86 | NL | 0 |
| Mollusca | Bivalvia | Veneroida | Astartidae | Digitaria | digitaria | 0.8 | NL | 0 |
| Mollusca | Bivalvia | Veneroida | Cardiidae | Acanthocardia | aculeata | 0.88 | NL | 0 |
| Mollusca | Bivalvia | Veneroida | Cardiidae | Acanthocardia | paucicostata | 0.73 | NL | 0 |
| Mollusca | Bivalvia | Veneroida | Cardiidae | Acanthocardia | tuberculata | 0.75 | NL | 0 |
| Mollusca | Bivalvia | Veneroida | Cardiidae | Cerastoderma | edule | 0.01 | NL | 0 |
| Mollusca | Bivalvia | Veneroida | Cardiidae | Cerastoderma | glaucum | 0.52 | NL | 0 |
| Mollusca | Bivalvia | Veneroida | Cardiidae | Laevicardium | crassum | 0.05 | NL | 0 |
| Mollusca | Bivalvia | Veneroida | Donacidae | Donax | semistriatus | 0.88 | NL | 0 |
| Mollusca | Bivalvia | Veneroida | Donacidae | Donax | trunculus | 0.82 | NL | 0 |
| Mollusca | Bivalvia | Veneroida | Donacidae | Donax | vittatus | 0.03 | NL | 0 |
| Mollusca | Bivalvia | Veneroida | Lucinidae | Anodontia | fragilis | 0.83 | NL | 0 |
| Mollusca | Bivalvia | Veneroida | Lucinidae | Ctena | decussata | 0.8 | NL | 0 |
| Mollusca | Bivalvia | Veneroida | Lucinidae | Loripes | lacteus | 0.94 | NL | 0 |
| Mollusca | Bivalvia | Veneroida | Lucinidae | Lucinella | divaricata | 0.82 | NL | 0 |
| Mollusca | Bivalvia | Veneroida | Lucinidae | Myrtea | spinifera | 0.85 | NL | 0 |
| Mollusca | Bivalvia | Veneroida | Mesodesmatidae | Paphies | australis | 0.08 | NL | 0 |
| Mollusca | Bivalvia | Veneroida | Petricolidae | Mysia | undata | 0.07 | NL | 0 |
| Mollusca | Bivalvia | Veneroida | Pharidae | Ensis | siliqua | 0.1 | NL | 0 |
| Mollusca | Bivalvia | Veneroida | Psammobiidae | Gari | depressa | 0.85 | NL | 0 |
| Mollusca | Bivalvia | Veneroida | Semelidae | Scrobicularia | plana | 0.29 | NL | 0 |
| Mollusca | Bivalvia | Veneroida | Tellinidae | Tellina | tenuis | 0.68 | NL | 0 |
| Mollusca | Bivalvia | Veneroida | Veneridae | Callista | chione | 0.77 | NL | 0 |
| Mollusca | Bivalvia | Veneroida | Veneridae | Chamelea | gallina | 0.45 | NL | 0 |
| Mollusca | Bivalvia | Veneroida | Veneridae | Clausinella | fasciata | 0.62 | NL | 0 |
| Mollusca | Bivalvia | Veneroida | Veneridae | Dosinia | exoleta | 0.49 | NL | 0 |
| Mollusca | Bivalvia | Veneroida | Veneridae | Dosinia | lupinus | 0.57 | NL | 0 |
| Mollusca | Bivalvia | Veneroida | Veneridae | Ruditapes | decussatus | 0.87 | NL | 0 |
| Mollusca | Bivalvia | Veneroida | Veneridae | Tapes | decussata | 0.79 | NL | 0 |
| Mollusca | Bivalvia | Veneroida | Veneridae | Venerupis | pullastra | 0.66 | NL | 0 |
| Mollusca | Bivalvia | Veneroida | Veneridae | Venus | verrucosa | 0.7 | NL | 0 |
| Mollusca | Cephalopoda | Octopoda | Octopodidae | Eledone | cirrhosa | 0.39 | NL | 0 |
| Mollusca | Cephalopoda | Octopoda | Octopodidae | Eledone | moschata | 0.56 | NL | 0 |
| Mollusca | Cephalopoda | Octopoda | Octopodidae | Illex | coindetii | 0.63 | NL | 0 |
| Mollusca | Cephalopoda | Octopoda | Octopodidae | Octopus | vulgaris | 0.59 | NL | 0 |
| Mollusca | Cephalopoda | Sepiida | Sepiidae | Sepia | orbignyana | 0.64 | NL | 0 |
| Mollusca | Cephalopoda | Sepiolida | Sepiolidae | Heteroteuthis | dispar | 0.69 | NL | 0 |
| Mollusca | Cephalopoda | Sepiolida | Sepiolidae | Rossia | macrosoma | 0.03 | NL | 0 |
| Mollusca | Cephalopoda | Sepiolida | Sepiolidae | Stoloteuthis | leucoptera | 0.06 | NL | 1 |
| Mollusca | Cephalopoda | Teuthida | Brachioteuthidae | Brachioteuthis | riisei | 0.53 | NL | 0 |
| Mollusca | Cephalopoda | Teuthida | Loliginidae | Loligo | forbesii | 0.33 | NL | 1 |
| Mollusca | Cephalopoda | Teuthida | Loliginidae | Loligo | vulgaris | 0.65 | NL | 1 |
| Mollusca | Cephalopoda | Teuthida | Ommastrephidae | Ornithoteuthis | antillarum | 0.25 | NL | 0 |
| Mollusca | Cephalopoda | Teuthida | Ommastrephidae | Todarodes | sagittatus | 0.57 | NL | 0 |
| Mollusca | Cephalopoda | Teuthida | Ommastrephidae | Todaropsis | eblanae | 0.13 | NL | 0 |
| Mollusca | Cephalopoda | Teuthida | Pyroteuthidae | Pterygioteuthis | giardi | 0.19 | NL | 0 |
| Mollusca | Gastropoda | Archaeogastropoda | Haliotididae | Haliotis | tuberculata | 0.78 | NL | 0 |
| Mollusca | Gastropoda | Archaeogastropoda | Patellidae | Patella | ulyssiponensis | 0.6 | NL | 0 |
| Mollusca | Gastropoda | Archaeogastropoda | Trochidae | Gibbula | magus | 0.49 | NL | 0 |
| Mollusca | Gastropoda | Cephalaspidea | Cylichnidae | Cylichna | cylindracea | 0.28 | NL | 0 |
| Mollusca | Gastropoda | Cephalaspidea | Philinidae | Philine | aperta | 0.59 | NL | 0 |
| Mollusca | Gastropoda | Neogastropoda | Muricidae | Bolinus | brandaris | 0.77 | NL | 0 |
| Mollusca | Gastropoda | Neogastropoda | Muricidae | Stramonita | haemastoma | 0.67 | NL | 0 |
| Mollusca | Gastropoda | Neotaenioglossa | Eulimidae | Eulima | glabra | 0.72 | NL | 0 |
| Mollusca | Gastropoda | Neotaenioglossa | Ranellidae | Charonia | lampas | 0.52 | NL | 0 |
| Mollusca | Gastropoda | Neotaenioglossa | Turritellidae | Turritella | communis | 0.43 | NL | 0 |
| Mollusca | Gastropoda | Notaspidea | Pleurobranchidae | Berthella | plumula | 0.78 | NL | 0 |
| Mollusca | Polyplacophora | Chitonida | Acanthochitonidae | Acanthochitona | fascicularis | 0.89 | NL | 0 |
| Mollusca | Polyplacophora | Chitonida | Callochitonidae | Callochiton | septemvalvis | 0.02 | NL | 0 |
| Mollusca | Polyplacophora | Chitonida | Hanleyidae | Hanleya | hanleyi | 0.05 | NL | 0 |
| Mollusca | Scaphopoda | Dentaliida | Dentaliidae | Antalis | vulgaris | 0.69 | NL | 0 |
| Mollusca | Scaphopoda | Dentaliida | Fustiariidae | Fustiaria | rubescens | 0.82 | NL | 0 |
| Mollusca | Scaphopoda | Gadilida | Entalinidae | Entalina | tetragona | 0.51 | NL | 0 |
| Mollusca | Scaphopoda | Gadilida | Gadilidae | Cadulus | jeffreysi | 0.68 | NL | 0 |
| Mollusca | Scaphopoda | Gadilida | Gadilidae | Cadulus | propinquus | 0.58 | NL | 0 |
| Mollusca | Scaphopoda | Gadilida | Gadilidae | Dischides | politus | 0.8 | NL | 0 |
| Mollusca | Scaphopoda | Gadilida | Pulsellidae | Pulsellum | lofotense | 0.52 | NL | 0 |
| Porifera | Demospongiae | Dictyoceratida | Spongiidae | Spongia | officinalis | 0.6 | NL | 0 |
| Sipuncula | Not assigned | Not assigned | Aspidosiphonidae | Aspidosiphon | muelleri | 0.28 | NL | 0 |
| Sipuncula | Not assigned | Not assigned | Golfingiidae | Golfingia | elongata | 0.31 | NL | 0 |
| Sipuncula | Not assigned | Not assigned | Golfingiidae | Golfingia | margaritacea | 0.16 | NL | 0 |
| Sipuncula | Not assigned | Not assigned | Golfingiidae | Golfingia | vulgaris | 0.31 | NL | 0 |
| Sipuncula | Not assigned | Not assigned | Golfingiidae | Nephasoma | abyssorum | 0.29 | NL | 0 |
| Sipuncula | Not assigned | Not assigned | Golfingiidae | Nephasoma | constricticervix | 0.08 | NL | 0 |
| Sipuncula | Not assigned | Not assigned | Golfingiidae | Nephasoma | constrictum | 0.38 | NL | 0 |
| Sipuncula | Not assigned | Not assigned | Golfingiidae | Thysanocardia | procera | 0.52 | NL | 0 |
| Sipuncula | Not assigned | Not assigned | Phascolionidae | Phascolion | strombus | 0.45 | NL | 0 |
| Sipuncula | Not assigned | Not assigned | Phascolionidae | Phascolion | tuberculosum | 0.22 | NL | 0 |
| Sipuncula | Not assigned | Not assigned | Sipunculidae | Sipunculus | nudus | 0.27 | NL | 0 |

## **Seaweeds and seagrasses (by Enric Ballesteros)**

## **Table S7. Mediterranean biodiversity (species/infraspecific taxa, families, orders, classes) for the phyla Heterokontophyta, Rhodophyta, Chlorophyta and Magnoliophyta and all the macrophytobenthos**

Total number of species/intraspecific taxa is also split into introduced, endemics and others.

## **Table S8. Percentage of introduced, endemics and other macrophytobenthic species/infraspecific taxa by phylum and totals**

## **Table S9. Checklist of the phylum Heterokontophyta and comments to the checklist**

Algae belonging to the phylum Heterokontophyta (Kingdom Chromista) have been classified by Classes, Orders, Families, Species and other infraspecific levels (subspecies, varieties and forms). Ordination is alphabetical. When no infraspecific level is indicated the taxa corresponds to the type variety.

This list is based in the check-list by Ribera et al. (1992), with some updates and modifications. Particular attention has been devoted to local checklists published after the revision by Ribera et al. (1992): Puglia (Southern Italy) (Cormaci et al. 2001), Tuscany (Northwestern Italy) (Rindi et al. 2002), Morocco (Benhissoune et al. 2002) and Italian coasts (Furnari et al. 2003). Lists of Mediterranean introduced species have also been considered (e.g. Boudouresque & Verlaque 2002; Zenetos et al. 2005, 2008; Verlaque et al. in press) as well as some specific papers (e.g. Cormaci et al. 1994; Ramon 2000; Ribera et al. 2005; Alongi et al. 2007; Taskin et al. 2010). Black Sea is not included.

Taxonomy follows Algaebase (www.algaebase.org) if not otherwise indicated. Endemic species (found only in the Mediterranean basin, Black Sea included) are preceded by the symbol *. A careful and critical examination of the existing records of every species from the available literature has been performed in order to consider a species as endemic. Introduced species are preceded by the symbol #. As it is not always easy to detect introduced species from the distributional records, we mainly follow the criteria by other authors specialized in this issue. Superscript numbers refer to notes. Some of the commonest synonyms found in Mediterranean literature (if any) are sometimes placed after the currently accepted name. Other synonyms can be found at Algaebase. A list of *taxa inquirenda* as well as a list of *taxa excludenda* is also given at the end of the list; species considered in Ribera et al. (1992) are not stated again.

| **Kingdom Chromista** |
| --- |
|  |
| **Phylum Heterokontophyta** |
|  |
| **Class Pelagophyceae** |
|  |
| *Order Sarcinochrysidales* |
|  |
| Family Sarcinochrysidaceae |
| *Chrysonephos lewisii* (Taylor) Taylor |
| *Nematochrysopsis marina* (J. Feldmann) Billard (=*Tribonema marinum* J. Feldmann) |
|  |
| **Class Xanthophyceae** |
|  |
| *Order Vaucheriales* |
|  |
| Family Vaucheriaceae |
| *Vaucheria dichotoma* (Linnaeus) Martius |
| *Vaucheria piloboloides* Thuret |
| *Vaucheria sescuplicaria* Christensen |
| *Vaucheria submarina* (Lyngbye) Berkeley |
| *Vaucheria synandra* Woronin |
| *Vaucheria velutina* C. Agardh (=*Vaucheria thuretii* Woronin) |
| *Vaucheria woroniniana* Heering |
|  |
| **Class Phaeophyceae** |
|  |
| *Order Cutleriales* |
|  |
| Family Cutleriaceae |
| *Cutleria adspersa* (Mertens ex Roth) De Notaris (=*Aglaozonia melanoidea* Sauvageau) |
| *Cutleria chilosa* (Falkenberg) Silva (=*Aglaozonia chilosa* Falkenberg; =*Cutleria monoica* Ollivier) |
| *Cutleria multifida* (Turner) Greville [=*Aglaozonia parvula* (Greville) Zanardini] |
| *Zanardinia typus* (Nardo) Silva [=*Zanardinia prototypus* (Nardo) Nardo] |
|  |
| *Order Desmarestiales* |
|  |
| Family Arthrocladiaceae |
| *Arthrocladia villosa* (Hudson) Duby |
|  |
| Family Desmarestiaceae |
| *Desmarestia aculeata* (Linnaeus) Lamouroux |
| *Desmarestia dresnayi* Lamouroux ex Leman |
| *Desmarestia ligulata* (Stackhouse) Lamouroux (=*Desmarestia adriatica* Ercegovic) |
| *#Desmarestia viridis* (O.F. Müller) Lamouroux |
|  |
| *Order Dictyotales* |
|  |
| Family Dictyotaceae |
| *Dictyopteris lucida* Ribera, Gómez-Garreta, Pérez-Ruzafa, Barceló & Rull |
| *Dictyopteris polypodioides* (De Candolle) Lamouroux [=*Dictyopteris membranacea* (Stackhouse) Batters] |
| *Dictyota dichotoma* (Hudson) Lamouroux |
| *Dictyota dichotoma* var. *intricata* (C. Agardh) Greville [=var. *implexa* (Desfontaines) Gray] |
| *Dictyota fasciola* (Roth) Lamouroux [=*Dilophus fasciola* (Roth) Howe] |
| *Dictyota fasciola* var. *repens* (J. Agardh) Ardissone |
| *Dictyota linearis* (C. Agardh) Greville |
| **Dictyota mediterranea* (Schiffner) Furnari (=*Dilophus mediterraneus* Schiffner) |
| *#Dictyota* sp.^1^ |
| *Dictyota spiralis* Montagne [=*Dilophus ligulatus* (Kützing) J. Feldmann] |
| *Lobophora variegata* (Lamouroux) Womersley ex Oliveira |
| *#Padina boergesenii* Allender & Kraft |
| *#Padina boryana* Thivy (=*Padina tenuis* Bory) |
| *Padina pavonica* (Linnaeus) Thivy |
| *#Rugulopteryx okamurae* (Dawson) Hwang, Lee & Kim [=*Dictyota okamurae* (Dawson) Hörnig, Schnetter & Prud'homme van Reine] |
| *Spatoglossum schroederi* (C. Agardh) Kützing |
| *Spatoglossum solierii* (Chauvin ex Montagne) Kützing |
| *#Spatoglossum variabile* Figari & De Notaris |
| *#Stypopodium schimperi* (Buchinger ex Kützing) Verlaque & Boudouresque |
| *Taonia atomaria* (Woodward) J. Agardh |
| **Taonia atomaria* f. *ciliata* (C. Agardh) Nizamuddin |
| **Taonia lacheana* Cormaci, Furnari & Pizzuto |
| *Zonaria tournefortii* (Lamouroux) Montagne |
|  |
| *Order Ectocarpales* |
|  |
| Family Acinetosporaceae |
| *Acinetospora crinita* (Carmichael ex Harvey) Sauvageau [=*Acinetospora vidovichii* (Meneghini) Sauvageau] |
| **Feldmannia battersiides* (Ercegovic) Cormaci & Furnari |
| *Feldmannia irregularis* (Kützing) Hamel |
| *Feldmannia lebelii* (Areschoug ex P.L. Crouan & H.M. Crouan) Hamel [=*Feldmannia caespitula* (J. Agardh) Knoepffler-Péguy] |
| *Feldmannia padinae* (Buffham) Hamel |
| *Feldmannia paradoxa* (Montagne) Hamel [=*Feldmannia globifera* (Kützing) Hamel] |
| **Feldmannia paradoxoides* (Ercegovic) Cormaci & Furnari |
| *Feldmannia simplex* (P.L. Crouan & H.M. Crouan*) Hamel* |
| **Hincksia dalmatica* (Ercegovic) Cormaci & Furnari |
| *Hincksia fuscata* (Zanardini) Silva |
| **Hincksia geniculata* (Ercegovic) Cormaci & Furnari |
| *Hincksia granulosa* (Smith) Silva |
| **Hincksia hauckii* (Ercegovic) Cormaci & Furnari |
| *Hincksia hincksiae* (Harvey) Silva |
| *Hincksia mitchelliae* (Harvey) Silva |
| *Hincksia ovata* (Kjellman) Silva |
| *Hincksia sandriana* (Zanardini) Silva |
| *Hincksia secunda* (Kützing) Silva |
|  |
| Family Chordariaceae |
| **Acrospongium ralfsioides* Schiffner |
| *#Acrothrix gracilis* Kylin |
| *Ascocyclus orbicularis* (J. Agardh) Kjellman^2^ |
| *Asperococcus bullosus* Lamouroux [=*Asperococcus turneri* (Smith) Hooker] |
| **Asperococcus bullosus* f. *profundus* J. Feldmann |
| *Asperococcus ensiformis* (Chiaje) Wynne (=*Asperococcus compressus* Griffiths ex Hooker) |
| *Asperococcus fistulosus* (Hudson) Hooker |
| *Asperococcus scaber* Kuckuck |
| *Botrytella micromora* Bory |
| *#Botrytella parva* (Takamatsu) Kim |
| *Chilionema hispanicum* (Sauvageau) Fletcher |
| *Cladosiphon contortus* (Thuret) Kylin |
| **Cladosiphon cylindricus* (Sauvageau) Kylin (=*Castagnea cylindrica* Sauvageau) |
| **Cladosiphon irregularis* (Sauvageau) Kylin (=*Castagnea irregularis* Sauvageau) |
| **Cladosiphon mediterraneus* Kützing (=*Castagnea mediterranea* (Kützing) Hauck) |
| *#Cladosiphon zosterae* (J. Agardh) Kylin |
| *Climacosorus mediterraneus* Sauvageau |
| *Corynophlaea crispa* (Harvey) Kuckuck |
| **Corynophlaea flaccida* (C. Agardh) Kützing |
| **Corynophlaea hamelii* J. Feldmann |
| **Corynophlaea umbellata* (C. Agardh) Kützing |
| **Cylindrocarpus kuckuckii* Taskin, Wynne & Özturk |
| *Cylindrocarpus microscopicus* P.L. Crouan & H.M. Crouan |
| *Elachista flaccida* (Dillwyn) Fries |
| *Elachista fucicola* (Velley) Areschoug |
| *Elachista intermedia* P.L. Crouan & H.M. Crouan |
| **Elachista intermedia* var. *clavaeformis* Ercegovic |
| **Elachista jabukae* Ercegovic |
| **Elachista neglecta* Kuckuck |
| *Elachista stellaris* Areschoug |
| *Eudesme virescens* (Carmichael ex Berkeley) J. Agardh |
| *Giraudia sphacelarioides* Derbès & Solier |
| **Gontrania lubrica* Sauvageau |
| *#Halothrix lumbricalis* (Kützing) Reinke |
| **Hecatonema liagorae* (J. Feldmann) Hamel |
| *Hecatonema terminale* (Kützing) Kylin [=*Hecatonema maculans* (Collins) Sauvageau] |
| **Herponema graniferum* Kuckuck |
| *Herponema solitarium* (Sauvageau) Hamel |
| *Herponema valiantei* (Bornet ex Sauvageau) Hamel |
| *Herponema velutinum* (Greville) J. Agardh |
| *Kuetzingiella battersii* (Bornet ex Sauvageau) Kornmann |
| **Kuetzingiella battersii* var. *mediterranea* (Sauvageau) Gómez & Ribera |
| *#Leathesia marina* (Lyngbye) Decaisne (=*Leathesia difformis* Areschoug) |
| **Leathesia mucosa* J. Feldmann |
| **Leathesia mucosa* var. *condensata* J. Feldmann |
| *Leptonematella fasciculata* (Reinke) Silva |
| **Leptonematella neapolitana* (Schussnig) Cormaci & Furnari |
| *Liebmannia leveillei* J. Agardh |
| *Lithosiphon laminariae* (Lyngbye) Harvey (=*Streblonema oligosporum* Strömfelt; =*Streblonema thuretii* Sauvageau) |
| *Mesogloia lanosa* P.L. Crouan & H.M. Crouan |
| *Mesogloia vermiculata* (Smith) Gray |
| *Microcoryne ocellata* Strömfelt |
| *Microspongium gelatinosum* Reinke |
| *Microspongium tenuissimum* (Hauck) A.F. Peters (=*Streblonema tenuissimum* Hauck) |
| *Mikrosyphar polysiphoniae* Kuckuck |
| *Myriactula arabica* (Kützing) J. Feldmann |
| **Myriactula elongata* (Sauvageau) Hamel |
| **Myriactula gracilis* van der Ben |
| **Myriactula rigida* (Sauvageau) Hamel |
| *Myriactula rivulariae* (Suhr) J. Feldmann |
| *Myriactula stellulata* (Harvey) Levring |
| *#Myriogloea sciurus* (Harvey) Kuckuck ex Oltmanns |
| **Myrionema conchicola* (J. Feldmann) Boudouresque |
| *Myrionema liechtensternii* Hauck |
| *Myrionema magnusii* (Sauvageau) Loiseaux |
| *Myrionema strangulans* Greville |
| *Myriotrichia adriatica* Hauck |
| *Myriotrichia repens* Hauck (=*Myriotrichia clavaeformis* Harvey?) |
| **Nemacystus flexuosus* (C. Agardh) Kylin var. *giraudyi* (J. Agardh) De Jong (=*Nemacystus ramulosus* Derbès & Solier) |
| *Nemacystus hispanicus* (Sauvageau) Kylin |
| *Petrospongium berkeleyi* (Greville) Nägeli ex Kützing |
| **Phaeostroma bertholdii* Kuckuck |
| **Protasperococcus myriotrichiiformis* Sauvageau |
| *Protectocarpus speciosus* (Boergesen) Kornmann |
| *Punctaria latifolia* Greville |
| *#Punctaria tenuissima* (C. Agardh) Greville (=*Streblonema effusum* Kylin) |
| *Sauvageaugloia griffithsiana* (Griffiths & Harvey) Hamel & Kylin *(=Myriocladia chordariaeformis* P.L. Crouan & H.M. Crouan; =*Cladosiphon chordariaeformis* P.L. Crouan & H.M. Crouan) |
| *Spermatochnus paradoxus* (Roth) Kützing |
| *Spongonema tomentosum* Hudson) Kützing |
| *Stictyosiphon adriaticus* Kützing |
| *Stictyosiphon soriferus* (Reinke) Rosenvinge |
| *Stictyosiphon tortilis* (Gobi) Reinke |
| *Stilophora tenella* (Esper) Silva [=*Stilophora rhizodes* (C. Agardh) J. Agardh] |
| *Streblonema infestans* (Gran) Batters |
| *Streblonema parasiticum* (Sauvageau) De Toni |
| *Streblonema sphaericum* (Derbès & Solier) Thuret |
| *Streblonema stilophorae* (P.L. Crouan & H.M. Crouan) Kylin |
| **Streblonemopsis irritans* Valiante |
| *Sphaerotrichia divaricata* (C. Agardh) Kylin |
| *#Sphaerotrichia firma* (Gepp) Zinova |
| *Strepsithalia liagorae* Sauvageau |
| *Strepsithalia liebmanniae* Miranda |
| *Striaria attenuata* (Greville) Greville [=*Striaria attenuata* f. *crinita* (J. Agardh) Hauck; =*Striaria attenuata* f. *ramosissima* (Kützing) Hauck] |
| **Zosterocarpus oedogonium* (Meneghini) Bornet |
|  |
| Family Ectocarpaceae |
| *Asterocladon rhodochortonoides* (Boergesen) Uwai, Nagasato, Motomura & Kogame (=*Ectocarpus rhodochortonoides* Boergesen) |
| *Ectocarpus fasciculatus* Harvey |
| *Ectocarpus fasciculatus* var. *abbreviatus* (Kützing) Sauvageau |
| *Ectocarpus fasciculatus* var. *pycnocarpus* (Rosenvinge) Cardinal |
| *Ectocarpus commensalis* Setchell & Gardner [=*Ectocarpus parvus* (Saunders) Hollenberg] |
| *Ectocarpus siliculosus* (Dillwyn) Lyngbye (=*Ectocarpus confervoides* Le Jolis; =*Ectocarpus siliculosus* var. *penicillatus* C. Agardh) |
| **Ectocarpus siliculosus* var. *adriaticus* (Ercegovic) Cormaci & Furnari |
| *Ectocarpus siliculosus* var. *arctus* (Kützing) Gallardo |
| *Ectocarpus siliculosus* var. *crouaniorum* (Thuret) Gallardo |
| *Ectocarpus siliculosus* var. *dasycarpus* (Kuckuck) Gallardo [=var. *crassus* (Kjellman) Gallardo] |
| *#Ectocarpus siliculosus* var. *hiemalis* (P.L. Crouan & H.M. Crouan ex Kjellman) Gallardo |
| *Ectocarpus siliculosus* var. *pygmaeus* (Areschoug) G. Russell |
| *Ectocarpus siliculosus* var. *subulatus* (Kützing) Gallardo |
| **Ectocarpus siliculosus* var. *venetus* (Kützing) Gallardo |
| *Ectocarpus virescens* Thuret ex Sauvageau |
| *Kuckuckia spinosa* (Kützing) Kornmann (=*Kuckuckia kylinii* Cardinal) |
| *Pilinia rimosa* Kützing |
|  |
| Family Pylaiellaceae |
| *Bachelotia antillarum* (Grunow) Gerloff |
| *#Pylaiella littoralis* (Linnaeus) Kjellman |
|  |
| *Order Fucales* |
|  |
| Family Cystoseiraceae |
| *Cystoseira abies-marina* (Gmelin) C. Agardh |
| **Cystoseira algeriensis* J. Feldmann |
| **Cystoseira amentacea* (C. Agardh) Bory |
| **Cystoseira amentacea* var. *spicata* (Ercegovic) Giaccone (=*Cystoseira spicata* Ercegovic) |
| **Cystoseira amentacea* var. *stricta* Montagne [=*Cystoseira stricta* (Montagne) Sauvageau] |
| *Cystoseira baccata* (Gmelin) Silva |
| **Cystoseira balearica* Sauvageau [=*Cystoseira brachycarpa* J. Agardh var. *balearica* (Sauvageau) Giaccone]^3^ |
| **Cystoseira balearica* var. *claudiae* Giaccone |
| *Cystoseira barbata* (Stackhouse) C. Agardh |
| **Cystoseira barbata* f. *repens* Zinova & Kalugina |
| **Cystoseira barbata* f. *insularum* Ercegovic |
| **Cystoseira barbata* var. *tophuloidea* (Ercegovic) Giaccone |
| **Cystoseira barbatula* Kützing (=*Cystoseira graeca* Schiffner ex Gerloff & Nizamuddin) |
| **Cystoseira brachycarpa* J. Agardh |
| **Cystoseira caespitosa* Sauvageau^4^ |
| *Cystoseira compressa* (Esper) Gerloff & Nizamuddin [=*Cystoseira compressa* f. *rosetta* (Ercegovic) Cormaci, Furnari, Scammacca & Serio] |
| **Cystoseira compressa* f. *plana* (Ercegovic) Cormaci, Furnari, Scammacca & Serio |
| **Cystoseira compressa* var. *pustulata* Ercegovic^5^ |
| **Cystoseira corniculata* (Turner) Zanardini^6^ |
| **Cystoseira crinita* Duby |
| **Cystoseira crinitophylla* Ercegovic |
| **Cystoseira dubia* Valiante |
| **Cystoseira elegans* Sauvageau |
| *Cystoseira foeniculacea* (Linnaeus) Greville [=*Cystoseira ercegovicii* Giaccone; =*Cystoseira discors* (Linnaeus) C. Agardh] |
| **Cystoseira foeniculacea* f. *latiramosa* (Ercegovic) Gómez-Garreta, Barceló, Ribera & Rull |
| **Cystoseira foeniculacea* f. *tenuiramosa* (Ercegovic) Gómez-Garreta, Barceló, Ribera & Rull |
| *Cystoseira foeniculacea f. schiffneri (Hamel) Gómez-Garreta, Barceló, Ribera & Rull |
| **Cystoseira funkii* Schiffner ex Gerloff & Nizamuddin^7^ |
| *Cystoseira humilis* Schousboe ex Kützing |
| *Cystoseira humilis* var. *myriophylloides* (Sauvageau) Price & John |
| **Cystoseira hyblaea* Giaccone |
| **Cystoseira jabukae* Ercegovic |
| **Cystoseira jabukae* f. *tenuissima* (Ercegovic) Cormaci, Furnari, Giaccone, Scammacca & Serio |
| *Cystoseira mauritanica* Sauvageau [=*Cystoseira gibraltarica* (Sauvageau) Dangeard?]^8^ |
| **Cystoseira mediterranea* Sauvageau |
| *Cystoseira nodicaulis* (Withering) Roberts |
| **Cystoseira pelagosae* Ercegovic |
| **Cystoseira rayssiae* Ramon |
| **Cystoseira sauvageauana* Hamel^9^ |
| **Cystoseira sedoides* (Desfontaines) C. Agardh |
| **Cystoseira spinosa* Sauvageau (=*Cystoseira adriatica* Sauvageau) |
| **Cystoseira spinosa* var. *compressa* (Ercegovic) Cormaci, Furnari, Giaccone, Scammacca & Serio (=*Cystoseira platyramosa* Ercegovic) |
| **Cystoseira spinosa* var. *tenuior* (Ercegovic) Cormaci, Furnari, Giaccone, Scammacca & Serio |
| **Cystoseira squarrosa* De Notaris |
| **Cystoseira susanensis* Nizamuddin |
| *Cystoseira tamariscifolia* (Hudson) Papenfuss |
| *Cystoseira usneoides* (Linnaeus) Roberts |
| **Cystoseira zosteroides* (Turner) C. Agardh (=*Cystoseira opuntioides* Bory ex Montagne)^10^ |
|  |
| Family Fucaceae |
| *Fucus spiralis* Linnaeus |
| *Fucus vesiculosus* Linnaeus |
| **Fucus virsoides* J. Agardh |
|  |
| Family Sargassaceae |
| *Sargassum acinarium* (Linnaeus) Setchell |
| *Sargassum desfontainesii* (Turner) C. Agardh |
| *Sargassum flavifolium* Kützing |
| *Sargassum furcatum* Kützing |
| **Sargassum hornschuchii* C. Agardh |
| *#Sargassum muticum* (Fensholt) Yendo |
| **Sargassum trichocarpum* J. Agardh |
| *Sargassum vulgare* C. Agardh |
|  |
| *Order Laminariales* |
|  |
| Family Alariaceae |
| *#Undaria pinnatifida* (Harvey) Suringar |
|  |
| Family Chordaceae |
| *#Chorda filum* (Linnaeus) Stackhouse |
|  |
| Family Laminariaceae |
| *Laminaria ochroleuca* Bachelot de la Pylaie |
| **Laminaria rodriguezii* Bornet |
| *#Saccharina japonica* (Areschoug) Lane, Mayes, Druehl & Saunders (=*Laminaria japonica* Areschoug) |
|  |
| *Order Nemodermatales* |
|  |
| Family Nemodermataceae |
| *Nemoderma tingitanum* Schousboe ex Bornet |
|  |
| *Order Onslowiales* |
|  |
| Family Onslowiaceae |
| **Verosphacela silvae Alongi, Cormaci & Furnari* |
|  |
| *Order Ralfsiales* |
|  |
| Family Ralfsiaceae |
| *Hapalospongidion macrocarpum* (J. Feldmann) León Álvarez & González (=*Mesospora mediterranea* J. Feldmann) |
| *Pseudolithoderma adriaticum* (Hauck) Verlaque (=*Lithoderma adriaticum* Hauck) |
| *Pseudolithoderma extensum* (P.L. Crouan & H.M. Crouan) Lund |
| *Ralfsia verrucosa* (Areschoug) Areschoug |
|  |
| Family Neoralfsiaceae |
| *Neoralfsia expansa* (J. Agardh) Lim & Kawai [=*Ralfsia expansa* (J. Agardh) J. Agardh] |
|  |
| *Order Scytosiphonales* |
|  |
| Family Scytosiphonaceae |
| *#Colpomenia peregrina* Sauvageau |
| *Colpomenia sinuosa* (Mertens ex Roth) Derbès & Solier |
| *Compsonema gracile* Kuckuck |
| *Compsonema minutum* (C. Agardh) Kornmann |
| *Hydroclathrus clathratus* (C. Agardh) Howe |
| *Petalonia fascia* (O.F. Müller) Kuntze |
| *Petalonia zosterifolia* (Reinke) Kuntze |
| *Rosenvingea intricata* (J. Agardh) Boergesen |
| *#Scytosiphon dotyi* Wynne |
| *Scytosiphon lomentaria* (Lyngbye) Link |
| *Stragularia clavata* (Harvey) Hamel (=*Ralfsia disciformis* P.L. Crouan & H.M. Crouan) |
|  |
| *Order Sphacelariales* |
|  |
| Family Choristocarpaceae |
| **Choristocarpus tenellus* Zanardini |
| *Discosporangium mesarthrocarpum* (Meneghini) Hauck |
|  |
| Family Sphacelariaceae |
| *Cladostephus spongiosus* (Hudson) C. Agardh |
| *Cladostephus spongiosus* f. *verticillatus* (Lightfoot) Prud'homme van Reine [=*Cladostephus hirsutus* (Linnaeus) Boudouresque & Perret-Boudouresque] |
| *Sphacelaria brachygonia* Montagne |
| *Sphacelaria cirrosa* (Roth) C. Agardh (=*Sphacelaria hystrix* Suhr ex Reinke) |
| *Sphacelaria fusca* (Hudson) Gray |
| *Sphacelaria nana* Nägeli ex Kützing |
| *Sphacelaria plumula* Zanardini |
| *Sphacelaria rigidula* Kützing |
| *Sphacelaria tribuloides* Meneghini |
| *Sphacella subtilissima* Reinke |
|  |
| Family Stypocaulaceae |
| *Halopteris filicina* (Grateloup) Kützing |
| *Stypocaulon scoparium* (Linnaeus) Kützing |
|  |
| *Order Sporochnales* |
|  |
| Family Sporochnaceae |
| *Carpomitra costata* (Stackhouse) Batters |
| **Carpomitra costata* var. *mediterranea* J. Feldmann |
| *Nereia filiformis* (J. Agardh) Zanardini |
| *Sporochnus gaertnera* (Gmelin) C. Agardh |
| *Sporochnus pedunculatus* (Hudson) C. Agardh |
| *Stilopsis lejolisii* (Thuret) Kuckuck & Nienburg ex Hamel |
|  |
| *Order Tilopteridales* |
|  |
| Family Phyllariaceae |
| *Phyllariopsis brevipes* (C. Agardh) Henry & South [=*Phyllaria reniformis* (Lamouroux) Rostafinsky] |
| *Phyllariopsis purpurascens* (C. Agardh) Henry & South |
| *Saccorhiza polyschides* (Lightfoot) Batters |

**Notes**

1. Rull et al. (2007) reported *Dictyota ciliolata* Sonder ex Kützing from the Northwestern Mediterranean but recent molecular examination of the collected specimens suggests that it is another, still unidentified, species.

2. We follow Algaebase in not considering *Ascocyclus orbicularis* (J. Agardh) Kjellman as synonym of *Myrionema magnusii* (Sauvageau) Loiseaux.

3. We prefer to maintain the specific category of *Cystoseira balearica* Sauvageau awaiting molecular evidences for its inclusion within *Cystoseira brachycarpa* J. Agardh.

4. We prefer to maintain the specific category of *Cystoseira caespitosa* Sauvageau awaiting molecular evidences indicating its identity with *Cystoseira balearica* Sauvageau.

5. Awaiting molecular evidences, we consider *Cystoseira compressa* (Esper) Gerloff & Nizamuddin var. *pustulata* Ercegovic (=*Cystoseira epiphytica* Schiffner ex Gerloff & Nizamuddin) to be a different taxa from *Cystoseira humilis* Kützing var. *humilis*.

6. This species is considered here to be a Mediterranean endemics. In our opinion, records from the Indian Ocean (Silva et al. 1996) need reexamination.

7. See Verlaque et al. (1999) for the differences between this species and *Cystoseira jabukae* Ercegovic.

8. Synonymy between *Cystoseira mauritanica* Sauvageau and *Cystoseira gibraltarica* (Sauvageau) Dangeard needs reexamination.

9. Includes *Cystoseira sauvageauana* var. *polyoedematis* (Sauvageau) Hamel. *C. sauvageauana* is considered here as endemic. In our opinion, records from the Atlantic Ocean (Prud'homme van Reine et al. 2005) need reexamination.

10. This species is considered here to be a Mediterranean endemic. In our opinion, records from the Indian Ocean (Silva et al. 1996) need reexamination.

*Taxa inquirenda* (see also Ribera et al. 1992)

*Cystoseira mediterranea* var. *valiantei* Sauvageau: see Verlaque et al. (1999).

*Ectocarpus elegans* Meneghini ex Ardissone: see Furnari et al. (2003).

*Leathesia cervicornis* Berthold: see Furnari et al. (2003).

*Sphacelaria olivacea* (Smith) C. Agardh: see Furnari et al. (2003).

*Taxa excludenda* (see also Ribera et al. 1992)

*Cystoseira myrica* (Gmelin) C. Agardh: see Zenetos et al. (2005).

*Dictyota ciliolata* Sonder ex Kützing: Molecular examination of specimens identified as *Dictyota ciliolata* in the Northwestern Mediterranean (Rull et al. 2007) suggest that they do not belong to this species (J. Rull, pers. comm.).

*Sargassum latifolium* (Turner) C. Agardh: unsupported records (Zenetos et al. 2008).

*Sorocarpus* sp.: Specimens tentatively identified as *Sorocarpus* sp. correspond to *Botrytella parva* (Takamatsu) Kim (Zenetos et al. 2008).

*Spatoglossum asperum* J. Agardh: see Zenetos et al. (2005)

**References**

Alongi G, Cormaci M, Furnari G. 2007. *Verosphacela silvae* sp. nov. (Onslowiaceae, Phaeophyceae) from the Mediterranean Sea. *Phycological Research* 55: 42-46.

Benhissoune S, Boudouresque CF, Verlaque M. 2002. A checklist of the seaweeds of the Mediterranean and Atlantic coasts of Morocco. II. Phaeophyceae. *Botanica Marina* 45: 217-230.

Boudouresque CF, Verlaque M. 2002. Biological pollution in the Mediterranean Sea: invasive versus introduced macrophytes. *Marine Pollution Bulletin* 44: 32-38.

Cormaci M, Furnari G, Alongi G, Serio D, Petrocelli A, Cecere E. 2001. Censimento delle macroalghe marine bentoniche delle coste pugliesi. *Thalassia Salentina* 25: 75-158.

Cormaci M, Furnari G, Pizzuto F. 1994*. Taonia lacheana* sp. nov. (Dictyotales, Phaeophyta), from the Mediterranean Sea. *Nordic Journal of Botany* 14: 463-466.

Furnari G, Giaccone G, Cormaci M, Alongi G, Serio D. 2003. Biodiversità marina delle coste italiane: catalogo del macrofitobentos. *Biologia Marina Mediterranea* 10(1): 1-482.

Prud'homme van Reine WF, Haroun RJ, Kostermans LBT. 2005. Checklists on seaweeds in the Atlantic Ocean and in the Cape Verde Archipelago. In: *IV Simposio Fauna e Flora das Ilhas Atlanticas*, Praia 9-13 setembro 2002: 13-26. Ministério do Ambiente, Agricultura e Pescas, República de Cabo Verde.

Ramon E. 2000. *Cystoseira rayssiae*: a new *Cystoseira* (Cystoseiraceae, Fucophyceae) from the shores of Israel, Eastern Mediterranean Sea. *Israel Journal of Plant Sciences* 48: 59-65.

Ribera MA, Gómez-Garreta AM, Gallardo T, Cormaci M, Furnari G, Giaccone G. 1992. Check-list of Mediterranean seaweeds I. Fucophyceae (Warming, 1884). *Botanica Marina* 35: 109-130.

Ribera MA, Gómez-Garreta A, Pérez-Ruzafa I, Barceló MC, Rull J. 2005. A new species of *Dictyopteris* (Dictyotales, Phaeophyceae) from the Iberian Peninsula: *Dictyopteris lucida* sp. nov. *Phycologia* 44: 651-657.

Rindi F, Sartoni G, Cinelli F. 2002. A floristic account of the benthic marine algae of Tuscany (Western Mediterranean Sea). *Nova Hedwigia* 74: 201-250.

Rull J, Ballesteros E, Barceló MC, Gómez-Garreta A, Ribera MA. 2007. *Dictyota ciliolata* Sonder ex Kützing (Phaeophyceae, Dictyotales) in the Mediterraean Sea. *Cryptogamie Algologie* 28: 89-97.

Silva PC, Basson PW, Moe RL. 1996. Catalogue of the benthic marine algae of the Indian Ocean. *University of California Publications in Botany* 79: 1-1259.

Taskin E, Wynne MJ, Özturk M. 2010. *Cylindrocarpus kuckuckii* sp. nov. (Chordariaceae, Phaeophyceae), a newly recognized species from the Aegean Sea (Turkey). *Nova Hedwigia* 90: 263-270.

Verlaque M, Ballesteros E, Sala E, Garrabou J. 1999. *Cystoseira jabukae* (Cystoseiraceae, Fucophyceae) from Corsica (Mediterranean) with notes on the previously misunderstood species *C. funkii*. *Phycologia* 38: 77-86.

Verlaque M, Ruitton S, Mineur F, Boudouresque CF. in press. 4. Macrophytes. In: Briand F (ed), *CIESM Atlas of Exotic species in the Mediterranean*. CIESM Publishers. Monaco.

Zenetos A, Çinar ME, Pancucci-Papadopoulou MA, Harmelin JG, Furnari G, Andaloro F, Bellou N, Streftaris N, Zibrowius H. 2005. Annotated list of marine alien species in the Mediterranean with records of the worst invasive species. *Mediterranean Marine Science* 6: 63-118.

Zenetos A, Meriç E, Verlaque M, Galil P, Boudouresque CF, Giangrande A, Çinar ME, Bilecenoglu M. 2008. Additions to the annotated list of marine alien biota in the Mediterranean with special emphasis on Foraminifera and Parasites. *Mediterranean Marine Science* 9: 119-165.

## **Table S10. Checklist of the phylum Rhodophyta and comments to the checklist**

Algae belonging to the phylum Rhodophyta (Kingdom Plantae) have been classified by Classes, Orders, Families, Species and other infraspecific levels (subspecies, varieties and forms). Ordination is alphabetical. When no infraspecific level is indicated the taxa corresponds to the type variety.

This list has been made using local checklists from Morocco (Benhissoune et al. 2002, 2003), Algeria (Perret-Boudouresque & Seridi 1989), Tunisia (Ben Maiz et al. 1987), Libya (Nizamuddin et al. 1979), Syria (Mayhoub 1976), Aegean Sea (Athanasiadis 1987), Italy (Furnari et al. 2003), Adriatic Sea (Giaccone 1978; Furnari et al. 1999), Corsica (Boudouresque & Perret-Boudouresque 1987), Albères coast (France) (Boudouresque et al. 1984; Knoepffler et al. 1990), Catalonia (Ballesteros 1990), Andalusia (Conde et al. 1996) and Spain (Gallardo et al. 1985), as well as a large number of smaller contributions. When Mediterranean revisions were already available for some taxonomic groups they have been used with some updates and modifications: Ceramiales (Gómez-Garreta et al. 2001), Corallinales (Bressan & Babbini-Benussi 1996, 2003), Acrochaetiaceae (Conde 1991), *Laurencia*-complex (Furnari et al. 2001; Furnari et al. 2002; Serio et al. 2004, 2008, 2010), *Gracilaria* (Gargiulo et al. 1992), and *Nemastoma* (Rodriguez et al. 2004). Lists of Mediterranean introduced species have also been considered (e.g. Boudouresque & Verlaque 2002; Zenetos et al. 2005, 2008; Verlaque 2001; Verlaque et al. in press) as well as some specific papers (e.g. Alongi et al. 2007, 2008; Athanasiadis 1999, 2002; Cormaci et al. 1993; Gargiulo et al. 1986, 1990; Perrone & Delle Foglie 2006; Rodriguez-Prieto & De Clerck 2009). Black Sea is not included.

Taxonomy follows Algaebase (www.algaebase.org) if not otherwise indicated. Endemic species (found only in the Mediterranean basin, Black Sea included) are preceded by the symbol *. A careful and critical examination of the existing records of every species from the available literature has been performed in order to consider a species as endemic. Introduced species are preceded by the symbol #. As it is not always easy to detect introduced species from the distributional records, we mainly follow the criteria by other authors specialized in this issue. Superscript numbers refer to notes. Some of the commonest synonyms found in Mediterranean literature (if any) are sometimes placed after the currently accepted name. Other synonyms can be found at Algaebase. A list of *taxa inquirenda* as well as a list of *taxa excludenda* is also given at the end of the list; species of the Order Ceramiales considered as *taxa inquirenda* or *taxa excludenda* in Gómez-Garreta et al. (2001) are not stated again.

| **Kingdom Plantae** |
| --- |
|  |
| **Phylum Rhodophyta** |
|  |
| **Class Bangiophyceae** |
|  |
| *Order Bangiales* |
|  |
| Family Bangiaceae^1^ |
| *Bangia fuscopurpurea* (Dillwyn) Lyngbye |
| *Porphyra atropurpurea* (Olivi) De Toni |
| *Porphyra dioica* Brodie & Irvine |
| *Porphyra leucosticta* Thuret |
| *Porphyra linearis* Greville |
| *Porphyra purpurea* (Roth) C. Agardh |
| *Porphyra umbilicalis* Kützing |
| *#Porphyra yezoensis* Ueda |
|  |
| **Class Compsopogonophyceae** |
|  |
| *Order Compsopogonales* |
|  |
| Family Compsopogonaceae |
| **Comsopogon aegyptiacus* Aleem |
| *Compsopogon coeruleus* (Balbis ex C. Agardh) Montagne |
|  |
| *Order Erythropeltidales* |
|  |
| Family Erythrotrichiaceae^2^ |
| *Erythrocladia irregularis* Rosenvinge |
| *Erythrocladia polystromatica* Dangeard |
| *Erythrocladia violacea* Dangeard |
| *Erythropeltis discigera* (Berthold) Schmitz |
| *Erythrotrichia bertholdii* Batters |
| *Erythrotrichia carnea* (Dillwyn) J. Agardh [=*Erythrotrichia biseriata* Tanaka; =*Erythrotrichia ceramicola* (Lyngbye) Areschoug] |
| *Erythrotrichia investiens* (Zanardini) Bornet |
| *Erythrotrichia reflexa* (P.L. Crouan & H.M. Crouan) Thuret ex De Toni |
| **Erythrotrichia rosea* Dangeard |
| *Erythrotrichia simplex* Dangeard |
| *Porphyrostromium boryanum* (Montagne) Silva [=*Erythrotrichia boryana* (Montagne) Berthold] |
| *Porphyrostromium ciliare* (Carmichael) Wynne [= *Erythrotrichia ciliaris* (Carmichael) Thuret; =*Erythrotrichia obscura* Berthold] |
| *Porphyrostromium obscurum* (Berthold) Kornmann (=*Erythrocladia grisea Dangeard*) |
| *Sahlingia subintegra* (Rosenvinge) Kornmann |
|  |
| *Order Rhodochaetales* |
|  |
| Family Rhodochaetaceae |
| *Rhodochaete pulchella* Thuret ex Bornet (=*Rhodochaete parvula* Thuret) |
|  |
| **Class Florideophyceae** |
|  |
| *Order Acrochaetiales* |
|  |
| Family Acrochaetiaceae^2^ |
| *Acrochaetium alariae* (Jónsson) Bornet |
| **Acrochaetium boergesenii* Schiffner |
| **Acrochaetium caesareae* J. Feldmann^3^ |
| *#Acrochaetium codicola* Borgesen [=*Rhodothamniella codicola* (Borgesen) Bidoux & Magne] |
| *Acrochaetium codii* (P.L. Crouan & H.M. Crouan) Hamel [=*Rhodothamniella codii* (P.L. Crouan & H.M. Crouan) J. Feldmann] |
| *Acrochaetium corymbiferum* (Thuret) Batters |
| **Acrochaetium duboscqii* J. Feldmann |
| *Acrochaetium endozoicum* (Darbishire) Batters |
| *Acrochaetium gynandrum* (Rosenvinge) Hamel |
| **Acrochaetium hamelii* J. Feldmann^3^ |
| **Acrochaetium hauckii* Schiffner [=*Rhodochorton hauckii* (Schiffner) Hamel] |
| *Acrochaetium humile* (Rosenvinge) Borgesen |
| **Acrochaetium incrassatum* Ercegovic |
| *Acrochaetium infestans* Howe & Hoyt |
| *Acrochaetium leptonema* (Rosenvinge) Borgesen |
| *Acrochaetium maluinum* Hamel |
| **Acrochaetium mediterraneum* (Levring) Athanasiadis |
| *Acrochaetium microscopicum* (Nägeli ex Kützing) Nägeli [=*Acrochaetium crassipes* (Boergesen) Boergesen] |
| **Acrochaetium minutum* (Suhr) Hamel [=*Acrochaetium minutissimum* (Kützing) Nägeli] |
| **Acrochaetium molinieri* Coppejans & Boudouresque |
| *Acrochaetium moniliforme* (Rosenvinge) Borgesen (=*Acrochaetium mahumetanum* Hamel) |
| *Acrochaetium nemalii* (De Notaris ex Dufour) Bornet |
| *Acrochaetium parvulum* (Kylin) Hoyt |
| *Acrochaetium reductum* (Rosenvinge) Hamel |
| *#Acrochaetium robustum* Borgesen (=*Acrochaetium sargassicola* Boergesen) |
| *Acrochaetium rosulatum* (Rosenvinge) Papenfuss |
| *Acrochaetium savianum* (Meneghini) Nägeli [=*Acrochaetium thuretii* (Bornet) Collins & Hervey] |
| *Acrochaetium secundatum* (Lyngbye) Nägeli [=*Acrochaetium virgatulum* (Harvey) Batters; =*Acrochaetium rhipidandrum* (Rosenvinge) Hamel] |
| *#Acrochaetium spathoglossi* Borgesen |
| *Acrochaetium subpinnatum* Bornet ex Hamel |
| *#Acrochaetium subseriatum* Borgesen |
| *Acrochaetium subtilissimum* (Kützing) Hamel |
| *Acrochaetium trifilum* (Buffham) Batters |
| *Colaconema bonnemaisoniae* Batters [=*Acrochaetium bonnemaisoniae* (Batters) J. Feldmann & G. Feldmann] |
| *Colaconema caespitosum* (J. Agardh) Jackelman, Stegenga & Bolton [=*Acrochaetium caespitosum* (J. Agardh) Nageli] |
| *Colaconema chylocladiae* Batters [=*Acrochaetium chylocladiae* (Batters) Batters] |
| *Colaconema daviesii* (Dillwyn) Stegenga [=*Acrochaetium daviesii* (Dillwyn) Nägeli] |
| *Colaconema garbaryi* Gabrielson |
| *Colaconema gracile* (Boergesen) Ateweberhan & Prud'homme van Reine (=*Acrochaetium gracile* Boergesen) |
| *Colaconema hallandicum* (Kylin) Afonso-Carrillo, Sansón, Sangil & Diaz-Villa [=*Acrochaetium hallandicum* (Kylin) Hamel f*. harmoricum* Hamel] |
| *Colaconema membranaceum* (Magnus) Woelkerling [=*Audouinella membranacea* (Magnus) Papenfuss] |
| *Rhodochorton purpureum* (Lightfoot) Rosenvinge [=*Rhodochorton rothii* (Turton) Nägeli; =*Audouinella purpurea* (Lightfoot) Woelkerling] |
| *Rhodochorton velutinum* (Hauck) Hamel |
| *Rhodothamniella floridula* (Dillwyn) J. Feldmann |
| *Schmitziella endophloea* Bornet & Batters |
|  |
| *Order Acrosymphytales* |
|  |
| Family Acrosymphytaceae |
| *Acrosymphyton purpuriferum* (J. Agardh) Sjösted [=*Hymenoclonium serpens* (P.L. Crouan & H.M. Crouan) Batters] |
| *Schimmelmannia schousboei* (J. Agardh) J. Agardh |
|  |
| *Order Bonnemaisoniales* |
|  |
| Family Bonnemaisoniaceae |
| *#Asparagopsis armata* Harvey [=*Falkenbergia rufolanosa* (Harvey) Schmitz] |
| *#Asparagopsis taxiformis* (Delile) Trevisan [=*Falkenbergia hillebrandii* (Bornet) Falkenberg] |
| *Bonnemaisonia asparagoides* (Woodward) C. Agardh [=*Hymenoclonium serpens* (P.L. Crouan & H.M. Crouan) Batters] |
| *Bonnemaisonia clavata* Hamel |
| *#Bonnemaisonia hamifera* Hariot (=*Trailliella intricata* Batters) |
|  |
| Family Naccariaceae |
| *Naccaria wiggii* (Turner) Endlicher |
|  |
| *Order Ceramiales* |
|  |
| Family Ceramiaceae |
| *#Acrothamnion pressii* (Sonder) Wollaston |
| *Aglaothamnion bipinnatum* (P.L. Crouan & H.M. Crouan) J. Feldmann & G. Feldmann |
| **Aglaothamnion caudatum* (J. Agardh) Feldmann-Mazoyer |
| *Aglaothamnion cordatum* (Boergesen) Feldmann-Mazoyer |
| *#Aglaothamnion feldmanniae* Halos |
| *Aglaothamnion gallicum* (Nägeli) L'Hardy-Halos & Ardré |
| *Aglaothamnion scopulorum* (C. Agardh) Feldmann-Mazoyer |
| *Aglaothamnion tenuissimum* (Bonnemaison) Feldmann-Mazoyer |
| **Aglaothamnion tenuissimum* var. *mazoyerae* Furnari, L'Hardy-Halos, Rueness & Serio |
| *Aglaothamnion tripinnatum* (C. Agardh) Feldman-Mazoyer |
| *Anotrichium barbatum* (C. Agardh) Nägeli |
| *Anotrichium furcellatum* (J. Agardh) Baldock |
| *#Anotrichium okamurae* Baldock |
| *Anotrichium tenue* (C. Agardh) Nägeli |
| *#Antithamnion amphigeneum* A. Millar |
| **Antithamnion compactum* (Grunow) Schiffner |
| *Antithamnion cruciatum* (C. Agardh) Nägeli |
| *Antithamnion decipiens* (J. Agardh) Athanasiadis (=*Antithamnion ogdeniae* Abbott) |
| **Antithamnion heterocladum* Funk |
| *#Antithamnion hubbsii* Dawson^4^ |
| **Antithamnion piliferum* Cormaci & Furnari |
| **Antithamnion tenuissimum* (Hauck) Schiffner |
| *#Antithamnionella boergesenii* (Cormaci & Furnari) Athanasiadis |
| *#Antithamnionella elegans* (Berthold) Price & John |
| *#Antithamnionella spirographidis* (Schiffner) Wollaston |
| *#Antithamnionella sublittoralis* (Setchell & Gardner) Athanasiadis (=*Antithamnionella elegans* var. *decussata* Cormaci & Furnari) |
| *#Antithamnionella ternifolia* (Hooker & Harvey) Lyle |
| **Balliella cladoderma* (Zanardini) Athanasiadis |
| *Bornetia secundiflora* (J. Agardh) Thuret |
| *Callithamniella tingitana* (Schousboe ex Bornet) Feldmann- Mazoyer |
| *Callithamnion corymbosum* (Smith) Lyngbye |
| *Callithamnion granulatum* (Ducluzeau) C. Agardh |
| *Callithamnion tetragonum* (Withering) Gray |
| *Callithamnion tetricum* (Dillwyn) Gray^5^ |
| **Ceramium bertholdii* Funk |
| *#Ceramium bisporum* Ballantine |
| *Ceramium ciliatum* (Ellis) Ducluzeau |
| *Ceramium ciliatum* var. *robustum* (J. Agardh) Feldmann-Mazoyer |
| *Ceramium cimbricum* Petersen |
| *Ceramium cimbricum* f. *flaccidum* (Petersen) Furnari & Serio |
| *Ceramium circinatum* (Kützing) J. Agardh |
| *Ceramium codii* (H. Richards) Feldmann-Mazoyer |
| *Ceramium comptum* Boergesen |
| *Ceramium deslongchampsii* Chauvin ex Duby |
| *Ceramium diaphanum* (Lightfoot) Roth |
| *Ceramium echionotum* J. Agardh |
| **Ceramium echionotum* var. *mediterraneum* Feldmann-Mazoyer |
| *Ceramium gaditanum* (Clemente) Cremades (=*Ceramium flabelligerum* J. Agardh) |
| **Ceramium gaditanum* var. *mediterraneum* (Debray) Cremades |
| **Ceramium giacconei* Cormaci & Furnari |
| **Ceramium graecum* Lazaridou & Boudouresque |
| **Ceramium incospicuum* Zanardini |
| **Ceramium petitii* Feldmann-Mazoyer |
| *Ceramium secundatum* Lyngbye |
| *Ceramium siliquosum* (Kützing) Maggs & Hommersand |
| *Ceramium siliquosum* var. *elegans* (Roth) Furnari |
| *Ceramium siliquosum* var. *lophophorum* (Feldmann-Mazoyer) Serio |
| *Ceramium siliquosum* var. *zostericola* (Feldmann-Mazoyer) Furnari |
| **Ceramium siliquosum* var. *zostericola* f. *acrocarpum* (Feldmann-Mazoyer) Furnari |
| **Ceramium siliquosum* var. *zostericola* f. *minusculum* (Feldmann-Mazoyer) Gómez-Garreta, Gallardo, Ribera, Cormaci, Furnari, Giaccone & Boudouresque |
| *#Ceramium strobiliforme* Lawson & John |
| *Ceramium tenerrimum* (Martens) Okamura |
| **Ceramium tenerrimum* var. *brevizonatum* (Petersen) Feldmann-Mazoyer |
| *Ceramium virgatum* Roth (=*Ceramium rubrum* auct.) |
| **Ceramium virgatum* var. *implexo-contortum* (Solier) Furnari |
| **Ceramium virgatum* var. *tenue* (C. Agardh) Furnari |
| *Compsothamnion gracillimum* De Toni |
| *Compsothamnion thuyoides* (Smith) Nägeli |
| *Corallophila cinnabarina* (Grateloup ex Bory) R.E. Norris |
| *Crouania attenuata* (C. Agardh) J. Agardh [inc. var. *bispora* (P.L. Crouan & H.M. Crouan) Hauck] |
| *Crouania francescoi* Cormaci, Furnari & Scammaca |
| **Crouania ischiana* (Funk) Boudouresque & M. Perret (=*Pseudocrouania ischiana* Funk) |
| **Dohrniella nana* Mayhoub |
| **Dohrniella neapolitana* Funk |
| *Gayliella flaccida* (Harvey ex Kützing) T.O. Cho & L.J. McIvor [=*Ceramium flaccidum* (Kützing) Ardissone] |
| *#Griffithsia corallinoides* (Linnaeus) Trevisan |
| **Griffithsia genovefae* J. Feldmann |
| *Griffithsia opuntioides* J. Agardh |
| *Griffithsia phyllamphora* J. Agardh |
| *Griffithsia schousboei* Montagne |
| **Griffithsia schousboei* var. *minor* J. Feldmann ex Feldmann-Mazoyer |
| **Gulsonia nodulosa* (Ercegovic) J. Feldmann & Feldmann-Mazoyer |
| *Gymnothamnion elegans* (Schousboe ex C. Agardh) J. Agardh |
| **Halosia elisae* Cormaci & Furnari |
| *Halurus equisetifolius* (Lightfoot) Kützing |
| *Halurus flosculosus* (Ellis) Maggs & Hommersand |
| **Halurus flosculosus* var. *irregularis* (C. Agardh) Gómez-Garreta, Gallardo, Ribera, Cormaci, Furnari, Giaccone & Boudouresque |
| **Halurus flosculosus* var. *sphaericus* (Schousboe ex C. Agardh) Gómez-Garreta, Gallardo, Ribera, Cormaci, Furnari, Giaccone & Boudouresque |
| *Lejolisia mediterranea* Bornet |
| *Microcladia glandulosa* (Solander ex Turner) Greville |
| *Monosporus pedicellatus* (Smith) Solier |
| *Monosporus pedicellatus* var. *tenuis* (Feldmann-Mazoyer) Huisman & Kraft |
| *Pleonosporium borreri* (Smith) Nägeli |
| *Pterothamnion crispum* (Ducluzeau) Nägeli |
| *Pterothamnion plumula* (Ellis) Nägeli |
| **Pterothamnion plumula* ssp. *haplokladion* Athanasiadis |
| *Pterothamnion plumula* ssp. *verticillatum* Athanasiadis |
| *Pterothamnion polyacanthum* (Kützing) Nägeli |
| **Ptilocladiopsis horrida* Berthold |
| *Ptilothamnion pluma* (Dillwyn) Thuret |
| **Seirospora apiculata* (Meneghini) Feldmann-Mazoyer |
| **Seirospora giraudyi* (Kützing) De Toni |
| *Seirospora interrupta* (Smith) Schmitz |
| **Seirospora sphaerospora* J. Feldmann |
| *Spermothamnion flabellatum* Bornet |
| **Spermothamnion flabellatum* f. *disporum* Feldmann-Mazoyer |
| *Spermothamnion irregulare* (J. Agardh) Ardissone |
| **Spermothamnion johannis* Feldmann-Mazoyer |
| *Spermothamnion repens* (Dillwyn) Rosenvinge |
| *Spermothamnion repens* var. *flagelliferum* (De Notaris) Feldmann-Mazoyer |
| **Spermothamnion repens* var. *variabile* (C. Agardh) Feldmann-Mazoyer |
| *Spermothamnion strictum* (C. Agardh) Ardissone |
| *Sphondylothamnion multifidum* (Hudson) Nägeli |
| *Sphondylothamnion multifidum* f. *distichum* Feldmann-Mazoyer |
| *#Spongoclonium caribaeum* (Boergesen) Wynne [=*Pleonosporium caribaeum* (Boergesen) R.E. Norris] |
| *Spyridia filamentosa* (Wulfen) Harvey |
| *Spyridia hypnoides* (Bory) Papenfuss |
| *Tiffaniella capitata* (Schousboe ex Bornet) Doty & Meñez |
| **Tiffaniella feldmanniae* (P. Huvé) Gillis & Coppejans |
| *Vickersia baccata* (J. Agardh) Karsakoff |
| **Woelkerlingia minuta* Alongi, Cormari & Furnari |
| *Wrangelia penicillata* (C. Agardh) C. Agardh |
|  |
| Family Dasyaceae |
| *Dasya baillouviana* (Gmelin) Montagne |
| *Dasya corymbifera* J. Agardh |
| *Dasya hutchinsiae* Harvey |
| *Dasya ocellata* (Grateloup) Harvey |
| **Dasya penicillata* Zanardini [=*Eupogodon penicillatus* (Zanardini) Silva] |
| *Dasya punicea* (Zanardini) Meneghini ex Zanardini |
| **Dasya rigescens* Zanardini |
| *Dasya rigidula* (Kützing) Ardissone |
| *#Dasya sessilis* Yamada |
| **Dasyella gracilis* Falkenberg |
| *#Dasysiphonia* sp.^6^ |
| *Eupogodon planus* (C. Agardh) Kützing [=*Eupogodon spinellus* (C. Agardh) Kützing; =*Eupogodon cervicornis* (J. Agardh) Kützing] |
| *Halydictyon mirabile* Zanardini |
| *Heterosiphonia crispella* (C. Agardh) Wynne |
| *Heterosiphonia crispella* var. *laxa* (Boergesen) Wynne |
| *Heterosiphonia plumosa* (Ellis) Batters |
|  |
| Family Delesseriaceae |
| *Acrosorium ciliolatum* (Harvey) Kylin [=*Acrosorium venulosum* (Zanardini) Kylin]^7^ |
| *#Apoglossum gregarium* (Dawson) Wynne |
| *Apoglossum ruscifolium* (Turner) J. Agardh |
| **Arachnophyllum confervaceum* (Meneghini) Zanardini^8^ |
| *Cottoniella filamentosa* (M. Howe) Boergesen [=*Cottoniella filamentosa* var. *fusiformis* (Boergesen) Cormaci & Furnari] |
| *Cottoniella filamentosa* var. *algeriensis* (Schotter) Cormaci & Furnari |
| **Cottoniella libyensis* Nizamuddin & Godeh |
| *Cryptopleura ramosa* (Hudson) Kylin ex L. Newton^7^ |
| **Erythroglossum balearicum* J. Agardh ex Kylin |
| *Erythroglossum laciniatum* (Lightfoot) Maggs & Hommersand [=*Polyneura gmelinii* (Lamouroux) Kylin] |
| *Erythroglossum sandrianum* (Kützing) Kylin |
| *Haraldia lenormandii* (Derbès & Solier) J. Feldmann |
| *Hypoglossum hypoglossoides* (Stackhouse) Collins & Hervey |
| **Myriogramme carnea* (J.J. Rodríguez) Kylin^9^ |
| **Myriogramme distromatica* J.J. Rodriguez ex Boudouresque^9^ |
| *Myriogramme minuta* Kylin^9^ |
| **Myriogramme tristromatica* (J.J. Rodríguez ex Mazza) Boudouresque^9^ |
| **Myriogramme unistromatica* Coppejans, nomen nudum^9^ |
| **Nitophyllum albidum* Ardissone |
| **Nitophyllum flabellatum* Ercegovic |
| **Nitophyllum micropunctatum* Funk |
| *Nitophyllum punctatum* (Stackhouse) Greville |
| *#Nithophyllum stellato-corticatum* Okamura |
| **Radicilingua adriatica* (Kylin) Papenfuss |
| *Radicilingua reptans* (Kylin) Papenfuss |
| *Radicilingua thysanorhizans* (Holmes) Papenfuss |
| *Taenioma nanum* (Kützing) Papenfuss |
| *Taenioma perpusillum* (J. Agardh) J. Agardh |
|  |
| Family Rhodomelaceae |
| *#Acanthophora nayadiformis* (Delile) Papenfuss |
| *Alsidium corallinum* C. Agardh |
| **Alsidium helminthochorton* (Schwendimann) Kützing |
| *Aphanocladia stichidiosa* (Funk) Ardré |
| **Boergeseniella deludens* (Falkenberg) Kylin |
| *Boergeseniella fruticulosa* (Wulfen) Kylin |
| *Boergeseniella thuyoides* (Harvey) Kylin |
| *Bostrychia scorpioides* (Hudson) Montagne ex Kützing |
| *Brongniartella byssoides* (Goodenough & Woodward) Schmitz |
| **Chondria boryana* (J. Agardh) De Toni |
| *Chondria capillaris* (Hudson) Wynne |
| *#Chondria coerulescens* (J. Agardh) Falkenberg |
| *#Chondria curvilineata* Collins & Hervey |
| *Chondria dasyphylla* (Woodward) C. Agardh |
| *Chondria mairei* Feldmann-Mazoyer |
| *#Chondria pygmaea* Garbary & Vandermeulen |
| *Chondria scintillans* Feldmann-Mazoyer |
| *Digenea simplex* (Wulfen) C. Agardh |
| *Dipterosiphonia dendritica* (C. Agardh) Schmitz |
| *Dipterosiphonia rigens* (Schousboe ex C. Agardh) Falkenberg |
| *Erythrocystis montagnei* (Derbès & Solier) Silva |
| *Halopithys incurva* (Hudson) Batters |
| *#Herposiphonia parca* Setchell |
| *Herposiphonia secunda* (C. Agardh) Ambronn |
| *Herposiphonia secunda* f. *tenella* (C. Agardh) Wynne |
| *Janczewskia verrucaeformis* Solms-Laubach |
| *#Laurencia caduciramulosa* Masuda & Kawaguchi |
| *Laurencia chondrioides* Boergesen |
| **Laurencia epiphylla* Boisset & Lino |
| *Laurencia glandulifera* (Kützing) Kützing |
| *Laurencia intricata* Lamouroux |
| *Laurencia majuscula* (Harvey) Lucas |
| *Laurencia microcladia* Kützing |
| **Laurencia minuta* Vandermeulen, Garbary & Guiry ssp. *scammaccae* Furnari & Cormaci |
| *Laurencia obtusa* (Hudson) Lamouroux |
| *#Laurencia okamurae* Yamada |
| *Laurencia pyramidalis* Bory ex Kützing |
| *Leptosiphonia schousboei* (Thuret) Kylin^5^ |
| *#Lophocladia lallemandii* (Montagne) Schmitz^8^ |
| *Lophocladia trichoclados* (C. Agardh) J. Agardh^8^ |
| *Lophosiphonia cristata* Falkenberg |
| *Lophosiphonia obscura* (C. Agardh) Falkenberg [=*Lophosiphonia subadunca* (Kützing) Falkenberg] |
| *Lophosiphonia reptabunda* (Suhr) Kylin |
| *Neosiphonia elongella* (Harvey) Kim & Lee (=*Polysiphonia elongella* Harvey) |
| *Neosiphonia ferulacea* (Suhr ex J. Agardh) Guimaraes & Fujii (=*Polysiphonia ferulacea* Suhr ex J. Agardh) |
| *#Neosiphonia harveyi* (Bailey) Kim, Choi, Guiry & Saunders (=*Polysiphonia harveyi* Bailey; =*Polysiphonia mottei* Lauret) |
| *Neosiphonia sphaerocarpa* (Boergesen) Kim & Lee (=*Polysiphonia sphaerocarpa* Boergesen) |
| *Ophidocladus simpliciusculus* (P.L. Crouan & H.M. Crouan) Falkenberg |
| *Osmundaria volubilis* (Linnaeus) J. Agardh |
| **Osmundea maggsiana* Serio, Cormaci & Furnari |
| *Osmundea oederi* (Gunnerus) Furnari [=*Osmundea ramosissima* (Oeder) Athanasiadis] |
| **Osmundea pelagiensis* Furnari |
| **Osmundea pelagosae* (Schiffner) K.W. Nam |
| *Osmundea pinnatifida* (Hudson) Stackhouse |
| *Osmundea truncata* (Kützing) K.W. Nam & Maggs |
| **Osmundea verlaquei* Furnari |
| *Palisada maris-rubri* (K.W. Nam & Saito) K.W. Nam |
| *Palisada papillosa* (C. Agardh) K.W. Nam [=*Chondrophycus papillosus* (C. Agardh) Garbary & Harper] |
| *Palisada patentiramea* (Montagne) Cassano, Sentíes, Gil-Rodriguez & M.T. Fujii [=*Chondrophycus* *patentirameus* (Montagne) K.W. Nam] |
| *Palisada tenerrima* (Cremades) Serio, Cormaci, Furnari & Boisset [=*Chondrophycus tenerrimus* (Cremades) Furnari, Boisset, Cormaci & Serio] |
| *Palisada thuyoides* (Kützing) Cassano, Sentíes, Gil-Rodriguez & M.T. Fujii [=*Laurencia paniculata* (C. Agardh) J. Agardh] |
| **Polysiphonia arachnoidea* (C. Agardh) Zanardini^10^ |
| *#Polysiphonia atlantica* Kapraun & J. Norris (=*Polysiphonia macrocarpa* Harvey) |
| **Polysiphonia atra* Zanardini |
| **Polysiphonia banyulensis* Coppejans |
| **Polysiphonia biformis* Zanardini (=*Dasya corallicola* Funk) |
| *Polysiphonia breviarticulata* (C. Agardh) Zanardini |
| *Polysiphonia brodiei* (Dillwyn) Sprengel |
| *Polysiphonia ceramiaeformis* P.L. & H.M. Crouan |
| **Polysiphonia cladorhiza* Ardissone |
| *Polysiphonia denudata* (Dillwyn) Greville ex Harvey |
| **Polysiphonia derbesii* Solier ex Kützing |
| **Polysiphonia deusta* (Roth) Sprengel |
| **Polysiphonia dichotoma* Kützing |
| *Polysiphonia elongata* (Hudson) Sprengel |
| *Polysiphonia fibrillosa* (Dillwyn) Sprengel |
| *Polysiphonia flexella* (C. Agardh) J. Agardh |
| *Polysiphonia flocculosa* (C. Agardh) Endlicher |
| **Polysiphonia foeniculacea* (C. Agardh) Sprengel |
| *#Polysiphonia fucoides* (Hudson) Greville |
| *Polysiphonia funebris* De Notaris ex J. Agardh |
| *Polysiphonia furcellata* (C. Agardh) Harvey |
| *#Polysiphonia morrowii* Harvey |
| *Polysiphonia opaca* (C. Agardh) Moris & De Notaris |
| **Polysiphonia ornata* J. Agardh |
| *Polysiphonia orthocarpa* Rosenvinge |
| *#Polysiphonia paniculata* Montagne |
| **Polysiphonia perforans* Cormaci, Furnari, Pizzuto & Serio |
| *Polysiphonia polyspora* (C. Agardh) Montagne |
| *Polysiphonia sanguinea* (C. Agardh) Zanardini |
| *Polysiphonia scopulorum* Harvey [=*Lophosiphonia scopulorum* (Harvey) Womersley] |
| *Polysiphonia sertularioides* (Grateloup) J. Agardh |
| **Polysiphonia setigera* Kützing |
| *Polysiphonia spinosa* (C. Agardh) J. Agardh |
| *Polysiphonia stricta* (Dillwyn) Greville [=*Polysiphonia urceolata* (Lightfoot ex Dillwyn) Greville] |
| *Polysiphonia stuposa* Zanardini ex Kützing (=*Polysiphonia foetidissima* Cocks ex Bornet)^8^ |
| *Polysiphonia subtilissima* Montagne |
| *Polysiphonia subulata* (Ducluzeau) P.L. Crouan & H.M. Crouan |
| *Polysiphonia subulifera* (C. Agardh) Harvey |
| *Polysiphonia tenerrima* Kützing |
| *Polysiphonia tripinnata* J. Agardh |
| *Pterosiphonia ardreana* Maggs & Hommersand^8^ |
| *Pterosiphonia complanata* (Clemente) Falkenberg |
| *Pterosiphonia parasitica* (Hudson) Falkenberg |
| *Pterosiphonia pennata* (C. Agardh) Sauvageau |
| *Pterosiphonia pinnulata* (Kützing) Maggs & Hommersand |
| *#Pterosiphonia tanakae* Uwai & Masuda |
| **Rodriguezella bornetii* (J.J. Rodriguez) Schmitz ex J.J. Rodriguez |
| **Rodriguezella ligulata* J. Feldmann, nomen nudum |
| **Rodriguezella pinnata* (Kützing) Schmitz ex Falkenberg |
| **Rodriguezella strafforelloi* Schmitz ex J.J. Rodriguez |
| *Rytiphlaea tinctoria* (Clemente) C. Agardh |
| *Stichothamnion cymatophilum* Boergesen |
| *Streblocladia collabens* (C. Agardh) Falkenberg |
| *#Symphyocladia marchantioides* (Harvey) Falkenberg |
| *#Womersleyella setacea* (Hollenberg) R.E. Norris (=*Polysiphonia setacea* Hollenberg) |
|  |
| *Order Corallinales* |
|  |
| Family Corallinaceae |
| *Amphiroa beauvoisii* Lamouroux |
| *Amphiroa cryptarthrodia* Zanardini [=*Amphiroa rubra* (Philippi) Woelkerling; =*Amphiroa verruculosa* auct. med.] |
| *Amphiroa fragilissima* (Linnaeus) Lamouroux |
| *Amphiroa rigida* Lamouroux |
| *Corallina caespitosa* Walker, Brodie & Irvine |
| *Corallina elongata* Ellis & Solander (=*Corallina mediterranea* Areschoug) |
| *Corallina officinalis* Linnaeus |
| **Haliptilon attenuatum* (Kützing) Garbary & Johansen |
| *Haliptilon virgatum* (Zanardini) Garbary & Johansen (=*Corallina granifera* Ellis & Solander) |
| *Hydrolithon boreale* (Foslie) Chamberlain (=*Melobesia farinosa* var. *borealis* Lemoine) |
| *Hydrolithon cruciatum* (Bressan) Chamberlain (=*Fosliella cruciata* Bressan) |
| *Hydrolithon farinosum* (Lamouroux) Penrose & Chamberlain [=*Melobesia farinosa* Lamouroux; =*Fosliella farinosa* (Lamouroux) Howe] |
| *Hydrolithon farinosum* var. *chalicodictyum* (Taylor) Serio |
| *Jania adhaerens* Lamouroux |
| *Jania longifurca* Zanardini |
| *Jania rubens* (Linnaeus) Lamouroux |
| *Jania rubens* var. *corniculata* (Linnaeus) Yendo [=*Jania nitidula* Meslin; =*Jania corniculata* (Linnaeus) Lamouroux] |
| *Jania squamata* (Linnaeus) Kim, Guiry & Choi [=*Haliptilon squamatum* (Linnaeus) Johansen, Irvine & Webster] |
| *Lithophyllum byssoides* (Lamarck) Foslie (=*Lithophyllum lichenoides* Philippi; =*Lithophyllum tortuosum* auct. non Esper) |
| **Lithophyllum cabiochae* (Boudouresque & Verlaque) Athanasiadis (=*Pseudolithophyllum cabiochae* Boudouresque & Verlaque) |
| *Lithophyllum corallinae* (P.L. Crouan & H.M. Crouan) Heydrich [=*Dermatolithon corallinae* (P.L. Crouan & H.M. Crouan) Foslie] |
| *Lithophyllum cystoseirae* (Hauck) Heydrich [=*Dermatolithon cystoseirae* (Hauck) H. Huvé] |
| *Lithophyllum decussatum* (Ellis & Solander) Philippi |
| *Lithophyllum dentatum* (Kützing) Foslie |
| *Lithophyllum fasciculatum* (Lamarck) Foslie |
| *Lithophyllum incrustans* Philippi |
| *Lithophyllum lobatum* Lemoine in Boergesen |
| *Lithophyllum orbiculatum* (Foslie) Foslie [=*Lithothamnion subtenellum* (Foslie) Lemoine] |
| *Lithophyllum papillosum* Zanardini ex Hauck [=*Goniolithon papillosum* (Zanardini ex Hauck) Boergesen] |
| *Lithophyllum pustulatum* (Lamouroux) Foslie [=*Lithophyllum hapalidioides* (P.L. Crouan & H.M. Crouan) Hariot; =*Titanoderma confine* (P.L. Crouan & H.M. Crouan) Price, John & Lawson; =*Dermatolithon litorale* (Suneson) Hamel & Lemoine] |
| *Lithophyllum racemus* (Lamarck) Foslie |
| *Lithophyllum stictaeforme* (Areschoug in J. Agardh) Hauck [=*Pseudolithophyllum expansum* auct., pro parte; *Lithophyllum frondosum* (Dufour) Furnari, Cormaci & Alongi] |
| *#Lithophyllum yessoense* Foslie |
| *Neogoniolithon brassica-florida* (Harvey) Setchell & Mason [=*Neogoniolithon notarisii* (Dufour) Hamel & Lemoine] |
| *Neogoniolithon mamillosum* (Hauck) Setchell & Mason [=*Lithophyllum hauckii* (Rothpletz) Lemoine] |
| *Pneophyllum confervicola* (Kützing) Chamberlain [=*Fosliella minutula* (Foslie) Ganesan; =*Melobesia confervicola* (Kützing) Foslie] |
| *Pneophyllum coronatum* (Rosanoff) Penrose [=*Pneophyllum caulerpae* (Foslie) Jones & Woelkerling; =*Pneophyllum rosanoffii* Chamberlain] |
| *Pneophyllum fragile* Kützing [=*Fosliella lejolisii* (Rosanoff) Howe] |
| *Pneophyllum zonale* (P.L. Crouan & H.M. Crouan) Chamberlain |
| *Spongites fruticulosa* Kützing [=*Lithothamnion ramulosum* Philippi; =*Lithothamnion fruticulosum* (Kützing) Foslie] |
| **Tenarea tortuosa* (Esper) Lemoine; [=*Tenarea undulosa* Bory; *Lithophyllum tortuosum* (Esper) Foslie]^11^ |
| *Titanoderma mediterraneum* (Foslie) Woelkerling (=*Litholepis mediterranea* Foslie) |
| **Titanoderma ramosissimum* (Heydrich) Bressan & Cabioch^12^ |
| **Titanoderma trochanter* (Bory) Benhissoune, Boudouresque, Perret-Boudouresque & Verlaque^12^ |
|  |
| Family Hapalidiaceae |
| *Boreolithon van-heurckii* (Heydrich in Chalon) A.S. Harvey & Woelkerling |
| *Choreonema thuretii* (Bornet) Schmitz |
| *Leptophytum bornetii* (Foslie) Adey (=*Lithothamnion bornetii* Foslie) |
| *Lithothamnion corallioides* (P.L. Crouan & H.M. Crouan) P.L. Crouan & H.M. Crouan [=*Lithothamnion solutum* (Foslie) Lemoine] |
| *Lithothamnion crispatum* Hauck |
| **Lithothamnion minervae* Basso (=*Lithothamnion fruticulosum* auct., pro parte) |
| *Lithothamnion philippi* Foslie |
| **Lithothamnion propontidis* Foslie |
| *Lithothamnion sonderi* Hauck |
| **Lithothamnion valens* Foslie |
| *Melobesia membranacea* (Esper) Lamouroux |
| *Mesophyllum alternans* (Foslie) Cabioch & Mendoza^13^ |
| *Mesophyllum expansum* (Philippi) Cabioch & Mendoza^14^ |
| *Mesophyllum lichenoides* (Ellis) Lemoine^13^ |
| **Mesophyllum macedonis* Athanasiadis |
| *Mesophyllum macroblastum* (Foslie) Adey |
| *Phymatolithon calcareum* (Pallas) Adey & McKibbin [=*Lithothamnion calcareum* (Pallas) Areschoug] |
| *Phymatolithon lenormandii* (Areschoug) Adey [=*Lithothamnion lenormandii* (Areschoug) Foslie] |
| *Phymatolithon purpureum* (P.L. Crouan & H.M. Crouan) Woelkerling & Irvine [=*Phymatolithon polymorphum* (Linnaeus) Foslie] |
| *Phymatolithon tenuissimum* (Foslie) Adey (=*Lithothamnion tenuissimum* Foslie) |
|  |
| Family Sporolithaceae |
| *Sporolithon ptychoides* Heydrich (=*Sporolithon mediterraneum* Heydrich)^15^ |
|  |
| *Order Gelidiales* |
|  |
| Family Gelidiaceae |
| *Gelidiella lubrica* (Kutzing) J. Feldmann & Hamel |
| **Gelidiella nigrescens* (J. Feldmann) J. Feldmann & Hamel |
| *Gelidiella ramellosa* (Kutzing) J. Feldmann & Hamel |
| *Gelidiella sanctarum* J. Feldmann & Hamel^5^ |
| **Gelidiocolax christianae* J. Feldmann & G. Feldmann |
| *Gelidium bipectinatum* Furnari (=*Gelidium pectinatum* Schousboe ex Montagne) |
| *Gelidium corneum* (Hudson) Lamouroux [=*Gelidium sesquipedale* (Clemente) Thuret] |
| *Gelidium crinale* (Hare ex Turner) Gaillon |
| *Gelidium microdon* Kützing^5^ |
| *Gelidium minusculum* (Weber van Bosse) R.E. Norris (=*Gelidium pusillum* var. *minusculum* Weber van Bosse) |
| *Gelidium pulchellum* (Turner) Kützing [=*Gelidium pulchellum* var. *claviferum* (Turner) Kützing] |
| *Gelidium pusillum* (Stackhouse) Le Jolis |
| *Gelidium reptans* (Suhr) Kylin^5^ |
| *Gelidium spathulatum* (Kutzing) Bornet |
| *Gelidium spinosum* (Gmelin) Silva (=*Gelidium latifolium* Bornet ex Hauck) |
| **Gelidium spinosum* (Gmelin) Silva var. *hystrix* (J. Agardh) Furnari |
| *Parviphycus antipai* (Celan) Santelices (=*Gelidiella antipai* Celan) |
| **Parviphycus felicinii* Perrone & Delle Foglie |
| *Parviphycus adnatus* (Dawson) Santelices (=*Gelidiella tenuissima* J. Feldmann & Hamel; =*Gelidiella pannosa* J. Feldmann & Hamel) |
| *Pterocladiella capillacea* (Gmelin) Santelices & Hommersand [=*Pterocladia capillacea* (Gmelin) Bornet] |
| *Pterocladiella melanoidea* (Schousboe ex Bornet) Santelices & Hommersand (=*Gelidium melanoideum* Schousboe ex Bornet) |
| *Pterocladiella melanoidea* var. *filamentosa* (Schousboe ex Bornet) Wynne |
| **Pterocladiella melanoidea* var. *gracilis* (J. Feldmann & Hamel) Wynne |
| **Ptilophora mediterranea* (Huvé) R.E. Norris (=*Beckerella mediterranea* H. Huvé) |
|  |
| *Order Gigartinales* |
|  |
| Family Areschougiaceae |
| *#Agardhiella subulata* (C. Agardh) Kraft & Wynne |
| *#Sarconema filiforme* (Sonder) Kylin |
| *#Sarconema scinaioides* Borgesen |
|  |
| Family Calosiphoniaceae |
| *Calosiphonia vermicularis* (J. Agardh) Schmitz [=*Calosiphonia dalmatica* (Kützing) Bornet & Flahault] |
| *Schmitzia neapolitana* (Berthold) Lagerheim ex Silva [=*Bertholdia neapolitana* (Berthold) Schmitz] |
|  |
| Family Caulacanthaceae |
| *Catenella caespitosa* (Withering) Irvine |
| *Caulacanthus ustulatus* (Mertens ex Turner) Kützing |
| *#Feldmannophycus okamurae* (Yamada) Mineur, Maggs & Verlaque |
| *Feldmannophycus rayssiae* (J. Feldmann & G. Feldmann) Augier & Boudouresque |
|  |
| Family Cruoriaceae |
| *Cruoria cruoriaeformis* (P.L. Crouan & H.M. Crouan) Denizot (=*Cruoria purpurea* P.L. Crouan & H.M. Crouan) |
|  |
| Family Cystocloniaceae^16^ |
| *Calliblepharis ciliata* (Hudson) Kützing |
| *Calliblepharis jubata* (Goodenough & Woodward) Kützing |
| *#Hypnea cornuta* (Kützing) J. Agardh |
| *#Hypnea flagelliformis* Graville ex J. Agardh |
| **Hypnea furnariana* Cormaci, Alongi & Dinaro |
| *Hypnea musciformis* (Wulfen) Lamouroux |
| *#Hypnea spinella* (C. Agardh) Kützing (=*Hypnea cervicornis* J. Agardh) |
| *#Hypnea valentiae* (Turner) Montagne |
| *Rhodophyllis divaricata* (Stackhouse) Papenfuss |
| **Rhodophyllis strafforelloi* Ardissone |
|  |
| Family Dumontiaceae |
| *Dudresnaya verticillata* (Withering) Le Jolis |
|  |
| Family Furcellariaceae |
| *Furcellaria lumbricalis* (Hudson) Lamouroux^17^ |
| *Halarachnion ligulatum* (Woodward) Kützing |
| **Neurocaulon foliosum* (Meneghini) Zanardini [=*Neurocaulon reniforme* (Postels & Ruprecht) Zanardini] |
|  |
| Family Gigartinaceae |
| *Chondracanthus acicularis* (Roth) Fredericq [=*Gigartina acicularis* (Roth) Lamouroux] |
| *Chondracanthus teedii* (Mertens ex Roth) Kützing [=*Gigartina teedii* (Mertens ex Roth) Lamouroux] |
| *#Chondrus giganteus* Yendo f. *flabellatus* Mikami |
| *Gigartina pistillata* (Gmelin) Stackhouse^18^ |
|  |
| Family Gloiosiphoniaceae |
| *Gloiosiphonia capillaris* (Hudson) Carmichael |
| *Thuretella schousboei* (Thuret) Schmitz |
|  |
| Family Kallymeniaceae |
| *Callophyllis laciniata* (Hudson) Kützing |
| *Kallymenia feldmannii* Codomier |
| **Kallymenia lacerata* J. Feldmann |
| **Kallymenia patens* (J. Agardh) Parkinson |
| *Kallymenia reniformis* (Turner) J. Agardh |
| *Kallymenia requienii* (J. Agardh) J. Agardh |
| **Kallymenia spathulata* (J. Agardh) Codomier ex Parkinson |
| *Meredithia microphylla* (J. Agardh) J. Agardh (=*Kallymenia microphylla* J. Agardh) |
|  |
| Family Peyssonneliaceae  **Ethelia van bosseae* J. Feldmann |
| **Metapeyssonnelia feldmanni* Boudouresque, Coppejans & Marcot-Coqueugniot |
| *Peyssonnelia armorica* (P.L. Crouan & H.M. Crouan) Weber van Bosse (=*Cruoriella armorica* P.L. Crouan & H.M. Crouan; =*Cruoriopsis cruciata* Dufour; *=Cruoriopsis rosenvingei* Boergesen) |
| *Peyssonnelia atropurpurea* P.L. Crouan & H.M. Crouan |
| **Peyssonnelia bornetii* Boudouresque & Denizot |
| *Peyssonnelia coriacea* J. Feldmann |
| **Peyssonnelia crispata* Boudouresque & Denizot |
| *Peyssonnelia dubyi* P.L. Crouan & H.M. Crouan [=*Cruoriella dubyi* P.L. Crouan & H.M. Crouan ex Kützing; =*Peyssonnelia codana* (Rosenvinge) Denizot] |
| *Peyssonnelia harveyana* P.L. Crouan & H.M. Crouan ex J. Agardh (=*Peyssonnelia adriatica* Hauck) |
| **Peyssonnelia hongii* Marcot-Coqueugniot^19^ |
| *Peyssonnelia immersa* Maggs & Irvine^20^ |
| *Peyssonnelia inamoena* Pilger |
| *Peyssonnelia magna* Ercegovic |
| *Peyssonnelia orientalis* (Weber van Bosse) Cormaci & Furnari |
| *Peyssonnelia polymorpha* (Zanardini) Schmitz |
| **Peyssonnelia rara-avis* Marcot & Boudouresque |
| **Peyssonnelia rosa marina* Boudouresque & Denizot |
| *Peyssonnelia rosa-marina* Boudouresque & Denizot f. *saxicola* Boudouresque & Denizot |
| *Peyssonnelia rubra* (Greville) J. Agardh |
| *Peyssonnelia squamaria* (Gmelin) Decaisne |
| **Peyssonnelia stoechas* Boudouresque & Denizot |
| *Polystrata compacta* (Foslie) Denizot |
| *Polystrata fosliei* (Weber van Bosse) Denizot |
|  |
| Family Phyllophoraceae |
| *Ahnfeltiopsis devoniensis* (Greville) Silva & DeCew [=*Gymnogongrus devoniensis* (Greville) Schotter]^21^ |
| *#Ahnfeltiopsis flabelliformis* (Harvey) Masuda |
| *Ahnfeltiopsis pusilla* (Montagne) Silva & DeCew [=*Gymnogongrus pusillus* (Montagne) J. Feldmann & Mazoyer] |
| *Coccotylus truncatus* (Pallas) Wynne & Heine [=*Phyllophora truncata* (Pallas) Zinova] |
| *Gymnogongrus crenulatus* (Turner) J. Agardh [=*Gymnogongrus norvegicus* (Gunnerus) J. Agardh] |
| *Gymnogongrus griffithsiae* (Turner) Martius (=*Actinococcus aggregatus* Schmitz) |
| *Gymnogongrus patens* (Goodenough & Woodward) J. Agardh^22^ |
| *Phyllophora crispa* (Hudson) Dixon [=*Phyllophora nervosa* (De Candolle) Greville] |
| *Phyllophora heredia* (Clemente) J. Agardh |
| *Phyllophora pseudoceranoïdes* (Gmelin) Newroth & A.R.A. Taylor^23^ |
| *Phyllophora sicula* (Kützing) Guiry & Irvine (=*Phyllophora palmettoides* J. Agardh) |
| *Schottera nicaeënsis* (Lamouroux ex Duby) Guiry & Hollenberg [=*Petroglossum nicaeense* (Lamouroux ex Duby) Schotter] |
| *Stenogramme interrupta* (C. Agardh) Montagne ex Harvey^5^ |
|  |
| Family Rhizophyllidaceae |
| **Contarinia peyssonneliaeformis* Zanardini (=*Rhizophyllis codii* J. Feldmann) |
| *Contarinia squamariae* (Meneghini) Denizot |
|  |
| Family Rissoëllaceae |
| *Rissoëlla verruculosa* (Bertoloni) J. Agardh |
|  |
| Family Sarcodiaceae |
| **Chondrymenia lobata* (Meneghini) Zanardini |
|  |
| Famly Solieriaceae |
| *#Solieria dura* (Zanardini) Schmitz |
| *#Solieria filiformis* (Kützing) Gabrielson |
| *Wurdermannia miniata* (Sprengel) J. Feldmann & Hamel |
|  |
| Family Sphaerococcacae |
| *Sphaerococcus coronopifolius* (Goodenough & Woodward) Stackhouse (=*Haematocelis fissurata* P.L. Crouan & H.M. Crouan) |
| **Sphaerococcus rhizophylloides* J.J. Rodriguez |
|  |
| *Order Gracilariales* |
|  |
| Family Gracilariaceae^2^ |
| *#Gracilaria arcuata* Zanardini |
| *Gracilaria armata* (C. Agardh) J. Agardh |
| *Gracilaria bursa-pastoris* (Gmelin) Silva [=*Gracilaria compressa* (C. Agardh) Greville] |
| *Gracilaria cervicornis* (Turner) J. Agardh |
| *Gracilaria conferta* (Schousboe ex Montagne) Montagne |
| **Gracilaria corallicola* Zanardini |
| **Gracilaria dendroides* Gargiulo, De Masi & Tripodi |
| *Gracilaria dura* (C. Agardh) J. Agardh |
| *Gracilaria foliifera* (Forsskal) Borgesen |
| *Gracilaria gracilis* (Stackhouse) Steentoft, Irvine & Farnham [=*Gracilaria verrucosa* (Hudson) Papenfuss; =*Gracilaria confervoides* (Linnaeus) Greville] |
| **Gracilaria heteroclada* (Montagne) J. Feldmann & G. Feldmann |
| *Gracilaria longa* Gargiulo, De Masi & Tripodi |
| *Gracilaria multipartita* (Clemente) Harvey |
| *Gracilariopsis longissima* (Gmelin) Steentoft, Irvine & Farnham |
|  |
| *Order Halymeniales* |
|  |
| Family Halymeniaceae |
| **Acrodiscus vidovichii* (Meneghini) Zanardini |
| **Aeodes marginata* (Roussel ex Montagne) Schmitz |
| *Cryptonemia lomation* (Bertoloni) J. Agardh |
| *Cryptonemia seminervis* (C. Agardh) J. Agardh^24^ |
| **Cryptonemia tunaeformis* (Bertoloni) Zanardini |
| *#Grateloupia asiatica* Kawaguchi & Wang |
| **Grateloupia cosentinii* Kützing |
| *Grateloupia dichotoma* J. Agardh |
| *Grateloupia doryphora* (Montagne) Howe |
| **Grateloupia filicina* (Lamouroux) C. Agardh^25^ |
| *#Grateloupia lanceolata* (Okamura) Kawaguchi |
| *#Grateloupia minima* P.L. Crouan & H.M. Crouan^26^ |
| *#Grateloupia patens* (Okamura) Kawaguchi & Wang (=*Prionitis patens* Okamura) |
| *Grateloupia proteus* Kützing |
| *#Grateloupia subpectinata* Holmes [=*Grateloupia luxurians* (A. Gepp & E.S. Gepp) Wilkes, McIvor & Guiry] |
| *#Grateloupia turuturu* Yamada |
| **Halymenia asymetrica* Gargiulo, De Masi & Tripodi |
| *Halymenia elongata* C. Agardh [=*Halymenia trigona* (Clemente) C. Agardh] |
| **Halymenia elongata* var. *decipiens* (J. Agardh) Cremades |
| **Halymenia elongata* var. *plana* (Codomier) Cremades |
| *Halymenia floresii* (Clemente) C. Agardh |
| **Halymenia floresii* var. *pinnata* Codomier |
| **Halymenia floresii* var. *ulvoidea* Codomier |
| *Halymenia latifolia* P.L. Crouan & H.M. Crouan ex Kützing (=*Halymenia trabeculata* Ercegovic) |
|  |
| *Order Hildenbrandiales* |
|  |
| Family Hildenbrandiaceae |
| *Hildenbrandia crouaniorum* J. Agardh (=*Hildenbrandia canariensis* Boergesen) |
| *Hildenbrandia occidentalis* Setchell (=*Hildenbrandia occidentalis* var. *lusitanica* Ardré) |
| *Hildenbrandia rubra* (Sommerfelt) Meneghini (=*Hildenbrandia prototypus* Nardo) |
|  |
| *Order Nemaliales* |
|  |
| Family Galaxauraceae |
| *#Galaxaura rugosa* (Ellis & Solander) Lamouroux |
| *Tricleocarpa fragilis* (Linné) Huisman & Townsend [=*Galaxaura oblongata* (Ellis & Solander) Lamouroux; =*Galaxaura adriatica* Zanardini] |
|  |
| Family Liagoraceae |
| *#Ganonema farinosum* (Lamouroux) Fan & Wang (=*Liagora farinosa* Lamouroux) |
| *Helminthocladia agardhiana* Dixon [=*Helminthocladia hudsonii* (C. Agardh) J. Agardh] |
| *Helminthora divaricata* (C. Agardh) J. Agardh [=*Helminthora stackhousei* (Clemente) Cremades & Pérez-Cirera] |
| *Liagora ceranoides* Lamouroux |
| *Liagora distenta* (Mertens ex Roth) Lamouroux |
| *Liagora tetrasporifera* Borgesen |
| *Liagora viscida* (Forsskal) C. Agardh |
| *Nemalion helminthoides* (Velley) Batters |
| *#Nemalion vermiculare* Suringar |
|  |
| Family Scinaiaceae |
| *Scinaia furcellata* (Turner) J. Agardh |
| *Scinaia interrupta* (De Candolle) Wynne [=*Scinaia turgida* Chemin; =*Scinaia subcostata* (J. Agardh) Chemin ex Hamel] |
| *Scinaia complanata* (Collins) Cotton |
| *Scinaia complanata* var. *intermedia* Boergesen |
|  |
| *Order Nemastomatales* |
|  |
| Family Nemastomataceae |
| *Nemastoma dichotomum* J. Agardh |
| **Nemastoma dichotomum* J. Agardh var. *caulescens* (Kützing) Rodriguez-Prieto, Verlaque & Verges (=*Nemastoma constrictum* Ercegovic) |
| **Nemastoma dichotomum* var. *biasolettianum* (Kützing) Rodriguez-Prieto, Verlaque & Verges |
| **Nemastoma dichotomum* var. *incrassatum* (Kützing) Rodriguez-Prieto, Verlaque & Verges |
| **Nemastoma dumontioides* J. Agardh |
| *Predaea ollivieri* J. Feldmann (=*Yadranella adriatica* Ercegovic) |
| *Predaea pusilla* (Berthold) J. Feldmann |
| **Predaea pusilla* f. *alboranensis* Conde, López-Mielgo & Flores-Moya |
|  |
| Family Schizymeniaceae |
| *Itonoa marginifera* (J. Agardh) Masuda & Guiry [=*Platoma marginiferum* (J. Agardh) Schmitz] |
| *Platoma cyclocolpum* (Montagne) Schmitz |
| *Schizymenia dubyi* (Chauvin ex Duby) J. Agardh (=*Haematocelis rubens* J. Agardh) |
|  |
| *Order Palmariales* |
|  |
| Family Rhodophysemataceae |
| *Rhodophysema georgii* Batters |
|  |
| *Order Plocamiales* |
|  |
| Family Plocamiaceae |
| *Plocamium cartilagineum* (Linnaeus) Dixon (=*Plocamium vulgare* Lamouroux) |
| *Plocamium raphelisianum* Dangeard^22^ |
| *#Plocamium secundatum* (Kützing) Kützing |
|  |
| *Order Sebdeniales* |
|  |
| Family Sebdeniaceae |
| *Sebdenia dichotoma* Berthold (=*Sebdenia feldmannii* Codomier) |
| **Sebdenia monardiana* (Montagne) Berthold |
| *Sebdenia rodrigueziana* (J. Feldmann) Codomier ex Parkinson (=*Halymenia mucosa* Ercegovic) |
|  |
| *Order Rhodymeniales* |
|  |
| Family Champiaceae |
| *Champia parvula* (C. Agardh) Harvey |
| **Chylocladia pelagosae* Ercegovic |
| *Chylocladia verticillata* (Lightfoot) Bliding [=*Chylocladia pygmaea* (Funk) Kylin; =*Chylocladia squarrosa* (Kützing) Thuret] |
| **Chylocladia verticillata* var. *kaliformis-unistratosa* (Ercegovic) Cormaci & Furnari |
| **Chylocladia verticillata* var. *kaliformis-unistratosa* f. *breviarticulata* (Ercegovic) Cormaci & Furnari |
| **Chylocladia wynnei* Alongi, Furnari & Cormaci |
| *Gastroclonium clavatum* (Roth) Ardissone |
| *Gastroclonium ovatum* (Hudson) Papenfuss^22^ |
| *Gastroclonium reflexum* (Chauvin) Kützing |
|  |
| Family Faucheaceae |
| *Gloiocladia furcata* (C. Agardh) J. Agardh |
| *Gloiocladia microspora* (Bornet in J.J. Rodriguez) Sánchez & Rodriguez-Prieto (=*Fauchea microspora* Bornet) |
| *Gloiocladia repens* (C. Agardh) Sánchez & Rodriguez-Prieto [=*Fauchea repens* (C. Agardh) Montagne & Bory] |
| **Leptofauchea coralligena* Rodriguez-Prieto & De Clerck |
|  |
| Family Lomentariaceae |
| *Lomentaria articulata* (Hudson) Lyngbye |
| *Lomentaria articulata* (Hudson) Lyngbye var. *linearis* Zanardini |
| *Lomentaria chylocladiella* Funk |
| **Lomentaria clavaeformis* Ercegovic |
| *Lomentaria clavellosa* (Turner) Gaillon |
| **Lomentaria clavellosa* f. *reducta* Ercegovic |
| *Lomentaria clavellosa* var. *conferta* (Meneghini) J. Feldmann |
| **Lomentaria compressa* (Kützing) Kylin |
| **Lomentaria ercegovicii* Verlaque, Boudouresque, Meinesz, Giraud & Marcot-Coqueugniot (=*Lomentaria tenera* Ercegovic) |
| *Lomentaria firma* (J. Agardh) Kylin |
| **Lomentaria firma* f. *compressa* Ercegovic |
| *#Lomentaria flaccida* Tanaka |
| *#Lomentaria hakodatensis* Yendo |
| **Lomentaria jabukae* Ercegovic |
| *Lomentaria subdichotoma* Ercegovic |
| *Lomentaria uncinata* Meneghini ex Zanardini |
| **Lomentaria verticillata* Funk |
|  |
| Family Rhodymeniaceae |
| *Botryocladia botryoides* (Wulfen) J. Feldmann |
| *Botryocladia chiajeana* (Meneghini) Kylin |
| *#Botryocladia madagascariensis* G. Feldmann |
| *Botryocladia microphysa* (Hauck) Kylin |
| *Cordylecladia erecta* (Greville) J. Agardh^27^ |
| *Cordylecladia guiry* Gargiulo, Furnari & Cormaci |
| *Chrysymenia ventricosa* (Lamouroux) J. Agardh |
| *#Chrysymenia wrightii* (Harvey) Yamada |
| *Irvinea boergesenii* (J. Feldmann) Wilkes, McIvor & Guiry (=*Botryocladia boergesenii* J. Feldmann) |
| *Rhodymenia ardissonei* (Kuntze) J. Feldmann |
| *Rhodymenia caespitosa* P. Dangeard^5^ |
| *Rhodymenia delicatula* Dangeard |
| *#Rhodymenia erythraea* Zanardini |
| *Rhodymenia ligulata* Zanardini |
| *Rhodymenia pseudopalmata* (Lamouroux) Silva [=*Rhodymenia palmetta* (Stackhouse) Greville] |
| **Rhodymeniocolax mediterraneus* Verges, Izquierdo & Verlaque |
| **Class Porphyridiophyceae** |
|  |
| *Order Porphyridiales* |
|  |
| Family Phragmonemataceae |
| *#Goniotrichopsis sublittoralis* Smith |
|  |
| **Class Stylonematophyceae** |
|  |
| *Order Stylonematales* |
|  |
| Family Stylonemataceae |
| *Chroodactylon ornatum* (C. Agardh) Basson [=*Asterocytis ornata* (C. Agardh) Hamel] |
| *Rhodosorus marinus* Geitler^28^ |
| *Stylonema alsidii* (Zanardini) K.M. Drew [=*Goniotrichum alsidii* (Zanardini) Howe] |
| *Stylonema cornu-cervi* Reinsch [=*Goniotrichum cornu-cervi* (Reinsch) Hauck] |
| *Stylonema subcoeruleum* (Dangeard) Wynne (=*Goniotrichum subcoeruleum* Dangeard)^29^ |

**Notes**

1. Some species from the genus *Porphyra* require confirmation.

2. Requires nomenclatural and taxonomical re-investigation.

3. Known only from the type description (Feldmann 1931). Requires taxonomical re-investigation.

4. According to Athanasiadis (2009), Mediterranean records of *Antithamnion nipponicum* Yamada & Inagaki and *Antithamnion pectinatum* (Montagne) Brauner ex Athanasiadis & Tittley correspond to *Antithamnion hubbsii*.

5. An Atlantic species recorded by Kazzaz (1989) in the Strait of Gibraltar.

6. See Verlaque (2001).

7. See comments by Gómez-Garreta et al. (2001) concerning taxonomical and nomenclatural problems with entities recorded as *Acrosorium reptans* (P.L. Crouan & H.M. Crouan) Kylin and *Acrosorium uncinatum* (Turner) Kylin.

8. See Gómez-Garreta et al. (2001).

9. Requires nomenclatural and taxonomical re-investigation (Gómez-Garreta et al. 2001).

10. Genus *Polysiphonia* Greville requires nomenclatural and taxonomical re-investigation.

11. According to Algaebase this entity should be named *Lithophyllum tortuosum* (Esper) Foslie. We follow Bressan & Babbini-Benussi (2003) for nomenclature.

12. See Bressan & Cabioch (2004) for circumscription of *Titanoderma trochanter* and *Titanoderma ramosissimum*.

13. See Cabioch & Mendoza (1998).

14. See Cabioch & Mendoza (2003).

15. See Alongi et al. (1996).

16. Due to taxonomical complexity and probable misidentifications in the genus *Hypnea*, we only list the two autochtonous species *Hypnea musciformis* and *Hypnea furnariana*, as well as the introduced species reported by Verlaque et al. (in press). Thus, we do not consider the following species as being established in the Mediterranean: *Hypnea variabilis* Okamura (Zeybek et al. 1986; Taskin et al. 2008), *Hypnea nidifica* J. Agardh (not considered by Zenetos et al. 2005), *Hypnea esperi* Bory (not considered by Zenetos et al. 2005) and *Hypnea spicifera* (Suhr) Harvey (=*Hypnea harveyi* Kützing) reported by Boudouresque & Verlaque (2002).

17. Athanasiadis (1987) excludes *Furcellaria lumbricalis* from the Aegean Sea but according to Furnari et al. (2003) the species is present in Sicily and Sardinia. It has been also reported from southern Spain (Conde et al. 1996).

18. Athanasiadis (1987) and Furnari et al. (2003) exclude this species from the Aegean Sea and Sicily, respectively, but the species seems to be present in Algeria, Morocco and southern Spain (Perret-Boudouresque & Seridi 1989; Benhissoune et al. 2002; Conde et al. 1996).

19. Known for sure only from the type description (Marcot-Coqueugniot 1980; Verlaque 1987). Requires taxonomical re-investigation.

20. Apart from the single record by Athanasiadis (1987) there is no further evidence of the occurrence of this species in the Mediterranean.

21. Atlantic species, known from the Mediterranean coast of southern Spain (Fernández et al. 1983).

22. Atlantic species, reported only from the Mediterranean coast of Morocco and southern Spain, close to the strait of Gibraltar (Conde et al. 1996; Benhissoune et al. 2002).

23. Athanasiadis excludes this Atlantic species from the Aegean Sea, but it has also been reported from Turkey (Taskin et al. 2008) and Tunisia (Ben Maïz et al. 1987).

24. *Cryptonemia seminervis* is an Atlantic species closely related to *Cryptonemia lomation* and according to Athanasiadis (1987) the two entities may be conspecific. Mediterranean citations of *Cryptonemia seminervis* should be confirmed.

25. See de Clerk et al. (2005) regarding the cryptic diversity associated to this species. According to their conclusions the genuine *Grateloupia filicina* seems to be limited to the Mediterranean basin and, thus, considered here as endemic. Whether plants collected near Gibraltar belong to *Grateloupia filicina* or to the reinstated *Grateloupia minima* P.L. Crouan & H.M. Crouan requires further investigations.

26. Reported by Zenetos et al. (2008) as introduced, although the determination of the alien or native status of Mediterranean populations requires further investigations. See also note 25.

27. According to Gargiulo et al. (1990) further investigations are required to confirm the presence of *Cordylecladia erecta* in the Mediterranean Sea.

28. Reported by Fresnel & Billard (1995) from Corsica and Syria.

29. Reported only once from the Eastern Pyrenees (Boudouresque et al. 1984).

*Taxa inquirenda* (see also Gómez-Garreta et al. 2001)

*Acrochaetium cheminii* J. Feldmann, *nomen nudum*

*Acrochaetium extensum* Ercegovic: see Furnari et al. (2003).

*Acrochaetium extensum* var. *longicellulare* Ercegovic: entity of uncertain taxonomic position.

*Acrochaetium griffithsianum* Nägeli: entity of uncertain taxonomic position.

*Acrochaetium lenormandii* (Suhr ex Kützing) Nägeli: see Furnari et al. (2003)

*Acrochaetium pallens* (Zanardini) Nägeli: see Furnari et al. (2003).

*Callithamnion piliferum* Kützing: entity of uncertain taxonomic position (Furnari et al. 1999).

*Chaetangium dichotomum* Kützing: see Furnari et al. (2003).

*Chylocladia breviramosa* Solander: see Furnari et al. (2003).

*Chylocladia scaliformis* Harvey: see Furnari et al. (2003).

*Fosliella ischiensis* Coppejans, *nomen nudum*.

*Gelidium affine* Schiffner: entity of uncertain taxonomic position.

*Gelidium claviferum* Kützing: entity of uncertain taxonomic position.

*Gelidium divergens* J. Agardh: see Furnari et al. (2003).

*Gelidium radicans* Montagne: see Furnari et al. (2003).

*Gelidium secundatum* Zanardini ex Kützing: see Furnari et al. (2003)

*Gelidium venetum* Schiffner: see Furnari et al. (2003)

*Goniotrichum alsidii* (Zanardini) Howe var. *strictum* Schiffner: entity of uncertain taxonomic position.

*Gymnogongrus dubius* (Montagne) Schotter: entity of uncertain taxonomic position.

*Halymenia hvari* Ercegovic: entity of uncertain taxonomic position.

*Halymenia pluriloba* Ercegovic: entity of uncertain taxonomic position.

*Halymenia rhodymenioides* Ercegovic: entity of uncertain taxonomic position.

*Helminthocladia stackhousei* (Clemente) Cremades & Pérez-Cirera: see comments in Benhissoune et al. (2002).

*Lithophyllum laeve* Kützing: see Furnari et al. (2003).

*Lithophyllum polycarpum* Zanardini: entity of uncertain taxonomic position (Furnari et al. 1999).

*Lithophyllum tarentinum* Mastrorilli: see Furnari et al. (2003).

*Lithoporella melobesioides* (Foslie) Foslie: see Furnari et al. (2003) as *Melobesia melobesioides* (Foslie) Lemoine.

*Lithothamnion elegans* Zanardini, *nomen nudum* (Furnari et al. 2003).

*Lithothamnion validum* Foslie: entity of uncertain taxonomic position (Furnari et al. 1999).

*Melobesia confervoides* Funk: see Furnari et al. (2003).

*Melobesia polycarpa* Zanardini: entity of uncertain taxonomic position (Furnari et al. 1999).

*Microgelidiopsis horrida* Ercegovic, *nomen nudum*.

*Nithophyllum magontanum* J.J. Rodriguez, *nomen nudum*.

*Phyllophora fimbriata* Ercegovic: entity of uncertain taxonomic position.

*Plocamium subtile* Kützing [=*Plocamium coccineum* var. *uncinatum* (C. Agardh) J. Agardh].

*Porphyra autumnalis* Zanardini (=*Porphyra laciniata* C. Agardh var. *umbilicata* C. Agardh?): entity of uncertain taxonomic position (Furnari et al. 1999).

*Rhodymenia leptofaucheoides* P. & H. Huvé: described by Huvé & Huvé (1971) it has not been reported again and it is not included in the Tunisian checklist by Ben Maïz et al. (1987).

*Turnerella rosacea* (J. Agardh) Schmitz: entity of uncertain taxonomic position.

*Taxa excludenda* (see also Gómez-Garreta et al. 2001)

*Acanthophora muscoides* (Linnaeus) Bory: unsupported records (Zenetos et al. 2008).

*Acrochaetium balticum* (Rosenvinge) Aleem & Schulz: misidentification (Zenetos et al. 2008).

*Acrochaetium porphyrae* (K.M. Drew) Smith: see discussion in Perret & Seridi (1989).

*Acrochaetium radiatum* Jao: see Furnari et al. (2003).

*Aglaothamnion hookeri* (Dillwyn) Maggs & Hommersand: see comments in Gómez-Garreta et al. (2001) and Furnari et al. (2003).

*Anotrichium crinitum* (Kützing) Bladock: see Furnari et al. (2003).

*Antithamnion densum* (Suhr) Howe: misidentification (Zenetos et al. 2008).

*Antithamnion nipponicum* Yamada & Inagaki: according to Athanasiadis (2009), this binomial is an heterotypic synonym of *Antithamnion pectinatum*.

*Antithamnion pectinatum* (Montagne) Brauner in Athanasiadis & Tittley: according to Athanasiadis (2009), Mediterranean records of *Antithamnion nipponicum* (=*Antithamnion pectinatum*) correspond to *Antithamnion hubbsii*.

*Chondria collinsiana* Howe: unsupported records (Zenetos et al. 2008).

*Chondria polyrhiza* Collins & Hervey: unsupported records (Zenetos et al. 2008).

*Chondrus crispus* Stackhouse: see Furnari et al. (2003) and Zenetos et al. (2008).

*Corallina muscoides* Kützing: see Furnari et al. (2003).

*Gelidiopsis intricata* (C. Agardh) Vickers: reported from the Adriatic Sea by Giaccone (1978) as *Gelidium intricatum* Kützing, with doubts.

*Gelidium pteridifolium*: see Furnari et al. (2003).

*Gracilaria disticha* (J. Agardh) J. Agardh: needs confirmation (Zenetos et al. 2005).

*Gracilaria divergens* (C. Agardh) J. Agardh: introduced species reported from Mare Piccolo (Italy), Furnari et al. (2003) considered it as "taxa excludenda". Not reported by Verlaque et al. (in press).

*Gracilaria ornata* Areschoug: reported only by Parenzan (1983); this record probably corresponds to a misidentification.

*Grania efflorescens* (J. Agardh) Kylin [=*Acrochaetium efflorescens* (J. Agardh) Nägeli]: reported by Gómez et al. (1981); this record probably corresponds to a misidentication.

*Grateloupia lanceola* (J. Agardh) J. Agardh: it is not clear whether this species has been reported from the Mediterranean coasts from southern Spain (see Conde et al. 1996).

*Heterosiphonia japonica*: Mediterranean records of this species correspond to *Dasysiphonia* sp. (Zenetos et al. 2008).

*Hypnea esperi* Bory: see Zenetos et al. (2005).

*Hypnea nidifica* J. Agardh: needs confirmation (Zenetos et al. 2005).

*Hypnea spicifera* (Suhr) Harvey: Mediterranean records of this species correspond to *Hypnea flagelliformis* Greville ex J. Agardh (Zenetos et al. 2008).

*Hypnea variabilis* Okamura: unsupported records (Zenetos et al. 2008).

*Jania longiarthra* Dawson: reported only by Ballesteros (1990); specimens probably correspond to *Jania longifurca*.

*Laurencia japonica* Yamada: misidentification (Zenetos et al. 2008).

*Laurencia radicans* (Kützing) Kützing: a Black Sea species reported from southern Italy and considered as "*taxa inquirenda*" by Furnari et al. (2003). Not reported in the Mediterranean checklist of species belonging to the *Laurencia* complex (Serio et al. 2008)

*Lithophyllum glaciale* Kjellman: reported only by Gómez-Menor & Fuertes (1982) from the island of Tabarca; unsupported record.

*Lithophyllum vickersiae* Lemoine: reported only by Gómez-Menor & Fuertes (1982) from the island of Tabarca; unsupported record.

*Lithothamnion australe*: see Furnari et al. (2003).

*Mastocarpus stellatus* (Stackhouse) Guiry: misidentification (Furnari et al. 2003; Zenetos et al. 2005).

*Meiodiscus spetsbergensis* (Kjellman) Saunders & McLachlan: A northern species whose record as south as Morocco requires confirmation (Benhissoune et al. 2002).

*Metamastophora flabellata* (Sonder) Setchell: see Furnari et al. (2003).

*Nothogenia erinacea* (Turner) Parkinson: see Furnari et al. (2003).

*Palmaria palmata* (Linnaeus) Kuntze: see Furnari et al. (2003).

*Phyllophora traillii* Holmes ex Batters: reported from Northeastern Spain (Ballesteros & Romero 1982); it requires confirmation (Athanasiadis 2002).

*Phymatolithon brunneum* Chamberlain: see Furnari et al. (2003).

*Polyides rotundus* (Hudson) Gaillon: see Conde et al. (1996) and Furnari et al. (2003).

*Polysiphonia kampsaxii* Boergesen: unsupported records (Zenetos et al. 2008).

*Pterothamnion simile* (Hooker & Harvey) Nägeli: misidentification (Zenetos et al. 2008).

*Rhodophysema elegans* (P.L. & H.M. Crouan ex J. Agardh) Dixon f. *polystromatica* (Batters) Dixon: see Furnari et al. (2003).

*Rhodymenia holmesii* Ardissone: This Atlantic species was reported from Tuscany by Papi et al. (1992) but it probably is a misidentification.

*Spongites absimile* (Foslie) Howe: reported only by Gómez-Menor & Fuertes (1982) from the island of Tabarca; unsupported record.

*Sporolithon molle* (Heydrich) Heydrich: see Alongi et al. (1996).

*Turnerella pennyi* (Harvey) Schmitz: see Furnari et al. (2003).

**References**

Alongi G, Cormaci M, Furnari G. 1996. On the occurrence of *Sporolithon ptychoides* Heydrich (Corallinales, Sporolithaceae, Rhodophyta) in the Mediterranean Sea. *Cryptogamie Algologie* 17: 131-137.

Alongi G, Cormaci M, Furnari G. 2007. *Woelkerlingia minuta* gen. et sp. nov. from the Mediterranean Sea and a reassessment of the genus *Lomathamnion*, with a description of two genera: *Hommersandiella* gen. nov. and *Stegengaea* gen. nov. *Cryptogamie Algologie* 28: 311-324.

Alongi G, Cormaci M, Furnari G. 2008. *Chylocladia wynnei* sp. nov. (Champiaceae, Rhodophyta) from the Mediterranean Sea. *Botanica Marina* 51: 145-150.

Athanasiadis A. 1987. *A survey of the seaweeds of the Aegean Sea with taxonomic studies on species of the tribe Antithamnieae (Rhodophyta)*. Göterna, Kungälv, 174 pp.

Athanasiadis A. 1999. *Mesophyllum macedonis* sp. nov. (Rhodophyta, Corallinales), a putative Tethyan relic in the north Aegean Sea. *European Journal of Phycology* 34: 239-252.

Athanasiadis A. 2002. Taxonomy and systematics of Rhodophyta with reference to the Mediterranean taxa. *Flora Mediterranea* 12: 93-167.

Athanasiadis A. 2009. Typification of *Antithamnion nipponicum* Yamada & Inagaki (Antithamnieae, Ceramioideae, Ceramiaceae, Ceramiales, Rhodophyta). *Botanica Marina* 52: 256-261.

Ballesteros E. 1990. Check-list of benthic marine algae from Catalonia (Northwestern Mediterranean). *Treballs Institut Botànic de Barcelona* 13: 1-52.

Ballesteros E, Romero J. 1982. Catálogo de las algas bentónicas (con exclusión de las diatomeas) de la costa catalana. *Collectanea Botanica* 13: 723-765.

Ben Maïz N, Boudouresque CF, Ouahchi F. 1987. Inventaire des algues et phanérogames marines benthiques de la Tunisie. *Giornale Botanico Italiano* 121: 259-304.

Benhissoune S, Boudouresque CF, Perret-Boudouresque M, Verlaque M. 2002. A checklist of the seaweeds of the Mediterranean and Atlantic coasts of Morocco. III. Rhodophyceae (excluding Ceramiales). *Botanica Marina* 45: 391-412

Benhissoune S, Boudouresque CF, Perret-Boudouresque M, Verlaque M. 2003. A checklist of the seaweeds of the Mediterranean and Atlantic coasts of Morocco. IV. Rhodophyceae-Ceramiales. *Botanica Marina* 46: 55-68.

Boudouresque CF, Perret-Boudouresque M. 1987. *A checklist of the benthic marine algae of Corsica*. GIS Posidonie Pubblication. Marseille. 121 pp.

Boudouresque CF, Perret-Boudouresque M, Knoepffler-Péguy M. 1984. Inventaire des algues marines benthiques dans les Pyrénées Orientales (Méditerranée, France). *Vie Milieu* 34: 41-59.

Boudouresque CF, Verlaque M. 2002. Biological pollution in the Mediterranean Sea: invasive versus introduced macrophytes. *Marine Pollution Bulletin* 44: 32-38.

Bressan G, Babbini-Benussi L. 1996. Phytoceanographical observations on coralline algae (Corallinales) in the Mediterranean Sea. *Rendiconti Accademia Lincei* *Scienze Fisiche e Naturali* 9(7): 179-207.

Bressan G, Babbini-Benussi L. 2003. Corallinales del Mar Mediterraneo: guida alla determinazione. *Biologia Marina Mediterranea* 10 (suppl. 2): 1-237.

Bressan G, Cabioch J. 2004. *Titanoderma trochanter* (Bory) Benhissoune, Boudouresque, Perret Boudouresque & Verlaque, et *Titanoderma ramosissimum* (Heydrich) comb. nov. (Corallinales, Rhodophytes), une rédefinition. *Cahiers de Biologie Marine* 45: 225-242.

Cabioch J, Mendoza ML. 1998. *Mesophyllum alternans* (Foslie) comb. nov. (Corallinales, Rhodophyta), a mediterraneo-atlantic species and new considerations on the *Lithothamnion philippi* Foslie complex. *Phycologia* 37: 208-221.

Cabioch J, Mendoza ML. 2003. *Mesophyllum expansum* (Philippi) comb. nov. (Corallinales, Rhodophytes), et mise au point sur les *Mesophyllum* des mers d'Europe. *Cahiers de Biologie Marine* 44: 257-273.

Conde F. 1991. Catálogo y comentarios sobre el "complejo *Audouinella*" (Acrochaetiaceae, Rhodophyceae) en el Mediterráneo. *Cryptogamie Algologie* 12: 163-170.

Conde F, Flores-Moya A, Altamirano M, Sánchez A. 1996. Check-list of Andalusia (S. Spain) seaweeds. III. Rhodophyceae. *Acta Botanica Malacitana* 21: 7-33.

Cormaci M, Alongi G, Dinaro R. 1993. *Hypnea furnariana* sp. nov. (Hypneaceae, Rhodophyta) from eastern Sicily (Mediterranean Sea). *Nordic Journal of Botany* 13: 227-231.

De Clerck O, Gavio B, Fredericq S, Bárbara I, Coppejans E. 2005. Systematics of *Grateloupia filicina* (Halymeniaceae, Rhodophyta), based on rbcL sequence analyses and morphological evidence, including the reinstatement of *G. minima* and the description of *G. capensis* sp. nov. *Journal of Phycology* 41: 391-410.

Feldmann J. 1931. Contribution à la flore algologique marine de l'Algérie. Les algues de Cherchell. *Bulletin de la Societé d'Histoire Naturelle de l'Afrique du Nord* 22: 179-254.

Fernández JA, Niell FX, Conde F. 1983. Sobre la entidad taxonómica de los *Gymnogongrus* Martius, 1928, foliosos en las costas de Málaga y Cádiz (SE de la Península Ibérica. *Investigación Pesquera* 47: 161-165.

Fresnel J, Brillard C. 1995. Une nouvelle microalgue rouge marine: *Rhodosorus magnei* sp. nov. (Rhodophyceae, Porphyridiales). *Cryptogamie Algologie* 16: 63-75.

Furnari G, Cormaci M, Serio D. 1999. Catalogue of the benthic marine macroalgae of the Italian coast of the Adriatic Sea. *Bocconea* 12: 1-214.

Furnari G, Cormaci M, Serio D. 2001. The *Laurencia* complex (Rhodophyta, Rhodomelaceae) in the Mediterranean Sea: an overview. *Cryptogamie Algologie* 22: 331-373.

Furnari G, Boisset F, Cormaci M, Serio D. 2002. The characterization of *Chondrophycus tenerrimus* (Cremades) comb. nov. (Ceramiales, Rhodophyta), a species often misidentified as *C. papillosus* (C. Agardh) Garbary et J. Harper in the Mediterranean Sea. *Cryptogamie Algologie* 23: 223-235.

Furnari G, Giaccone G, Cormaci M, Alongi G, Serio D. 2003. Biodiversità marina delle coste italiane: catalogo del macrofitobentos. *Biologia Marina Mediterranea* 10(1): 1-482.

Gallardo T, Gómez-Garreta A, Ribera MA, Álvarez M, Conde F. 1985*. A preliminary checklist of Iberian benthic marine algae*. Real Jardín Botánico Madrid. 83 pp.

Gargiulo GM, De Masi F, Tripodi F. 1986. Structure and reproduction of *Halymenia asymmetrica* sp. nov. (Rhodophyta) from the Mediterranean Sea. *Phycologia* 25: 144-151.

Gargiulo GM, De Masi F, Tripodi G. 1992. Morphology, reproduction and taxonomy of the Mediterranean species of *Gracilaria* (Gracilariales, Rhodophyta). *Phycologia* 31: 53-80.

Gargiulo GM, Furnari G, Cormaci M. 1990. Structure and reproduction of *Cordylecladia guiry* sp. nov. (Rhodophyta, Rhodymeniales) from the Mediterranean Sea. *Giornale Botanico Italiano* 124: 631-640.

Giaccone G. 1978. Revisione della flora marina del mare Adriatico. *Annuario Parco Marino di Miramare (supplemento)* 6(19): 1- 118.

Gómez-Garreta A, Gallardo T, Ribera MA, Cormaci M, Furnari G, Giaccone G, Boudouresque CF. 2001. Checklist of Mediterranean seaweeds III. Rhodophyceae Rabenh. 1. Ceramiales Oltm. *Botanica Marina* 44: 425-460.

Gómez A, Ribera MA, Romero J. 1981. Addicions a la flora algològica del Principat de Catalunya. *Butlletí de la Institució Catalana d'Història Natural* 46: 75-78.

Gómez-Menor JM, Fuertes E. 1982. Contribución al estudio de las algas rojas de la isla de Tabarca (Alicante). *Collectanea Botanica* 13: 865-872.

Huvé P, Huvé H. 1971. Une nouvelle espèce de *Rhodymenia* des côtes orientales de Tunisie: *Rhodymenia leptofaucheoides* nov. sp. (Rhodophycées, Rhodyméniales, Rhodyméniacées). *Bulletin Societé Botanique de France* 16: 51-60.

Kazzaz M. 1989. *Contribution à l'étude de la flore algale marine de la région ouest de la Méditerranée.* Thèse 3ème cycle, Université Mohammed V, Faculté des Sciences de Rabat, Morocco, 246 pp.

Knoepffler M, Noailles MC, Boudouresque CF, Abelard C. 1990. Phytobenthos des Pyrénées Orientales: complément à l'inventaire-présence d'espèces non indigènes (*Sargassum* et *Undaria*). *Bulletin Societé Zoologique de France* 115: 37-43.

Marcot-Coqueugniot J. 1980. Recherches sur le genre *Peyssonnelia*. XIII. Sur un *Peyssonnelia* du complexe "*harveyana*". *Botanica Marina* 23: 35-39.

Mayhoub H. 1976. *Recherches sur la végétation marine de la côte syrienne. Étude expérimentale sur la morphogénèse et le développement de quelques espèces peu connues.* Thèse de Sciences Naturelles, Université de Caen. 286 pp.

Nizamuddin M, West JE, Meñez EG. 1979. A list of marine algae from Libya. *Botanica Marina* 22: 465-476.

Papi I, Pardi G, Lenzini S, Benedetti Cecchi L, Cinelli F. 1992. Benthic marine flora in the Tuscan Archipelago. A first contribution: Isles of Capraia, Elba, Formiche di Grosseto, Scoglio d'Africa, Montecristo and Giannutri. *Giornale Botanico Italiano* 126: 549-593.

Parenzan P. 1983. *Fondo a Gracilaria. In: Puglia marittima. Aspetti geologici e biologia marina*. Congedo, Galatina (Lecce) vol II: 469-472.

Perret-Boudouresque M, Seridi H. 1989. *Inventaire des algues marines benthiques d'Algerie*. GIS Posidonie Pubblication. 116 pp.

Perrone C, Delle Foglie CI. 2006. *Parviphycus felicinii* sp. nov. (Gelidiales, Rhodophyta) from south-east Italy. *Cryptogamie Algologie* 27: 199-209.

Rodriguez-Prieto C, De Clerck O. 2009. *Leptofauchea coralligena* (Faucheaceae, Rhodophyta), a new species from the Mediterranean Sea. *European Journal of Phycology* 44: 107-121.

Rodriguez-Prieto C, Verges A, Sánchez N, Polo L, Verlaque M. 2004. The morphology and reproductive structures of Mediterranean species of the genus *Nemastoma* J. Agardh, nom. cons. (Nemastomataceae, Nemastomatales): *Nemastoma dichotomum* and *N. dumontioides*. *Botanica Marina* 47: 38-52.

Serio D, Furnari G, Cormaci M. 2004. On the occurrence of *Laurencia pyramidalis* Bory ex Kützing (Rhodophyta, Rhodomelaceae) in the Mediterranean Sea. *Cryptogamie Algologie* 25: 329-336.

Serio D, Petrocelli A, Cormaci M, Cecere E, Furnari G. 2008. First record of *Osmundea oederi* (Gunnerus) G. Furnari comb. nov. (Rhodomelaceae, Rhodophyta) from the Mediterranean Sea. *Cryptogamie Algologie* 29: 119-127.

Serio D, Cormaci M, Furnari G, Boisset F. 2010. First record of *Palisada maris-rubri* (Ceramiales, Rhodophyta) from the Mediterranean Sea along with three proposed transfers to the genus *Palisada*. *Phycological Research* 58: 9-16.

Taskin E, Özturk M, Kurt O, Özturk M. 2008. *The check-list of the marine algae of Turkey*. Manisa, Turkey. Ecem Kirtasiye. 87 pp.

Verlaque M. 1987. *Contribution à l'étude du phytobenthos d'un ecosystème photophile thermophile en Méditerranée Occidentale*. Thèse d'État. Université d'Aix-Marseille II. Luminy. 389 pp.

Verlaque M. 2001. Checklist of the macroalgae of the Thau lagoon (Hérault, France), a hot spot of marine species introduction in Europe. *Oceanologica Acta* 24: 29-49.

Verlaque M, Ruitton S, Mineur F, Boudouresque CF. in press. 4. Macrophytes. In: Briand F (ed), *CIESM Atlas of Exotic species in the Mediterranean*. CIESM Publishers. Monaco.

Zenetos A, Çinar ME, Pancucci-Papadopoulou MA, Harmelin JG, Furnari G, Andaloro F, Bellou N, Streftaris N, Zibrowius H. 2005. Annotated list of marine alien species in the Mediterranean with records of the worst invasive species. *Mediterranean Marine Science* 6: 63-118.

Zenetos A, Meriç E, Verlaque M, Galli P, Boudouresque CF, Giangrande A, Çinar ME, Bilecenoglu M. 2008. Additions to the annotated list of marine alien biota in the Mediterranean with special emphasis on Foraminifera and Parasites. *Mediterranean Marine Science* 9: 119-165.

## **Table S11. Checklist of the phylum Chlorophyta and comments to the checklist**

Algae belonging to the phylum Chlorophyta (Kingdom Plantae) have been classified by Classes, Orders, Families, Species and other infraspecific levels (subspecies, varieties and forms). Ordination is alphabetical. When no infraspecific level is indicated the taxa corresponds to the type variety.

This list is based in the check-list by Gallardo et al. (1993), with some updates and modifications. Particular attention has been devoted to local checklists published after the revision by Gallardo et al. (1993): Puglia (Southern Italy) (Cormaci et al., 2001), Tuscany (Northwestern Italy) (Rindi et al. 2002), Morocco (Benhissoune et al. 2001) and Italian coasts (Furnari et al. 2003). Lists of Mediterranean introduced species have also been considered (e.g. Verlaque 2001; Boudouresque & Verlaque 2002; Zenetos et al. 2005, 2008; Verlaque et al. in press). Black Sea is not included.

Taxonomy follows Algaebase (www.algaebase.org) if not otherwise indicated. Endemic species (found only in the Mediterranean basin, Black Sea included) are preceded by the symbol *. A careful and critical examination of the existing records of every species from the available literature has been performed in order to consider a species as endemic. Introduced species are preceded by the symbol #. As it is not always easy to detect introduced species from the distributional records, we mainly follow the criteria by other authors specialized in this issue. Superscript numbers refer to notes. Some of the commonest synonyms found in Mediterranean literature (if any) are sometimes placed after the currently accepted name. Other synonyms can be found at Algaebase. A list of *taxa inquirenda* as well as a list of *taxa excludenda* is also given at the end of the list; species considered as *taxa inquirenda* or *taxa excludenda* in Gallardo et al. (1993) are not stated again.

| **Phylum Chlorophyta** |
| --- |
|  |
| **Class Bryopsidophyceae** |
|  |
| *Order Bryopsidales* |
|  |
| Family Bryopsidaceae^1^ |
| *Blastophysa rhizopus* Reinke (=*Blastophysa polymorpha* Kjellman) |
| *Bryopsidella neglecta* (Berthold) Rietema [=*Derbesia neglecta* Berthold; *Bryopsidella halymeniae* (Berthold) J. Feldmann] |
| **Bryopsidella ostreobiformis* Calderón-Sáenz & Schnetter |
| *Bryopsis adriatica* (J. Agardh) Frauenfeld |
| *Bryopsis corymbosa* J. Agardh |
| *Bryopsis cupressina* Lamouroux (=*Bryopsis penicillata* Kützing) |
| **Bryopsis dichotoma* De Notaris |
| *Bryopsis duplex* De Notaris [=*Bryopsis balbisiana* Lamouroux; =*Bryopsis disticha* (J. Agardh) Kützing] |
| *Bryopsis feldmannii* Gallardo & Furnari (=Bryopsis *cupressoides* J. Feldmann) |
| *Bryopsis hypnoides* Lamouroux (=*Bryopsis monoica* Berthold ex Funk) |
| *Bryopsis muscosa* Lamouroux |
| **Bryopsis penicillum* Meneghini |
| *Bryopsis pennata* Lamouroux |
| *Bryopsis plumosa* (Hudson) C. Agardh |
| **Bryopsis secunda* J. Agardh |
| *Pseudobryopsis myura* (J. Agardh) Berthold [=*Trichosolen myurus* (J. Agardh) Taylor] |
|  |
| Family Caulerpaceae |
| *#Caulerpa mexicana* Sonder ex Kützing |
| *Caulerpa prolifera* (Forsskal) Lamouroux (=*Caulerpa ollivieri* Dostál)^2^ |
| *#Caulerpa racemosa* (Forsskal) J. Agardh var. *cylindracea* (Sonder) Verlaque, Huisman & Boudouresque^3,4^ |
| *#Caulerpa racemosa* var. *lamourouxii* (Turner) Weber van Bosse f. *requienii* (Montagne) Weber van Bosse |
| *#Caulerpa racemosa* var. *turbinata* (J. Agardh) Eubank/*uvifera* (C. Agardh) J. Agardh |
| *#Caulerpa scalpelliformis* (Brown ex Turner) C. Agardh |
| *#Caulerpa taxifolia* (Vahl) C. Agardh |
|  |
| Family Chaetosiphonaceae |
| *Chaetosiphon moniliformis* Huber |
|  |
| Family Codiaceae |
| *Codium adhaerens* C. Agardh^5^ |
| *Codium bursa* (Linnaeus) C. Agardh |
| **Codium coralloides* (Kützing) Silva |
| *Codium decorticatum* (Woodward) Howe [=*Codium elongatum* (Turner) C. Agardh] |
| *Codium effusum* (Rafinesque) Delle Chiaje (=*Codium difforme* Kützing) |
| *#Codium fragile* (Suringar) Hariot ssp. *tomentosoides* (van Goor) Silva^6^ |
| *#Codium taylorii* Silva |
| *Codium tomentosum* Stackhouse |
| *Codium vermilara* (Olivi) Delle Chiajei |
|  |
| Family Derbesiaceae |
| *#Derbesia boergesenii* (Iyengar & Ramanathan) Mayhoub |
| **Derbesia corallicola* Funk |
| *#Derbesia rhizophora* Yamada |
| *Derbesia tenuissima* (Moris & De Notaris) P.L. Crouan & H.M. Crouan (=*Halicystis parvula* Schmitz ex Murray) |
| *Pedobesia simplex* (Meneghini ex Kützing) Wynne & Leliaert (=*Pedobesia lamourouxii* (J. Agardh) J. Feldmann, Loreau, Codomier & Couté) |
| *Pedobesia solieri* J. Feldmann ex Abélard & Knoepffler |
|  |
| Family Ostreobiaceae |
| *Ostreobium quekettii* Bornet & Flahault |
|  |
| Family Udoteaceae |
| *Flabellia petiolata* (Turra) Nizamuddin [=*Udotea petiolata* (Turra) Boergesen] |
| *Halimeda tuna* (Ellis & Solander) Lamouroux [=*Halimeda tuna* var. *platydisca* (Decaisne) Barton; =*Halimeda tuna* f. *albertisii* Piccone] |
| *Penicillus capitatus* Lamarck (=*Espera mediterranea* Decaisne) |
| *Pseudochlorodesmis furcellata* (Zanardini) Boergesen |
| *Pseudochlorodesmis furcellata* var. *canariensis* Boergesen |
| **Pseudochlorodesmis tenuis* Ercegovic |
|  |
| *Order Dasycladales* |
|  |
| Family Polyphysaceae |
| *Acetabularia acetabulum* (Linnaeus) Silva (=*Acetabularia mediterranea* Lamouroux) |
| *Acetabularia calyculus* Lamouroux |
| *Parvocaulis parvulus* (Solms-Laubach) Berger, Fettweiss, Gleissberg, Liddle, Richter, Sawitsky & Zuccarello (=*Acetabularia parvula* Solms-Laubach) |
|  |
| Family Dasycladaceae |
| *#Batophora* sp.^7^ |
| *Dasycladus vermicularis* (Scopoli) Kraser |
| *#Neomeris annulata* Dickie |
|  |
| ***Class Chlorophyceae*** |
|  |
| *Order Chlorococcales* |
|  |
| Family Chlorochytriaceae |
| *Chlorochytrium cohnii* Wright |
| *Chlorochytrium lemnae* Cohn (=*Chlorochytrium willei* Printz) |
|  |
| *Order Chaetophorales* |
|  |
| Family Chaetophoraceae |
| **Didymosporangium repens* Lambert |
| *Stromatella monostromatica* (Dangeard) Kornmann & Sahling |
| *Stromatella papillosa* (Dangeard) Kornmann & Sahling |
|  |
| *Order Tetrasporales* |
|  |
| Family Palmellopsidaceae |
| *Palmophyllum crassum* (Naccari) Rabenhorst [=*Palmophyllum crassum* f. *gestroi* (Piccone) Giaccone; =*Palmophyllum crassum* var. *orbiculare* (Bornet) J. Feldmann] |
|  |
| Family Tetrasporaceae |
| *Tetraspora gelatinosa* (Vaucher) Desvaux |
|  |
| *Order Volvocales* |
|  |
| Family Carteriaceae |
| *Carteria feldmannii* Conrad & Kufferath^8^ |
|  |
| Family Chlamydomonadaceae |
| *Brachiomonas submarina* Bohlin (=*Brachiomonas gracilis* Bohlin; =*Brachiomonas westiana* Pascher)^8^ |
| *Oltmannsiella lineata* Zimmermann^8^ |
|  |
| Family Dunaliellaceae |
| *Asteromonas gracilis* Artari^8^ |
| *Dunaliella salina* (Dunal) Teodoresco^8^ |
|  |
| **Class Prasinophyceae** |
|  |
| *Order Chlorodendrales* |
|  |
| Family Chlorodendraceae |
| *Prasinocladus lubricus* Kuckuck f. *subsalsus* (Davis) Zimmermann |
| **Tetraselmis fontiana* (Margalef) R.E. Norris, Hohi & Chihara (=*Platymonas fontiana* Margalef)^8^ |
| *Tetraselmis tetrathele* (West) Bucher (=*Platymonas tetrathele* West)^8^ |
|  |
| *Order Pyramimonadales* |
|  |
| Family Pyramimonadaceae |
| *Halosphaera viridis* Schmitz^8^ |
| *Pyramimonas amylifera* Conrad^8^ |
| *Pyramimonas octociliata* N. Carter^8^ |
|  |
| **Class Trebouxiophyceae** |
|  |
| *Order Prasiolales* |
|  |
| Family Prasiolaceae |
| *Prasiola crispa* (Lightfoot) Kützing |
| *Prasiola stipitata* Suhr ex Jessen |
| *Rosenvingiella polyrhiza* (Rosenvinge) Silva |
|  |
| **Class Ulvophyceae** |
|  |
| *Order Cladophorales* |
|  |
| Family Anadyomenaceae |
| *Anadyomene stellata* (Wulfen) C. Agardh |
| *Microdictyon tenuius* Gray |
|  |
| Family Cladophoraceae |
| *Aegagropila linnaei* Kützing (=*Cladophora aegagropila* (Linnaeus) Trevisan) |
| *Chaetomorpha gracilis* Kützing |
| *Chaetomorpha ligustica* (Kützing) Kützing [=*Rhizoclonium lubricum* Setchell & Gardner; *Rhizoclonium tortuosum* (Dillwyn) Kützing]^9,10^ |
| *Chaetomorpha linum* (O.F. Müller) Kützing [=*Chaetomorpha aerea* (Dillwyn) Kützing; =*Chaetomorpha crassa* (C. Agardh) Kützing] |
| *Chaetomorpha mediterranea* (Kützing) Kützing^9,10^ |
| **Chaetomorpha mediterranea* var. *crispa* (J. Feldmann) Gallardo, Gómez-Garreta, Ribera, Cormaci, Furnari, Giaccone & Boudouresque^9, 10^ |
| *Chaetomorpha pachynema* (Montagne) Kützing |
| *Cladophora albida* (Nees) Kützing |
| *Cladophora battersii* Hoek |
| *Cladophora coelothrix* Kützing |
| *Cladophora dalmatica* Kützing |
| *Cladophora echinus* (Biasoletto) Kützing |
| *Cladophora feredayi* Harvey |
| *Cladophora flexuosa* (O.F. Müller) Kützing |
| *Cladophora fracta* (O.F. Müller ex Vahl) Kützing |
| *Cladophora globulina* (Kützing) Kützing |
| *Cladophora glomerata* (Linnaeus) Kützing |
| *Cladophora glomerata* var. *crassior* (C. Agardh) Hoek |
| *#Cladophora herpestica* (Montagne) Kützing [=*Cladophoropsis zollingerii* (Kützing) Reinbold; =*Cladophoropsis javanica* (Kützing) Silva] |
| *Cladophora hutchinsiae* (Dillwyn) Kützing |
| *Cladophora laetevirens* (Dillwyn) Kützing |
| *Cladophora lehmanniana* (Lindenberg) Kützing |
| *Cladophora liebetruthii* Grunow |
| *Cladophora liniformis* Kützing |
| *Cladophora nigrescens* Zanardini ex Frauenfeld |
| *#Cladophora patentiramea* (Montagne) Kützing |
| *Cladophora pellucida* (Hudson) Kützing |
| *Cladophora prolifera* (Roth) Kützing |
| *Cladophora retroflexa* (Bonnemaison ex P.L. Crouan & H.M. Crouan) Hamel |
| *Cladophora ruchingeri* (C. Agardh) Kützing |
| *Cladophora rupestris* (Linnaeus) Kützing |
| *Cladophora sericea* (Hudson) Kützing |
| *Cladophora socialis* Kützing |
| *Cladophora vadorum* (Areschoug) Kützing |
| *Cladophora vagabunda* (Linnaeus) Hoek |
| *Cladophoropsis membranacea* (Hofman Bang ex C. Agardh) Boergesen (=*Cladophoropsis gerloffii* Nizamuddin; *Cladophoropsis modonensis* auct. non (Kützing) Reinbold] |
| *Rhizoclonium riparium* (Roth) Harvey [=*Rhizoclonium implexum* (Dillwyn) Kützing; =*Rhizoclonium kerneri* Stockmayer; =*Rhizoclonium kochianum* Kützing]^10^ |
|  |
| *Order Oltmannsiellopsidales* |
|  |
| Family Oltmannsiellopsidaceae |
| *Dangemannia microcystis* (Dangeard) T. Friedl & C.J. O'Kelly [=*Planophila microcystis* (Dangeard) Kornmann & Sahling] |
|  |
| *Order Siphonocladales* |
|  |
| Family Siphonocladaceae |
| **Siphonocladus pusillus* (C. Agardh ex Kützing) Hauck |
|  |
| Family Valoniaceae |
| *Valonia aegagropila* C. Agardh |
| *Valonia macrophysa* Kützing |
| *Valonia utricularis* (Roth) C. Agardh |
| *Valonia ventricosa* J. Agardh [*Ventricaria ventricosa* (J. Agardh) Olsen & West] |
|  |
| *Order Ulothricales* |
|  |
| Family Gomontiaceae |
| *Eugomontia sacculata* Kornmann^11^ |
| *Gomontia polyrhiza* (Lagerheim) Bornet & Flahault |
|  |
| Family Ulothricaceae |
| *Spongomorpha aeruginosa* (Linnaeus) Hoek [=*Spongomorpha lanosa* (Roth) Kützing] |
| *Ulothrix flacca* (Dillwyn) Thuret (=*Ulothrix pseudoflacca* Wille)^10^ |
| *Ulothrix implexa* (Kützing) Kützing^10^ |
| *Ulothrix subflaccida* Wille^10^ |
| *Urospora penicilliformis* (Roth) Areschoug (=*Urospora mirabilis* Areschoug) |
|  |
| *Order Ulvales* |
|  |
| Family Bolbocoleonaceae |
| *Bolbocoleon piliferum* Pringsheim |
|  |
| Family Capsosiphonaceae |
| *Capsosiphon fulvescens* (C. Agardh) Setchell & Gardner |
|  |
| Family Gayraliaceae |
| *Gayralia oxysperma* (Kützing) Vinogradova ex Scagel, Gabrielson, Garbary, Golden, Hawkes, Lindstrom, Oliveira & Widdowson |
| *Gayralia oxysperma* f. *wittrockii* (Bornet) Bliding |
|  |
| Family Kornmanniaceae^12^ |
| *Blidingia chadefaudii* (J. Feldmann) Bliding |
| *Blidingia marginata* (J. Agardh) Dangeard |
| *Blidingia minima* (Nägeli ex Kützing) Kylin |
| *Blidingia ramifera* (Bliding) Garbary & Barkhouse (=*Blidingia minima* var. *ramifera* Bliding) |
| *Blidingia subsalsa* (Kjellman) Kornmann & Sahling ex Scagel, Gabrielson, Garbary, Golden, Hawkes, Lindstrom, Oliveira & Widdowson |
| *Pseudendoclonium submarinum* Wille |
|  |
| Family Phaeophilaceae |
| *Phaeophila dendroides* (P.L. Crouan & H.M. Crouan) Batters |
| **Phaeophila hirsuta* (Ercegovic) R. Nielsen |
|  |
| Family Ulvaceae^13^ |
| *Ochlochaete hystrix* Thwaites (=*Ochlochaete ferox* Huber) |
| *Percusaria percusa* (C. Agardh) Rosenvinge |
| *Tellamia contorta* Batters (=*Tellamia intricata* Batters) |
| **Ulva adriatica* (Bliding) Ballesteros (=*Enteromorpha adriatica* Bliding) |
| **Ulva aragoënsis* (Bliding) Ballesteros (=*Enteromorpha aragoënsis* Bliding) |
| *Ulva bifrons* Ardré |
| *Ulva clathrata* (Roth) C. Agardh [=*Enteromorpha clathrata* (Roth) Greville; =*Enteromorpha ramulosa* (Smith) Carmichael]^14^ |
| *Ulva compressa* Linnaeus [=*Enteromorpha compressa* (Linnaeus) Nees; =*Enteromorpha compressa* var. *usneoides* (Bonnemaison ex J. Agardh) Bliding] |
| *Ulva curvata* (Kützing) De Toni |
| *#Ulva fasciata* Delile |
| *Ulva flexuosa* Wulfen [=*Enteromorpha flexuosa* (Wulfen) J. Agardh] |
| *Ulva flexuosa* ssp. *linziformis* (Bliding) Ballesteros |
| *Ulva flexuosa* ssp. *paradoxa* (C. Agardh) Wynne |
| *Ulva flexuosa* ssp. *pilifera* (Kützing) Wynne |
| *Ulva intestinalis* Linnaeus [=*Enteromorpha intestinalis* (Linnaeus) Nees] |
| *Ulva intestinalis* f. *cornucopiae* (Lyngbye) Ballesteros |
| **Ulva jugoslavica* (Bliding) Ballesteros (=*Enteromorpha jugoslavica* Bliding) |
| *Ulva kylinii* (Bliding) Hayden, Blomster, Maggs, Silva, Stanhope & Waaland (=*Enteromorpha kylinii* Bliding) |
| *Ulva lactuca* Linnaeus |
| *Ulva linearis* Dangeard |
| *Ulva linza* Linnaeus [=*Enteromorpha linza* (Linnaeus) J. Agardh; =*Enteromorpha ahlneriana* Bliding] |
| *Ulva multiramosa* (Bliding) Ballesteros (=*Enteromorpha multiramosa* Bliding)^15^ |
| **Ulva neapolitana* Bliding |
| *#Ulva pertusa* Kjellman |
| *Ulva prolifera* O.F. Müller [=*Enteromorpha prolifera* (O.F. Müller) J. Agardh] |
| *Ulva prolifera* ssp. *gullmariensis* (Bliding) Taskin |
| *Ulva pseudolinza* (Koeman & Hoek) Hayden, Blomster, Maggs, Silva, Stanhope & Waaland (=*Enteromorpha pseudolinza* Koeman & Hoek) |
| *Ulva radiata* (J. Agardh) Hayden, Blomster, Maggs, Silva, Stanhope & Waaland (=*Enteromorpha radiata* J. Agardh) |
| *Ulva ralfsii* (Harvey) Le Jolis (=*Enteromorpha ralfsii* Harvey) |
| *Ulva rigida* C. Agardh (=*Ulva scandinavica* Bliding) |
| *Ulva rotundata* Bliding |
| *Ulva simplex* (Vinogradova) Hayden, Blomster, Maggs, Silva, Stanhope & Waaland [=*Enteromorpha simplex* (Vinogradova) Koeman & Hoek] |
| **Ulva stipitata* (Dangeard) Ballesteros var. *linzoides* (Bliding) Ballesteros (=*Enteromorpha stipitata* Dangeard var. *linzoides* Bliding) |
| *Ulva torta* (Mertens) Trevisan [=*Enteromorpha torta* (Mertens) Reinbold] |
| *#Ulvaria obscura* (Kützing) Gayral ex Bliding [=*Monostroma obscurum* (Kützing) J. Agardh] |
| *Umbraulva olivascens* (Dangeard) Furnari |
|  |
| Family Ulvellaceae |
| *Acrochaete flustrae* (Reinke) O'Kelly (=*Epicladia flustrae* Reinke) |
| *Acrochaete geniculata* (N.L. Gardner) O'Kelly |
| *Acrochaete inflata* (Ercegovic) Gallardo, Gómez-Garreta, Ribera, Cormaci, Furnari, Giaccone & Boudouresque (=*Pseudodictyon inflatum* Ercegovic) |
| *Acrochaete leptochaete* (Huber) Nielsen |
| *Acrochaete repens* Pringsheim |
| *Acrochaete viridis* (Reinke) R. Nielsen (=*Entocladia viridis* Reinke) |
| *Acrochaete wittrockii* (Wille) R. Nielsen (=*Entocladia wittrockii* Wille) |
| **Entocladia endolithica* (Ercegovic) R. Nielsen |
| *Entocladia major* (J. Feldmann) R. Nielsen (=*Endoderma majus* J. Feldmann) |
| **Entocladia pennata* (J. Feldmann) R. Nielsen |
| *Epicladia perforans* (Huber) R. Nielsen |
| **Pringsheimiella conchyliophila* J. Feldmann |
| *Pringsheimiella scutata* (Reinke) Marchewianka |
| *Pseudopringsheimia confluens* (Rosenvinge) Wille (=*Ulvella confluens* Rosenvinge) |
| *Ulvella acervus* Dangeard |
| *Ulvella lens* P.L. Crouan & H.M. Crouan |
| *Ulvella setchellii* Dangeard |

**Notes**

1. Genus *Bryopsis* Lamouroux requires nomenclatural and taxonomical re-investigation.

2. We agree with González Henríquez & Santos Guerra (1983) in considering that *Caulerpa prolifera* and *Caulerpa ollivieri* are conspecific.

3. Delimitation of *Caulerpa racemosa* varieties follows Verlaque et al. (2000) amended by Verlaque et al. (2003).

4. *Caulerpa racemosa* var. *cylindracea* was first reported as *Caulerpa racemosa* aff. var. *occidentalis* (J. Agardh) Boergesen (Verlaque et al. 2000).

5. *Codium adhaerens* C. Agardh is present in the Mediterranean (Alboran Sea) (Benhissoune et al. 2001; Ballesteros pers. obs., confirmed by PC Silva).

6. The subspecies of *Codium fragile* that has been introduced in the Mediterranean has been usually identified as ssp. *tomentosoides* (e.g. Gallardo et al. 1993; Boudouresque & Verlaque 2002; Zenetos et al. 2005). However, Verlaque et al. (in press) only report ssp. *fragile*.

7. Reported by Bottalico et al. (2006).

8. Planktonic flagellates, not benthic.

9. We do not follow John et al. (2004) nor Algaebase in considering *Chaetomorpha ligustica* (Kützing) Kützing and *Chaetomorpha mediterranea* (Kützing) Kützing as synonyms.

10. Requires nomenclatural and taxonomical re-investigation.

11. See Verlaque (2001).

12. Genus *Blidingia* requires nomenclatural and taxonomical re-investigation.

13. Taxonomy of Mediterranean species of *Ulva* requires further research.

14. Blomster et al. (1999) consider *Enteromorpha ramulosa* (Smith) Carmichael, *Enteromorpha muscoides* (Clemente) Cremades and *Enteromorpha crinita* Nees to be heterotypic synonyms of *Enteromorpha clathrata* (Roth) Greville. We maintain the synonymy although in our opinion distinction between these entities at a Mediterranean level should be reassessed.

15. John et al. (2004) note that, according to Hayden et al. (2003), the species is invalid because Bliding (1960) did not cite the type.

*Taxa inquirenda* (see also Gallardo et al. 1993)

*Cladophora hutchinsioides* Hoek & Womersley: see Zenetos et al. (2008) regarding the identification of this species.

*Cladophora suhriana* Kützing: see Furnari et al. (2003).

*Enteromorpha prolifera* (O.F. Müller) J. Agardh var. *crispatissima* Schiffner: see Furnari et al. (2003).

*Enteromorpha prolifera* (O.F. Müller) J. Agardh var. *tenuis* Schiffner: see Furnari et al. (2003).

*Flabellia minima* (Ernst) Nizamuddin.

*Ulothrix mucosa* Oltmanns: see Furnari et al. (2003).

*Ulva lactuca* Linnaeus f. *laciniata* (J. Agardh) De Toni: see Furnari et al. (2003).

*Taxa excludenda* (see also Gallardo et al. 1993)

*Caulerpa racemosa* (Forsskal) J. Agardh var. *racemosa*: see Verlaque et al. (2000).

*Caulerpa sertularioides* (Gmelin) Howe: doubtful record; its presence in the Mediterranean should be confirmed (Gallardo et al. 1993).

*Microdictyon laxereticulatum* Setchell: included in the Mediterranean checklist (Gallardo et al. 1993), it is based on a supposed record by Setchell (1929) from Italy. This species is not reported in the Italian checklist by Furnari et al. (2003).

*Sphaeroplea braunii* Kützing: reported by Güven & Öztig (1971), it is a freshwater species.

*Ulva flexuosa* Wulfen ssp. *biflagellata* (Bliding) Ballesteros, comb. nov.: reported as *Enteromorpha flexuosa* ssp. *biflagellata* by Sfriso (1987). The taxonomy of the complex *flexuosa* is so complicated that, in our opinion, the presence of this subspecies in the Mediterranean needs to be confirmed.

*Ulva flexuosa* Wulfen ssp. *paradoxa* (C. Agardh) Wynne var. *profunda* (Bliding) Ballesteros, comb. nov.: reported as *Enteromorpha flexuosa* ssp. *paradoxa* var. *profunda* by Sfriso (1987). The taxonomy of the complex *flexuosa* is so complicated that, in our opinion, the presence of this variety in the Mediterranean needs to be confirmed.

*Ulva hendayensis* (Dangeard & Parriaud) Ballesteros comb. nov.: reported from southern Spain by Conde (1984) as *Enteromorpha hendayensis*. According to Gallardo et al. (1993) the presence of this species in the Mediterranean needs to be confirmed.

*Ulothrix zonata* (Weber & Mohr) Kützing: reported from southern Italy (Giaccone et al. 1985), it is a freshwater species.

**Nomenclatural changes**

According to Hayden *et al*. (2003) the genus *Enteromorpha* should be included in *Ulva*. Therefore the following new combinations are proposed:

*Ulva adriatica* (Bliding) Ballesteros comb. nov. [Basionym: *Enteromorpha adriatica* Bliding (1960, *Botaniska Notiser* 113, p. 174, fig. 3)].

*Ulva aragoënsis* (Bliding) Ballesteros comb. nov. [Basionym: *Enteromorpha aragoënsis* Bliding (1960, *Botaniska Notiser* 113, p. 174, fig. 2a-f)].

*Ulva flexuosa* Wulfen ssp. *biflagellata* (Bliding) Ballesteros comb. nov. [Basionym: *Enteromorpha biflagellata* Bliding (1944, *Botaniska Notiser* 1944, p. 346, figs. 19-23). Homotypic synonym: *Enteromorpha flexuosa* (Wulfen) C. Agardh ssp. *biflagellata* Bliding (1963, *Opera Botanica* 8(3), p. 88)].

*Ulva flexuosa* Wulfen ssp. *paradoxa* (C. Agardh) Wynne var. *profunda* (Bliding) Ballesteros comb. nov. [Basionym: *Enteromorpha flexuosa* (Wulfen) C. Agardh ssp. *paradoxa* (Dillwyn) Bliding var. *profunda* Bliding (1963, *Opera Botanica* 8(3), p. 85, figs. 46-47)].

*Ulva flexuosa* Wulfen ssp. *linziformis* (Bliding) Ballesteros comb. nov. [Basionym: *Enteromorpha linziformis* Bliding (1960, *Botaniska Notiser* 113, p. 181, figs. 8-9). Homotypic synonym: *Enteromorpha flexuosa* (Wulfen) C. Agardh ssp. *linziformis* (Bliding) Bliding (1963, *Opera Botanica* 8(3), p. 87)].

*Ulva hendayensis* (Dangeard & Parriaud) Ballesteros comb. nov. [Basionym: *Enteromorpha hendayensis* Dangeard & Parriaud (1960, Comptes Rendus des Séances de l'Académie des Sciences 250, p. 2972, figs. 1-2)].

*Ulva intestinalis* Linnaeus f. *cornucopiae* (Lyngbye) Ballesteros comb. nov. [Basionym: *Scytosiphon intestinalis* c *cornucopiae* Lyngbye (1819, *Tentamen hydrophytologiae danicae; continens omnia hydrophyta cryptogama Daniae, Holsatiae, Faeroae, Islandiae, Groenlandiae hucusqve cognita, systematice disposita, descripta et iconibus illustrata, adjectis simul speciebus norvegicis,* Hafniae, Copenhagen, p. 67). Homotypic synonym: *Enteromorpha intestinalis* (Linnaeus) Nees f. *cornucopiae* (Lyngbye) J. Agardh].

*Ulva jugoslavica* (Bliding) Ballesteros comb. nov. [Basionym: *Enteromorpha jugoslavica* Bliding (1960, *Botaniska Notiser* 113, p. 172, fig. 1)].

*Ulva multiramosa* (Bliding) Ballesteros comb. nov. [Basionym: *Enteromorpha multiramosa* Bliding (1960, *Botaniska Notiser* 113, p. 177, figs. 4-5)].

*Ulva stipitata* (Dangeard) Ballesteros comb. nov. [Basionym: *Enteromorpha stipitata* P. Dangeard (1959, *Botaniste* 42, p. 42, figs. 16-17)].

*Ulva stipitata* (Dangeard) Ballesteros var. *linzoides* (Bliding) Ballesteros comb. nov. [Basionym: *Enteromorpha stipitata* Dangeard var. *linzoides* Bliding (1960, *Botaniska Notiser* 113, p. 179)].

**References**

Benhissoune S, Boudouresque CF, Verlaque M. 2001. A checklist of marine seaweeds of the Mediterranean and Atlantic coasts of Morocco I. Chlorophyceae Wille s.l. *Botanica Marina* 44: 171-182.

Bliding C. 1944. Zur Systematik der schwedischen Enteromorphen. *Botaniska Notiser* 1944: 331-356.

Bliding C. 1960. A preliminary report on some new Mediterranean green algae. *Botaniska Notiser* 113: 172-184.

Bliding C. 1963. A critical survey of European taxa in Ulvales. Part I. *Capsosiphon*, *Percusaria*, *Blidingia*, *Enteromorpha*. *Opera Botanica* 8(3): 1-160.

Blomster J, Maggs CA, Stanhope MJ. 1999. Extensive intraspecific morphological variation in *Enteromorpha muscoides* (Chlorophyta) revealed by molecular analysis. *Journal of Phycology* 35: 575-586.

Bottalico A, Delle Foglie CI, Perrone C, 2006. New records along the Apulian coasts. In: *Proceedings of the* *Second Mediterranean Symposium on Marine Vegetation* (Athens, 12-13 December 2003): 77-82. RAC-SPA edit., Tunis.

Boudouresque CF, Verlaque M. 2002. Biological pollution in the Mediterranean Sea: invasive versus introduced macrophytes. *Marine Pollution Bulletin* 44: 32-38.

Conde F. 1984. Catálogo de las algas macrobentónicas marinas de Málaga. *Acta Botanica Malacitana* 9: 47-78.

Cormaci M, Furnari G, Alongi G, Serio D, Petrocelli A, Cecere E. 2001. Censimento delle macroalghe marine bentoniche delle coste pugliesi. *Thalassia Salentina* 25: 75-158.

Gallardo T, Gómez-Garreta A, Ribera MA, Cormaci M, Furnari G, Giaccone G, Boudouresque CF. 1993. Checklist of Mediterranean seaweeds II. Chlorophyceae Wille s.l. *Botanica Marina* 36: 399-421.

González Henríquez N, Santos Guerra A. 1983. El género *Caulerpa* Lamouroux en las islas Canarias. *Botanica Macaronesica* 11: 3-24.

Dangeard P. 1959. Observations sur queques Ulvacées du Maroc. *Botaniste* 42: 5-63.

Dangeard P., Parriaud H. 1960. Sur une Entéromorphe nouvelle (*E. hendayensis* nov. sp.) à développement du type *tubulosa*. *Comptes Rendus de l'Académie des Sciences* 250: 2970-2973.

Furnari G, Giaccone G, Cormaci M, Alongi G, Serio D. 2003. Biodiversità marina delle coste italiane: catalogo del macrofitobentos. *Biologia Marina Mediterranea* 10(1): 1-482.

Giaccone G, Colonna P, Graziano C, Mannino AM, Tornatore E, Cormaci M, Furnari G, Scammacca B. 1985. Revisione della flora marina di Sicilia e isole minori. *Bollettino della Accademia Gioenia Scienze Naturale di Catania* 18: 537-781.

Güven KC, Öztig F. 1971. Über die marinen Algen an den Küsten der Türkei. *Botanica Marina* 14: 121-128.

Hayden HS, Blomster J, Maggs CA, Silva PC, Stanhope MJ, Waaland JR. 2003. Linnaeus was right all along: *Ulva* and *Enteromorpha* are not distinct genera. *European Journal of Phycology* 38: 277-294.

John DM, Prud'homme van Reine WF, Lawson GW, Kostermans TB, Price JH. 2004. A taxonomic geographical catalogue of the seaweeds of the western coast of Africa and adjacent islands. *Nova Hedwigia* 127: 1-339.

Lyngbye HC. 1819. *Tentamen hydrophytologiae danicae; continens omnia hydrophyta cryptogama Daniae, Holsatiae, Faeroae, Islandiae, Groenlandiae hucusqve cognita, systematice disposita, descripta et iconibus illustrata, adjectis simul speciebus norvegicis,* Hafniae, Copenhagen, 248 pp.

Rindi F, Sartoni G, Cinelli F. 2002. A floristic account of the benthic marine algae of Tuscany (Western Mediterranean Sea). *Nova Hedwigia* 74: 201-250.

Setchell WA. 1929. The genus *Microdictyon*. *University of California Publications in Botany* 14: 453-588.

Sfriso A. 1987. Flora and vertical distribution of macroalgae in the lagoon of Venice: a comparison with previous studies. *Giornale Botanico Italiano* 121: 69-85.

Verlaque M. 2001. Checklist of the macroalgae of the Thau lagoon (Hérault, France), a hot spot of marine species introduction in Europe. *Oceanologica Acta* 24: 29-49.

Verlaque M, Boudouresque CF, Meinesz A, Gravez V. 2000. The *Caulerpa racemosa* complex (Caulerpales, Ulvophyceae) in the Mediterranean Sea. *Botanica Marina* 43: 49-68.

Verlaque M, Durand C, Huisman JM, Boudouresque CF, Le Parco Y. 2003. On the identity and origin of the Mediterranean invasive *Caulerpa racemosa* (Caulerpales, Chlorophyta). *European Journal of Phycology* 38: 325-339.

Verlaque M, Ruitton S, Mineur F, Boudouresque CF. in press. 4. Macrophytes. In: Briand F (ed), *CIESM Atlas of Exotic species in the Mediterranean*. CIESM Publishers. Monaco.

Zenetos A, Çinar ME, Pancucci-Papadopoulou MA, Harmelin JG, Furnari G, Andaloro F, Bellou N, Streftaris N, Zibrowius H. 2005. Annotated list of marine alien species in the Mediterranean with records of the worst invasive species. *Mediterranean Marine Science* 6: 63-118.

Zenetos A, Meriç E, Verlaque M, Galli P, Boudouresque CF, Giangrande A, Çinar ME, Bilecenoglu M. 2008. Additions to the annotated list of marine alien biota in the Mediterranean with special emphasis on Foraminifera and Parasites. *Mediterranean Marine Science* 9: 119-165.

## **Table S12. Checklist of the phylum Magnoliophyta and comments to the checklist**

Flowering plants (phylum Magnoliophyta, Kingdom Plantae) have been classified by Classes, Orders, Families and Species. Ordination is alphabetical.

This list is based in the previous knowledge on seagrass distribution (e.g. Phillips & Meñez 1988; Green & Short 2003). Two other species thriving in brackish waters that can be occasionally found in extremely sheltered and shallow bays and lagoons have also been listed. Black Sea is not included.

Taxonomy follows *Flora Europaea* (Tutin et al. 1964). Endemic species (found only in the Mediterranean basin) are preceded by the symbol *. Introduced species are preceded by the symbol #. Some of the commonest synonyms found in Mediterranean literature (if any) are sometimes placed after the currently accepted name.

| **Phylum Magnoliophyta** |
| --- |
|  |
| **Class Angiospermae** |
|  |
| *Order Potamogetonales* |
|  |
| Family Cymodoceae |
| *Cymodocea nodosa* (Ucria) Ascherson |
|  |
| Family Posidoniaceae |
| **Posidonia oceanica* (Linnaeus) Delile |
|  |
| Family Ruppiaceae |
| *Ruppia cirrhosa* (Petagna) Grande |
| *Ruppia maritima* Linnaeus |
|  |
| Family Zosteraceae |
| *Zostera marina* Linnaeus |
| *Zostera noltii* Hornemann [=*Nanozostera noltii* (Hornemann) Tomlinson & Posluzny] |
|  |
| *Order Hydrocharitales* |
|  |
| Family Hydrocharitaceae |
| *#Halophila stipulacea* (Forsskal) Ascherson |

**References**

Green EP, Short FT. 2003. *World atlas of seagrasses*. University of California, Berkeley, Los Angeles & London. 298 pp.

Phillips RC, Meñez EG. 1998. *Seagrasses*. *Smithsonian Contributions to the Marine Sciences* 34: 1-104.

Tutin TG, Heywood VH, Burges NA, Valentine DH, Walters SM, Webb DA (eds). 1964. *Flora Europaea, volume 5*. Cambridge University, Cambridge, 452 pp.

## **Sponges (by Eleni Voultsiadou & Thanos Dailianis)**

## **Figure S1. Mediterranean percentages of the world sponge families and genera for each demosponge order**


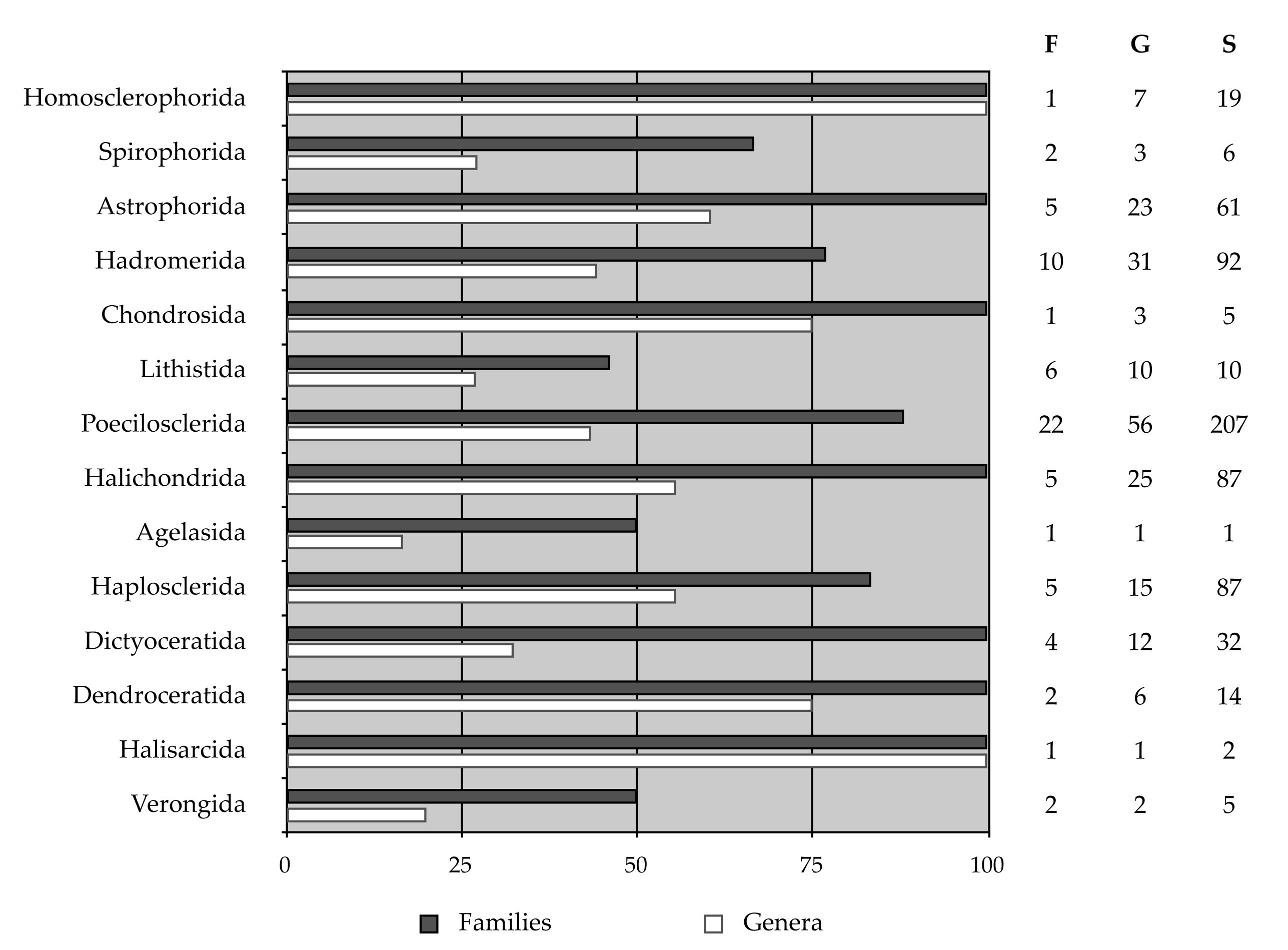
The total numbers of Mediterranean families (F), genera (G) and species (S) are given.

## **Figure S2. Distribution of the recorded demosponge species (outer circle) and genera (inner circle) richness in distinct zoogeographic areas of the Mediterranean**


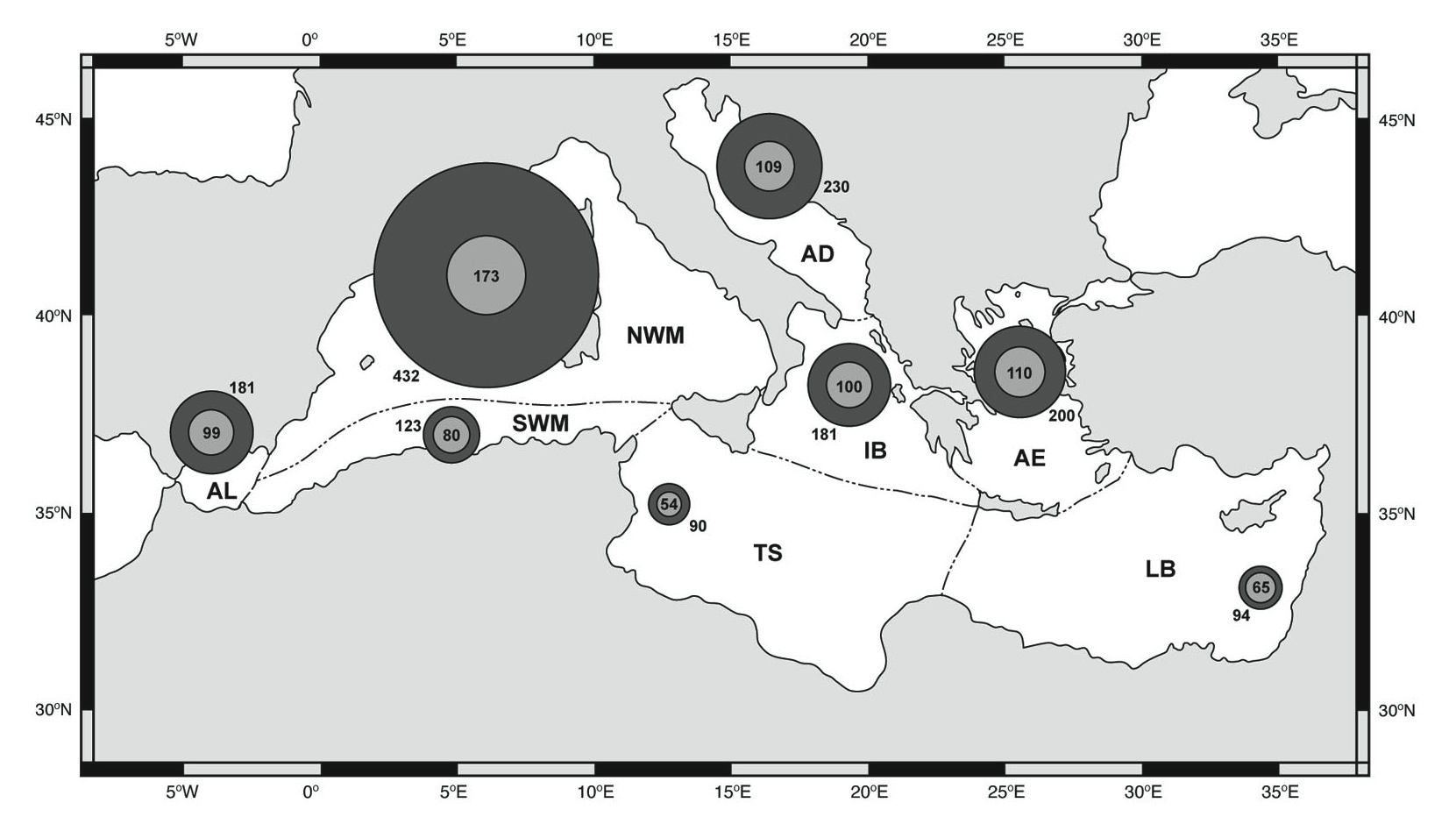
Numbers of species for each area are given outside the outer circle and number of genera inside the inner circle. AD: Adriatic Sea, AE: Aegean Sea, AL: Alboran Sea, IB: Ionian Basin, LB: Levantine Basin, NWM: north-western Mediterranean, SWM: south-western Mediterranean, TS: Tunisian Plateau/Gulf of Sidra [for the division of the Mediterranean we consulted 1,2,3].

**References**

1. Por FD, Dimentman C (2006) Mare Nostrum: Neogene and anthropic natural history of the Mediterranean basin, with emphasis on the Levant. Bulgaria: Pensof Publishers.

2. Spalding MD, Fox HE, Halpern BS, McManus MA, Molnar J, et al. (2007) Marine ecoregions of the world: A bioregionalization of coastal and shelf areas. Bioscience 57: 573-583.

3. Voultsiadou E (2009) Reevaluating sponge diversity and distribution in the Mediterranean Sea. Hydrobiologia 628: 1-12.

## **Anthozoans (by Dimitris Vafidis)**

## **Table S13. Checklist of the Class Anthozoa (Phylum Cnidaria)**

WB: Western basin; AD: Adriatic Sea; IB: Ionian basin; AS: Aegean Sea; LB: Levantine basin; BS: Black Sea; DZ: Depth zone; I: Infralittoral zone; C: Circalittoral zone; B: Bathyal zone; ZC: Zoogeographical characterization; AM: Atlanto-Mediterranean; E: Endemic; C: Cosmopolitan; AA: Amphi-Atlantic; B: Boreal; IP: Indo-Pacific.

| **Mediterranean species** | **WB** | **AD** | **IB** | **AS** | **LB** | **DZ** | **ZC** |
| --- | --- | --- | --- | --- | --- | --- | --- |
|  |  |  |  |  |  |  |  |
|  |  |  |  |  |  |  |  |
| **Order Stolonifera** |  |  |  |  |  |  |  |
| Family Cornulariidae |  |  |  |  |  |  |  |
| *Cornularia cornucopiae* (Pallas, 1766) | **x** | **x** |  | **x** |  | **I** | **AM** |
| *Cervera atlantica* (Johnson, 1861) | **x** |  |  |  |  | **I** | **AM** |
| Family Clavulariidae |  |  |  |  |  |  |  |
| *Clavularia crassa* (Milne Edwards, 1848) | **x** | **x** |  | **x** |  | **I** | **E** |
| *Clavularia marioni* Koch, 1891 | **x** |  |  |  |  | **C** | **AM** |
| *Clavularia carpediem* Weinberg, 1986 | **x** |  |  |  |  | **I** | **E** |
| *Sarcodictyon catenatum* Forbes, 1847 | **x** |  |  |  |  | **IC** | **AM** |
| *Rolandia coralloides* De Lacaze-Duthiers, 1900 | **x** | **x** |  | **x** |  | **IC** | **AM** |
| *Scleranthelia rugosa* (Pourtales, 1867) | **x** |  |  |  |  | **CB** | **AA** |
| *Scleranthelia microsclera* Lopez-Gonzales, Ocana & Garcia-Gomez, 1995 | **x** |  |  |  |  | **I** | **AM** |
| *Telestula septentrionalis* Madsen, 1944 | **x** |  |  |  |  | **B** | **AM** |
|  |  |  |  |  |  |  |  |
| **Order Alcyonacea** |  |  |  |  |  |  |  |
| Family Alcyoniidae |  |  |  |  |  |  |  |
| *Alcyonium palmatum* Pallas, 1766 | **x** | **x** | **x** | **x** |  | **IC** | **AM** |
| *Alcyonium acaule* Marion, 1878 | **x** | **x** |  | **x** |  | **IC** | **AM** |
| *Alcyonium coralloides* (Pallas, 1766) | **x** | **x** |  | **x** |  | **IC** | **AM** |
| Family Maasellidae |  |  |  |  |  |  |  |
| *Maasella edwardsi* (Lacaze-Duthiers, 1888) | **x** | **x** | **x** |  |  | **IC** | **E** |
| *Paralcyonium spinulosum* (Delle Chiaje, 1822) | **x** |  |  | **x** |  | **IC** | **AM** |
|  |  |  |  |  |  |  |  |
| **Order Gorgonacea** |  |  |  |  |  |  |  |
| Family Coralliidae |  |  |  |  |  |  |  |
| *Corallium rubrum* (Linnaeus, 1758) | **x** | **x** | **x** | **x** | **x** | **ICB** | **AM** |
| Family Melithaeidae |  |  |  |  |  |  |  |
| *Acabaria erythraea* (Ehrenberg,1834) |  |  |  |  | **x** | **I** | **IP** |
| Family Acanthogorgiidae |  |  |  |  |  |  |  |
| *Acanthogorgia hirsuta* Gray, 1857 | **x** |  | **x** |  |  | **B** | **AM** |
| Family Plexauridae |  |  |  |  |  |  |  |
| *Paramuricea clavata* (Risso, 1826) | **x** | **x** |  | **x** |  | **C** | **E** |
| *Paramuricea macrospina* (Koch, 1882) | **x** | **x** | **x** | **x** |  | **C** | **E** |
| *Bebryce mollis* Philippi, 1842 | **x** |  | **x** |  |  | **B** | **AM** |
| *Spinimuricea atlantica* (Johnson, 1862) | **x** |  |  |  |  | **C** | **AM** |
| *Spinimuricea klavereni* Carpine & Grasshoff, 1975 | **x** |  |  | **x** |  | **C** | **E** |
| *Muriceides lepida* Carpine & Grasshoff, 1975 | **x** |  |  |  |  | **CB** | **AM** |
| *Placogorgia coronata* Carpine & Grasshoff, 1975 | **x** |  |  |  |  | **B** | **AM** |
| *Placogorgia massiliensis* Carpine & Grasshoff, 1975 | **x** |  |  |  |  | **B** | **E** |
| *Swiftia pallida* Madsen, 1970 | **x** |  |  | **x** |  | **B** | **AM** |
| *Villogorgia bebrycoides* (Koch, 1887) | **x** |  |  | **x** |  | **CB** | **AM** |
| Family Gorgoniidae |  |  |  |  |  |  |  |
| *Eunicella singularis* (Esper, 1791) | **x** | **x** | **x** | **x** |  | **ICB** | **E** |
| *Eunicella cavolini* (Koch, 1887) | **x** | **x** | **x** | **x** |  | **ICB** | **E** |
| *Eunicella verrucosa* (Pallas, 1766) | **x** |  | **x** | **x** |  | **ICB** | **AM** |
| *Eunicella filiformis* Studer, 1901 | **x** |  |  |  |  | **CB** | **AM** |
| *Eunicella gazella* Studer, 1901 | **x** |  |  |  |  | **I** | **AM** |
| *Eunicella labiata* Thomson, 1927 | **x** |  |  |  |  | **I** | **AM** |
| *Leptogorgia sarmentosa* Esper, 1789 | **x** | **x** |  | **x** |  | **ICB** | **AM** |
| *Leptogorgia viminalis* (Pallas, 1766) | **x** |  |  |  |  | **C** | **AM** |
| *Leptogorgia guineensis* Grasshoff, 1988 | **x** |  |  |  |  | **B** | **AM** |
| Family Ellisellidae |  |  |  |  |  |  |  |
| *Ellisella paraplexauroides* Stiasny, 1936 | **x** |  |  |  |  | **CB** | **AM** |
| *Viminella flagellum* (Johnson, 1863) | **x** |  |  |  |  | **C** | **AM** |
| Family Primnoidae |  |  |  |  |  |  |  |
| *Callogorgia verticillata* (Pallas, 1766) | **x** |  | **x** | **x** |  | **CB** | **AM** |
| Family Isididae |  |  |  |  |  |  |  |
| *Isidella elongata* (Esper, 1788) | **x** | **x** |  | **x** |  | **B** | **AM** |
|  |  |  |  |  |  |  |  |
| **Order Pennatulacea** |  |  |  |  |  |  |  |
| Family Veretillidae |  |  |  |  |  |  |  |
| *Veretillum cynomorium* (Pallas, 1766) | **x** | **x** | **x** | **x** |  | **CB** | **C** |
| *Cavernularia pusilla* (Philippi, 1835) | **x** |  |  | **x** |  | **CB** | **AM** |
| Family Kophobelemnidae |  |  |  |  |  |  |  |
| *Kophobelemnon stelliferum* (O.F. Muller, 1776) | **x** |  | **x** | **x** |  | **CB** | **C** |
| Family Funiculinidae |  |  |  |  |  |  |  |
| *Funiculina quadrangularis* (Pallas, 1766) | **x** | **x** | **x** | **x** | **x** | **CB** | **C** |
| Family Penatulidae |  |  |  |  |  |  |  |
| *Virgularia mirabilis* (Linnaeus, 1758) | **x** | **x** |  | **x** |  | **CB** | **C** |
| Family Penatulidae |  |  |  |  |  |  |  |
| *Pennatula aculeata* Danielsen, 1860 | **x** |  |  |  |  | **B** | **B** |
| *Pennatula phosphorea* Linnaeus, 1758 | **x** | **x** | **x** | **x** |  | **CB** | **C** |
| *Pennatula rubra* Pallas, 1766 | **x** | **x** |  | **x** |  | **C** | **AM** |
| *Pteroeides griseum* (Linnaeus, 1767) | **x** | **x** | **x** | **x** |  | **CB** | **AM** |
| *Crassophyllum thessalonicae* Vafidis & Koukouras, 1991 |  |  |  | **x** |  | **C** | **E** |
|  |  |  |  |  |  |  |  |
| **Order Antipatharia** |  |  |  |  |  |  |  |
| Family Antipathidae |  |  |  |  |  |  |  |
| *Antipathes dichotoma* Pallas, 1766 | **x** |  |  | **x** |  | **CB** | **C** |
| *Antipathes fragilis* Gravier, 1918 | **x** |  |  |  |  | **C** | **AM** |
| *Parantipathes larix* (Esper, 1790) | **x** |  |  | **x** |  | **B** | **AM** |
| Family Myriopathidae |  |  |  |  |  |  |  |
| *Antipathella subpinnata* (Ellis & Solander, 1786) | **x** | **x** |  | **x** |  | **CB** | **AM** |
| Family Leiopathidae |  |  |  |  |  |  |  |
| *Leiopathes glaberrima* (Esper, 1792) | **x** | **x** | **x** | **x** |  | **B** | **AA** |
|  |  |  |  |  |  |  |  |
| **Order Ceriantharia** |  |  |  |  |  |  |  |
| Family Cerianthidae |  |  |  |  |  |  |  |
| *Cerianthus membranaceus* (Spallanzani, 1784) | **x** | **x** |  | **x** | **x** | **IC** | **AM** |
| *Cerianthus lloydii* (Gosse, 1859) | **x** | **x** |  |  |  | **I** | **B** |
| *Pachycerianthus solitarius* (Rapp, 1829) | **x** | **x** |  | **x** | **x** | **IC** | **AM** |
| *Pachycerianthus dohrni* (van Beneden, 1923) | **x** |  |  |  |  | **IC** | **E** |
| Family Botrucnidiferiidae |  |  |  |  |  |  |  |
| *Cerianthula mediterranea* van Beneden, 1897 | **x** | **x** |  |  |  | **IC** | **AM** |
| Family Arachnactidae |  |  |  |  |  |  |  |
| *Arachnactis albida* Sarsi, 1846 | **x** |  |  |  |  | **I** | **AM** |
| *Arachnanthus oligopodus* (Cerfontaine, 1891) | **x** |  |  | **x** | **x** | **I** | **E** |
|  |  |  |  |  |  |  |  |
| **Order Actiniaria** |  |  |  |  |  |  |  |
| Family Gonactiniidae |  |  |  |  |  |  |  |
| *Gonactinia prolifera* (Sars, 1835) |  | **x** |  |  |  | **IC** | **AA** |
| *Protanthea simplex* (Carlgren, 1891) | **x** |  |  |  |  | **ICB** | **AM** |
| Family Edwardsiidae |  |  |  |  |  |  |  |
| *Edwardsia claparedii* (Panceri, 1869) | **x** | **x** |  | **x** |  | **IC** | **AM** |
| *Scolanthus callimorphus* (Gosse, 1853) | **x** |  |  | **x** |  | **I** | **AM** |
| Family Halcampoididae |  |  |  |  |  |  |  |
| *Halcampoides purpurea* (Studer, 1878) | **x** | **x** |  |  |  | **IC** | **B** |
| Family Haloclavidae |  |  |  |  |  |  |  |
| *Anemonactis mazeli* (Jourdan,1880) | **x** | **x** |  |  |  | **IC** | **AM** |
| *Mesacmaea mitchellii*  (Gosse, 1853) | **x** | **x** |  | **x** |  | **I** | **AM** |
| *Peachia cylindrica* (Reid, 1848) | **x** | **x** |  | **x** |  | **IC** | **AM** |
| Family Andresiidae |  |  |  |  |  |  |  |
| *Andresia partenopea* (Andres, 1884) | **x** | **x** |  | **x** |  | **I** | **AM** |
| Family Boloceroididae |  |  |  |  |  |  |  |
| *Bunodeopsis strumosa* Andres, 1881 | **x** | **x** | **x** | **x** |  | **I** | **E** |
| Family Aliciidae |  |  |  |  |  |  |  |
| *Alicia mirabilis* Johnson, 1861 | **x** | **x** |  | **x** | **x** | **C** | **AM** |
| Family Condylanthidae |  |  |  |  |  |  |  |
| *Segonzactis hartogi* Vafidis & Chintiroglou, 2002 |  |  |  | **x** |  | **CB** | **E** |
| Family Actiniidae |  |  |  |  |  |  |  |
| *Actinia cari*  Delle Chiaje, 1825 | **x** | **x** | **x** | **x** |  | **I** | **E** |
| *Actinia equina mediterranea* Schmidt, 1971 | **x** | **x** | **x** | **x** | **x** | **I** | **C** |
| *Actinia striata* (Rizzi, 1907) | **x** | **x** |  | **x** |  | **IC** | **AM** |
| *Paranemonia vouliagmeniensis* Doumenc, England & Chintiroglou, 1987 |  |  |  | **x** |  | **I** | **E** |
| *Anemonia melanaster* (Verrill, 1901) |  |  |  |  | **x** | **I** | **AM** |
| *Anemonia viridis* (Forskal, 1775) | **x** | **x** | **x** | **x** | **x** | **I** | **AM** |
| *Anthopleura ballii* (Cocks, 1850) | **x** | **x** |  |  | **x** | **IC** | **AM** |
| *Anthopleura thallia* (Gosse,1854) | **x** |  |  |  |  | **I** | **AM** |
| *Paranemonia cinerea* (Contarini, 1844) | **x** | **x** |  | **x** |  | **I** | **E** |
| *Condylactis aurantiaca* (Delle Chiaje, 1825) | **x** | **x** | **x** | **x** | **x** | **I** | **E** |
| *Cribrinopsis crassa* (Andres,1884) | **x** | **x** |  |  |  | **C** | **E** |
| *Bunodactis rubripunctata* (Grube, 1840) | **x** | **x** |  |  | **x** | **I** | **AM** |
| *Bunodactis verrucosa* (Pennant, 1777) | **x** | **x** |  | **x** |  | **I** | **AM** |
| Family Aurelianidae |  |  |  |  |  |  |  |
| *Aureliana heterocera* (Thompson, 1853) | **x** | **x** |  |  |  | **C** | **AM** |
| Family Phymanthidae |  |  |  |  |  |  |  |
| *Phymanthus pulcher* (Andres, 1883) | **x** | **x** |  | **x** |  | **IC** | **E** |
| Family Actinostolidae |  |  |  |  |  |  |  |
| *Paranthus rugosus* (Andres,1881) | **x** | **x** |  |  |  | **I** | **E** |
| Family Isophellidae |  |  |  |  |  |  |  |
| *Telmatactis cricoides* (Duchassaing, 1850) |  |  | **x** | **x** | **x** | **I** | **AM** |
| *Telmatactis forskalii* (Ehrenberg, 1834) | **x** | **x** | **x** | **x** | **x** | **I** | **AM** |
| *Telmatactis solidago* (Duchassaing & Michelotti, 1864) |  |  |  | **x** |  | **I** | **AM** |
| Family Hormathiidae |  |  |  |  |  |  |  |
| *Hormathia alba* (Andres,1881) | **x** |  | **x** |  |  | **I** | **AM** |
| *Hormathia coronata* (Gosse, 1858) | **x** | **x** | **x** | **x** |  | **CB** | **AM** |
| *Paractinia striata* (Risso,1826) | **x** |  |  |  |  | **I** | **E** |
| *Actinauge richardi* (Marion, 1882) | **x** | **x** | **x** | **x** |  | **CB** | **AM** |
| *Paracalliactis robusta* Tur, 1991 | **x** |  |  |  |  | **I** | **E** |
| *Calliactis parasitica* (Couch, 1842) | **x** | **x** | **x** | **x** | **x** | **ICB** | **AM** |
| *Adamsia palliata* (O.F. Muller, 1776) | **x** | **x** | **x** | **x** | **x** | **ICB** | **AM** |
| *Amphianthus dohrnii* (Koch, 1878) | **x** | **x** | **x** | **x** |  | **CB** | **AM** |
| Family Sagartiidae |  |  |  |  |  |  |  |
| *Sagartia elegans* (Dalyell, 1848) | **x** | **x** |  | **x** |  | **IC** | **AM** |
| *Sagartia troglodytes* (Price, 1847) | **x** | **x** |  |  |  | **I** | **AM** |
| *Cereus pedunculatus* (Pennant, 1777) | **x** | **x** | **x** | **x** | **x** | **I** | **AM** |
| *Actinothoe sphyrodeta* (Gosse, 1860) | **x** |  |  |  |  | **I** | **AM** |
| *Sagartiogeton entellae* Schmidt, 1972 |  | **x** |  |  |  | **C** | **E** |
| *Sagartiogeton undatus* (O.F. Muller, 1788) | **x** | **x** | **x** | **x** |  | **ICB** | **AM** |
| *Kadophellia bathyalis* Tur, 1991 | **x** |  |  |  |  | **B** | **E** |
| Family Aiptasiidae |  |  |  |  |  |  |  |
| *Aiptasia diaphana* (Rapp, 1829) | **x** | **x** | **x** | **x** | **x** | **I** | **AM** |
| *Aiptasia mutabilis* (Gravenhorst, 1831) | **x** | **x** | **x** | **x** | **x** | **I** | **AM** |
| *Aiptasiogeton pellucidus* (Hollard, 1848) | **x** | **x** |  | **x** | **x** | **I** | **AM** |
| Family Diadumenidae |  |  |  |  |  |  |  |
| *Diadumene cincta* Stephenson, 1925 |  | **x** |  |  |  | **I** | **AM** |
| *Diadumene luciae* (Verrill, 1898) | **x** | **x** |  |  | **x** | **I** | **C** |
|  |  |  |  |  |  |  |  |
| **Order Corralimorpharia** |  |  |  |  |  |  |  |
| Family Diadumenidae |  |  |  |  |  |  |  |
| *Corynactis viridis* Allman, 1846 | **x** | **x** |  | **x** |  | **IC** | **AM** |
| Family Corralimorphidae |  |  |  |  |  |  |  |
| *Sideractis glacialis*Danielssen, 1890 | **x** |  |  |  |  | **B** | **AM** |
|  |  |  |  |  |  |  |  |
| **Order Scleractinia** |  |  |  |  |  |  |  |
| Family Pocilloporidae |  |  |  |  |  |  |  |
| *Madracis pharensis* (Heller, 1868) | **x** | **x** | **x** | **x** | **x** | **IC** | **AM** |
| Family Faviidae |  |  |  |  |  |  |  |
| *Cladocora caespitosa* (Linnaeus, 1767) | **x** | **x** | **x** | **x** | **x** | **IC** | **AM** |
| *Cladocora debilis* Milne Edwards & Haime, 1849 | **x** | **x** | **x** |  |  | **C** | **AA** |
| Family Oculinidae |  |  |  |  |  |  |  |
| *Oculina patagonica* De Angelis, 1908 | **x** |  |  |  | **x** | **I** | **AA** |
| *Madrepora oculata* Linnaeus, 1758 | **x** | **x** | **x** | **x** | **x** | **B** | **C** |
| Family Caryophylliidae |  |  |  |  |  |  |  |
| *Caryophyllia cyathus* (Ellis & Solander, 1786) | **x** | **x** |  |  |  | **CB** | **AM** |
| *Caryophyllia smithii* Stokes & Broderip, 1828 | **x** | **x** | **x** | **x** |  | **ICB** | **AM** |
| *Caryophyllia inornata* (Duncan, 1878) | **x** | **x** | **x** | **x** | **x** | **IC** | **AA** |
| *Caryophyllia calveri* Duncan, 1873 | **x** | **x** |  | **x** | **x** | **CB** | **AM** |
| *Ceratotrochus magnaghii* Cecchini, 1914 | **x** | **x** | **x** |  |  | **ICB** | **AM** |
| *Coenocyathus cylindricus* Milne Edwards & Haime, 1848 | **x** | **x** |  |  |  | **CB** | **AM** |
| *Coenocyathus anthophyllites* Milne Edwards & Haime, 1848 | **x** | **x** |  |  |  | **CB** | **AM** |
| *Paracyathus pulchellus* (Philippi, 1842) | **x** | **x** | **x** | **x** | **x** | **CB** | **AA** |
| *Polycyathus muellerae*  (Abel, 1959) | **x** | **x** | **x** | **x** | **x** | **IC** | **AM** |
| *Sphenotrochus andrewianus* Milne Edwards & Haime, 1848 | **x** | **x** |  |  |  | **IC** | **AM** |
| *Desmophyllum cristagalli* Milne Edwards & Haime, 1848 | **x** |  | **x** | **x** | **x** | **CB** | **C** |
| *Thalamophyllia gasti* (Doderlein, 1913) | **x** | **x** | **x** |  |  | **ICB** | **AM** |
| *Hoplangia durotrix*  Gosse, 1860 | **x** | **x** | **x** | **x** | **x** | **IC** | **AM** |
| *Lophelia pertusa* (Linnaeus, 1758) | **x** | **x** | **x** | **x** | **x** | **B** | **C** |
| *Pourtalosmilia anthophyllites* (Ellis & Solander, 1786) | **x** |  |  |  |  | **B** | **AM** |
| *Phyllangia mouchezii* (Lacaze-Duthiers, 1897) | **x** | **x** | **x** | **x** | **x** | **IC** | **AM** |
| Family Flabellidae |  |  |  |  |  |  |  |
| *Monomyces pygmaea* (Risso, 1826) | **x** | **x** | **x** | **x** |  | **IC** | **AM** |
| *Javania cailleti* (Duchassaing & Michelotti, 1864) | **x** |  |  |  |  | **B** | **C** |
| Family Guyniidae |  |  |  |  |  |  |  |
| *Guynia annulata* Duncan, 1872 | **x** | **x** | **x** | **x** |  | **CB** | **C** |
| *Stenocyathus vermiformis* (Pourtales, 1868) | **x** |  | **x** | **x** | **x** | **B** | **C** |
| Family Dendrophylliidae |  |  |  |  |  |  |  |
| *Dendrophyllia ramea* (Linnaeus, 1758) | **x** | **x** | **x** |  |  | **C** | **AM** |
| *Dendrophyllia cornigera* (Lamarck, 1816) | **x** |  | **x** | **x** | **x** | **CB** | **AM** |
| *Cladopsammia rolandi* Lacaze-Duthiers, 1897 | **x** |  | **x** | **x** |  | **IC** | **E** |
| *Balanophyllia europaea* (Risso, 1826) | **x** | **x** | **x** | **x** | **x** | **IC** | **AM** |
| *Balanophyllia regia* Gosse, 1860 | **x** | **x** |  | **x** |  | **I** | **AM** |
| *Balanophyllia cellulosa* Duncan, 1873 | **x** |  |  |  |  | **CB** | **AM** |
| *Leptopsammia pruvoti* Lacaze-Duthiers, 1897 | **x** | **x** | **x** | **x** | **x** | **IC** | **AM** |
| *Astroides calycularis* (Pallas, 1766) | **x** | **x** | **x** |  |  | **C** | **AM** |
|  |  |  |  |  |  |  |  |
| **Order Zoantharia** |  |  |  |  |  |  |  |
| Family Parazoanthidae |  |  |  |  |  |  |  |
| *Parazoanthus axinellae* (O. Schmidt, 1862) | **x** | **x** | **x** | **x** |  | **ICB** | **AM** |
| *Parazoanthus axinellae adriaticus* Pax, 1937 |  | **x** |  |  |  | **ICB** | **E** |
| *Parazoanthus axinellae liguricus* Pax, 1937 | **x** |  |  |  |  | **CB** | **E** |
| *Parazoanthus axinellae muelleri* Pax, 1957 | **x** |  |  |  |  | **IC** | **E** |
| *Parazoanthus axinellae brevitentacularis* Abel, 1959 | **x** | **x** |  |  |  | **I** | **E** |
| *Savalia savaglia* (Bertoloni, 1819) | **x** | **x** |  | **x** |  | **C** | **AM** |
| Family Epizoanthidae |  |  |  |  |  |  |  |
| *Epizoanthus arenaceus* (Delle Chiaje, 1823) | **x** | **x** | **x** | **x** |  | **ICB** | **AM** |
| *Epizoanthus arenaceus ingeborgae* Pax, 1952 | **x** | **x** |  |  |  | **ICB** | **E** |
| *Epizoanthus incrustatus* (Duben & Koren, 1847) | **x** |  |  |  |  | **CB** | **AA** |
| *Epizoanthus paguricola* (Roule, 1900) | **x** |  |  |  |  | **CB** | **E** |
| *Epizoanthus paxii* Abel,1955 | **x** | **x** |  |  |  | **I** | **E** |
| *Epizoanthus mediterraneus* Carlgren, 1935 | **x** | **x** |  |  |  | **CB** | **E** |
| *Epizoanthus vagus* Herberts, 1972 | **x** |  |  |  |  | **C** | **E** |
| *Epizoanthus univitatus* (Lorenz, 1860) |  | **x** |  |  |  | **C** | **E** |
| *Epizoanthus vatovai* Pax & Louchter, 1935 |  | **x** |  |  |  | **IC** | **E** |

## **Mollusks (by José Templado & Roger Villanueva)**

## **Table S14. Checklist of the Phylum Mollusca**

Information based on Sabelli et al. [1] and Bello [2] and updated from CLEMAM database, Check List of the European Marine Mollusca: http://www.somali.asso.fr/clemam/index

**E = exotic species (species introduced by humans)**

**P = species included in the Annex II of the Barcelona Agreement (list of endangered or threatened species)**

**Pl = planktoninc species**

**CAUDOFOVEATA**

**Chaetodermatidae**

*Chaetoderma strigisquamatum* Salvini-Plawen, 1977

*Falcidens aequabilis* Salvini-Plawen, 1972

*Falcidens gutturosus* (Kowalewsky, 1901)

**Limifossoridae**

*Psilodens elongatus* (Salvini-Plawen, 1972)

*Scutopus robustus* Salvini-Plawen, 1970

*Scutopus ventrolineatus* Salvini-Plawen, 1968

**Prochaetodermatidae**

*Prochaetoderma boucheti* Scheltema & Ivanov, 2000

*Prochaetoderma breve* Salvini-Plawen, 1999

*Prochaetoderma raduliferum* (Kowalewsky, 1901)

**SOLENOGASTRES**

PHOLIDOSKEPIA

**Dondersiidae**

*Dondersia festiva* Hubrecht, 1888

*Ichthyomenia ichthyoides* (Pruvot, 1890)

*Micromenia subrubra* Salvini-Plawen, 2003

*Nematomenia banyulensis* (Pruvot, 1890)

*Nematomenia corallophila* (Kowalewsky, 1881)

*Nematomenia flavens* (Pruvot, 1890)

*Stylomenia salvatori* Pruvot, 1899

**Lepidomeniidae**

*Lepidomenia hystrix* Marion & Kowalewsky in Fischer, 1885

*Lepidomenia swedmarki* Salvini-Plawen, 1985

*Tegulaherpia myodoryata* Salvini-Plawen, 1988

*Tegulaherpia stimulosa* Salvini-Plawen, 1983

**Macellomeniidae**

*Macellomenia* *aciculata* Scheltema, 1999

**Neomeniidae**

*Neomenia carinata* Tullberg, 1875

CAVIBELONIA

**Amphimeniidae**

*Amphimenia neapolitana* Thiele, 1889

*Paragymnomenia richardi* Leloup, 1947

**Pararrhopaliidae**

*Pararrhopalia pruvoti* Simroth, 1893

*Eleutheromenia carinata* Salvini-Plawen & Öztürk, 2006

*Eleutheromenia sierra* (Pruvot, 1890)

*Hypomenia nierstraszi* Lummel, 1930

*Pruvotina impexa* (Pruvot, 1890)

**Simrothiellidae**

*Kruppomenia minima* Nierstrasz in Lo Bianco, 1903

*Uncimenia neapolitana* Nierstrasz, 1903

**Pronomeniidae**

*Proneomenia desiderata* (Kowalewsky & Marion, 1887)

*Dorymenia vegans* (Kowalewsky & Marion, 1887)

**Rhopalomeniidae**

*Rhopalomenia aglaopheniae* (Kowalewsky & Marion, 1887)

*Pruvotia sopita* (Pruvot, 1891)

*Urgorria monoplicata* Salvini-Plawen, 2003

**Strophomeniidae**

*Strophomenia lacazei* Pruvot, 1899

*Anamenia gorgonophila* (Kowalewsky, 1880)

**MONOPLACOPHORA**

**Neopilinidae**

*Veleropilina zografi* (Dautzenberg & Fischer H., 1896)

**POLYPLACOPHORA**

LEPIDOPLEURIDA

**Leptochitonidae**

*Lepidopleurus cajetanus* (Poli, 1791)

*Leptochiton africanus* (Nierstrasz, 1906)

*Leptochiton algesirensis* (Capellini, 1859)

*Leptochiton asellus* (Gmelin, 1791)

*Leptochiton boettgeri* Sulc, 1934

*Leptochiton cancellatus* (Sowerby G.B. II, 1840)

*Leptochiton cimicoides* (Monterosato, 1879)

*Leptochiton geronensis* Kaas & Van Belle, 1985

*Leptochiton scabridus* (Jeffreys, 1880)

**Hanleyidae**

*Hanleya hanleyi* (Bean in Thorpe, 1844)

**Ichnochitonidae**

*Ischnochiton dolii* Van Belle & Dell'Angelo, 1998

*Ischnochiton rissoi* (Payraudeau, 1826)

*Ischnochiton tsekosi* Koukouras & Karachle, 2005

*Ischnochiton usticensis* Dell'Angelo & Castriota, 1999

*Ischnochiton vanbellei* Kaas, 1985

*Connexochiton platynomenus* Kaas, 1979

*Callistochiton pachylasmae* (Monterosato, 1879)

*Callochiton calcatus* Dell'Angelo & Palazzi, 1994

*Callochiton septemvalvis* (Montagu, 1803)

*Lepidochitona canariensis* (Thiele, 1909)

*Lepidochitona caprearum* (Scacchi, 1836)

*Lepidochitona cinerea* (Linné, 1767)

*Lepidochitona furtiva* (Monterosato 1879)

*Lepidochitona monterosatoi* Kaas & Van Belle, 1981

**Chitonidae**

*Chiton corallinus* (Risso, 1826)

*Chiton hululensis* (Smith E.A., 1903) (E)

*Chiton olivaceus* Spengler, 1797

*Chiton phaseolinus* Monterosato, 1879

**Acanthochitonidae**

*Acanthochitona crinita* (Pennant, 1777)

*Acanthochitona fascicularis* (Linné, 1767)

**GASTROPODA**

DOCOGLOSSA

**Patellidae**

*Patella caerulea* Linné, 1758

*Patella ferruginea* Gmelin, 1791 (P)

*Patella intermedia* Murray in Knapp, 1857

*Patella rustica* Linné, 1758

*Patella ulyssiponensis* Gmelin, 1791

*Ansates pellucida* (Linné, 1758)

*Cymbula nigra* (da Costa, 1771) (P)

**Nacellidae**

*Cellana rota* (Gmelin, 1791) (E)

**Lottiidae**

*Tectura virginea* (Müller O.F., 1776)

**Lepetidae**

*Iothia fulva* (Müller O.F., 1776)

*Propilidium exiguum* (Thompson W., 1844)

*Propilidium pertenue* Jeffreys, 1883

*Propilidium scabrosum* Jeffreys, 1883

VETIGASTROPODA

**Fissurellidae**

*Fissurella nubecula* (Linné, 1758)

*Diodora demartiniorum* Buzzurro & Russo, 2005

*Diodora dorsata* (Monterosato, 1878)

*Diodora gibberula* (Lamarck, 1822)

*Diodora graeca* (Linné, 1758)

*Diodora italica* (Defrance, 1820)

*Diodora producta* (Monterosato, 1880)

*Diodora ruppellii* (Sowerby G.B. I, 1835) (E)

*Emarginula adriatica* Costa O.G., 1829

*Emarginula bonfittoi* Smriglio & Mariottini, 2001

*Emarginula christiaensi* Piani, 1985

*Emarginula divae* van Aartsen & Carrozza, 1995

*Emarginula fissura* (Linné, 1758)

*Emarginula multistriata* Jeffreys, 1882

*Emarginula octaviana* Coen, 1939

*Emarginula punctulum* Piani, 1980

*Emarginula pustula* Thiele in Küster, 1913

*Emarginula rosea* Bell T., 1824

*Emarginula sicula* Gray, 1825

*Emarginula solidula* Costa O.G., 1829

*Emarginula tenera* Locard, 1892

*Emarginula tuberculosa* Libassi, 1859

*Emarginella huzardii* (Payraudeau, 1826)

*Fissurisepta granulosa* Jeffreys, 1883

*Puncturella noachina* (Linné, 1771)

*Puncturella piccirida* Palazzi & Villari, 2001

*Zeidora naufraga* Watson, 1883

**Scissurellidae**

*Scissurella costata* d'Orbigny, 1824

*Anatoma aspera* (Philippi, 1844)

*Anatoma crispata* (Fleming, 1828)

*Anatoma umbilicata* (Jeffreys, 1883)

*Sinezona cingulata* (Costa O.G., 1861)

**Haliotidae**

*Haliotis mykonosensis* Owen, Hanavan & Hall, 2001

*Haliotis pustulata* Reeve, 1846 (E)

*Haliotis stomatiaeformis* Reeve, 1846

*Haliotis tuberculata* Linné, 1758

**Lepetellidae**

*Lepetella barrajoni* Dantart & Luque, 1994

*Lepetella espinosae* Dantart & Luque, 1994

*Lepetella laterocompressa* (de Rayneval & Ponzi, 1854)

*Bogia labronica* (Bogi, 1984)

*Choristella nofronii* McLean, 1992

**Addisoniidae**

*Addisonia excentrica* (Tiberi, 1855)

**Pseudococculinidae**

*Copulabyssia corrugata* (Jeffreys, 1883)

*Pilus conicus* (Verrill, 1884)

**Trochidae**

*Trochus erithraeus* Brocchi, 1821 (E)

*Clanculus corallinus* (Gmelin, 1791)

*Clanculus cruciatus* (Linné, 1758)

*Clanculus jussieui* (Payraudeau, 1826)

*Clelandella miliaris* (Brocchi, 1814)

*Clelandella myriamae* Gofas, 2005

*Callumbonella suturalis* (Philippi, 1836)

*Gibbula adansonii* (Payraudeau, 1826)

*Gibbula adriatica* (Philippi, 1844)

*Gibbula albida* (Gmelin, 1791)

*Gibbula ardens* (Salis, 1793)

*Gibbula cineraria* (Linné, 1758)

Gibbu*la divaricata* (Linné, 1758)

*Gibbula drepanensis* (Brugnone, 1873)

*Gibbula fanulum* (Gmelin, 1791)

*Gibbula guttadauri* (Philippi 1836)

*Gibbula leucophaea* (Philippi, 1836)

*Gibbula magus* (Linné, 1758)

*Gibbula nivosa* Adams A., 1851 (P)

*Gibbula pennanti* (Philippi 1846)

*Gibbula philberti* (Récluz, 1843)

*Gibbula racketti* (Payraudeau, 1826)

*Gibbula rarilineata* (Michaud, 1829)

*Gibbula spratti* (Forbes, 1844)

*Gibbula tantilla* Monterosato, 1890

*Gibbula tingitana* Pallary, 1901

*Gibbula turbinoides* (Deshayes, 1835)

*Gibbula umbilicalis* (da Costa, 1778)

*Gibbula umbilicaris* (Linné, 1758)

*Gibbula varia* (Linné, 1758)

*Gibbula vimontiae* Monterosato, 1884

*Jujubinus baudoni* (Monterosato, 1891)

*Jujubinus catenatus* Ardovini, 2006

*Jujubinus curinii* Bogi & Campani, 2006

*Jujubinus dispar* Curini-Galletti, 1982

*Jujubinus exasperatus* (Pennant, 1777)

*Jujubinus gravinae* (Dautzenberg 1881

*Jujubinus karpathoensis* Nordsieck, 1973

*Jujubinus montagui* (Wood W., 1828)

Jujubinus ruscurianus (Weinkauff, 1868)

*Jujubinus striatus* (Linné, 1758)

*Jujubinus tumidulus* (Aradas, 1846)

*Jujubinus unidentatus* (Philippi, 1844)

*Osilinus articulatus* (Lamarck, 1822)

*Osilinus lineatus* (da Costa, 1778)

*Osilinus mutabilis* (Philippi, 1846)

*Osilinus richardi* (Payraudeau, 1826)

*Osilinus turbinatus* (Born, 1778)

*Stomatella impertusa* (Burrow, 1815) (E)

**Solariellidae**

*Pseudominolia nedyma* (Melvill, 1897) (E)

**Calliostomatidae**

*Calliostoma conulus* (Linné, 1758)

*Calliostoma granulatum* (Born, 1778)

*Calliostoma gualterianum* (Philippi, 1848)

*Calliostoma gubbiolii* Nofroni, 1984

*Calliostoma kochi* Pallary, 1902

*Calliostoma laugieri* (Payraudeau, 1826)

*Calliostoma virescens* Coen, 1933

*Calliostoma zizyphinum* (Linné, 1758)

**Chilodontidae**

*Calliotropis ottoi* (Philippi, 1844)

*Danilia tinei* (Calcara, 1839)

*Putzeysia wiseri* (Calcara, 1842)

**Turbinidae**

*Bolma rugosa* (Linné, 1758)

*Cantrainea peloritana* (Cantraine, 1835)

*Homalopoma carmelae* Oliverio & Buzzurro, 1994

*Homalopoma sanguineum* (Linné, 1758)

*Moelleria costulata* (Møller, 1842)

**Skeneidae**

*Skenea basistriata* (Jeffreys, 1877)

*Skenea catenoides* (Monterosato, 1877)

*Skenea divae* Carrozza & van Aartsen, 2001

*Skenea pelagia* Nofroni & Valenti, 1987

*Skenea serpuloides* (Montagu, 1808)

*Adeuomphalus ammoniformis* Seguenza G., 1876

*Akritogyra conspicua* (Monterosato, 1880)

*Cirsonella romettensis* (Granata-Grillo, 1877)

*Dasyskenea suavis* Fasulo & Cretella, 2002

*Dikoleps cutleriana* (Clark W., 1848)

*Dikoleps depressa* (Monterosato, 1880)

*Dikoleps marianae* Rubio, Dantart & Luque, 1998

*Dikoleps nitens* (Philippi, 1844)

*Dikoleps pruinosa* (Chaster, 1896)

*Dikoleps rolani* Rubio, Dantart & Luque, 1998

*Dikoleps templadoi* Rubio, Dantart & Luque, 2004

*Dikoleps umbilicostriata* (Gaglini, 1987)

*Granigyra granulifera* Warén, 1992

*Lissomphalia bithynoides* (Monterosato, 1880)

*Lissotesta gittenbergeri* (van Aartsen & Bogi, 1988)

*Lissotesta turrita* (Gaglini, 1987)

*Mikro giustii* (Bogi & Nofroni, 1989)

*Moelleriopsis messanensis* (Seguenza G., 1876)

*Palazzia ausonia* (Palazzi, 1988)

*Parviturbo alboranensis* Peñas & Rolán, 2006

*Parviturbo dibellai* Buzzurro & Cecalupo, 2007

*Pseudorbis granulum* (Brugnone, 1873)

*Skeneoides digeronimoi* La Perna 1998

*Skeneoides exilissima* (Philippi, 1844)

*Skeneoides jeffreysii* (Monterosato, 1872)

*Xenoskenea pellucida* (Monterosato, 1874)

**Tricoliidae**

*Tricolia algoidea* (Pallary, 1920)

*Tricolia deschampsi* Gofas, 1993

*Tricolia entomocheila* Gofas, 1993

*Tricolia landinii* Bogi & Campani, 2007

*Tricolia miniata* (Monterosato, 1884)

*Tricolia pullus* (Linné, 1758)

*Tricolia punctura* Gofas, 1993

*Tricolia speciosa* (von Mühlfeld, 1824

*Tricolia tenuis* (Michaud, 1829)

*Tricolia tingitana* Gofas, 1982

**Ataphridae**

*Trochaclis versiliensis* Warén, 1992

**Pendromidae**

*Rugulina monterosatoi* (van Aartsen & Bogi, 1987)

COCCULINIFORMIA

**Cocculinidae**

*Coccopigya viminensis* (Rocchini, 1990)

**Bathysciadiidae**

*Bathysciadium xylophagum* Warén & Carrozzza, 1997

NERITIMORPHA

**Neritidae**

*Nerita sanguinolenta* Menke, 1829 (E)

*Smaragdia souverbiana* (Montrouzier, 1863 (E)

*Smaragdia viridis* (Linné, 1758)

CAENOGASTROPODA

**Cerithiidae**

*Cerithium alucastrum* (Brocchi, 1814)

*Cerithium egenum* Gould 1849 (E)

*Cerithium lividulum* Risso, 1826

*Cerithium nesioticum* Pilsbry & Vanatta, 1906 (E)

*Cerithium protractum* Bivona Ant., 1838

*Cerithium renovatum* Monterosato, 1884

*Cerithium scabridum* Philippi, 1848 (E)

*Cerithium vulgatum* Bruguière, 1792

*Bittium circa* Moreno, 2006

*Bittium incile* Watson, 1897

*Bittium lacteum* (Philippi, 1836)

*Bittium latreillii* (Payraudeau, 1826)

*Bittium reticulatum* (da Costa, 1778)

*Bittium simplex* (Jeffreys, 1867)

*Bittium submamillatum* (de Rayneval & Ponzi, 1854)

*Cassiella abylensis* Gofas, 1987

*Clypeomorus bifasciatus* (Sowerby G.B. II, 1855) (E)

*Rhinoclavis kochi* (Philippi, 1848) (E)

**Dialidae**

*Diala varia* Adams A., 1861 (E)

**Litiopidae**

*Gibborissoa virgata* (Philippi, 1849) (E)

**Scaliolidae**

*Scaliola elata* Issel, 1869) (E)

*Clathrofenella diplax* (Watson, 1886) (E)

*Clathrofenella perparvula* (Watson, 1886) (E)

*Finella pupoides* Adams A., 1860 (E)

**Planaxidae**

*Planaxis savignyi* Deshayes, 1844 (E)

*Angiola punctostriata* Smith E.A., 1872) (E)

*Fossarus ambiguus* (Linné, 1758)

**Potamididae**

*Potamides conicus* (de Blainville, 1829)

**Siliquariidae**

*Petalopoma elisabettae* Schiaparelli, 2002

*Tenagodus obtusus* (Schumacher, 1817)

**Turritellidae**

*Turritella communis* Risso, 1826

*Turritella decipiens* Monterosato, 1878

*Turritella turbona* Monterosato, 1877

*Mesalia varia* (Kiener, 1844)

**Triphoridae**

*Cheirodonta pallescens* (Jeffreys, 1867)

*Cosmotriphora melanura* (Adams C.B., 1850)

*Marshallora adversa* (Montagu, 1803)

*Metaxia bacillum* (Issel, 1869)

*Metaxia metaxae* (Delle Chiaje, 1828)

*Monophorus alboranensis* Rolán & Peñas, 2001

*Monophorus erythrosoma* (Bouchet & Guillemot, 1978)

*Monophorus pantherinus* Rolán & Peñas, 2001

*Monophorus perversus* (Linné, 1758)

*Monophorus thiriotae* Bouchet, 1985

*Obesula marisnostri* Bouchet, 1985

*Pogonodon pseudocanaricus* (Bouchet, 1985)

*Similiphora similior* (Bouchet & Guillemot, 1978)

*Strobiligera brychia* (Bouchet & Guillemot, 1978)

*Strobiligera flammulata* Bouchet & Warén, 1993

**Cerithiopsidae**

*Cerithiopsis annae* Cecalupo & Buzzurro, 2005

*Cerithiopsis barleei* Jeffreys, 1867

*Cerithiopsis diadema* Monterosato, 1874

*Cerithiopsis fayalensis* Watson, 1880

*Cerithiopsis greppii* Buzzurro & Cecalupo, 2005

*Cerithiopsis horrida* Monterosato, 1874

*Cerithiopsis iudithae* Reitano & Buzzurro, 2006

*Cerithiopsis jeffreysi* Watson, 1885

*Cerithiopsis ladae* Prkić & Buzzurro, 2007

*Cerithiopsis minima* (Brusina, 1865)

*Cerithiopsis nofronii* Amati, 1987

*Cerithiopsis perlata* Monterosato, 1889

*Cerithiopsis pulchresculpta* Cachia, Mifsud & Sammut, 2004

*Cerithiopsis pulvis* (Issel, 1869) (E)

*Cerithiopsis scalaris* Locard, 1892

*Cerithiopsis tarruellasi* Peñas & Rolán, 2006

*Cerithiopsis tenthrenois* (Melvill, 1896)

*Cerithiopsis tubercularis* (Montagu, 1803)

*Cerithiella metula* (Lovén, 1846)

*Dizoniopsis coppolae* (Aradas, 1870)

*Dizoniopsis micalii* Cecalupo & Villari, 1997

*Krachia cossmanni* (Dautzenberg & Fischer H., 1896)

*Krachia cylindrata* (Jeffreys, 1885)

*Krachia guernei* (Dautzenberg & Fischer H., 1896)

*Krachia tiara* (Monterosato, 1874)

*Krachiopsis giannuzzii* Smriglio & Mariottini, 1999

*Onchodia valeriae* (Giusti Fr., 1987)

*Seila trilineata* (Philippi, 1836)

**Janthinidae**

*Janthina exigua* Lamarck, 1816

*Janthina globosa* Swainson, 1822

*Janthina janthina* (Linné, 1758)

*Janthina pallida* Thompson W., 1840

**Aclididae**

*Aclis ascaris* (Turton, 1819)

*Aclis attenuans* Jeffreys, 1883

*Aclis gulsonae* (Clark W., 1850)

*Aclis minor* (Brown, 1827)

*Aclis trilineata* Watson, 1897

**Epitoniidae**

*Epitonium algerianum* (Weinkauff, 1866)

*Epitonium brevissimum* (Seguenza G., 1876)

*Epitonium candidissimum* (Monterosato, 1877)

*Epitonium celesti* (Aradas, 1854)

*Epitonium clathratulum* (Kanmacher, 1798)

*Epitonium hispidulum* (Monterosato, 1874)

*Epitonium jolyi* (Monterosato, 1878)

*Epitonium linctum* (de Boury & Monterosato, 1890)

*Epitonium pseudonanum* Bouchet & Warén, 1986

*Epitonium pulchellum* (Bivona Ant., 1832)

*Epitonium striatissimum* (Monterosato, 1878)

*Epitonium tiberii* (de Boury, 1890)

*Epitonium tryoni* (de Boury, 1913)

*Epitonium turtonis* (Turton, 1819)

*Epitonium vittatum* (Jeffreys, 1884)

*Acirsa subdecussata* (Cantraine, 1835)

*Acrilloscala lamyi* (de Boury, 1909)

*Cirsotrema cochlea* (Sowerby G.B. II, 1844)

*Claviscala richardi* (Dautzenberg & de Boury, 1897)

*Cycloscala hyalina* (Sowerby G.B. II, 1844) (E)

*Epidendrium dendrophylliae* (Bouchet & Warén, 1986)

*Gregorioiscala sarsi* (Kobelt, 1904)

*Gyroscala lamellosa* (Lamarck, 1822)

*Iphitus marshalli* (Sykes, 1925)

*Iphitus tuberatus* Jeffreys, 1883

*Narrimania concinna* (Sykes, 1925)

*Opalia abbotti* Clench & Turner, 1952

*Opalia crenata* (Linné, 1758)

*Opalia hellenica* (Forbes, 1844)

*Papuliscala tavianii* Bouchet & Warén, 1986

*Punctiscala cerigottana* (Sturany, 1896)

**Eulimidae**

*Eulima altimirai* Nordsieck, 1977

*Eulima bilineata* Alder, 1848

*Eulima glabra* (da Costa, 1778)

*Eulima fuscozonata* Bouchet & Waren, 1986

*Eulima leptozona* Dautzenberg & Fischer H., 1896

*Auriculigerina miranda* Dautzenberg, 1925

*Bathycrinicola nacraensis* Peñas & Giribet, 2003

*Campylorhaphion famelicum* (Watson, 1883)

*Crinophtheiros comatulicola* (Graff, 1875)

*Crinophtheiros giustii* Gaglini,1991

*Curveulima beneitoi* Peñas & Rolán, 2006

*Curveulima devians* (Monterosato, 1884)

*Entoconcha mirabilis* Müller J., 1852

*Ersilia mediterranea* (Monterosato, 1869)

*Fusceulima minuta* (Jeffreys, 1884)

*Haliella stenostoma* (Jeffreys, 1858)

*Halielloides fragilis* Bouchet & Warén, 1986

*Melanella alba* (da Costa, 1778)

*Melanella boscii* (Payraudeau, 1826)

*Melanella crosseana* (Brusina, 1886)

*Melanella doederleini* (Brusina, 1886)

*Melanella frielei* (Jordan, 1895)

*Melanella glypta* Bouchet & Warén, 1986

*Melanella lineata* (Monterosato, 1869)

*Melanella lubrica* (Monterosato, 1890)

*Melanella microsculpta* Bouchet & Warén, 1986

*Melanella monterosatoi* (Monterosato, 1890)

*Melanella petitiana* (Brusina, 1869)

*Melanella polita* (Linné, 1758)

*Melanella praecurta* (Pallary, 1904)

*Melanella spiridioni* (Dautzenberg & Fischer H., 1896)

*Melanella stalioi* (Brusina, 1869)

*Melanella translucens* (Monterosato, 1890)

*Nanobalcis nana* (Monterosato, 1878)

*Oceanida confluens* Bouchet & Warén, 1986

*Parvioris ibizenca* (Nordsieck, 1968)

*Pelseneeria minor* Koehler & Vaney, 1908

*Sabinella piriformis* Brugnone, 1873

*Sticteulima jeffreysiana* (Brusina, 1869)

*Sticteulima lentiginosa* (Adams A., 1861) (E)

*Vitreolina antiflexa* Monterosato, 1884

*Vitreolina cionella* (Monterosato, 1878)

*Vitreolina curva* (Monterosato, 1874)

*Vitreolina incurva* (Bucquoy, Dautzenberg & Dollfus, 1883)

*Vitreolina levantina* Oliverio, Buzzurro & Villa, 1994

*Vitreolina perminima* (Jeffreys, 1883)

*Vitreolina philippi* (de Rayneval & Ponzi, 1854)

**Littorinidae**

*Littorina littorea* (Linné, 1758)

*Littorina obtusata* (Linné, 1758)

*Littorina saxatilis* (Olivi, 1792)

*Echinolittorina punctata* (Gmelin, 1791)

*Melarhaphe neritoides* (Linné, 1758)

**Skeneopsidae**

*Skeneopsis planorbis* (Fabricius O., 1780)

**Cingulopsidae**

*Eatonina cossurae* (Calcara, 1841)

*Eatonina fulgida* (Adams J., 1797)

*Eatonina ochroleuca* (Brusina, 1869)

*Eatonina pumila* (Monterosato, 1884)

*Tubbreva micrometrica* (Aradas & Benoit, 1876)

**Rissoidae**

*Rissoa aartseni* Verduin, 1985

*Rissoa alleryi* (Nordsieck, 1972)

*Rissoa angustior* (Monterosato, 1917)

*Rissoa auriformis* Pallary, 1904

*Rissoa auriscalpium* (Linné, 1758)

*Rissoa decorata* Philippi, 1846

*Rissoa frauenfeldiana* Brusina, 1866

*Rissoa guerinii* Récluz, 1843

*Rissoa italiensis* Verduin, 1985

*Rissoa lia* (Monterosato, 1884)

*Rissoa monodonta* Philippi, 1836

*Rissoa multicincta* Smriglio & Mariottini, 1995

*Rissoa panhormensis* Verduin, 1985

*Rissoa paradoxa* (Monterosato, 1884)

*Rissoa parva* (da Costa, 1778)

*Rissoa rodhensis* Verduin, 1985

*Rissoa scurra* (Monterosato, 1917)

*Rissoa similis* Scacchi, 1836

*Rissoa splendida* Eichwald, 1830

*Rissoa torquilla* Pallary, 1912

*Rissoa variabilis* (von Mühlfeld, 1824)

*Rissoa ventricosa* Desmarest, 1814

*Rissoa violacea* Desmarest, 1814

*Alvania aartseni* Verduin, 1986

*Alvania aeoliae* Palazzi, 1988

*Alvania alboranensis* Peñas & Rolán, 2006

*Alvania algeriana* (Monterosato, 1877)

*Alvania amatii* Oliverio, 1986

*Alvania aspera* (Philippi, 1844)

*Alvania balearica* Oliver & Templado, 2009

*Alvania beani* (Hanley in Thorpe, 1844)

*Alvania bicingulata* (Seguenza L., 1903)

*Alvania cancellata* (da Costa, 1778)

*Alvania carinata* (da Costa, 1778)

*Alvania cimex* (Linné, 1758)

*Alvania cimicoides* (Forbes, 1844)

*Alvania cingulata* (Philippi, 1836)

*Alvania clarae* Nofroni & Pizzini, 1991

*Alvania clathrella* (Seguenza L., 1903)

*Alvania claudioi* Buzzurro & Landini, 2007

*Alvania colossophilus* Oberling, 1970

*Alvania consociella* Monterosato, 1884

*Alvania corona* Nordsieck, 1972

*Alvania dalmatica* Buzzurro & Prkić, 2007

*Alvania datchaensis* Amati & Oliverio, 1987

*Alvania dianiensis* Oliverio, 1988

*Alvania dictyophora* (Philippi, 1844)

*Alvania dipacoi* Giusti Fr. & Nofroni, 1989

*Alvania discors* (Allan, 1818)

*Alvania disparilis* Monterosato, 1890

*Alvania dorbignyi* (Audouin, 1826) (E)

*Alvania electa* (Monterosato, 1874)

*Alvania elegantissima* (Monterosato, 1875)

*Alvania elisae* Margelli, 2001

*Alvania fischeri* (Jeffreys, 1884)

*Alvania fractospira* Oberling, 1970

*Alvania gagliniae* Amati, 1985

*Alvania garrafensis* Peñas & Rolán, 2008

*Alvania geryonia* (Nardo, 1847)

*Alvania hallgassi* Amati & Oliverio, 1985

*Alvania hirta* (Monterosato, 1884)

*Alvania hispidula* (Monterosato, 1884)

*Alvania imperspicua* (Pallary, 1920)

*Alvania josefoi* Oliver & Templado, 2009

*Alvania lactea* (Michaud, 1830)

*Alvania lanciae* (Calcara, 1845)

*Alvania lineata* Risso, 1826

*Alvania litoralis* (Nordsieck, 1972)

*Alvania lucinae* Oberling, 1970

*Alvania mamillata* Risso, 1826

*Alvania nestaresi* Oliverio & Amati, 1990

*Alvania oliverioi* Buzzurro, 2003

*Alvania pagodula* (Bucquoy, Dautzenberg & Dollfus, 1884)

*Alvania parvula* (Jeffreys, 1884)

*Alvania punctura* (Montagu, 1803)

*Alvania rudis* Philippi, 1844)

*Alvania scabra* (Philippi, 1844)

*Alvania schwartziana* Brusina, 1866

*Alvania sculptilis* (Monterosato, 1877)

*Alvania settepassii* Amati & Nofroni, 1985

*Alvania simulans* Locard, 1886

*Alvania sleursi* (Amati, 1987)

*Alvania sororcula* Granata-Grillo, 1877

*Alvania spinosa* (Monterosato, 1890)

*Alvania subareolata* Monterosato, 1869

*Alvania subcrenulata* (Bucquoy, Dautzenberg & Dollfus, 1884)

*Alvania subsoluta* (Aradas, 1847)

*Alvania tenera* (Philippi, 1844)

*Alvania tessellata* Weinkauff, 1868

*Alvania testae* (Aradas & Maggiore, 1844)

*Alvania vermaasi* van Aartsen, 1975

*Alvania villarii* Micali, Tisselli & Giunchi, 2005

*Alvania weinkauffi* Weinkauff, 1868

*Alvania zetlandica* (Montagu, 1815)

*Alvania zylensis* Gofas & Warén, 1982

*Benthonella tenella* (Jeffreys 1869)

*Botryphallus epidauricus* (Brusina 1866)

*Cingula trifasciata* (Adams J., 1800)

*Crisilla amphiglypha* Bouchet & Warén, 1993

*Crisilla beniamina* (Monterosato, 1884)

*Crisilla chiarellii* (Cecalupo & Quadri, 1995)

*Crisilla iunoniae* (Palazzi, 1988)

*Crisilla marioni* (Fasulo & Gaglini, 1987)

*Crisilla pseudocingulata* (Nordsieck, 1972)

*Crisilla semistriata* (Montagu, 1808)

*Crisilla spadix* (Watson, 1897)

*Manzonia crassa* (Kanmacher, 1798

*Obtusella intersecta* (Wood S., 1857)

*Obtusella macilenta* (Monterosato, 1880)

*Obtusella ovulata* (Nordsieck, 1972)

*Onoba aculeus* (Gould, 1841)

*Onoba dimassai* Amati & Nofroni, 1991

*Onoba gianninii* (Nordsieck, 1974)

*Onoba oliverioi* Smriglio & Mariottini, 2000

*Onoba semicostata* (Montagu, 1803)

*Peringiella denticulata* Ponder, 1985

*Peringiella elegans* (Locard, 1892)

*Plagyostila asturiana* Fischer P. in de Folin, 1872

*Pseudosetia ficaratiensis* (Brugnone, 1876)

*Pusillina benzi* (Aradas & Maggiore, 1844)

*Pusillina ehrenbergi* (Philippi, 1844)

*Pusillina inconspicua* (Alder, 1844)

*Pusillina lineolata* (Michaud, 1830)

*Pusillina marginata* (Michaud, 1830)

*Pusillina munda* (Monterosato, 1884)

*Pusillina philippi* (Aradas & Maggiore, 1844)

*Pusillina radiata* (Philippi, 1836)

*Pusillina sarsii* (Lovén, 1846)

*Pusillina testudae* (Verduin, 1979)

*Rissoina bertholleti* Issel, 1869 (E)

*Rissoina bruguieri* (Payraudeau, 1826)

*Setia alboranensis* Peñas & Rolán, 2006

*Setia amabilis* (Locard, 1886)

*Setia ambigua* (Brugnone, 1873)

*Setia antipolitana* (van der Linden & Wagner, 1987)

*Setia bruggeni* (Verduin, 1984)

*Setia fusca* (Philippi, 1841)

*Setia kuiperi* Verduin, 1984)

*Setia levantina* Bogi & Galil, 2007

*Setia maculata* (Monterosato, 1869)

*Setia scillae* (Aradas & Benoit, 1876)

*Setia turriculata* Monterosato, 1884

*Voorwindia tiberiana* (Issel 1869) (E)

**Anabathridae**

*Nodulus contortus* (Jeffreys, 1856)

*Pisinna glabrata* (Megerle von Mühlfeld, 1824)

**Assimineidae**

*Assiminea gittenbergeri* van Aartsen, 2008

*Assiminea grayana* Fleming, 1828

*Paludinella littorina* (Delle Chiaje 1828)

*Paludinella sicana* (Brugnone 1876)

**Barleeidae**

*Barleeia gougeti* (Michaud, 1830)

*Barleeia seminulum* (Monterosato, 1877)

*Barleeia unifasciata* (Montagu, 1803)

**Caecidae**

*Caecum armoricum* de Folin, 1869

*Caecum auriculatum* de Folin, 1868

*Caecum clarkii* Carpenter, 1859

*Caecum glabrum* (Montagu, 1803)

*Caecum subannulatum* de Folin, 1870

*Caecum trachea* (Montagu, 1803)

*Parastrophia asturiana* de Folin, 1870

**Elachisinidae**

*Laeviphitus verduini* van Aartsen, Bogi & Giusti, 1989

**Hydrobiidae**

*Hydrobia acuta* (Draparnaud, 1805)

*Hydrobia djerbaensis* Wilke, Pfenninger & Davis, 2002

*Ventrosia maritima* (Milaschewitsch, 1916)

*Ventrosia ventrosa* (Montagu, 1803)

*Heleobia stagnorum* (Gmelin, 1791)

**Iravadiidae**

*Ceratia proxima* (Thompson, 1850)

*Hyala vitrea* (Montagu, 1803)

**Tornidae**

*Tornus jullieni* Adam & Knudsen, 1969

*Tornus mienisi* van Aartsen, Carrozza & Menkhorst, 1998

*Tornus subcarinatus* (Montagu, 1803)

*Circulus striatus* (Philipp,i 1836)

*Circulus tricarinatus* (Wood S., 1848)

*Discopsis costulatus* de Folin, 1870

**Truncatellidae**

*Truncatella subcylindrica* (Linné, 1767)

**Vermetidae**

*Vermetus granulatus* (Gravenhorst, 1831)

*Vermetus rugulosus* Monterosato, 1878

*Vermetus semisurrectus* Bivona Ant., 1832

*Vermetus triquetrus* Bivona Ant., 1832

*Dendropoma anguliferum* (Monterosato, 1884)

*Dendropoma petraeum* (Monterosato, 1884) (P)

*Petaloconchus glomeratus* (Linné, 1758)

*Serpulorbis arenarius* (Linné, 1767)

**Strombidae**

*Strombus mutabilis* Swainson, 1821 (E)

*Strombus persicus* Swainson, 1821 (E)

**Aporrhaidae**

*Aporrhais pespelecani* (Linné, 1758)

*Aporrhais serresianus* (Michaud, 1828)

**Vanikoridae**

*Macromphalus abylensis* Warén & Bouchet, 1988

*Megalomphalus azonus* (Brusina, 1865)

*Megalomphalus disciformis* (Granata-Grillo, 1877)

*Megalomphalus petitianus* (Tiberi, 1869)

*Talassia dagueneti* (de Folin, 1873)

**Hipponicidae**

*Sabia conica* (Schumacher, 1817) (E)

**Xenophoridae**

*Xenophora crispa* (Koenig, 1825)

**Calyptraeidae**

*Calyptraea chinensis* (Linné, 1758)

*Crepidula aculeata* (Gmelin, 1791) (E)

*Crepidula fornicata* (Linné, 1758) (E)

*Crepidula moulinsii* Michaud, 1829

*Crepidula unguiformis* Lamarck, 1822

**Capulidae**

*Capulus ungaricus* (Linné, 1758)

*Torellia delicata* (Philippi, 1844)

**Velutinidae**

*Velutina undata* Smith J., 1839

*Velutina velutina* (Müller O.F., 1776)

*Lamellaria latens* (Müller O.F., 1776)

*Lamellaria perspicua* (Linné, 1758)

**Triviidae**

*Trivia arctica* (Pulteney, 1799)

*Trivia bitou* Pallary, 1912

*Trivia levantina* Smriglio, Mariottini & Buzzurro, 1998

*Trivia monacha* (da Costa, 1778)

*Trivia multilirata* (Sowerby G.B. II, 1870)

*Trivia pulex* (Solander, 1828)

*Trivia spongicola* Monterosato, 1923

*Erato voluta* (Montagu, 1803)

**Cypraeidae**

*Erosaria spurca* (Linné, 1758) (P)

*Erosaria turdus* (Lamarck, 1810) (E)

*Luria lurida* (Linné, 1758) (P)

*Purpuradusta gracilis notata* (Gill, 1858) (E)

*Palmadusta lentiginosa* (Gray, 1825) (E)

*Schilderia achatidea* (Gray in Sowerby G.B. II, 1837) (P)

*Zonaria pyrum* (Gmelin, 1791) (P)

**Ovulidae**

*Aperiovula adriatica* (Sowerby G.B. I, 1828

*Neosimnia illyrica* Schilder, 1927

*Neosimnia spelta* (Linné, 1758)

*Pseudosimnia carnea* (Poiret, 1789)

*Simnia nicaeensis* Risso, 1826

*Simnia purpurea* Risso, 1826

*Pedicularia sicula* Swainson, 1840

**Naticidae**

*Natica hebraea* (Martyn, 1784)

*Natica prietoi* Hidalgo, 1873

*Natica stercusmuscarum* (Gmelin, 1791)

*Natica vittata* (Gmelin, 1791)

*Cryptonatica operculata* (Jeffreys, 1885)

*Euspira catena* (da Costa, 1778)

*Euspira fusca* (de Blainville, 1825)

*Euspira grossularia* (Marche-Marchad, 1957)

*Euspira guillemini* (Payraudeau, 1826)

*Euspira macilenta* (Philippi, 1844

*Euspira pulchella* (Risso, 1826)

*Neverita josephinia* Risso, 1826

*Notocochlis dillwynii* (Payraudeau, 1826)

*Notocochlis gualteriana* (Récluz, 1844)

*Payraudeautia intricata* (Donovan, 1804)

*Polinices lacteus* (Guilding, 1834)

*Sinum bifasciatum* (Récluz, 1851)

*Tectonatica rizzae* (Philippi, 1844)

*Tectonatica sagraiana* (d'Orbigny, 1842)

**Tonnidae**

*Tonna galea* (Linné, 1758) (P)

*Eudolium bairdii* (Verrill & Smith, 1881)

*Eudolium crosseanum* (Monterosato, 1869)

*Galeodea echinophora* (Linné, 1758)

*Galeodea rugosa* (Linné, 1771)

*Oocorys sulcata* Fischer P., 1883

*Phalium granulatum* (Born, 1778)

*Phalium saburon* (Bruguière, 1792)

**Ranellidae**
[truncated: 463,757 more chars]
